# Aminocatalytic 1,6-Addition of 2‑Benzyl-3-furaldehyde to 3‑Cyano-4-styrylcoumarins: A Dearomative Approach for the Synthesis of Furan–Coumarin Hybrids

**DOI:** 10.1021/acs.joc.5c01406

**Published:** 2025-10-11

**Authors:** Aleksandra Topolska, Artur Przydacz, Lesław Sieroń, Anna Skrzyńska, Alberto Fraile, Jose Alemán, Łukasz Albrecht

**Affiliations:** † Institute of Organic Chemistry, Faculty of Chemistry, 49584 Lodz University of Technology, Żeromskiego 116, 90-924 Łódź, Poland; ‡ Institute of General and Ecological Chemistry, Faculty of Chemistry, Lodz University of Technology, Żeromskiego 116, 90-924 Łódź, Poland; § Organic Chemistry Department, 16722 Universidad Autónoma de Madrid, 28049 Madrid, Spain; ∥ Institute for Advanced Research in Chemical Sciences (IAdChem), Universidad Autónoma de Madrid, 28049 Madrid, Spain

## Abstract

In the manuscript, the application of temporary dearomatization of 2-benzyl-3-furaldehyde under aminocatalytic conditions in a 1,6-addition pathway is described. In such reaction setup catalytically generated dienamine derived from heteroaromatic aldehydes reacts with coumarin derivatives in 1,6-addition. Developed approach utilizes a catalytic system consisting of an aminocatalyst and an acidic cocatalyst, which is crucial for the reaction efficiency. The reaction displays a wide substrate scope generating target products containing furan and coumarin moieties.

## Graphical abstract

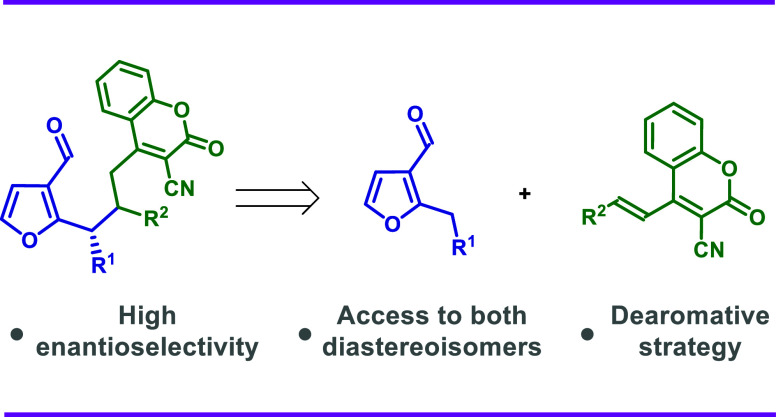

## Introduction

Dearomative processes that proceed with temporary or permanent disturbance of the (hetero)­aromatic character of starting materials are of key importance in the contemporary organic synthesis.^1–5^ They constitute a valuable synthetic tool for the functionalization of (hetero)­aromatic compounds opening new routes to the products with intriguing biological properties.^6^ The utilization of *ortho*- or *para*-quinodimethanes constitutes an interesting approach to accomplish this task.^7,8^ In particular, the evolution of aminocatalysis enabled generation of such synthetic intermediates from heteroaromatic carbonyl compounds in the presence of suitable aminocatalyst, introducing new methods for their functionalization (top, Scheme 1).^9,10^ Their attractiveness has been confirmed in a wide range of new reactions proceeding in a highly chemo-, regio- and stereo-selective manner. While dearomative 1,4-additions or cascade reactions of furfural derivatives are known, their applications in more demanding 1,6-type additions in an asymmetric fashion are barely established.^11–15^ This concept of aminocatalytic dearomative functionalization of furan derivatives has been developed by various authors, including reactions with nitroalkenes,^12^ 2-nitroallylic acetates,^13^ azadiene derivatives,^14^ or 4-alkylidene-2,6-dialkylcyclohexa-2,5-dienone (Scheme 1, bottom) .^15^ To the best of our knowledge, the use of these reactions with 4-styrylcoumarins have not been carried out.

**Scheme 1:**
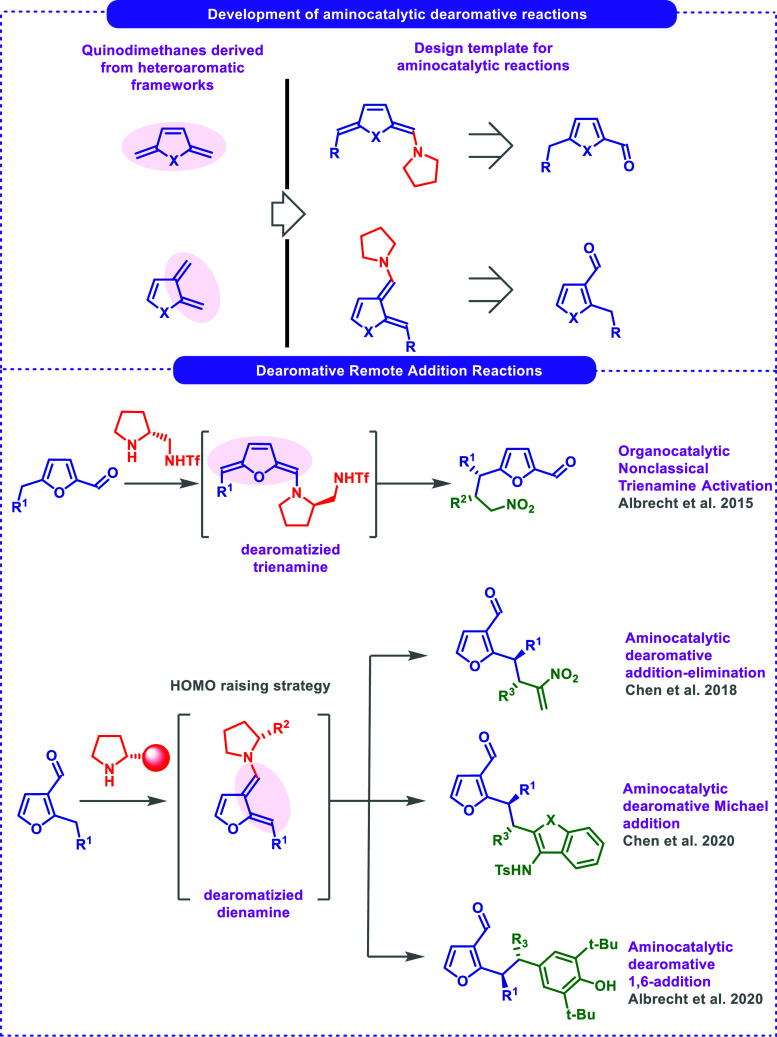
Polyenamine-Mediated Dearomative Functionalization of Heteroaromatic Aldehydes—Available Activation Modes

Coumarin and its derivatives are organic compounds of great significance in chemistry and biology due to their wide range of pharmaceutical, agrochemical, and industrial applications. These coumarin-derived molecules exhibit antioxidant, anti-inflammatory, antimicrobial, and anticoagulant properties, making them key components in the development of drugs for treating various diseases, including cardiovascular disorders and cancer.^16^ Therefore, the search for new coumarin-based hybrid compounds and their selective reactions at various reactive positions is very important. Notably, functionalizations of coumarin derivatives have been achieved with electrophiles,^17^ radical species,^18^ nucleophilic species,^19^ or via deprotonation at their allylic position (Scheme 2, top).^20^ Recently, we became interested in the development of new strategies for the remote functionalization of 4-(alk-1-en-1-yl)-3-cyanocoumarins **2**. We have demonstrated new synthetic opportunities arising from their application as vinylogous Michael acceptors confirming that these systems constitute interesting building blocks for the synthesis of coumarin derivatives.^21^ However, their dearomative processes with heteroaromatic aldehydes have not been accomplished.

**Scheme 2:**
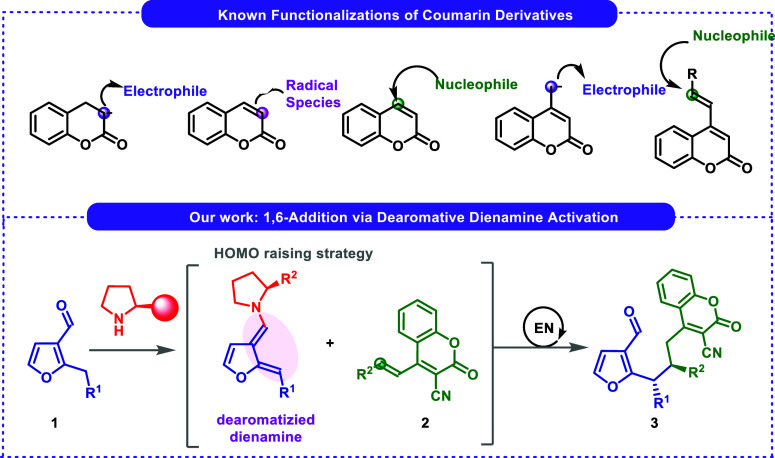
Different Functionalizations of Coumarin Derivatives and Our Approach via Dearomative Dienamine Activation

Herein, we present our studies on the application of 4-(alk-1-en-1-yl)-3-cyanocoumarins **2** in the aminocatalytic reaction with the heteroaromatic aldehydes **1** (Scheme 2, bottom). The developed approach relies on dearomative 1,6-addition and proceeds efficiently using secondary amines **4** as catalysts, providing enantioenriched products **3**.

## Results and Discussion

Our investigation studies were focused on the dearomative 1,6-addition of 2-benzylfuran-3-carbaldehyde **1a** to 2-oxo-4-styryl-2*H*-chromene-3-carbonitrile **2a** as model substrates in the presence of various aminocatalysts **4**. The initial experiments, performed with secondary amines **4a–e** as catalysts in dichloromethane at room temperature or at 40 °C, did not result in the formation of the product **3a** (Table 1, entry 1). Thus, the progress of the reaction with catalysts **4a–e** in the presence of an acidic cocatalyst as an additive was examined (Table 1, entries 2–6). Delightfully, utilization of *o*-fluorobenzoic acid (*o*-FBA) resulted in the formation of the desired product **3a**, but the efficiency of the reaction was not satisfying. The application of secondary amine **4a** provided only traces of **3a** (Table 1, entry 2). In the presence of Hayashi–Jørgensen catalyst **4b**, target product **3a** was obtained with 72% conversion but with low diastereoselectivity (Table 1, entry 3). Noteworthily, the diastereoisomers were separated by FC. The enantiomeric excess for the major diastereoisomer was excellent (>99:1 er) but the minor diastereoisomer was formed as a racemic mixture. Reaction with catalyst **4c** led to **3a** with 65% conversion and 2.2:1 dr but the enantiomeric excess for major diastereoisomer was lower (97:3 er) and the minor diastereoisomer was again obtained as a racemic mixture (Table 1, entry 4). The utility of **4d** bearing spatially demanding *tert*-butyldimethylsilyl group (TBDMS) did not lead to better results (Table 1, entry 5). To our delight, the use of catalyst **4e** protected with diphenylmethylsilyl group (DPMS) significantly improved enantiomeric excess for both major and minor diastereoisomers (Table 1, entry 6). Nevertheless, the conversion rate was unsatisfactory. Therefore, cocatalysts screening was performed (Table 1, entries 7–8). The use of 4-(dimethylamino)­benzoic acid (DMABA) or benzoic acid did not improve the process. Thus, the effect of the increased amount of catalyst and reaction time in the reaction was studied. The usage of 30 mol % of aminocatalyst **4e** and the prolongation of the reaction time from 48 h to 72 h significantly improved the conversion of substrates. However, the diastereoselectivity was still low (Table 1, entry 9). Therefore, the concentration of the reaction mixture was checked (Table 1, entry 10). The change of concentration of the reaction mixture to 0.05 M provided target **3a** with the 80% yield, 2.2:1 dr, and high enantioselectivity for both diastereoisomers, indicating the final reaction conditions. Additionally, the efficiency of the process under the optimized reaction conditions was confirmed by 1 mmol scale experiment (Table 1, entry 11). The product **3a** was formed with similar results confirming good scalability of the developed method.

**Table 1:**
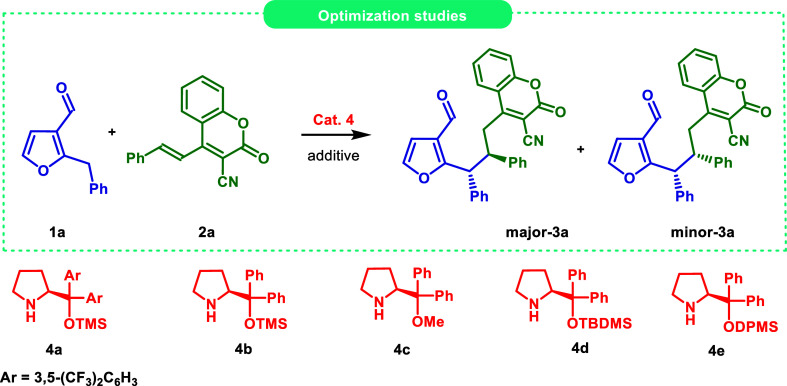
Enantioselective 1,6-Addition of 2-Alkyl-3-furfurals **1** to 3-Cyano-4-styrylcoumarins **2** via Dearomative Dienamine Activation—Optimization Studies^a^

	catalyst	additive	conv.^b^ (%)	dr^c^	er^d^
1	**4a–4e**				
2	**4a**	*o*-FBA	<5		
3	**4b**	*o*-FBA	72	1.5:1	>99:1/rac
4	**4c**	*o*-FBA	65	2.2:1	97:3/rac
5	**4d**	*o*-FBA	90	1.1:1	97:3/rac
6	**4e**	*o*-FBA	40	3.2:1	99:1/98:2
7	**4e**	PhCO_2_H	23	3.4:1	
8	**4e**	DMABA	<5		
9^e^ ^,^ ^f^	**4e**	*o*-FBA	74	2:1	>99:1/98:2
**10** ^e^ ^,^ ^f^ ^,^ ^g^	**4e**	***o*-FBA**	**90 (80)**	**2.2**:**1**	**>99:1/98:2**
**11** ^h^	**4e**	***o*-FBA**	**90 (74)**	**2.2**:**1**	**>99:1/98:2**

a The reactions were performed in 0.05 mmol scale using **1a** (1.0 equiv), **2a** (1.0 equiv), **4** (20 mol %) and additive (40 mol %) in CH_2_Cl_2_ (0.2 mL) at 40 °C.

b Determined by ^1^H NMR of a crude reaction mixture after 48 h. In parentheses isolated yield is given (%).

c Determined by ^1^H NMR of a crude reaction mixture.

d Determined by UPC^2^.

e Reaction performed for 72 h.

f Reaction performed using catalyst **4e** (30 mol %).

g Reaction performed in CH_2_Cl_2_ (0.05 M).

h Reaction performed on 1 mmol scale using **4e** (30 mol %) and *o*-FBA (40 mol %) in CH_2_Cl_2_ (0.05 M) for 72 h. DMABA: 4-(dimethylamino)­benzoic acid; *o*-FBA: *ortho*-fluorobenzoic acid.

Having accomplished optimization studies, the substrate scope of the dearomative 1,6-addition was evaluated under the optimized conditions (Scheme 3). Each of the target products **3a–s** was obtained with moderate diastereoselectivity but the diastereoisomers were readily separated using flash chromatography (FC). First, structurally different 4-styryl-2*H*-chromene-3-carbonitrile derivatives **2** were employed (Scheme 3, top). The presence of the electron-withdrawing substituents on the styryl aromatic ring in **2** led to final product **3b–g** with moderate to good yields, and excellent enantioselectivity. The reaction with chloro-substituted (**2c** and **2d**) in *ortho*- or *meta*-position afforded the chromene-carbonitrile derivative **3c** and **3d** with very good results. The presence of electron-donating methyl substituent in either *meta*- or *para*-positions in **2** allowed to obtain optically active products **3h–i**, respectively, in high yields. However, the introduction of methoxy group in **2j** resulted in formation of **3j** with diminished yield and enantioselectivity (97:3 er for major and 90:10 er for minor diastereoisomers). The reaction proceeded with moderate yield for **2k** bearing more sterically demanding 2-naphthyl group. The modification of the coumarin ring in **2** was also evaluated. Incorporation of bromine or methoxy substituents on the coumarin ring in **2** gave access to products **3l–m** with good yields and excellent enantioselectivity for both diastereoisomers. Additionally, the use of 4-alkyl-2*H*-chromene-3-carbonitrile derivative **2n** in the synthesis of furan–coumarin hybrids was evaluated. The reaction between **1a** and **2n** led to final product **3n** with high yield and excellent enantioselectivity.

**Scheme 3:**
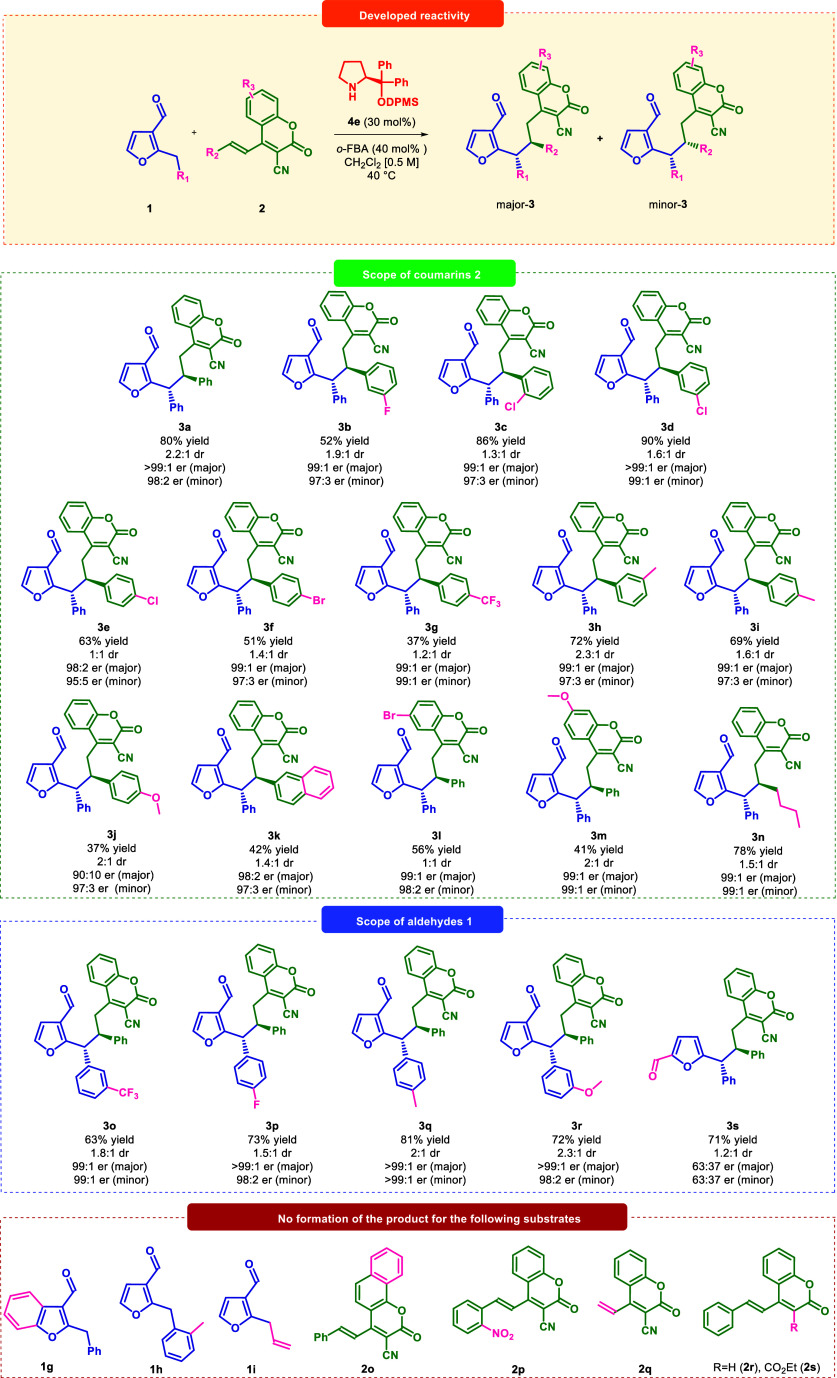
Enantioselective 1,6-Addition of 2-Alkyl-3-furfurals **1** to 3-Cyano-4-styrylcoumarins **2** via Dearomative Dienamine Activation—Scope and Limitation of the Developed Process

Then, the scope of heteroaromatic aldehydes **1** was studied (Scheme 3, middle). The presence of electron-withdrawing groups in the *meta*- or *para*-position of the aromatic ring in **1** led to formation of the derivatives **3o**,**p** with excellent yield and enantioselectivity. The introduction of methyl-group on the aromatic ring in **1d** gave access to optically active **3q** with satisfactory results. Similarly, the presence of EDG group in the *meta*-position of **1e** did not change the reaction outcome, providing **3r** in very good results. Interestingly, 5-benzylfurfural **1f** was also employed in the 1,6-addition with the corresponding substrate **2a** providing **3s** but with decreased enantioselectivity. Unfortunately, this reaction has also its limitations (Scheme 3, bottom). 2-Benzylbenzofuran-3-carbaldehyde **1g**, 2-furan-3-carbaldehyde **1h**, substituted with methyl group in *ortho*-position, and furan-3-carbaldehyde **1l** **,** which lacks the phenyl ring did not exhibit the desired reactivity. The possible explanation is the difficulty in the formation of key dearomatized intermediate due to electronic (**1g** and **1l**) or steric (**1h**) reasons. On the other side, the modification of the coumarin backbone with additional phenyl ring in **2o** also did not afford the target product. Reactivity of substrates **2p**, decorated with electron-withdrawing –NO_2_ group, and **2q** with styryl group replaced by vinyl moiety was also examined, but no target compound was observed in both cases. What is more, experiments with coumarin substrate without the nitrile moiety as in **2r** or with its substitution by a carboester moiety in **2s** were performed. Under the optimized conditions, reactions did not take place, which confirmed that the cyano group in the 3-position was essential for the developed dearomative reaction.

In the last step of the research the synthetic utility of the products **3** was investigated (Scheme 4). First, the aldehyde group of minor-**3i** was transformed into 1,1-dibromomethylidene group using Ramirez olefination approach. The reaction proceeded smoothly giving product **5** with high yield. Alternatively, a new six-membered ring was formed by means of vinylogous intermolecular aldol condensation under basic conditions. Interestingly, the annulation was accompanied by the decyanation of coumarin ring which most likely proceeded in a sequence involving hydrolysis of the nitrile to carboxylic acid moiety followed by oxa-Michael addition of water and its decarboxylative elimination. Product **6** was thus obtained in good yield as a single diastereoisomer.

**Scheme 4:**
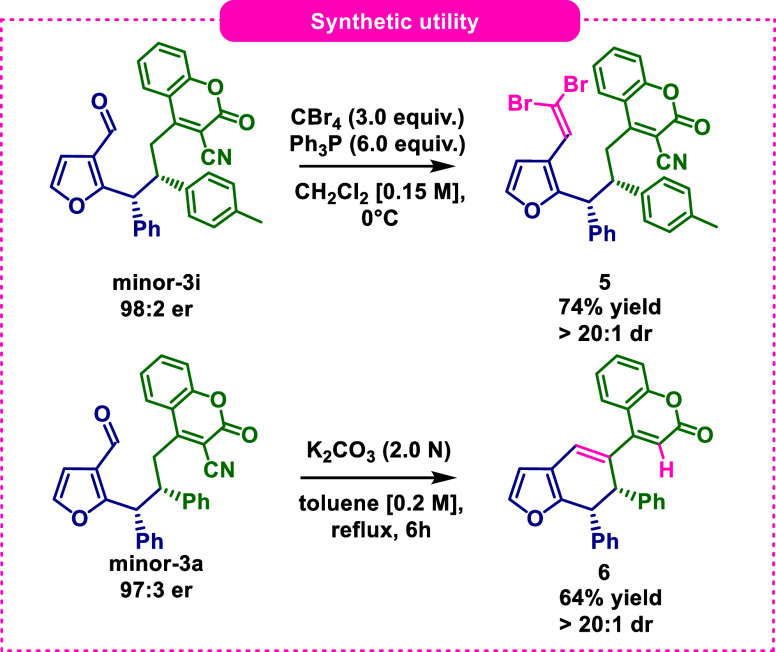
Synthetic Utility of Products **3**—Ramirez Olefination (Top) and Intermolecular Vinylogous Aldol Condensation—Decyanation Sequence (Bottom)

Finally, the absolute configuration of the major-**3a** and the minor-**3d** were unambiguously determined by the single-crystal X-ray crystallographic analysis (see Supporting Information for further details).^22^ The stereochemistry of all other products **3a**–**s** was assigned by analogy. The diastereoisomers differ in configuration of stereocenters that originated from electrophilic carbon atoms of coumarin substrate **2**. This is in line with our predictions based on previous DFT studies on the stability and reactivity of intermediate **8**.^15,23^ The dearomatized dienamine **7** exhibits *Z* configuration of benzylidene moiety exclusively, hence the diastereoselectivity is decided by the facial selectivity of its addition to the coumarin **2**. Given the results obtained, a possible mechanism was proposed (Scheme 5). The catalytic cycle was initiated by the condensation of aminocatalyst **4e** with heteroaromatic aldehyde **1**, forming the iminium ion **7**. The *o*-FBA is acting as a cocatalyst, enabling faster condensation of the catalyst with aldehyde **1**. Subsequent deprotonation of benzyl position in intermediate **7** yielded dearomative dienamine **8**, which was involved in the 1,6-addition reaction with **9**—additional activation of **2** via protonation is of key importance. The resulting adduct **10** underwent hydrolytic catalyst cleavage, giving access to the target product **3**. Enantioselectivity of the reaction is governed by the aminocatalyst **4e** directing the approach of Michael acceptor from the side opposite to the bulky substituent present on the pyrrolidine ring of the dienamine intermediate. Moderate diastereoselection arises from the two plausible arrangements of the Michael acceptor with relation to the dienamine intermediate.

**Scheme 5:**
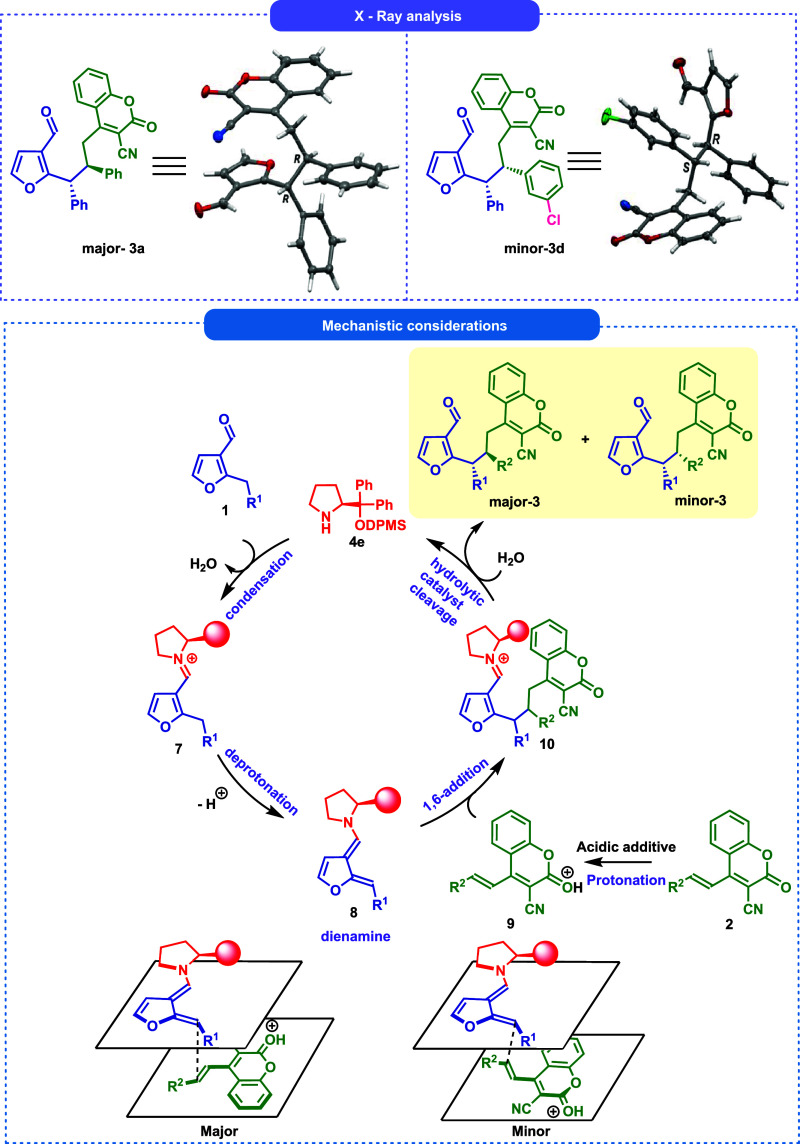
Enantioselective 1,6-Addition of 2-Alkyl-3-furfurals **1** to 3-Cyano-4-styrylcoumarins **2** via Dearomative Dienamine Activation—Postulated Catalytic Cycle

## Conclusions

In summary, we have developed a strategy for the enantioselective construction of optically active products **3** bearing two stereogenic centers. The reaction between dearomative dienamine **7** derived from heteroaryl aldehydes **1** and 3-cyano-4-styrylcoumarins **2** proceeded via a 1,6-addition and was realized under aminocatalytic conditions. The scope of the substrates was explored, and scale-up experiments were conducted.

## Experimental Section

### General Procedure for the Synthesis of **3**

In an ordinary 4 mL glass vial equipped with a magnetic stirring bar 2-benzyl-3-furfural **1** (0.1 mmol, 1.0 equiv) and 4-(alk-1-en-1-yl)-3-cyanocoumarin **2** (0.1 mmol, 1.0 equiv) were dissolved in CH_2_Cl_2_ (0.2 mL). (*S*)-2-(((Methyldiphenylsilyl)­oxy)­diphenylmethyl)­pyrrolidine **4e** (13.5 mg, 0.03 mmol, 0.3 equiv) and *o*-fluorobenzoic acid (5.6 mg, 0.04 mmol, 0.4 equiv) were added and the reaction mixture was stirred in 40 °C for 72 h. The progress of the reaction was controlled by ^1^H NMR spectroscopy. After full conversion of the starting material **1**, the reaction mixture was directly subjected to column chromatography on silica gel (hexanes/ethyl acetate 4:1 to 3:2) to afford pure product major-**3** and minor-**3**.

**3a** (2.2:1 dr in a crude reaction mixture) was isolated after 3 days in 80% yield (36.7 mg).

#### 4-((2*R*,3*R*)-3-(3-Formylfuran-2-yl)-2,3-diphenylpropyl)-2-oxo-2*H*-chromene-3-carbonitrile Major-**3a**

Light-yellow oil (25.2 mg). The er was determined by UPC^2^ using a chiral Chiralpak IB column gradient from 100% CO_2_ up to 40%; *i*-PrOH, 2.5 mL/min; *t*_R_ = 4.491 (major), *t*_R_ = 4.761 (minor): >99:1 er. [α]_D_^21^ = 29.5 (*c* = 1.0, CHCl_3_). HRMS *m*/*z*: [M + H]^+^ calculated for [C_30_H_21_NO_4_ + H^+^], 459.1470; found, 459.1538. ^1^H NMR (700 MHz, CDCl_3_): δ 10.09 (s, 1H), 7.65 (t, *J* = 7.8 Hz, 1H), 7.59 (d, *J* = 7.8 Hz, 1H), 7.51 (d, *J* = 1.9 Hz, 1H), 7.38–7.34 (m, 1H), 7.32 (d, *J* = 7.8 Hz, 1H), 7.25–7.22 (m, 2H), 7.13–7.11 (m, 2H), 7.10–7.08 (m, 2H), 7.07–7.04 (m, 2H), 7.03–7.00 (m, 2H), 6.77 (d, *J* = 1.9 Hz, 1H), 5.24 (d, *J* = 11.0 Hz, 1H), 4.19–4.11 (m, 1H), 3.47–3.41 (m, 1H), 3.34–3.29 (m, 1H). ^13^C NMR (176 MHz, CDCl_3_): δ 185.1, 163.5, 161.9, 156.5, 153.4, 142.9 (2C), 138.3, 135.0, 128.8 (2C), 128.7 (2C), 128.5 (2C), 128.3 (2C), 127.9, 127.4, 126.0, 125.3, 123.6, 118.0, 117.6, 113.4, 110.2, 103.0, 50.4, 49.6, 37.7.

#### 4-((2*S*,3*R*)-3-(3-Formylfuran-2-yl)-2,3-diphenylpropyl)-2-oxo-2*H*-chromene-3-carbonitrile Minor-**3a**

Light-yellow oil (11.5 mg). The er was determined by UPC^2^ using a chiral Chiralpak IB column gradient from 100% CO_2_ up to 40%; *i*-PrOH, 2.5 mL/min; *t*_R_ = 4.835 (major), *t*_R_ = 4.634 (minor): 98:2 er. [α]_D_^21^ = −54.7 (*c* = 1.0, CHCl_3_). HRMS *m*/*z*: [M + H]^+^ calculated for [C_30_H_21_NO_4_ + H^+^], 459.1470; found, 459.1537. ^1^H NMR (700 MHz, CDCl_3_): δ 9.84 (s, 1H), 7.72 (d, *J* = 7.6 Hz, 2H), 7.63 (t, *J* = 7.6 Hz, 1H), 7.46 (t, *J* = 7.6 Hz, 2H), 7.40–7.35 (m, 2H), 7.33–7.27 (m, 2H), 7.16 (d, *J* = 1.9 Hz, 1H), 7.13–7.10 (m, 2H), 7.10–7.05 (m, 3H), 6.39 (d, *J* = 1.9 Hz, 1H), 5.21–5.19 (m, 1H), 4.10–4.05 (m, 1H), 3.32–3.29 (m, 2H). ^13^C NMR (176 MHz, CDCl_3_): δ 185.0, 163.6, 161.6, 156.4, 153.4, 142.4 (2C), 138.5, 138.4, 134.9, 129.5 (2C), 128.9 (2C), 128.8 (2C), 128.6, 128.1, 127.7, 126.1, 125.2, 122.4, 118.0, 117.4, 113.4, 109.1, 103.2, 50.4, 50.3, 37.7.

**3b** (1.9:1 dr in a crude reaction mixture) was isolated after 5 days in 52% yield (24.8 mg).

#### 4-((2*R*,3*R*)-2-(3-Fluorophenyl)-3-(3-formylfuran-2-yl)-3-phenylpropyl)-2-oxo-2*H*-chromene-3-carbonitrile Major-**3b**

Light-orange oil (16.2 mg). The er was determined by UPC^2^ using a chiral Chiralpak IB column gradient from 100% CO_2_ up to 40%; *i*-PrOH, 2.5 mL/min; *t*_R_ = 4.439 (major), *t*_R_ = 4.698 (minor): 99:1 er. [α]_D_^21^ = 81.5 (*c* = 1.0, CHCl_3_). HRMS *m*/*z*: [M + H]^+^ calculated for [C_30_H_20_FNO_4_ + H^+^], 477.1376; found, 477.1452. ^1^H NMR (700 MHz, CDCl_3_): δ 10.06 (s, 1H), 7.67–7.65 (m, 1H), 7.59–7.57 (m, 1H), 7.50 (d, *J* = 2.0 Hz, 1H), 7.38–7.36 (m, 1H), 7.34–7.33 (m, 1H), 7.25–7.23 (m, 2H), 7.15–7.12 (m, 2H), 7.10–7.08 (m, 1H), 7.08–7.06 (m, 1H), 6.87–6.86 (m, 1H), 6.76 (d, *J* = 2.0 Hz, 1H), 6.75–6.71 (m, 2H), 5.23–5.21 (m, 1H), 4.18–4.14 (m, 1H), 3.44–3.41 (m, 1H), 3.32–3.29 (m, 1H). ^13^C NMR (176 MHz, CDCl_3_): δ 185.2, 163.1, 162,7 (d, *J* = 247.7 Hz), 161.2, 156.4, 153.5, 142.9, 141.1 (d, *J* = 7.1 Hz), 138.0, 135.1, 130.5 (d, *J* = 8.3 Hz), 128.8 (2C), 128.5 (2C), 127.6, 125.8, 125.4, 124.0 (d, *J* = 2.8 Hz), 123.6, 118.1, 117.4, 115.4 (d, *J* = 21.7 Hz), 114.9 (d, *J* = 21.0 Hz), 113.4, 110.4, 103.0, 50.0, 49.5, 37.5.

#### 4-((2*S*,3*R*)-2-(3-Fluorophenyl)-3-(3-formylfuran-2-yl)-3-phenylpropyl)-2-oxo-2*H*-chromene-3-carbonitrile Minor-**3b**

Light-yellow oil (8.6 mg). The er was determined by UPC^2^ using a chiral Chiralpak IB column gradient from 100% CO_2_ up to 40%; *i*-PrOH, 2.5 mL/min; *t*_R_ = 4.774 (major), *t*_R_ = 4.467 (minor): 97:3 er. [α]_D_^21^ = −105.3 (*c* = 1.0, CHCl_3_). HRMS *m*/*z*: [M + H]^+^ calculated for [C_30_H_20_FNO_4_ + H^+^], 477.1376; found, 477.1451. ^1^H NMR (700 MHz, CDCl_3_): δ 9.84 (s, 1H), 7.71–7.69 (m, 2H), 7.66–7.63 (m, 1H), 7.47–7.45 (m, 2H), 7.40–7.37 (m, 1H), 7.32–7.31 (m, 1H), 7.31–7.30 (m, 2H), 7.19 (d, *J* = 2.0 Hz, 1H), 7.14–7.11 (m, 1H), 6.95–6.94 (m, 1H), 6.81–6.79 (m, 1H), 6.67–6.75 (m, 1H), 6.42 (d, *J* = 2.0 Hz, 1H), 5.21–5.20 (m, 1H), 4.09–4.05 (m, 1H), 3.32–3.30 (m, 1H), 3.29–3.28 (m, 1H). ^13^C NMR (176 MHz, CDCl_3_): δ 185.1, 163.1, 162.7 (d, *J* = 247.6 Hz), 161.0, 156.3, 153.5, 142.5, 141.2 (d, *J* = 6.7 Hz), 138.1, 135.1, 130.6 (d, *J* = 8.2 Hz), 129.6 (2C), 128.9, 128.8, 128.7, 125.9, 125.3, 123.4, 122.3, 118.1, 117.2, 115.0 (d, *J* = 21.8 Hz), 114.9 (d, *J* = 21.0 Hz), 113.3, 109.4, 103.2, 50.1, 50.0, 37.4.

**3c** (1.3:1 dr in a crude reaction mixture) was isolated after 7 days in 86% yield (42.4 mg).

#### 4-((2*R*,3*R*)-2-(2-Chlorophenyl)-3-(3-formylfuran-2-yl)-3-phenylpropyl)-2-oxo-2*H*-chromene-3-carbonitrile Major-**3c**

Yellow oil (23.6 mg). The er was determined by UPC^2^ using a chiral Chiralpak IB column gradient from 100% CO_2_ up to 40%; *i*-PrOH, 2.5 mL/min; *t*_R_ = 4.750 (major), *t*_R_ = 5.067 (minor): 99:1 er. [α]_D_^21^ = 58.1 (*c* = 1.0, CHCl_3_). HRMS *m*/*z*: [M + H]^+^ calculated for [C_30_H_20_ClNO_4_ + H^+^], 493.1081; found, 493.1151. ^1^H NMR (700 MHz, CDCl_3_): δ 10.11 (s, 1H), 7.65 (t, *J* = 7.6 Hz, 1H), 7.59 (d, *J* = 7.8 Hz, 1H), 7.56 (d, *J* = 7.8 Hz, 1H), 7.53–7.52 (m, 1H), 7.36–7.32 (m, 2H), 7.32–7.30 (m, 2H), 7.26–7.23 (m, 1H), 7.13 (t, *J* = 7.8 Hz, 2H), 7.08–7.06 (m, 1H), 7.01–7.00 (m, 2H), 6.81–6.80 (m, 1H), 5.29 (d, *J* = 11.1 Hz, 1H), 4.97–4.93 (m, 1H), 3.43–3.39 (m, 1H), 3.38–3.35 (m, 1H). ^13^C NMR (176 MHz, CDCl_3_): δ 185.1, 162.5, 161.4, 156.5, 153.4, 143.0 (2C), 137.8, 136.4, 134.9, 134.4, 129.5, 129.0, 128.9, 128.7 (2C), 128.4 (2C), 127.9, 127.6, 126.0, 125.3, 123.6, 117.9, 117.8, 110.4, 103.2, 49.3, 44.7, 37.5.

#### 4-((2*S*,3*R*)-2-(2-Chlorophenyl)-3-(3-formylfuran-2-yl)-3-phenylpropyl)-2-oxo-2*H*-chromene-3-carbonitrile Minor-**3c**

Light-orange oil (18.8 mg). The er was determined by UPC^2^ using a chiral Chiralpak IB column gradient from 100% CO_2_ up to 40%; *i*-PrOH, 2.5 mL/min; *t*_R_ = 5.187 (major), *t*_R_ = 4.764 (minor): 97:3 er. [α]_D_^21^ = −76.0 (*c* = 1.0, CHCl_3_). HRMS *m*/*z*: [M + H]^+^ calculated for [C_30_H_20_ClNO_4_ + H^+^], 493.1081; found, 493.1152. ^1^H NMR (700 MHz, CDCl_3_): δ 9.86 (s, 1H), 7.76–7.73 (m, 2H), 7.64–7.60 (m, 2H), 7.50–7.48 (m, 2H), 7.43–7.39 (m, 1H), 7.30–7.27 (m, 3H), 7.25–7.23 (m, 1H), 7.16 (d, *J* = 2.0 Hz, 1H), 7.10–7.04 (m, 2H), 6.40 (d, *J* = 2.0 Hz, 1H), 5.26–5.24 (m, 1H), 4.80–4.76 (m, 1H), 3.38–3.36 (m, 1H), 3.28–3.24 (m, 1H). ^13^C NMR (176 MHz, CDCl_3_): δ 185.1, 162.4, 161.0, 156.4, 153.4, 142.7, 138.3, 136.4, 134.9, 134.3, 129.6, 129.5 (2C), 129.1, 129.0 (2C), 128.8 (2C), 128.0, 126.0, 125.1, 122.2, 117.9, 117.8, 113.2, 109.3, 103.2, 49.8, 45.1, 37.7.

**3d** (1.6:1 dr in a crude reaction mixture) was isolated after 3 days in 90% yield (44.4 mg).

#### 4-((2*R*,3*R*)-2-(3-Chlorophenyl)-3-(3-formylfuran-2-yl)-3-phenylpropyl)-2-oxo-2*H*-chromene-3-carbonitrile Major-**3d**

Yellow oil (27.4 mg). The er was determined by UPC^2^ using a chiral Chiralpak IB column gradient from 100% CO_2_ up to 40%; *i*-PrOH, 2.5 mL/min; *t*_R_ = 4.158 (minor), *t*_R_ = 4.279 (major): >99:1 er. [α]_D_^21^ = 110.1 (*c* = 1.0, CHCl_3_). HRMS *m*/*z*: [M + H]^+^ calculated for [C_30_H_20_ClNO_4_ + H^+^], 493.1081; found, 493.1083. ^1^H NMR (700 MHz, CDCl_3_): δ 10.06 (s, 1H), 7.67–7.64 (m, 1H), 7.58–7.56 (m, 1H), 7.50 (d, *J* = 2.0 Hz, 1H), 7.38–7.36 (m, 1H), 7.34–7.32 (m, 1H), 7.25–7.23 (m, 2H), 7.15–7.13 (m, 2H), 7.09–7.06 (m, 1H), 7.03–7.02 (m, 1H), 7.02–7.01 (m, 2H), 6.95–6.94 (m, 1H), 6.76 (d, *J* = 2.0 Hz, 1H), 5.23–5.21 (m, 1H), 4.16–4.12 (m, 1H), 3.44–3.41 (m, 1H), 3.32–3.29 (m, 1H). ^13^C NMR (176 MHz, CDCl_3_): δ 185.2, 163.1, 161.2, 156.5, 153.5, 143.0, 140.6, 137.9, 135.2, 134.6, 130.1, 128.9 (2C), 128.5 (2C), 128.4, 128.1, 127.6, 126.5, 125.9, 125.4, 123.6, 118.1, 117.4, 113.4, 110.4, 102.9, 50.0, 49.5, 37.4.

#### 4-((2*S*,3*R*)-2-(3-Chlorophenyl)-3-(3-formylfuran-2-yl)-3-phenylpropyl)-2-oxo-2*H*-chromene-3-carbonitrile Minor-**3d**

Light-orange oil (17.1 mg). The er was determined by UPC^2^ using a chiral Chiralpak IA column gradient from 100% CO_2_ up to 40%; *i*-PrOH, 2.5 mL/min; *t*_R_ = 4.076 (minor), *t*_R_ = 4.270 (major): 99:1 er. [α]_D_^21^ = −135.0 (*c* = 1.0, CHCl_3_). HRMS *m*/*z*: [M + H]^+^ calculated for [C_30_H_20_ClNO_4_ + H^+^], 493.1081; found, 49.1085. ^1^H NMR (700 MHz, CDCl_3_): δ 9.84 (s, 1H), 7.73–7.70 (m, 2H), 7.64–7.62 (m, 1H), 7.47–7.46 (m, 2H), 7.40–7.36 (m, 1H), 7.34–7.32 (m, 1H), 7.30–7.28 (m, 1H), 7.1 (d, *J* = 2.0 Hz, 1H), 6.96–6.94 (m, 1H), 6.88–6.87 (m, 1H), 6.39 (d, *J* = 2.0 Hz, 1H), 5.15–5.14 (m, 1H), 4.04–4.01 (m, 1H), 3.33–3.24 (m, 2H), 2.20 (s, 3H). ^13^C NMR (176 MHz, CDCl_3_): δ 184.8, 163.6, 161.8, 156.5, 153.4, 142.3, 138.6, 138.4, 138.2, 134.8, 129.5 (2C), 128.8 (2C), 128.7, 126.6, 128.2, 126.1, 125.2, 124.9, 122.4, 118.0, 117.4, 113.5, 109.0, 103.2, 50.4, 50.3, 37.7, 21.4.

**3e** (1:1 dr in a crude reaction mixture) was isolated after 7 days in 63% yield (31 mg).

#### 4-((2*R*,3*R*)-2-(4-Chlorophenyl)-3-(3-formylfuran-2-yl)-3-phenylpropyl)-2-oxo-2*H*-chromene-3-carbonitrile Major-**3e**

Yellow oil (15.7 mg). The er was determined by UPC^2^ using a chiral Chiralpak IB column gradient from 100% CO_2_ up to 40%; *i*-PrOH, 2.5 mL/min; *t*_R_ = 4.602 (major), *t*_R_ = 5.044 (minor): 98:2 er. [α]_D_^21^ = 86.4 (*c* = 1.0, CHCl_3_). HRMS *m*/*z*: [M + H]^+^ calculated for [C_30_H_20_ClNO_4_ + H^+^], 493.1081; found, 493.1148. ^1^H NMR (700 MHz, CDCl_3_): δ 10.06 (s, 1H), 7.68–7.65 (m, 1H), 7.59–7.57 (m, 1H), 7.50 (d, *J* = 2.0 Hz, 1H), 7.38–7.36 (m, 1H), 7.34–7.33 (m, 1H), 7.24–7.21 (m, 2H), 7.14–7.12 (m, 2H), 7.09–7.07 (m, 2H), 7.07–7.06 (m, 1H), 6.98–6.97 (m, 2H), 6.76 (d, *J* = 2.0 Hz, 1H), 5.22–5.20 (m, 1H), 4.17–4.14 (m, 1H), 3.43–3.40 (m, 1H), 3.31–3.28 (m, 1H). ^13^C NMR (176 MHz, CDCl_3_): δ 185.2, 163.2, 161.3, 156.4, 153.5, 142.9, 138.0, 137.0, 135.1, 133.6, 129.6 (2C), 129.0 (2C), 128.9 (2C), 128.5 (2C), 127.6, 125.9, 125.4, 123.6, 118.1, 117.4, 113.4, 110.4, 103.0, 49.8, 49.6, 37.5.

#### 4-((2*S*,3*R*)-2-(4-Chlorophenyl)-3-(3-formylfuran-2-yl)-3-phenylpropyl)-2-oxo-2*H*-chromene-3-carbonitrile Minor-**3e**

Light-orange oil (15.3 mg). The er was determined by UPC^2^ using a chiral Chiralpak IA column gradient from 100% CO_2_ up to 40%; *i*-PrOH, 2.5 mL/min; *t*_R_ = 4.335 (major), *t*_R_ = 4.636 (minor): 95:5 er. [α]_D_^21^ = −127.2 (*c* = 1.0, CHCl_3_). HRMS *m*/*z*: [M + H]^+^ calculated for [C_30_H_20_ClNO_4_ + H^+^], 493.1081; found, 493.1150. ^1^H NMR (700 MHz, CDCl_3_): δ 9.83 (s, 1H), 7.71–7.69 (m, 2H), 7.66–7.63 (m, 1H), 7.47–7.45 (m, 2H), 7.40–7.36 (m, 1H), 7.33–7.31 (m, 2H), 7.31–7.29 (m, 1H), 7.17 (d, *J* = 2.0 Hz, 1H), 7.10–7.09 (m, 2H), 7.06–7.03 (m, 2H), 6.42 (d, *J* = 2.0 Hz, 1H), 5.21 (d, *J* = 11.7 Hz, 1H), 4.08–4.04 (m, 1H), 3.29–3.27 (m, 2H). ^13^C NMR (176 MHz, CDCl_3_): δ 185.2, 163.2, 160.9, 156.3, 153.5, 142.5 (2C), 138.2, 137.1, 135.1 (2C), 133.8, 129.6 (2C), 129.1 (2C), 128.8 (2C), 128.7, 125.9, 125.3, 122.3, 118.1, 117.2, 113.4, 109.6, 103.2, 50.2, 49.8, 37.5.

**3f** (1.4:1 dr in a crude reaction mixture) was isolated after 7 days in 51% yield (27.4 mg).

#### 4-((2*R*,3*R*)-2-(4-Bromophenyl)-3-(3-formylfuran-2-yl)-3-phenylpropyl)-2-oxo-2*H*-chromene-3-carbonitrile Major-**3f**

Yellow oil (16.2 mg). The er was determined by UPC^2^ using a chiral Chiralpak IB column gradient from 100% CO_2_ up to 40%; *i*-PrOH, 2.5 mL/min; *t*_R_ = 4.786 (major), *t*_R_ = 5.316 (minor): 99:1 er. [α]_D_^21^ = 60.8 (*c* = 1.0, CHCl_3_). HRMS *m*/*z*: [M + H]^+^ calculated for [C_30_H_20_BrNO_4_H^+^], 537.0576; found, 537.0645. ^1^H NMR (700 MHz, CDCl_3_): δ 10.06 (s, 1H), 7.68–7.66 (m, 1H), 7.59–7.58 (m, 1H), 7.50 (d, *J* = 1.9 Hz, 1H), 7.38–7.36 (m, 1H), 7.34–7.33 (m, 1H), 7.23–7.22 (m, 2H), 7.22–7.20 (m, 2H), 7.14–7.12 (m, 2H), 7.09–7.07 (m, 1H), 6.93–6.92 (m, 2H), 6.76 (d, *J* = 1.8 Hz, 1H), 5.22–5.20 (m, 1H), 4.17–4.13 (m, 1H), 3.43–3.40 (m, 1H), 3.31–3.29 (m, 1H). ^13^C NMR (176 MHz, CDCl_3_): δ 185.3, 163.2, 161.3, 156.4, 153.5, 142.9, 138.0, 137.5, 135.2, 132.0 (2C), 129.9 (2C), 128.9 (2C), 128.5 (2C), 127.6, 125.9, 125.4, 123.6, 121.7, 118.1, 117.4, 113.4, 110.4, 103.0, 49.8, 49.6, 37.5.

#### 4-((2*S*,3*R*)-2-(4-Bromophenyl)-3-(3-formylfuran-2-yl)-3-phenylpropyl)-2-oxo-2*H*-chromene-3-carbonitrile Minor-**3f**

Light-orange oil (11.2 mg). The er was determined by UPC^2^ using a chiral Chiralpak IB column gradient from 100% CO_2_ up to 40%; *i*-PrOH, 2.5 mL/min; *t*_R_ = 4.774 (major), *t*_R_ = 4.467 (minor): 97:3 er. [α]_D_^21^ = −77.8 (*c* = 1.0, CHCl_3_). HRMS *m*/*z*: [M + H]^+^ calculated for [C_30_H_20_BrNO_4_H^+^], 537.0576; found, 537.0648. ^1^H NMR (700 MHz, CDCl_3_): δ 9.83 (s, 1H), 7.70–7.69 (m, 2H), 7.66–7.64 (m, 1H), 7.47–7.45 (m, 2H), 7.39–7.37 (m, 1H), 7.32–7.31 (m, 1H), 7.31–7.30 (m, 2H), 7.25–7.24 (m, 2H), 7.18–7.17 (m, 1H), 7.00–6.98 (m, 2H), 6.43–6.41 (m, 1H), 5.22–5.21 (m, 1H), 4.09–4.01 (m, 1H), 3.29–3.28 (m, 1H), 3.27–3.26 (m, 1H). ^13^C NMR (176 MHz, CDCl_3_): δ 185.3, 163.2, 160.9, 156.3, 153.5, 142.5, 138.1, 137.6, 135.1, 132.0 (2C), 129.6 (2C), 129.4 (2C), 128.8 (2C), 128.7, 125.9, 125.3, 122.3, 121.9, 118.1, 117.2, 113.4, 109.6, 103.2, 50.1, 49.8, 37.4.

**3g** (1.2:1 dr in a crude reaction mixture) was isolated after 7 days in 37% yield (19.5 mg).

#### 4-((2*R*,3*R*)-3-(3-Formylfuran-2-yl)-3-phenyl-2-(4-(trifluoromethyl)­phenyl)­propyl)-2-oxo-2*H*-chromene-3-carbonitrile Major-**3g**

Yellow oil (10.6 mg). The er was determined by UPC^2^ using a chiral Chiralpak IB column gradient from 100% CO_2_ up to 40%; *i*-PrOH, 2.5 mL/min; *t*_R_ = 3.900 (major), *t*_R_ = 4.359 (minor): 99:1 er. [α]_D_^21^ = 76.9 (*c* = 1.0, CHCl_3_). HRMS *m*/*z*: [M + H]^+^ calculated for [C_31_H_20_F_3_NO_4_H^+^], 527.1344; found, 527.1413. ^1^H NMR (700 MHz, CDCl_3_): δ 10.05 (s, 1H), 7.67–7.64 (m, 1H), 7.54–7.53 (m, 1H), 7.49 (d, *J* = 1.9 Hz, 1H), 7.36–7.35 (m, 2H), 7.34–7.32 (m, 2H), 7.24–7.21 (m, 2H), 7.19–7.18 (m, 2H), 7.13–7.11 (m, 2H), 7.08–7.06 (m, 1H), 6.75 (d, *J* = 1.9 Hz, 1H), 5.29–5.58 (m, 1H), 4.27–4.27 (m, 1H), 3.46–3.43 (m, 1H), 3.36–3.33 (m, 1H). ^13^C NMR (176 MHz, CDCl_3_): δ 185.4, 163.0, 160.8, 156.4, 153.5, 143.0, 142.8, 137.8, 135.2, 130.1, 129.9 (q, *J* = 32.8 Hz), 128.9 (2C), 128.7 (2C), 128.5 (2C), 127.7, 125.9, 125.8 (q, *J* = 3.9 Hz, 2C), 125.4, 123.8 (q, *J* = 272.1 Hz), 118.1, 117.4, 113.4, 110.5, 103.0, 50.1, 49.4, 37.3.

#### 4-((2*S*,3*R*)-3-(3-Formylfuran-2-yl)-3-phenyl-2-(4-(trifluoromethyl)­phenyl)­propyl)-2-oxo-2*H*-chromene-3-carbonitrile Minor-**3g**

Light-orange oil (8.9 mg). The er was determined by UPC^2^ using a chiral Chiralpak IA column gradient from 100% CO_2_ up to 40%; *i*-PrOH, 2.5 mL/min; *t*_R_ = 4.067 (major), *t*_R_ = 3.942 (minor): 99:1 er. [α]_D_^21^ = −99.3 (*c* = 1.0, CHCl_3_). HRMS *m*/*z*: [M + H]^+^ calculated for [C_31_H_20_F_3_NO_4_H^+^], 527.1344; found, 527.1419. ^1^H NMR (700 MHz, CDCl_3_): δ 9.82 (s, 1H), 7.72–7.69 (m, 2H), 7.65–7.63 (m, 1H), 7.47–7.45 (m, 2H), 7.40–7.38 (m, 2H), 7.38–7.37 (m, 1H), 7.31–7.30 (m, 1H), 7.30–7.29 (m, 2H), 7.25–7.24 (m, 2H), 7.17 (d, *J* = 2.0 Hz, 1H), 6.41 (d, *J* = 2.0 Hz, 1H), 5.31–5.30 (m, 1H), 4.18–4.14 (m, 1H), 3.33–3.32 (m, 1H), 3.32–3.31 (m, 1H). ^13^C NMR (176 MHz, CDCl_3_): δ 185.4, 163.0, 160.5, 156.2, 153.5, 142.8, 142.5, 138.0, 135.1, 130.3 (q, *J* = 32.7 Hz), 129.6 (2C), 128.9 (2C), 128.8, 128.3 (2C), 125.9, 125.8 (q, *J* = 3.7 Hz, 2C), 125.3, 123.8 (d, *J* = 272.1 Hz), 122.2, 118.1, 117.2, 113.3, 109.8, 103.2, 50.1, 49.9, 37.3.

**3h** (2.3:1 dr in a crude reaction mixture) was isolated after 3 days in 72% yield (34.1 mg).

#### 4-((2*R*,3*R*)-3-(3-Formylfuran-2-yl)-3-phenyl-2-(*m*-tolyl)­propyl)-2-oxo-2*H*-chromene-3-carbonitrile Major-**3h**

Yellow oil (13.8 mg). The er was determined by UPC^2^ using a chiral Chiralpak IB column gradient from 100% CO_2_ up to 40%; *i*-PrOH, 2.5 mL/min; *t*_R_ = 4.301 (major), *t*_R_ = 4.501 (minor): 99:1 er. [α]_D_^21^ = 31.5 (*c* = 1.0, CHCl_3_). HRMS *m*/*z*: [M + H]^+^ calculated for [C_31_H_23_NO_4_ + H^+^], 474.1627; found, 474.1700. ^1^H NMR (700 MHz, CDCl_3_): δ 10.08 (s, 1H), 7.66–7.63 (m, 1H), 7.61 (d, *J* = 8.2 Hz, 1H), 7.51 (d, *J* = 2.0 Hz, 1H), 7.36 (t, *J* = 7.7 Hz, 1H), 7.32 (d, *J* = 8.2 Hz, 1H), 7.24 (d, *J* = 7.7 Hz, 2H), 7.12 (t, *J* = 7.6 Hz, 2H), 7.06 (t, *J* = 7.5 Hz, 1H), 6.92 (t, *J* = 7.5 Hz, 1H), 6.89 (s, 1H), 6.84 (d, *J* = 7.5 Hz, 1H), 6.77 (d, *J* = 2.0 Hz, 1H), 6.70 (d, *J* = 7.6 Hz, 1H), 5.21 (d, *J* = 11.0 Hz, 1H), 4.10 (td, *J* = 10.3, 5.2 Hz, 1H), 3.43 (dd, *J* = 13.3, 10.3 Hz, 1H), 3.29 (dd, *J* = 13.3, 5.2 Hz, 1H), 2.18 (s, 3H). ^13^C NMR (176 MHz, CDCl_3_): δ 185.0, 163.6, 162.1, 156.6, 153.4, 142.8, 138.5, 138.4, 138.1, 134.9, 128.8, 128.7 (2C), 128.6, 128.5, 128.4 (2C), 127.4, 126.0, 125.5, 125.3, 123.6, 118.0, 117.6, 113.5, 110.1, 103.0, 50.4, 49.6, 37.7, 21.4. ***4-((2S,3R)-3-(3-Formylfuran-2-yl)-3-phenyl-2-(m-tolyl)­propyl)-2-oxo-2H-chromene-3-carbonitrile minor-3h.*** Light-orange oil (10.3 mg). The er was determined by UPC^2^ using a chiral Chiralpak IA column gradient from 100% CO_2_ up to 40%; acetonitrile, 2.5 mL/min; *t*_R_ = 3.665 (major), *t*_R_ = 4.099 (minor): 97:3 er. [α]_D_^21^ = −44.5 (*c* = 1.0, CHCl_3_). HRMS *m*/*z*: [M + H]^+^ calculated for [C_31_H_23_NO_4_ + H^+^], 474.1627; found, 474.1700. ^1^H NMR (700 MHz, CDCl_3_): δ 9.84 (s, 1H), 7.72–7.70 (m, 2H), 7.64–7.62 (m, 1H), 7.47–7.45 (m, 2H), 7.40–7.37 (m, 2H), 7.34–7.32 (m, 1H), 7.30–7.28 (m, 1H), 7.17 (d, *J* = 2.0 Hz, 1H), 6.96–6.94 (m, 2H), 6.88–6.87 (m, 1H), 6.75 (s, 1H), 6.39 (d, *J* = 2.0 Hz, 1H), 5.15–5.14 (m, 1H), 4.04–4.01 (m, 1H), 3.32–3.29 (m, 1H), 3.27–3.25 (m, 1H), 2.20 (s, 3H). ^13^C NMR (176 MHz, CDCl_3_): δ 184.8, 163.6, 161.8, 156.5, 153.4, 142.3, 138.6, 138.4, 138.2, 134.8, 129.5 (2C), 128.9 (2C), 128.6, 128.5, 128.1, 126.1, 125.2, 124.9, 122.4, 118.0 (2C), 117.4, 113.5, 109.0 (2C), 103.2, 50.4, 37.7, 21.4.

**3i** (1.6:1 dr in a crude reaction mixture) was isolated after 7 days in 69% yield (32.7 mg).

#### 4-((2*R*,3*R*)-3-(3-Formylfuran-2-yl)-3-phenyl-2-(*p*-tolyl)­propyl)-2-oxo-2*H*-chromene-3-carbonitrile Major-**3i**

Yellow oil (20.1 mg). The er was determined by UPC^2^ using a chiral Chiralpak IB column gradient from 100% CO_2_ up to 40%; *i*-PrOH, 2.5 mL/min; *t*_R_ = 4.341 (major), *t*_R_ = 4.650 (minor): 99:1 er. [α]_D_^21^ = 81.2 (*c* = 1.0, CHCl_3_). HRMS *m*/*z*: [M + H]^+^ calculated for [C_31_H_23_NO_4_ + H^+^], 474.1627; found, 474.1696. ^1^H NMR (700 MHz, CDCl_3_): δ 10.08 (s, 1H), 7.66–7.64 (m, 1H), 7.60–7.59 (m, 1H), 7.50 (d, *J* = 2.0 Hz, 1H), 7.37–7.35 (m, 1H), 7.32–7.31 (m, 1H), 7.25–7.23 (m, 2H), 7.13–7.11 (m, 2H), 7.08–7.05 (m, 1H), 6.89–6.88 (m, 4H), 6.77 (d, *J* = 2.0 Hz, 1H), 5.21–5.19 (m, 1H), 4.14–4.10 (m, 1H), 3.44–3.40 (m, 1H), 3.30–3.28 (m, 1H), 2.15 (s, 3H). ^13^C NMR (176 MHz, CDCl_3_): δ 185.0, 163.6, 162.2, 156.6, 153.4, 142.8 (2C), 138.5, 137.4, 135.1, 134.9, 129.5 (2C), 128.7 (2C), 128.5 (2C), 128.0, 127.3, 126.1, 125.3, 123.6, 118.0, 117.6, 113.5, 110.1, 103.0, 50.1, 49.7, 37.9, 21.1.

#### 4-((2*S*,3*R*)-3-(3-Formylfuran-2-yl)-3-phenyl-2-(*p*-tolyl)­propyl)-2-oxo-2*H*-chromene-3-carbonitrile Minor-**3i**

Light-orange oil (12.6 mg). The er was determined by UPC^2^ using a chiral Chiralpak IA column gradient from 100% CO_2_ up to 40%; *i*-PrOH, 2.5 mL/min; *t*_R_ = 4.125 (major), *t*_R_ = 4.593 (minor): 97:3 er. [α]_D_^21^ = −96.3 (*c* = 1.0, CHCl_3_). HRMS *m*/*z*: [M + H]^+^ calculated for [C_31_H_23_NO_4_ + H^+^], 474.1627; found, 474.1699. ^1^H NMR (700 MHz, CDCl_3_): δ 9.85 (s, 1H), 7.72–7.69 (m, 2H), 7.64–7.62 (m, 1H), 7.47–7.45 (m, 2H), 7.39–7.37 (m, 2H), 7.34–7.31 (m, 1H), 7.30–7.28 (m, 1H), 7.17 (d, *J* = 2.0 Hz, 1H), 6.93–6.91 (m, 2H), 6.91–6.90 (m, 2H), 6.39 (d, *J* = 2.0 Hz, 1H), 5.15–5.13 (m, 1H), 4.06–4.02 (m, 1H), 3.31–3.27 (m, 1H), 3.26–7.24 (m, 1H), 2.17 (s, 3H). ^13^C NMR (176 MHz, CDCl_3_): δ 184.9, 163.7, 162.0, 156.5, 153.4, 142.3 (2C), 138.5, 137.7, 135.2, 134.8, 129.6 (2C), 129.5 (2C), 128.8 (2C), 128.6, 127.5, 126.1, 125.2, 122.4, 118.0, 117.4, 113.5, 109.0, 103.2, 50.4, 50.0, 37.8, 21.1.

**3j** (2:1 dr in a crude reaction mixture) was isolated after 5 days in 37% yield (18.1 mg).

#### 4-((2*R*,3*R*)-3-(3-Formylfuran-2-yl)-2-(4-methoxyphenyl)-3-phenylpropyl)-2-oxo-2*H*-chromene-3-carbonitrile Major-**3j**

Yellow oil (12.1 mg). The er was determined by UPC^2^ using a chiral Chiralpak IB column gradient from 100% CO_2_ up to 40%; *i*-PrOH, 2.5 mL/min; *t*_R_ = 4.563 (major), *t*_R_ = 4.992 (minor): 90:10 er. [α]_D_^21^ = 9.1 (*c* = 1.0, CHCl_3_). HRMS *m*/*z*: [M + H]^+^ calculated for [C_31_H_23_NO_5_ + H^+^], 490.1576; found, 490.1643. ^1^H NMR (700 MHz, CDCl_3_): δ 10.08 (s, 1H), 7.66–7.64 (m, 1H), 7.61–7.60 (m, 1H), 7.51 (d, *J* = 2.0 Hz, 1H), 7.38–7.35 (m, 1H), 7.33–7.31 (m, 1H), 7.25–7.22 (m, 2H), 7.14–7.11 (m, 2H), 7.08–7.05 (m, 1H), 6.92–6.91 (m, 2H), 6.77 (d, *J* = 2.0 Hz, 1H), 6.62–6.59 (m, 2H), 5.18–5.17 (m, 1H), 4.12–4.08 (m, 1H), 3.66 (s, 3H), 3.43–3.39 (m, 1H), 3.29–3.27 (m, 1H). ^13^C NMR (176 MHz, CDCl_3_): δ 185.1, 163.7, 162.2, 158.9, 156.6, 153.4, 142.9, 138.5, 134.9, 130.7, 130.2, 129.3, 128.7 (2C), 128.5 (2C), 127.4, 126.1, 125.3, 123.6, 118.0, 117.6, 114.2 (2C), 113.5, 110.1, 103.0, 55.2, 49.9, 49.8, 37.9. ***4-((2S,3R)-3-(3-Formylfuran-2-yl)-2-(4-methoxyphenyl)-3-phenylpropyl)-2-oxo-2H-chromene-3-carbonitrile minor-3j.*** Light-orange oil (6.0 mg). The er was determined by UPC^2^ using a chiral Chiralpak IB column gradient from 100% CO_2_ up to 40%; *i*-PrOH, 2.5 mL/min; *t*_R_ = 4.519 (major), *t*_R_ = 4.665 (minor): 97:3 er. [α]_D_^21^ = −16.0 (*c* = 1.0, CHCl_3_). HRMS *m*/*z*: [M + H]^+^ calculated for [C_31_H_23_NO_5_ + H^+^], 490.1576; found, 490.1647. ^1^H NMR (700 MHz, CDCl_3_): δ 9.84 (s, 1H), 7.71–7.69 (m, 2H), 7.64–7.62 (m, 2H), 7.47–7.45 (m, 3H), 7.38–7.36 (m, 3H), 7.33–7.31 (m, 1H), 7.29–7.28 (m, 1H), 7.17 (d, *J* = 1.9 Hz, 1H), 6.98–6.96 (m, 1H), 6.63–6.62 (m, 2H), 6.40 (d, *J* = 1.9 Hz, 1H), 3.67 (s, 3H), 3.27–3.26 (m, 1H), 3.26–3.25 (m, 1H). ^13^C NMR (176 MHz, CDCl_3_): δ 185.1, 163.7, 162.2, 158.9, 156.6, 153.4, 142.9, 138.5, 134.9, 130.7, 130.2, 129.3, 128.7 (2C), 128.5 (2C), 127.4, 126.1, 125.3, 123.6, 118.0, 117.6, 114.2 (2C), 113.5, 110.1, 103.0, 55.2, 49.9, 49.8, 37.9.

**3k** (1.4:1 dr in a crude reaction mixture) was isolated after 7 days in 42% yield (21.4 mg).

#### 4-((2*R*,3*R*)-3-(3-Formylfuran-2-yl)-2-(naphthalen-2-yl)-3-phenylpropyl)-2-oxo-2*H*-chromene-3-carbonitrile Major-**3k**

Yellow oil (12.5 mg). The er was determined by UPC^2^ using a chiral Chiralpak IB column gradient from 100% CO_2_ up to 40%; *i*-PrOH, 2.5 mL/min; *t*_R_ = 4.996 (major), *t*_R_ = 5.545 (minor): 98:2 er. [α]_D_^21^ = 49.6 (*c* = 1.0, CHCl_3_). HRMS *m*/*z*: [M + H]^+^ calculated for [C_34_H_23_NO_4_ + H^+^], 510.1627; found, 510.1700. ^1^H NMR (700 MHz, CDCl_3_): δ 10.10 (s, 1H), 7.67–7.64 (m, 2H), 7.64–7.62 (m, 1H), 7.62–7.60 (m, 1H), 7.60–7.58 (m, 1H), 7.52 (d, *J* = 1.9 Hz, 1H), 7.41–7.40 (m, 1H), 7.38–7.37 (m, 1H), 7.37–7.36 (m, 1H), 7.33–7.30 (m, 1H), 7.28–7.27 (m, 1H), 7.27–7.26 (m, 2H), 7.26–7.25 (m, 1H), 7.07–7.05 (m, 2H), 7.00–6.97 (m, 1H), 6.77 (d, *J* = 1.9 Hz, 1H), 5.36–5.35 (m, 1H), 4.38–4.34 (m, 1H), 3.57–3.54 (m, 1H), 3.41–3.38 (m, 1H). ^13^C NMR (176 MHz, CDCl_3_): δ 185.2, 163.5, 161.8, 156.5, 153.4, 142.9, 138.3, 135.9, 134.9, 133.1, 132.7, 128.8, 128.7 (2C), 128.5 (2C), 127.8, 127.7, 127.6, 127.4, 126.4, 126.1, 126.0, 125.5, 125.3, 123.6, 118.0, 117.6, 113.5, 110.3, 102.9, 50.5, 49.7, 38.7.

#### 4-((2*S*,3*R*)-3-(3-Formylfuran-2-yl)-2-(naphthalen-2-yl)-3-phenylpropyl)-2-oxo-2*H*-chromene-3-carbonitrile Minor-**3k**

Light-orange oil (8.9 mg). The er was determined by UPC^2^ using a chiral Chiralpak IA column gradient from 100% CO_2_ up to 40%; *i*-PrOH, 2.5 mL/min; *t*_R_ = 4.705 (major), *t*_R_ = 5.088 (minor): 97:3 er. [α]_D_^21^ = −81.0 (*c* = 1.0, CHCl_3_). HRMS *m*/*z*: [M + H]^+^ calculated for [C_34_H_23_NO_4_ + H^+^], 510.1627; found, 510.1694. ^1^H NMR (700 MHz, CDCl_3_): δ 9.82 (s, 1H), 7.77–7.75 (m, 2H), 7.69–7.67 (m, 1H), 7.66–7.64 (m, 1H), 7.64–7.62 (m, 1H), 7.6–7.59 (m, 1H), 7.49–7.46 (m, 2H), 7.44–7.42 (m, 1H), 7.39–7.38 (m, 2H), 7.38–7.37 (m, 2H), 7.32–7.29 (m, 2H), 7.25–7.24 (m, 1H), 7.13 (d, *J* = 1.9 Hz, 1H), 6.28 (d, *J* = 1.9 Hz, 1H), 5.35–5.33 (m, 1H), 4.31–4.27 (m, 1H), 3.45–3.41 (m, 1H), 3.38–3.35 (m, 1H). ^13^C NMR (176 MHz, CDCl_3_): δ 185.0, 163.6, 161.5, 156.4, 153.4, 142.4, 138.4, 135.9, 134.9, 133.2, 132.9, 129.5 (C), 128.9 (2C), 128.7 (2C), 127.8, 127.7, 127.1, 126.5, 126.2, 126.0, 125.2, 125.0, 122.3, 118.0, 117.4, 113.4, 109.3, 103.2, 50.4, 50.5, 37.6.

**3l** (1:1 dr in a crude reaction mixture) was isolated after 7 days in 56% yield (30.1 mg).

#### 6-Bromo-4-((2*R*,3*R*)-3-(3-formylfuran-2-yl)-2,3-diphenylpropyl)-2-oxo-2*H*-chromene-3-carbonitrile Major-**3l**

Yellow oil (15.2 mg) The er was determined by UPC^2^ using a chiral Chiralpak IB column gradient from 100% CO_2_ up to 40%; *i*-PrOH, 2.5 mL/min; *t*_R_ = 5.099 (major), *t*_R_ = 5.400 (minor): 99:1 er. [α]_D_^21^ = 12.2 (*c* = 1.0, CHCl_3_). HRMS *m*/*z*: [M + H]^+^ calculated for [C_30_H_20_BrNO_4_ + H^+^], 538.0576; found, 538.0647. ^1^H NMR (700 MHz, CDCl_3_): δ 10.12 (s, 1H), 7.74–7.73 (m, 1H), 7.72–7.71 (m, 1H), 7.61 (d, *J* = 2.0 Hz, 1H), 7.26–7.24 (m, 2H), 7.21–7.20 (m, 1H), 7.13–7.12 (m, 2H), 7.11–7.10 (m, 2H), 7.07–7.04 (m, 2H), 7.02–7.01 (m, 2H), 6.80 (d, *J* = 2.0 Hz, 1H), 5.29–5.27­(m, 1H), 4.07–4.04 (m, 1H), 3.41–3.38 (m, 1H), 3.23–3.20 (m, 1H). ^13^C NMR (176 MHz, CDCl_3_): δ 185.2, 162.3, 161.8, 155.8, 152.2, 143.2, 138.1, 137.8, 137.6, 128.9 (2C), 128.8 (2C), 128.7, 128.5 (2C), 128.4 (2C), 128.1, 127.5, 123.7, 119.6, 119.0, 118.3, 112.8, 110.2, 103.9, 50.6, 49.4, 37.9.

#### 6-Bromo-4-((2*S*,3*R*)-3-(3-formylfuran-2-yl)-2,3-diphenylpropyl)-2-oxo-2*H*-chromene-3-carbonitrile Minor-**3l**

Light-orange oil (14.9 mg). The er was determined by UPC^2^ using a chiral Chiralpak IB column gradient from 100% CO_2_ up to 40%; *i*-PrOH, 2.5 mL/min; *t*_R_ = 5.379 (major), *t*_R_ = 5.176 (minor): 98:2 er. [α]_D_^21^ = −26.9 (*c* = 1.0, CHCl_3_). HRMS *m*/*z*: [M + H]^+^ calculated for [C_30_H_20_BrNO_4_ + H^+^], 538.0576; found, 538.0648. ^1^H NMR (700 MHz, CDCl_3_): δ 9.85 (s, 1H), 8.10–8.09 (m, 1H), 7.74–7.43 (m, 2H), 7.71–7.69 (m, 1H), 7.53–7.71 (m, 2H), 7.51–7.48 (m, 2H), 7.41–7.39 (m, 1H), 7.19–7.17 (m, 1H), 7.15–7.13 (m, 1H), 7.11–7.10 (m, 1H), 6.40–6.39 (m, 1H), 5.41–5.32 (m, 1H), 5.24–5.22 (m, 1H), 4.70–4.69 (m, 1H), 4.01–3.97 (m, 1H), 3.28–3.24 (m, 1H), 3.22–3.19 (m, 1H). ^13^C NMR (176 MHz, CDCl_3_): δ 185.1, 165.4, 162.4, 161.3, 155.7, 152.2, 142.5, 138.3, 138.0, 137.5, 133.9, 130.0, 129.9 (2C), 129.0, 128.7 (2C), 128.6, 128.3, 127.8, 122.4, 119.6, 118.8, 118.3, 112.9, 109.2, 104.1, 50.6, 50.0, 37.6.

**3m** (2:1 dr in a crude reaction mixture) was isolated after 5 days in 41% yield (20.1 mg).

#### 4-((2*R*,3*R*)-3-(3-Formylfuran-2-yl)-2,3-diphenylpropyl)-7-methoxy-2-oxo-2*H*-chromene-3-carbonitrile Major-**3m**

Yellow oil (13.4 mg). The er was determined by UPC^2^ using a chiral Chiralpak IB column gradient from 100% CO_2_ up to 40%; *i*-PrOH, 2.5 mL/min; *t*_R_ = 5.001 (major), *t*_R_ = 5.308 (minor): 99:1 er. [α]_D_^21^ = 12.7 (*c* = 1.0, CHCl_3_). HRMS *m*/*z*: [M + H]^+^ calculated for [C_31_H_23_NO_5_ + H^+^], 490.1576; found, 490.1648. ^1^H NMR (700 MHz, CDCl_3_): δ 10.09 (s, 1H), 7.52–7.50 (m, 1H), 7.47–7.46 (m, 1H), 7.25–7.24 (m, 2H), 7.12–7.10 (m, 2H), 7.08–7.06 (m, 2H), 7.05–7.04 (m, 2H), 7.02–7.01 (m, 2H), 6.89–6.87 (m, 1H), 6.76–6.74 (m, 2H), 5.23–5.21 (m, 1H), 4.15–4.11 (m, 1H), 3.90 (s, 3H), 3.39–3.36 (m, 1H), 3.25–3.22 (m, 1H). ^13^C NMR (176 MHz, CDCl_3_): δ 185.1, 165.2, 163.4, 162.0, 157.3, 155.8, 142.9, 138.4, 138.3, 128.8 (2C), 128.7 (2C), 128.6 (2C), 128.3 (2C), 127.8, 127.3 (2C), 123.6, 114.0, 113.9, 111.3, 110.1, 101.3, 99.1, 56.3, 50.5, 49.6, 37.7.

#### 4-((2*S*,3*R*)-3-(3-Formylfuran-2-yl)-2,3-diphenylpropyl)-7-methoxy-2-oxo-2*H*-chromene-3-carbonitrile Minor-**3m**

Light-orange oil (6.7 mg). The er was determined by UPC^2^ using a chiral Chiralpak IA column gradient from 100% CO_2_ up to 40%; *i*-PrOH, 2.5 mL/min; *t*_R_ = 4.312 (major), *t*_R_ = 4.762 (minor): 99:1 er. [α]_D_^21^ = −25.5 (*c* = 1.0, CHCl_3_). HRMS *m*/*z*: [M + H]^+^ calculated for [C_31_H_23_NO_5_ + H^+^], 490.1576; found, 490.1647. ^1^H NMR (700 MHz, CDCl_3_): δ 9.84 (s, 1H), 7.72–7.70 (m, 2H), 7.48–7.44 (m, 2H), 7.39–7.37 (m, 1H), 7.25–7.22 (m, 1H), 7.16 (d, *J* = 1.9 Hz, 1H), 7.13–7.12 (m, 2H), 7.11–7.09 (m, 1H), 7.09–7.05 (m, 2H), 6.87–6.83 (m, 1H), 6.72–6.71 (m, 1H), 6.38 (d, *J* = 1.9 Hz, 1H), 5.18–5.15 (m, 1H), 4.11–4.01 (m, 1H), 3.90 (s, 3H), 3.24–3.23 (s, 1H), 3.22–3.19 (m, 1H). ^13^C NMR (176 MHz, CDCl_3_): δ 185.2, 165.2, 163.4, 162.0, 157.3, 155.8, 142.9, 138.4, 138.3, 128.7 (2C), 128.7 (2C), 128.6 (2C), 128.3 (2C), 127.8, 127.3 (2C), 123.6, 114.0, 113.9, 111.3, 110.1, 101.3, 99.0, 56.3, 50.4, 49.6, 37.7.

**3n** (1.8:1 dr in a crude reaction mixture) was isolated after 3 days in 63% yield (33.2 mg).

#### 4-((2*R*,3*R*)-3-(3-Formylfuran-2-yl)-2-phenyl-3-(3-(trifluoromethyl)­phenyl)­propyl)-2-oxo-2*H*-chromene-3-carbonitrile Major-**3n**

Yellow oil (21.3 mg). The er was determined by UPC^2^ using a chiral Chiralpak IB column gradient from 100% CO_2_ up to 40%; *i*-PrOH, 2.5 mL/min; *t*_R_ = 3.936 (major), *t*_R_ = 4.172 (minor): 99:1 er. [α]_D_^21^ = −51.5 (*c* = 1.0, CHCl_3_). HRMS *m*/*z*: [M + H]^+^ calculated for [C_31_H_20_F_3_NO_4_ + H^+^], 528.1344; found, 528.1409. ^1^H NMR (700 MHz, CDCl_3_): δ 10.07 (s, 1H), 7.67–7.63 (m, 1H), 7.60–7.57 (m, 1H), 7.52 (d, *J* = 2.0 Hz, 1H), 7.45 (s, 1H), 7.42 (d, *J* = 7.9 Hz, 1H), 7.37 (t, *J* = 7.6 Hz, 1H), 7.32 (d, *J* = 8.3 Hz, 1H), 7.30 (d, *J* = 7.8 Hz, 1H), 7.22 (t, *J* = 7.8 Hz, 1H), 7.09 (t, *J* = 7.4 Hz, 2H), 7.04 (d, *J* = 7.2 Hz, 1H), 7.02 (d, *J* = 8.1 Hz, 2H), 6.79 (d, *J* = 2.0 Hz, 1H), 5.37 (d, *J* = 11.4 Hz, 1H), 4.18–4.09 (m, 1H), 3.46 (dd, *J* = 13.4, 9.8 Hz, 1H), 3.31 (dd, *J* = 13.4, 5.4 Hz, 1H). ^13^C NMR (176 MHz, CDCl_3_): δ 185.5, 163.2, 160.2, 156.5, 153.4, 143.2, 139.4, 137.9, 135.1, 132.1, 130.8 (q, *J* = 32.4 Hz), 129.1, 129.0 (2C), 128.2 (2C), 128.1, 126.0, 125.4, 125.3 (q, *J* = 3.9 Hz), 124.2 (q, *J* = 3.9 Hz), 123.8 (q, *J* = 273.5 Hz), 123.7, 118.0, 117.5, 113.4, 110.7, 102.9, 50.3, 49.4, 37.6.

#### 4-((2*S*,3*R*)-3-(3-Formylfuran-2-yl)-2-phenyl-3-(3-(trifluoromethyl)­phenyl)­propyl)-2-oxo-2*H*-chromene-3-carbonitrile Minor-**3n**

Light-orange oil (11.9 mg). The er was determined by UPC^2^ using a chiral Chiralpak IB column gradient from 100% CO_2_ up to 40%; *i*-PrOH, 2.5 mL/min; *t*_R_ = 4.417 (major), *t*_R_ = 3.947 (minor): 99:1 er. [α]_D_^21^ = −75.9 (*c* = 1.0, CHCl_3_). HRMS *m*/*z*: [M + H]^+^ calculated for [C_31_H_20_F_3_NO_4_ + H^+^], 528.1344; found, 528.1407. ^1^H NMR (700 MHz, CDCl_3_): δ 9.81 (s, 1H), 8.00 (s, 1H), 7.95 (d, *J* = 7.5 Hz, 1H), 7.67–7.61 (m, 3H), 7.32 (d, *J* = 4.4 Hz, 2H), 7.30 (d, *J* = 8.3 Hz, 1H), 7.19 (d, *J* = 1.9 Hz, 1H), 7.12 (d, *J* = 7.2 Hz, 2H), 7.09 (dd, *J* = 15.1, 8.1 Hz, 3H), 6.40 (d, *J* = 2.0 Hz, 1H), 5.37 (d, *J* = 11.7 Hz, 1H), 4.08 (td, *J* = 11.3, 4.4 Hz, 1H), 3.33 (dd, *J* = 13.3, 11.0 Hz, 1H), 3.20 (dd, *J* = 13.3, 4.5 Hz, 1H). ^13^C NMR (176 MHz, CDCl_3_): δ 185.3, 163.1, 159.9, 156.3, 153.4, 142.7, 139.5, 137.9, 135.1, 132.9, 131.9 (q, *J* = 32.6 Hz), 130.2, 129.0 (2C), 128.3, 127.7 (2C), 125.7, 125.4 (q, *J* = 3.4 Hz), 125.3, 125.0 (q, *J* = 3.6 Hz), 124.0 (q, *J* = 272.7 Hz), 122.4, 118.1, 117.2, 113.3, 109.6, 103.3, 50.3, 49.8, 37.5.

**3o** (2.3:1 dr in a crude reaction mixture) was isolated after 3 days in 72% yield (35.2 mg).

#### 4-((2*R*,3*R*)-3-(3-Formylfuran-2-yl)-3-(3-methoxyphenyl)-2-phenylpropyl)-2-oxo-2*H*-chromene-3-carbonitrile Major-**3o**

Yellow oil (24.5 mg). The er was determined by UPC^2^ using a chiral Chiralpak IA column gradient from 100% CO_2_ up to 40%; *i*-PrOH, 2.5 mL/min; *t*_R_ = 4.398 (major), *t*_R_ = 4.137 (minor): >99:1 er. [α]_D_^21^ = 12.9 (*c* = 1.0, CHCl_3_). HRMS *m*/*z*: [M + H]^+^ calculated for [C_31_H_23_NO_5_ + H^+^], 490.1576; found, 490.1571. ^1^H NMR (700 MHz, CDCl_3_): δ 10.08 (s, 1H), 7.66–7.63 (m, 1H), 7.60 (d, *J* = 7.8 Hz, 1H), 7.50 (d, *J* = 2.0 Hz, 1H), 7.36 (t, *J* = 7.8 Hz, 1H), 7.32–7.31 (m, 1H), 7.10 (t, *J* = 7.8 Hz, 2H), 7.06–7.05 (m, 1H), 7.04–7.03 (m, 1H), 7.03–7.00 (m, 2H), 6.83–6.81 (m, 1H), 6.79–6.67 (m, 1H), 6.77 (d, *J* = 2.0 Hz, 1H), 6.60–6.57 (m, 1H), 5.21 (d, *J* = 11.1 Hz, 1H), 4.13 (td, *J* = 10.3, 5.2 Hz, 1H), 3.66 (s, 3H), 3.43 (dd, *J* = 13.3, 10.3 Hz, 1H), 3.30 (dd, *J* = 13.3, 5.2 Hz, 1H). ^13^C NMR (176 MHz, CDCl_3_): δ 185.1, 163.5, 161.8, 159.6, 156.5, 153.4, 142.9, 139.8, 138.3, 135.0, 129.7, 128.8 (2C), 128.2 (2C), 127.9, 126.0, 125.3, 123.7, 121.0, 118.0, 117.5, 114.7, 113.4, 112.4, 110.2, 102.9, 55.3, 50.3, 49.5, 37.8.

#### 4-((2*S*,3*R*)-3-(3-Formylfuran-2-yl)-3-(3-methoxyphenyl)-2-phenylpropyl)-2-oxo-2*H*-chromene-3-carbonitrile Minor-**3o**

Light-orange oil (10.7 mg). The er was determined by UPC^2^ using a chiral Chiralpak IA column gradient from 100% CO_2_ up to 40%; *i*-PrOH, 2.5 mL/min; *t*_R_ = 4.231 (major), *t*_R_ = 4.404 (minor): 98:2 er. [α]_D_^21^ = −37.1 (*c* = 1.0, CHCl_3_). HRMS *m*/*z*: [M + H]^+^ calculated for [C_31_H_23_NO_5_ + H^+^], 490.1576; found, 490.1573. ^1^H NMR (700 MHz, CDCl_3_): δ 9.84 (s, 1H), 7.73–7.69 (m, 2H), 7.48–7.43 (m, 2H), 7.40–7.35 (m, 1H), 7.25–7.21 (m, 1H), 7.17–7.16 (m, 1H), 7.14–7.10 (m, 2H), 7.10–7.08 (m, 2H), 7.07–7.04 (m, 1H), 6.87–6.83 (m, 1H), 6.72–6.71 (m, 1H), 6.40–6.37 (m, 1H), 5.18–5.15 (m, 1H), 4.10–4.02 (m, 1H), 3.90 (s, 3H), 3.29–3.24 (s, 1H), 3.23–3.17 (m, 1H). ^13^C NMR (176 MHz, CDCl_3_): δ 184.9, 163.5, 162.0, 158.7, 156.4, 153.3, 142.7, 138.3, 134.8, 130.5, 130.0, 129.1 (2C), 128.5 (2C), 128.4 (2C), 127.2, 125.9, 125.1, 123.4, 117.8, 117.4, 114.0, 113.3, 109.9, 102.8, 55.1, 49.7, 49.6, 37.7.

**3p** (2:1 dr in a crude reaction mixture) was isolated after 5 days in 81% yield (38.4 mg).

#### 4-((2*R*,3*R*)-3-(3-Formylfuran-2-yl)-2-phenyl-3-(*p*-tolyl)­propyl)-2-oxo-2*H*-chromene-3-carbonitrile Major-**3p**

Yellow oil (25.6 mg). The er was determined by UPC^2^ using a chiral Chiralpak IB column gradient from 100% CO_2_ up to 40%; *i*-PrOH, 2.5 mL/min; *t*_R_ = 4.485 (major), *t*_R_ = 4.767 (minor): >99:1 er. [α]_D_^21^ = 64.3 (*c* = 1.0, CHCl_3_). HRMS *m*/*z*: [M + H]^+^ calculated for [C_31_H_23_NO_4_ + H^+^], 474.1626; found, 474.1692. ^1^H NMR (700 MHz, CDCl_3_): δ 10.10 (s, 1H), 7.65 (t, *J* = 7.8 Hz, 1H), 7.61 (d, *J* = 7.8 Hz, 1H), 7.50 (d, *J* = 1.8 Hz, 1H), 7.37 (t, *J* = 7.8 Hz, 1H), 7.31 (d, *J* = 8.3 Hz, 1H), 7.14 (d, *J* = 7.8 Hz, 2H), 7.10–7.08 (m, 2H), 7.04–7.03 (m, 2H), 7.03–7.02 (m, 1H), 6.91 (d, *J* = 7.8 Hz, 2H), 6.76 (d, *J* = 1.8 Hz, 1H), 5.22 (d, *J* = 11.0 Hz, 1H), 4.15 (td, *J* = 10.3, 5.0 Hz, 1H), 3.43 (dd, *J* = 13.3, 10.3 Hz, 1H), 3.31 (dd, *J* = 13.3, 5.0 Hz, 1H), 2.15 (s, 3H). ^13^C NMR (176 MHz, CDCl_3_): δ 185.1, 163.6, 162.4, 156.5, 153.3, 142.8, 138.4, 137.0, 135.2, 135.0, 129.4 (2C), 128.7 (2C), 128.3 (2C), 128.2 (2C), 127.7, 126.0, 125.3, 123.5, 117.9, 117.5, 113.4, 110.0, 102.8, 50.3, 49.0, 37.8, 21.01.

#### 4-((2*S*,3*R*)-3-(3-Formylfuran-2-yl)-2-phenyl-3-(*p*-tolyl)­propyl)-2-oxo-2*H*-chromene-3-carbonitrile Minor-**3p**

Light-orange oil (12.8 mg). The er was determined by UPC^2^ using a chiral Chiralpak IB column gradient from 100% CO_2_ up to 40%; *i*-PrOH, 2.5 mL/min; *t*_R_ = 4.845 (major), *t*_R_ = 4.573 (minor): >99:1 er. [α]_D_^21^ = −94.5 (*c* = 1.0, CHCl_3_). HRMS *m*/*z*: [M + H]^+^ calculated for [C_31_H_23_NO_4_ + H^+^], 474.1626; found, 474.1694. ^1^H NMR (700 MHz, CDCl_3_): δ 9.84 (s, 1H), 7.64 (t, *J* = 7.7 Hz, 1H), 7.57 (d, *J* = 7.7 Hz, 2H), 7.43 (d, *J* = 7.7 Hz, 1H), 7.35–7.33 (m, 1H), 7.29–7.28 (m, 1H), 7.25–7.24 (m, 2H), 7.15 (d, *J* = 1.9 Hz, 1H), 7.12–7.10 (m, 2H), 7.09–7.07 (m, 2H), 7.07–7.06 (m, 1H), 6.37 (d, *J* = 1.9 Hz, 1H), 5.14 (d, *J* = 11.6 Hz, 1H), 4.09–4.06 (m, 1H), 3.34–3.32 (m, 1H), 3.30–3.28 (m, 1H), 2.37 (s, 3H). ^13^C NMR (176 MHz, CDCl_3_): δ 184.9, 163.7, 162.1, 156.4, 153.4, 142.3, 138.6, 138.5, 135.2, 134.9, 130.1 (2C), 128.8 (2C), 128.7 (2C), 128.0, 127.6 (2C), 126.1, 125.2, 122.2, 117.9, 117.4, 113.4, 108.9, 103.1, 50.4, 49.9, 37.7, 21.2.

**3r** (1.5:1 dr in a crude reaction mixture) was isolated after 3 days in 73% yield (34.8 mg).

#### 4-((2*R*,3*R*)-3-(4-Fluorophenyl)-3-(3-formylfuran-2-yl)-2-phenylpropyl)-2-oxo-2*H*-chromene-3-carbonitrile Major-**3r**

Yellow oil (20.9 mg). The er was determined by UPC^2^ using a chiral Chiralpak IB column gradient from 100% CO_2_ up to 40%; *i*-PrOH, 2.5 mL/min; *t*_R_ = 4.334 (major), *t*_R_ = 4.609 (minor): >99:1 er. [α]_D_^21^ = 33.5 (*c* = 1.0, CHCl_3_). HRMS *m*/*z*: [M + H]^+^ calculated for [C_30_H_20_FNO_4_ + H^+^], 478.1376; found, 478.1448. ^1^H NMR (700 MHz, CDCl_3_): δ 10.07 (s, 1H), 7.64 (t, *J* = 7.6 Hz, 1H), 7.59 (d, *J* = 7.6 Hz, 1H), 7.51–7.50 (m, 1H), 7.35 (t, *J* = 7.6 Hz, 1H), 7.32–7.31 (m, 1H), 7.21–7.19 (m, 2H), 7.11–7.09 (m, 2H), 7.09–7.05 (m, 1H), 7.04–7.01 (m, 2H), 6.80–6.77 (m, 3H), 5.28–5.26 (m, 1H), 4.13–4.10 (m, 1H), 3.44–3.41 (m, 1H), 3.30–3.27 (m, 1H). ^13^C NMR (176 MHz, CDCl_3_): δ 185.4, 163.4, 161.8 (d, *J* = 246.1 Hz), 161.3, 156.5, 153.4, 142.9, 138.2, 135.0, 135.0, 134.2 (d, *J* = 2.6 Hz), 130.1 (d, *J* = 7.9 Hz, 2C), 128.9, 128.2 (2C), 127.9, 126.0, 125.3, 123.4, 118.0, 117.5, 115.6 (d, *J* = 21.3 Hz, 2C), 113.4, 110.5, 102.9, 50.4, 48.8, 37.7.

#### 4-((2*S*,3*R*)-3-(4-Fluorophenyl)-3-(3-formylfuran-2-yl)-2-phenylpropyl)-2-oxo-2*H*-chromene-3-carbonitrile Minor-**3r**

Light-orange oil (13.9 mg). The er was determined by UPC^2^ using a chiral Chiralpak IB column gradient from 100% CO_2_ up to 40%; *i*-PrOH, 2.5 mL/min; *t*_R_ = 4.904 (major), *t*_R_ = 4.393 (minor): 98:2 er. [α]_D_^21^ = −54.5 (*c* = 1.0, CHCl_3_). HRMS *m*/*z*: [M + H]^+^ calculated for [C_30_H_20_FNO_4_ + H^+^], 478.1376; found, 478.1449. ^1^H NMR (700 MHz, CDCl_3_): δ 9.81 (s, 1H), 7.73–7.69 (m, 2H), 7.64–7.61 (m, 1H), 7.36–7.35 (m, 1H), 7.32–7.28 (m, 3H), 7.17–7.14 (m, 3H), 7.11–7.07 (m, 2H), 7.07–7.02 (m, 2H), 6.39 (d, *J* = 2.0 Hz, 1H), 5.25 (d, *J* = 11.4 Hz, 1H), 4.05 (td, *J* = 11.4, 4.9 Hz, 1H), 3.31–3.28 (m, 1H), 3.27–3.24 (m, 1H). ^13^C NMR (176 MHz, CDCl_3_): δ 185.2, 163.6, 162.7 (d, *J* = 248.0 Hz), 161.1, 156.4, 153.4, 142.4, 138.2, 135.0, 134.1 (d, *J* = 3.2 Hz), 130.5 (d, *J* = 8.1 Hz, 2C), 128.9 (2C), 128.1, 127.7 (2C), 125.9, 125.2, 122.2, 118.0, 117.4, 116.5 (d, *J* = 21.4 Hz, 2C), 113.4, 109.4, 103.2, 50.4, 49.4, 37.5.

**3s** (1.2:1 dr in a crude reaction mixture) was isolated after 3 days in 71% yield (32.6 mg).

#### 4-((2*R*,3*R*)-3-(5-Formylfuran-2-yl)-2,3-diphenylpropyl)-2-oxo-2*H*-chromene-3-carbonitrile Major-**3s**

Yellow oil (17.8 mg). The er was determined by UPC^2^ using a chiral Chiralpak IA column gradient from 100% CO_2_ up to 40%; acetonitrile, 2.5 mL/min; *t*_R_ = 4.307 (major), *t*_R_ = 4.033 (minor): 63:37 er. [α]_D_^21^ = 59.3 (*c* = 1.0, CHCl_3_). HRMS *m*/*z*: [M + H]^+^ calculated for [C_30_H_21_NO_4_ + H^+^], 460.1470; found, 460.1543. ^1^H NMR (700 MHz, CDCl_3_): δ 9.62 (s, 1H), 7.97–7.95 (m, 1H), 7.72–7.69 (m, 1H), 7.61–7.59 (m, 1H), 7.33–7.32 (m, 1H), 7.17–7.15 (m, 2H), 7.12–7.10 (m, 1H), 7.10–7.08 (m, 2H), 7.08–7.06 (m, 2H), 7.05–7.02 (m, 2H), 6.97–6.92 (m, 2H) 6.62–6.61 (m, 1H), 4.57–4.56 (m, 1H), 4.13–4.09 (m, 1H), 3.46–3.43 (m, 1H), 3.36–3.34 (m, 1H). ^13^C NMR (176 MHz, CDCl_3_): δ 184.9, 163.3, 161.7, 156.4, 153.2, 142.7, 138.1, 134.8, 134.2, 134.1, 128.6 (2C), 128.5 (2C), 128.4 (2C), 128.1 (2C), 127.7, 127.2, 125.8, 125.1, 123.4, 117.8, 117.3, 113.2, 109.9, 50.2, 49.3, 37.5.

#### 4-((2*S*,3*R*)-3-(5-Formylfuran-2-yl)-2,3-diphenylpropyl)-2-oxo-2*H*-chromene-3-carbonitrile Minor-**3s**

Light-orange oil (14.8 mg). The er was determined by UPC^2^ using a chiral Chiralpak IB column gradient from 100% CO_2_ up to 40%; *i*-PrOH, 2.5 mL/min; *t*_R_ = 4.801 (major), *t*_R_ = 4.667 (minor): 63:37 er. [α]_D_^21^ = −72.1 (*c* = 1.0, CHCl_3_). HRMS *m*/*z*: [M + H]^+^ calculated for [C_30_H_21_ClNO_4_ + H^+^], 460.1470; found, 460.1543. ^1^H NMR (700 MHz, CDCl_3_): δ 9.38 (s, 1H), 7.66–7.65 (m, 1H), 7.64–7.62 (m, 2H), 7.48–7.45 (m, 2H), 7.41–7.36 (m, 1H), 7.35–7.34 (m, 1H), 7.33–7.32 (m, 1H), 7.17–7.15 (m, 2H), 7.14–7.12 (m, 2H), 7.07–7.06 (m, 2H), 6.88 (d, *J* = 3.6 Hz, 1H), 6.07 (d, *J* = 3.6 Hz, 1H), 4.54 (d, *J* = 11.3 Hz, 1H), 3.95–3.91 (m, 1H), 3.31–3.28 (m, 1H), 3.28–3.27 (m, 1H). ^13^C NMR (176 MHz, CDCl_3_): δ 177.0, 163.4, 161.5, 156.4, 153.4, 152.1, 138.6, 138.5, 135.0, 129.5 (2C), 129.0 (2C), 128.8 (2C), 128.6, 128.1, 127.9 (2C), 126.0, 125.5, 118.0, 117.2, 113.5, 110.5, 103.3, 52.5, 50.8, 37.9.

### General Procedure for Synthesis of **3a** on a 1 mmol Scale

In an ordinary 10 mL flask with a magnetic stirring bar 2-benzyl-3-furfural **1a** (1.0 mmol, 1.0 equiv) and 4-(alk-1-en-1-yl)-3-cyanocoumarin **2a** (1.0 mmol, 1.0 equiv). The starting materials were dissolved in CH_2_Cl_2_ (2.0 mL). Catalyst—(*S*)-2-(((methyldiphenylsilyl)­oxy)­diphenylmethyl)­pyrrolidine **4e** (0.3 mmol, 0.3 equiv) and *o*-fluorobenzoic acid (0.4 mmol, 0.4 equiv) were added. The resulting mixture was stirred for 72 h in 40 °C, and conversion of the starting material **1** was controlled by ^1^H NMR spectroscopy. Then, the reaction mixture was directly subjected to column chromatography on silica gel (hexanes: ethyl acetate 4:1 to 3:2) to obtain pure product **3a** as a light-yellow oil with a 74% yield.

## Supplementary Material



## Data Availability

The data underlying this study are available in the published article, in its Supporting Information, and openly available in Lodz University of Technology Research Data Repository at 10.34658/RDB.EARUJF.

## Notes

The Supporting Information containing additional experimental procedures, characterization of the products, NMR data, and UPC^2^ traces (PDF)
