# Supplementary material for: Aminocatalytic 1,6-Addition of 2‑Benzyl-3-furaldehyde to 3‑Cyano-4-styrylcoumarins: A Dearomative Approach for the Synthesis of Furan–Coumarin Hybrids
Source: J Org Chem. 2025 Oct 11;90(42):14931–42. doi: 10.1021/acs.joc.5c01406 (PMC12570265; doi:10.1021/acs.joc.5c01406)
Supplement: Supplementary file 1 [file jo5c01406_si_001.pdf]

Aminocatalytic 1,6-Addition of 2-Benzyl-3-Furaldehyde to 3-Cyano-4-styrylcoumarins: A Dearomative Approach for the Synthesis of Furan-Coumarin Hybrids

Aleksandra Topolska,[a] Artur Przydacz[a], Lesław Sieroń,[b] Anna Skrzyńska,[a] Alberto Fraile,[c,d] Jose Alemán,[c,d]* and Łukasz Albrecht[a]*

[a] Institute of Organic Chemistry Faculty of Chemistry, Lodz University of Technology
Żeromskiego 116, 90-924 Łódź, Poland
E-mail: lukasz.albrecht@p.lodz.pl

[b] Institute of General and Ecological Chemistry, Faculty of Chemistry, Lodz University of Technology, Żeromskiego 116, Łódź, 90-924, Poland

[c] Organic Chemistry Department, Universidad Autónoma de Madrid
28049 Madrid, Spain
E-mail: jose.aleman@uam.es

[d] Institute for Advanced Research in Chemical Sciences (IAdChem),
Universidad Autónoma de Madrid
28049 Madrid, Spain
E-mail: jose.aleman@uam.es

Table of contents

1. Materials and methods — S2
2. General procedure for synthesis of **3** — S3
3. Characterization data of products **3** — S4
4. Synhesis of 4-((2*R*,3*R*)-3-(3-formylfuran-2-yl)-2,3-diphenylpropyl)-2-oxo-2*H*-chromene-3-carbonitrile major – **3a** and 4-((2*S*,3*R*)-3-(3-formylfuran-2-yl)-2,3-diphenylpropyl)-2-oxo-2*H*-chromene-3-carbonitrile minor – **3a** on a 1 mmol scale — S23
5. Synthesis of 4-((2*S*,3*R*)-3-(3-(2,2-dibromovinyl)furan-2-yl)-3-phenyl-2-(*p*-tolyl)propyl)-2-oxo-2*H*-chromene-3-carbonitrile **5** — S25
6. Synthesis of 4-((6*S*,7*R*)-6,7-diphenyl-6,7-dihydrobenzofuran-5-yl)-2*H*-chromen-2-one **6** — S26
7. Crystal and X-ray data for **3a** – major and **3d** - minor — S27
8. NMR data — S30
9. UPC^2 data — S70

1. Materials and methods

NMR spectra were acquired on a Bruker Ultra Shield 700 instrument, running at 700 MHz for ^{1}H and 176 MHz for $^{13}C\{^{1}H\}$, respectively. Chemical shifts (δ) are reported in ppm relative to residual solvent signals ($CDCl_3$: 7.26 ppm for ^{1}H NMR, 77.16 ppm for ^{13}C NMR). Highresolution mass spectra (HRMS) were obtained on Bruker ESI-Q-TOF Impact II spectrometer using electrospray (ESI+) ionization. Optical rotations were measured on a Perkin-Elmer 241 polarimeter and $[\alpha]_D$ values are given in deg•cm•g^{-1}•dm^{-1}; concentration *c* is listed in g•(100 mL)$^{-1}$. Analytical thin layer chromatography (TLC) was performed using pre-coated aluminum-backed plates (Merck Kieselgel 60 F254) and visualized by ultraviolet irradiation or Hanessian's stain. Unless otherwise noted, analytical grade solvents and commercially available reagents were used without further purification. For column chromatography silica gel (Silica gel 60, 230-400 mesh, Fluka) was applied. The enantiomeric ratios (er) of the products were determined by Ultra Performance Convergence Chromatography (UPC^2) using Daicel Chiralpak IA or IB column as chiral stationary phases. 2-benzyl-3-furfurals **1a-h**[1a], 2-allyl-3-furfural **1i**[1b] and 4-(alk-1-en-1-yl)-3-cyanocoumarins **2**[2] were synthesized according to the literature procedures from the corresponding starting materials. The catalysts 4a-d were purchased from Merck and used without further purification. Catalysts 4e was synthesized according to the literature procedure.[3] The enantiomeric samples of products **3** for chiral UPC^2 separation studies were prepared using (*S*)-2-(((methyldiphenylsilyl)oxy)diphenylmethyl)pyrrolidine **4e.** The racemic samples of products **3** for chiral UPC^2 separation studies were prepared using mixture of commercially available compounds (*S*)-(–)-*α*,*α*-diphenyl-2-pyrrolidinemethanol trimethylsilyl ether and (*R*)-(+)-*α*,*α*-diphenyl-2-pyrrolidinemethanol trimethylsilyl ether under the general reaction conditions.

1a. Bojanowski, J.; Skrzyńska, A.; Albrecht, A. Dearomatizative and Decarboxylative Reaction Cascade in the Aminocatalytic Synthesis of 3,4-Dihydrocoumarins. *Asian J. Org. Chem*. **2019**, *8*, 844-848.

1b. Kelly, A.-R.; Kerrigan, M.-H.; Walsh, P.-J. Addition/Oxidative Rearrangement of 3-Furfurals and 3-Furyl Imines: New Approaches to Substituted Furans and Pyrroles. *J. Am. Chem. Soc.* **2008**, *130*, 4097–4104.

2. Romaniszyn, M.; Gronowska, K.; Albrecht, Ł. Remote Functionalization of 4-(Alk-1-en-1-yl)-3-Cyanocoumarins via the Asymmetric Organocatalytic 1,6-Addition. *Adv. Synth. Catal.* **2021**, *363*, 5116– 5121.

3. Kemppainen, E. K.; Sahoo, G.; Valkonen, A.; Pihko, P. M. Mukaiyama–Michael Reactions with Acrolein and Methacrolein: A Catalytic Enantioselective Synthesis of the C17–C28 Fragment of aPectenotoxins. *Org. Lett.* **2012**, *14*, 1086-1089.

2. General procedure for synthesis of 3

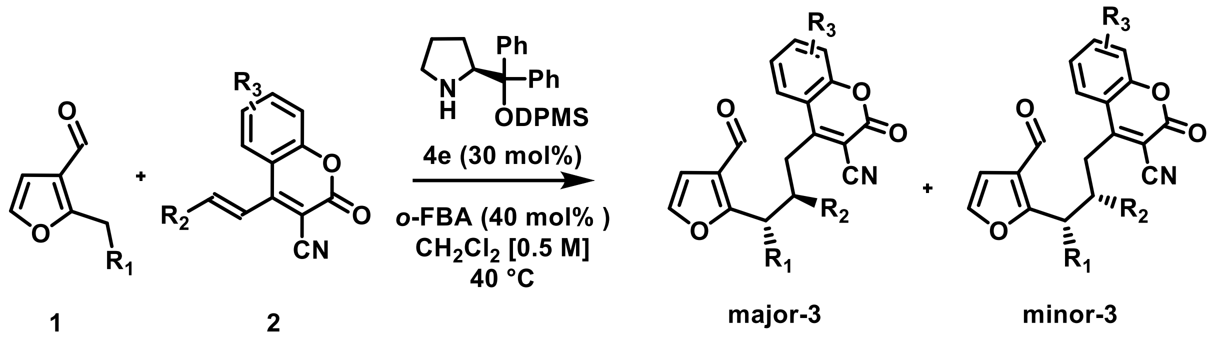

In an ordinary 4 mL glass vial equipped with a magnetic stirring bar 2-benzyl-3-furfural **1** (0.1 mmol, 1.0 equiv.) and 4-(alk-1-en-1-yl)-3-cyanocoumarin **2** (0.1 mmol, 1.0 equiv.) were dissolved in CH_2Cl_2 (0.2 mL). (*S*)-2-(((methyldiphenylsilyl)oxy)diphenylmethyl)pyrrolidine **4e** (13.5 mg, 0.03 mmol, 0.3 equiv.) and *o*-fluorobenzoic acid (5.6 mg, 0.04 mmol, 0.4 equiv.) were added and the reaction mixture was stirred in 40 °C for 72 hours. The progress of the reaction was controlled by ^{1}H NMR spectroscopy. After full conversion of the starting material **1**, the reaction mixture was directly subjected to column chromatography on silica gel (hexane : ethyl acetate 4:1 to 3:2) to afford pure product major – **3** and minor – **3**.

3. Characterization data of products 3

Following the general procedure product **3a** (2.2:1 dr in a crude reaction mixture) was isolated after 3 days in 80% yield (36.7 mg) by column chromatography on silica gel (hexane : ethyl acetate 4:1 to 3:2)

4-((2*R*,3*R*)-3-(3-Formylfuran-2-yl)-2,3-diphenylpropyl)-2-oxo-2*H*-chromene-3-carbonitrile major – 3a

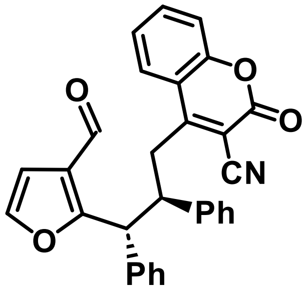

Major diasteroisomer **3a** was isolated as light-yellow oil (25.2 mg). The er was determined by UPC[2] using a chiral Chiralpack IB column gradient from 100% CO_2 up to 40%; *i*-PrOH, 2.5 mL/min; t_R = 4.491 (major), t_R = 4.761 (minor) : >99:1 er. $[\alpha]_D^{21}$ = + 29.5 (*c* = 1.0, $CHCl_3$). HRMS m/z $[M+H]^+$ Calculated for $[C_{30}H_{21}NO_4+H^+]$: 460.1543; found 460.1538.

^{1}H NMR (700 MHz, $CDCl_3$) δ 10.09 (s, 1H), 7.65 (t, *J* = 7.8 Hz, 1H), 7.59 (d, *J* = 7.8 Hz, 1H), 7.51 (d, *J* = 1.9 Hz, 1H), 7.38 – 7.34 (m, 1H), 7.32 (d, *J* = 7.8 Hz, 1H), 7.25 – 7.22 (m, 2H), 7.13 – 7.11 (m, 2H), 7.10 – 7.08 (m, 2H), 7.07 – 7.04 (m, 2H), 7.03 – 7.00 (m, 2H), 6.77 (d, *J* = 1.9 Hz, 1H), 5.24 (d, *J* = 11.0 Hz, 1H), 4.19 – 4.11 (m, 1H), 3.47 – 3.41 (m, 1H), 3.34 – 3.29 (m, 1H).

^{13}C{^{1}H} NMR (176 MHz, $CDCl_3$) δ 185.1, 163.5, 161.9, 156.5, 153.4, 142.9 (2C), 138.3, 135.0 128.8 (2C), 128.7 (2C), 128.5(2C), 128.3 (2C), 127.9, 127.4, 126.0, 125.3, 123.6, 118.0, 117.6, 113.4, 110.2, 103.0, 50.4, 49.6, 37.7.

4-((2*S*,3*R*)-3-(3-Formylfuran-2-yl)-2,3-diphenylpropyl)-2-oxo-2*H*-chromene-3-carbonitrile minor – 3a

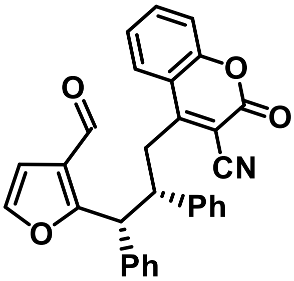

Minor diastereoisomer **3** was isolated as light-yellow oil (11.5 mg). The er was determined by UPC[2] using a chiral Chiralpack IB column gradient from 100% CO_2 up to 40%; *i*-PrOH, 2.5 mL/min; t_R = 4.835 (major), t_R = 4.634 (minor) : 98:2 er. $[\alpha]_D^{21}$ = - 54.7 (*c* = 1.0, $CHCl_3$). HRMS m/z $[M+H]^+$ calculated for $[C_{30}H_{21}NO_4+H^+]$: 460.1543; found 460.1537.

^{1}H NMR (700 MHz, $CDCl_3$) δ 9.84 (s, 1H), 7.72 (d, *J* = 7.6 Hz, 2H), 7.63 (t, *J* = 7.6 Hz, 1H), 7.46 (t, *J* = 7.6 Hz, 2H), 7.40 – 7.35 (m, 2H), 7.33 – 7.27 (m, 2H), 7.16 (d, *J* = 1.9 Hz, 1H), 7.13 – 7.10 (m, 2H), 7.10 – 7.05 (m, 3H), 6.39 (d, *J* = 1.9 Hz, 1H), 5.21 – 5.19 (m, 1H), 4.10 – 4.05 (m, 1H), 3.32 – 3.29 (m, 2H).

^{13}C{^{1}H} NMR (176 MHz, $CDCl_3$) δ 185.0, 163.6, 161.6, 156.4, 153.4, 142.4 (2C), 138.5, 138.4, 134.9, 129.5 (2C), 128.9 (2C), 128.8 (2C), 128.6, 128.1, 127.7, 126.1, 125.2, 122.4, 118.0, 117.4, 113.4, 109.1, 103.2, 50.4, 50.3, 37.7.

Following the general procedure product **3b** (1.9:1 dr in a crude reaction mixture) was isolated after 5 days in 52% yield (24.8 mg) by column chromatography on silica gel (hexane : ethyl acetate 4:1 to 3:2).

4-((2*R*,3*R*)-2-(3-Fluorophenyl)-3-(3-formylfuran-2-yl)-3-phenylpropyl)-2-oxo-2*H*-chromene-3-carbonitrile major – 3b

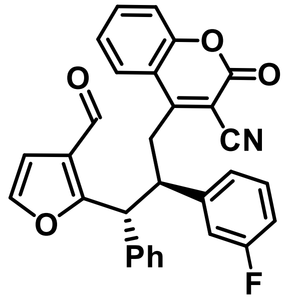

Major diasteroisomer **3b** was isolated as light-orange oil (16.2 mg). The er was determined by UPC^2 using a chiral Chiralpack IB column gradient from 100% CO_2 up to 40%; *i*-PrOH, 2.5 mL/min; t_R = 4.439 (major), t_R = 4.698 (minor) : 99:1 er. $[\alpha]_D^{21}$ = + 81.5 (*c* = 1.0, $CHCl_3$). HRMS m/z $[M+H]^+$ calculated for $[C_{30}H_{20}FNO_4+H^+]$: 478.1449; found 478.1452.

^{1}H NMR (700 MHz, $CDCl_3$) δ 10.06 (s, 1H), 7.67 – 7.65 (m, 1H), 7.59 – 7.57 (m, 1H), 7.50 (d, *J* = 2.0 Hz, 1H), 7.38 – 7.36 (m, 1H), 7.34 – 7.33 (m, 1H), 7.25 – 7.23 (m, 2H), 7.15 – 7.12 (m, 2H), 7.10 – 7.08 (m, 1H), 7.08 – 7.06 (m, 1H), 6.87 – 6.86 (m, 1H), 6.76 (d, *J* = 2.0 Hz, 1H), 6.75 – 6.71 (m, 2H), 5.23 – 5.21 (m, 1H), 4.18 – 4.14 (m, 1H), 3.44 – 3.41 (m, 1H), 3.32 – 3.29 (m, 1H).

^{13}C{^{1}H} NMR (176 MHz, $CDCl_3$) δ 185.2, 163.1, 162,7 (d, *J* = 247.7 Hz), 161.2, 156.4, 153.5, 142.9, 141.1 (d, *J* = 7.1 Hz), 138.0, 135.1, 130.5 (d, *J* = 8.3 Hz), 128.8 (2C), 128.5 (2C), 127.6, 125.8, 125.4, 124.0 (d, *J* = 2.8 Hz), 123.6, 118.1, 117.4, 115.4 (d, *J* = 21.7 Hz), 114.9 (d, *J* = 21.0 Hz), 113.4, 110.4, 103.0, 50.0, 49.5, 37.5.

4-((2*S*,3*R*)-2-(3-Fluorophenyl)-3-(3-formylfuran-2-yl)-3-phenylpropyl)-2-oxo-2*H*-chromene-3-carbonitrile minor – 3b

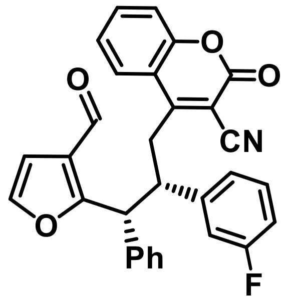

Minor diastereoisomer **3b** was isolated as light-yellow oil (8.6 mg). The er was determined by UPC^2 using a chiral Chiralpack IB column gradient from 100% CO_2 up to 40%; *i*-PrOH, 2.5 mL/min; t_R = 4.774 (major), t_R = 4.467 (minor) : 97:3 er. $[\alpha]_D^{21}$ = - 105.3 (*c* = 1.0, $CHCl_3$). HRMS m/z $[M+H]^+$ calculated for $[C_{30}H_{20}FNO_4+H^+]$: 478.1449; found 478.1451.

^{1}H NMR (700 MHz, $CDCl_3$) δ 9.85 (s, 1H), 7.71 – 7.69 (m, 2H), 7.66 – 7.63 (m, 1H), 7.47 – 7.45 (m, 2H), 7.40 – 7.37 (m, 1H), 7.32 – 7.31 (m, 2H), 7.31 – 7.30 (m, 1H), 7.19 (d, *J* = 2.0 Hz, 1H), 7.14 – 7.11 (m, 1H), 6.96 – 6.94 (m, 1H), 6.81 – 6.75 (m, 2H), 6.42 (d, *J* = 2.0 Hz, 1H), 5.22 – 5.20 (m, 1H), 4.09 – 4.05 (m, 1H), 3.32 – 3.29 (m, 2H).

^{13}C{^{1}H} NMR (176 MHz, $CDCl_3$) δ 185.1, 163.1, 162.7 (d, *J* = 247.6 Hz), 161.0, 156.3, 153.5, 142.5, 141.2 (d, *J* = 6.7 Hz), 138.1, 135.1, 130.6 (d, *J* = 8.2 Hz), 129.6 (2C), 128.9, 128.8, 128.7, 125.9, 125.3, 123.4, 122.3, 118.1, 117.2, 115.0 (d, *J* = 21.8 Hz), 114.9 (d, *J* = 21.0 Hz), 113.3, 109.4, 103.2, 50.1, 50.0, 37.4.

Following the general procedure product **3c** (1.3:1 dr in a crude reaction mixture) was isolated after 7 days in 86% yield (42.4 mg) by column chromatography on silica gel (hexane : ethyl acetate 4:1 to 3:2).

4-((2*R*,3*R*)-2-(2-Chlorophenyl)-3-(3-formylfuran-2-yl)-3-phenylpropyl)-2-oxo-2*H*-chromene-3-carbonitrile major – 3c

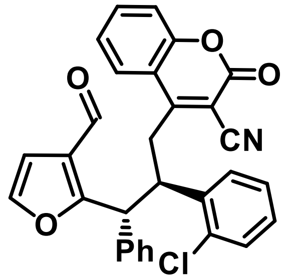

Major diasteroisomer **3c** was isolated as yellow oil (23.6 mg). The er was determined by UPC[2] using a chiral Chiralpack IB column gradient from 100% CO_2 up to 40%; *i*-PrOH, 2.5 mL/min; t_R = 4.750 (major), t_R = 5.067 (minor) : 99:1 er. $[\alpha]_D^{21}$ = + 58.1 (c = 1.0, $CHCl_3$). HRMS m/z $[M+H]^+$ calculated for $[C_{30}H_{20}ClNO_4+H^+]$: 494.1154; found 494.1151.

^{1}H NMR (700 MHz, $CDCl_3$) δ 10.11 (s, 1H), 7.65 (t, J = 7.6 Hz, 1H), 7.59 (d, J = 7.8 Hz, 1H), 7.56 (d, J = 7.8 Hz, 1H), 7.53 – 7.52 (m, 1H), 7.36 – 7.32 (m, 2H), 7.32 – 7.30 (m, 2H), 7.26 – 7.23 (m, 1H), 7.13 (t, J = 7.8 Hz, 2H), 7.08 – 7.06 (m, 1H), 7.01 – 7.00 (m, 2H), 6.81 – 6.80 (m, 1H), 5.29 (d, J = 11.1 Hz, 1H), 4.97 – 4.93 (m, 1H), 3.43 – 3.39 (m, 1H), 3.38 – 3.35 (m, 1H).

^{13}C{^{1}H} NMR (176 MHz, $CDCl_3$) δ 185.1, 162.5, 161.4, 156.5, 153.4, 143.0 (2C), 137.8, 136.4, 134.9, 134.4, 129.5, 129.0, 128.9, 128.7 (2C), 128.4 (2C), 127.9, 127.6, 126.0, 125.3, 123.6, 117.9, 117.8, 110.4, 103.2, 49.3, 44.7, 37.5.

4-((2*S*,3*R*)-2-(2-Chlorophenyl)-3-(3-formylfuran-2-yl)-3-phenylpropyl)-2-oxo-2*H*-chromene-3-carbonitrile minor – 3c

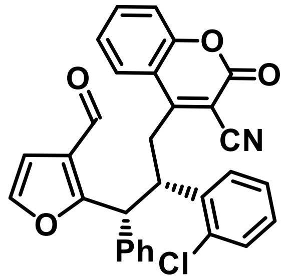

Minor diastereoisomer **3c** was isolated as light-orange oil (18.8 mg). The er was determined by UPC[2] using a chiral Chiralpack IB column gradient from 100% CO_2 up to 40%; *i*-PrOH, 2.5 mL/min; t_R = 5.187 (major), t_R = 4.764 (minor) : 97:3 er. $[\alpha]_D^{21}$ = - 76.0 (c = 1.0, $CHCl_3$). HRMS m/z $[M+H]^+$ calculated for $[C_{30}H_{20}ClNO_4+H^+]$: 494.1154; found 494.1152.

^{1}H NMR (700 MHz, $CDCl_3$) δ 9.86 (s, 1H), 7.76 – 7.73 (m, 2H), 7.64 – 7.60 (m, 2H), 7.50 – 7.48 (m, 2H), 7.43 – 7.39 (m, 1H), 7.30 – 7.27 (m, 3H), 7.25 – 7.23 (m, 1H), 7.16 (d, J = 2.0 Hz, 1H), 7.10 – 7.04 (m, 2H), 6.40 (d, J = 2.0 Hz, 1H), 5.26 – 5.24 (m, 1H), 4.80 – 4.76 (m, 1H), 3.38 – 3.36 (m, 1H), 3.28 – 3.24 (m, 1H).

^{13}C{^{1}H} NMR (176 MHz, $CDCl_3$) δ 185.1, 162.4, 161.0, 156.4, 153.4, 142.7, 138.3, 136.4, 134.9, 134.3, 129.6, 129.5 (2C), 129.1, 129.0 (2C), 128.8 (2C), 128.0, 126.0, 125.1, 122.2, 117.9, 117.8, 113.2, 109.3, 103.2, 49.8, 45.1, 37.7.

Following the general procedure product **3d** (1.6:1 dr in a crude reaction mixture) was isolated after 3 days in 90% yield (44.4 mg) by column chromatography on silica gel (hexane : ethyl acetate 4:1 to 3:2).

4-((2*R*,3*R*)-2-(3-Chlorophenyl)-3-(3-formylfuran-2-yl)-3-phenylpropyl)-2-oxo-2*H*-chromene-3-carbonitrile major – 3d

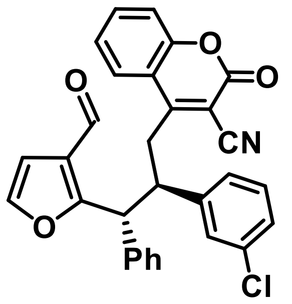

Major diasteroisomer **3d** was isolated as yellow oil (27.4 mg). The er was determined by UPC[2] using a chiral Chiralpack IB column gradient from 100% CO_2 up to 40%; *i*-PrOH, 2.5 mL/min; t_R = 4.158 (minor), t_R = 4.279 (major) : >99:1 er. $[\alpha]_D^{21}$ = + 110.1 (*c* = 1.0, $CHCl_3$). HRMS m/z $[M+H]^+$ calculated for $[C_{30}H_{20}ClNO_4+H^+]$: 494.1154; found 494.1152.

^{1}H NMR (700 MHz, $CDCl_3$) δ 10.06 (s, 1H), 7.67 – 7.64 (m, 1H), 7.58 – 7.56 (m, 1H), 7.50 (d, *J* = 2.0 Hz, 1H), 7.38 – 7.36 (m, 1H), 7.34 – 7.32 (m, 1H), 7.25 – 7.23 (m, 2H), 7.15 – 7.13 (m, 2H), 7.09 – 7.06 (m, 1H), 7.03 – 7.02 (m, 1H), 7.02 – 7.01 (m, 2H), 6.95 – 6.94 (m, 1H), 6.76 (d, *J* = 2.0 Hz, 1H), 5.23 – 5.21 (m, 1H), 4.16 – 4.12 (m, 1H), 3.44 – 3.41 (m, 1H), 3.32 – 3.29 (m, 1H).

$^{13}C\{^1H\}$ NMR (176 MHz, $CDCl_3$) δ 185.2, 163.1, 161.2, 156.5, 153.5, 143.0, 140.6, 137.9, 135.2, 134.6, 130.1, 128.9 (2C), 128.5 (2C), 128.4, 128.1, 127.6, 126.5, 125.9, 125.4, 123.6, 118.1, 117.4, 113.4, 110.4, 102.9, 50.0, 49.5, 37.4.

4-((2*S*,3*R*)-2-(3-Chlorophenyl)-3-(3-formylfuran-2-yl)-3-phenylpropyl)-2-oxo-2*H*-chromene-3-carbonitrile minor – 3d

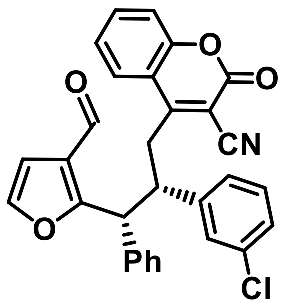

Minor diastereoisomer **3d** was isolated as light-orange oil (17.1 mg). The er was determined by UPC[2] using a chiral Chiralpack IA column gradient from 100% CO_2 up to 40%; *i*-PrOH, 2.5 mL/min; t_R = 4.076 (minor), t_R = 4.270 (major) : 99:1 er. $[\alpha]_D^{21}$ = - 135.0 (c = 1.0, $CHCl_3$). HRMS m/z $[M+H]^+$ calculated for $[C_{30}H_{20}ClNO_4+H^+]$: 494.1154; found 494.1153.

^{1}H NMR (700 MHz, $CDCl_3$) δ 9.84 (s, 1H), 7.73 – 7.70 (m, 2H), 7.64 – 7.62 (m, 1H), 7.53 – 7.46 (m, 2H), 7.43 – 7.41 (m, 1H), 7.40 – 7.36 (m, 1H), 7.34 – 7.32 (m, 1H), 7.30 – 7.28 (m, 2H), 7.1 (d, *J* = 2.0 Hz, 1H), 6.96 – 6.94 (m, 1H), 6.88 – 6.87 (m, 2H), 6.39 (d, *J* = 2.0 Hz, 1H), 5.20 – 5.15 (m, 1H), 4.04 – 4.01 (m, 1H), 3.33 – 3.27 (m, 2H).

$^{13}C\{^1H\}$ NMR (176 MHz, $CDCl_3$) δ 184.8, 163.6, 161.8, 156.5, 153.4, 142.3, 138.6, 138.4, 138.2, 134.8, 129.5 (2C), 128.9 (2C), 128.6, 128.5, 128.1, 126.1, 125.2, 124.9, 122.4, 118.0 (2C), 117.4, 113.5, 109.0 (2C), 103.2, 50.4, 37.7, 21.4.

Following the general procedure product **3e** (1:1 dr in a crude reaction mixture) was isolated after 7 days in 63% yield (31 mg) by column chromatography on silica gel (hexane : ethyl acetate 4:1 to 3:2).

4-((2*R*,3*R*)-2-(4-Chlorophenyl)-3-(3-formylfuran-2-yl)-3-phenylpropyl)-2-oxo-2*H*-chromene-3-carbonitrile major – 3e

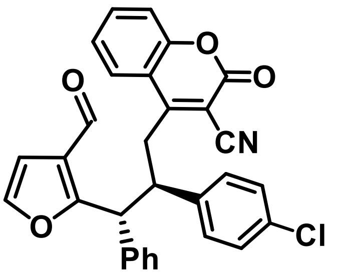

Major diasteroisomer **3e** was isolated as yellow oil (15.7 mg). The er was determined by UPC[2] using a chiral Chiralpack IB column gradient from 100% CO_2 up to 40%; *i*-PrOH, 2.5 mL/min; t_R = 4.602 (major), t_R = 5.044 (minor) : 98:2 er. $[\alpha]_D^{21}$ = + 86.4 (c = 1.0, $CHCl_3$). HRMS m/z $[M+H]^+$ calculated for $[C_{30}H_{20}ClNO_4+H^+]$: 494.1154; found 494.1148.

^{1}H NMR (700 MHz, $CDCl_3$) δ 10.06 (s, 1H), 7.68 – 7.65 (m, 1H), 7.59 – 7.57 (m, 1H), 7.50 (d, *J* = 2.0 Hz, 1H), 7.38 – 7.36 (m, 1H), 7.34 – 7.33 (m, 1H), 7.24 – 7.21 (m, 2H), 7.14 – 7.12 (m, 2H), 7.09 – 7.07 (m, 2H), 7.07 – 7.06 (m, 1H), 6.98 – 6.97 (m, 2H), 6.76 (d, *J* = 2.0 Hz, 1H), 5.22 – 5.20 (m, 1H), 4.17 – 4.14 (m, 1H), 3.43 – 3.40 (m, 1H), 3.31 – 3.28 (m, 1H).

^{13}C{^{1}H} NMR (176 MHz, $CDCl_3$) δ 185.2, 163.2, 161.3, 156.4, 153.5, 142.9, 138.0, 137.0, 135.1, 133.6, 129.6 (2C), 129.0 (2C), 128.9 (2C), 128.5 (2C), 127.6, 125.9, 125.4, 123.6, 118.1, 117.4, 113.4, 110.4, 103.0, 49.8, 49.6, 37.5.

4-((2*S*,3*R*)-2-(4-Chlorophenyl)-3-(3-formylfuran-2-yl)-3-phenylpropyl)-2-oxo-2*H*-chromene-3-carbonitrile minor – 3e

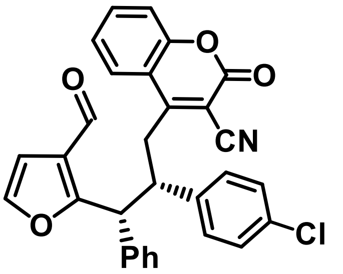

Minor diastereoisomer **3e** was isolated as light-orange oil (15.3 mg). The er was determined by UPC[2] using a chiral Chiralpack IA column gradient from 100% CO_2 up to 40%; *i*-PrOH, 2.5 mL/min; t_R = 4.335 (major), t_R = 4.636 (minor) : 95:5 er. $[\alpha]_D^{21}$ = - 127.2 (c = 1.0, $CHCl_3$). HRMS m/z $[M+H]^+$ calculated for $[C_{30}H_{20}ClNO_4+H^+]$: 494.1154; found 494.1150.

^{1}H NMR (700 MHz, $CDCl_3$) δ 9.83 (s, 1H), 7.71 – 7.69 (m, 2H), 7.66 – 7.63 (m, 1H), 7.47 – 7.45 (m, 2H), 7.40 – 7.36 (m, 1H), 7.33 – 7.31 (m, 2H), 7.31 – 7.29 (m, 1H), 7.17 (d, *J* = 2.0 Hz, 1H), 7.10 – 7.09 (m, 2H), 7.06 – 7.03 (m, 2H), 6.42 (d, *J* = 2.0 Hz, 1H), 5.21 (d, *J* = 11.7 Hz, 1H), 4.08 – 4.04 (m, 1H), 3.29 – 3.27 (m, 2H).

^{13}C{^{1}H} NMR (176 MHz, $CDCl_3$) δ 185.2, 163.2, 160.9, 156.3, 153.5, 142.5 (2C), 138.2, 137.1, 135.1 (2C), 133.8, 129.6 (2C), 129.1 (2C), 128.8 (2C), 128.7, 125.9, 125.3, 122.3, 118.1, 117.2, 113.4, 109.6, 103.2, 50.2, 49.8, 37.5.

Following the general procedure product **3f** (1.4:1 dr in a crude reaction mixture) was isolated after 7 days in 51% yield (27.4 mg) by column chromatography on silica gel (hexane : ethyl acetate 4:1 to 3:2).

4-((2*R*,3*R*)-2-(4-Bromophenyl)-3-(3-formylfuran-2-yl)-3-phenylpropyl)-2-oxo-2*H*-chromene-3-carbonitrile major – 3f

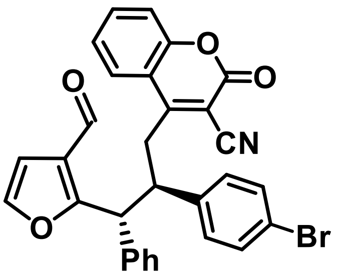

Major diasteroisomer **3f** was isolated as yellow oil (16.2 mg). The er was determined by UPC^2 using a chiral Chiralpack IB column gradient from 100% CO_2 up to 40%; *i*-PrOH, 2.5 mL/min; t_R = 4.786 (major), t_R = 5.316 (minor) : 99:1 er. $[\alpha]_D^{21}$ = + 60.8 (c = 1.0, $CHCl_3$). HRMS m/z $[M+H]^+$ calculated for $[C_{30}H_{20}BrNO_4H^+]$: 538.0649; found 538.0645.

^{1}H NMR (700 MHz, $CDCl_3$) δ 10.06 (s, 1H), 7.68 – 7.66 (m, 1H), 7.59 – 7.58 (m, 1H), 7.50 (d, *J* = 1.9 Hz, 1H), 7.38 – 7.36 (m, 1H), 7.34 – 7.33 (m, 1H), 7.23 – 7.22 (m, 2H), 7.22 – 7.20 (m, 2H), 7.14 – 7.12 (m, 2H), 7.09 – 7.07 (m, 1H), 6.93 – 6.92 (m, 2H), 6.76 (d, *J* = 1.8 Hz, 1H), 5.22 – 5.20 (m, 1H), 4.17 – 4.13 (m, 1H), 3.43 – 3.40 (m, 1H), 3.31 – 3.29 (m, 1H).

^{13}C{^{1}H} NMR (176 MHz, $CDCl_3$) δ 185.3, 163.2, 161.3, 156.4, 153.5, 142.9, 138.0, 137.5, 135.2, 132.0 (2C), 129.9 (2C), 128.9 (2C), 128.5 (2C), 127.6, 125.9, 125.4, 123.6, 121.7, 118.1, 117.4, 113.4, 110.4, 103.0, 49.8, 49.6, 37.5.

4-((2*S*,3*R*)-2-(4-Bromophenyl)-3-(3-formylfuran-2-yl)-3-phenylpropyl)-2-oxo-2*H*-chromene-3-carbonitrile minor – 3f

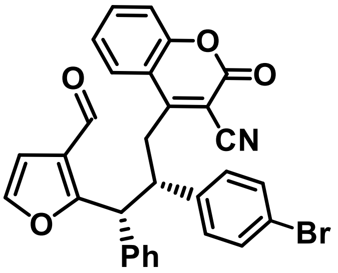

Minor diastereoisomer **3f** was isolated as light-orange oil (11.2 mg). The er was determined by UPC^2 using a chiral Chiralpack IB column gradient from 100% CO_2 up to 40%; *i*-PrOH, 2.5 mL/min; t_R = 4.774 (major), t_R = 4.467 (minor) : 97:3 er. $[\alpha]_D^{21}$ = - 77.8 (c = 1.0, $CHCl_3$). HRMS m/z $[M+H]^+$ calculated for $[C_{30}H_{20}BrNO_4H^+]$: 538.0649; found 538.0648.

^{1}H NMR (700 MHz, $CDCl_3$) δ 9.83 (s, 1H), 7.70 – 7.69 (m, 2H), 7.66 – 7.64 (m, 1H), 7.47 – 7.45 (m, 2H), 7.39 – 7.37 (m, 1H), 7.32 – 7.31 (m, 1H), 7.31 – 7.30 (m, 2H), 7.25 – 7.24 (m, 2H), 7.18 – 7.17 (m, 1H), 7.00 – 6.98 (m, 2H), 6.43 – 6.41 (m, 1H), 5.22 – 5.21 (m, 1H), 4.09 – 4.01 (m, 1H), 3.29 – 3.28 (m, 1H), 3.27 – 3.26 (m, 1H).

^{13}C{^{1}H} NMR (176 MHz, $CDCl_3$) δ 185.3, 163.2, 160.9, 156.3, 153.5, 142.5, 138.1, 137.6, 135.1, 132.0 (2C), 129.6 (2C), 129.4 (2C), 128.8 (2C), 128.7, 125.9, 125.3, 122.3, 121.9, 118.1, 117.2, 113.4, 109.6, 103.2, 50.1, 49.8, 37.4.

Following the general procedure product **3g** (1.2:1 dr in a crude reaction mixture) was isolated after 7 days in 37% yield (19.5 mg) by column chromatography on silica gel (hexane : ethyl acetate 4:1 to 3:2).

4-((2*R*,3*R*)-3-(3-Formylfuran-2-yl)-3-phenyl-2-(4-(trifluoromethyl)phenyl)propyl)-2-oxo-2*H*-chromene-3-carbonitrile major – 3g

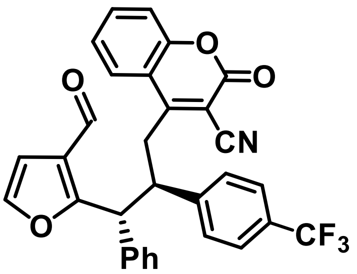

Major diasteroisomer **3g** was isolated as yellow oil (10.6 mg). The er was determined by UPC^2 using a chiral Chiralpack IB column gradient from 100% CO_2 up to 40%; *i*-PrOH, 2.5 mL/min; t_R = 3.900 (major), t_R = 4.359 (minor) : 99:1 er. $[\alpha]_D^{21}$ = + 76.9 (c = 1.0, $CHCl_3$). HRMS m/z $[M+H]^+$ Calculated for $[C_{31}H_{20}F_3NO_4H^+]$: 528.1417; found 528.1413.

^{1}H NMR (700 MHz, $CDCl_3$) δ 10.05 (s, 1H), 7.67 – 7.64 (m, 1H), 7.54 – 7.53 (m, 1H), 7.49 (d, *J* = 1.9 Hz, 1H), 7.36 – 7.35 (m, 2H), 7.34 – 7.32 (m, 2H), 7.24 – 7.21 (m, 2H), 7.19 – 7.18 (m, 2H), 7.13 – 7.11 (m, 2H), 7.08 – 7.06 (m, 1H), 6.75 (d, *J* = 1.9 Hz, 1H), 5.29 – 5.58 (m, 1H), 4.27 – 4.27 (m, 1H), 3.46 – 3.43 (m, 1H), 3.36 – 3.33 (m, 1H).

$^{13}C\{^1H\}$ NMR (176 MHz, $CDCl_3$) δ 185.4, 163.0, 160.8, 156.4, 153.5, 143.0, 142.8, 137.8, 135.2, 130.1, 129.9 (q, *J* = 32.8 Hz), 128.9 (2C), 128.7 (2C), 128.5 (2C), 127.7, 125.9, 125.8 (q, *J* = 3.9 Hz, 2C), 125.4, 123.8 (q, *J* = 272.1 Hz), 118.1, 117.4, 113.4, 110.5, 103.0, 50.1, 49.4, 37.3.

4-((2*S*,3*R*)-3-(3-Formylfuran-2-yl)-3-phenyl-2-(4-(trifluoromethyl)phenyl)propyl)-2-oxo-2*H*-chromene-3-carbonitrile minor – 3g

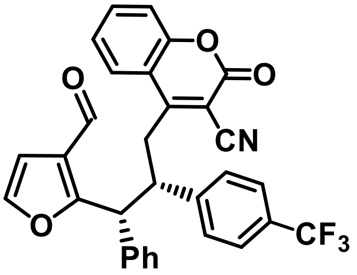

Minor diastereoisomer **3g** was isolated as light-orange oil (8.9 mg). The er was determined by UPC^2 using a chiral Chiralpack IA column gradient from 100% CO_2 up to 40%; *i*-PrOH, 2.5 mL/min; t_R = 4.067 (major), t_R = 3.942 (minor) : 99:1 er. $[\alpha]_D^{21}$ = - 99.3 (c = 1.0, $CHCl_3$). HRMS m/z $[M+H]^+$ calculated for $[C_{31}H_{20}F_3NO_4H^+]$: 528.1417; found 528.1419.

^{1}H NMR (700 MHz, $CDCl_3$) δ 9.82 (s, 1H), 7.72 – 7.69 (m, 2H), 7.65 – 7.63 (m, 1H), 7.47 – 7.45 (m, 2H), 7.40 – 7.38 (m, 2H), 7.38 – 7.37 (m, 1H), 7.31 – 7.30 (m, 1H), 7.30 – 7.29 (m, 2H), 7.25 – 7.24 (m, 2H), 7.17 (d, *J* = 2.0 Hz, 1H), 6.41 (d, *J* = 2.0 Hz, 1H), 5.31 – 5.30 (m, 1H), 4.18 – 4.14 (m, 1H), 3.33 – 3.32 (m, 1H), 3.32 – 3.31 (m, 1H).

$^{13}C\{^1H\}$ NMR (176 MHz, $CDCl_3$) δ 185.4, 163.0, 160.5, 156.2, 153.5, 142.8, 142.5, 138.0, 135.1, 130.3 (q, *J* = 32.7 Hz), 129.6 (2C), 128.9 (2C), 128.8, 128.3 (2C), 125.9, 125.8 (q, *J* = 3.7 Hz, 2C), 125.3, 123.8 (d, *J* = 272.1 Hz), 122.2, 118.1, 117.2, 113.3, 109.8, 103.2, 50.1, 49.9, 37.3.

Following the general procedure product **3h** (2.3:1 dr in a crude reaction mixture) was isolated after 3 days in 72% yield (34.1 mg) by column chromatography on silica gel (hexane : ethyl acetate 4:1 to 3:2).

4-((2*R*,3*R*)-3-(3-Formylfuran-2-yl)-3-phenyl-2-(*m*-tolyl)propyl)-2-oxo-2*H*-chromene-3-carbonitrile major – 3h

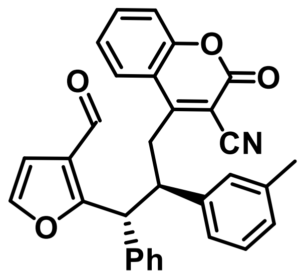

Major diasteroisomer **3h** was isolated as yellow oil (13.8 mg). The er was determined by UPC[2] using a chiral Chiralpack IB column gradient from 100% CO_2 up to 40%; *i*-PrOH, 2.5 mL/min; t_R = 4.301 (major), t_R = 4.501 (minor) : 99:1 er. $[\alpha]_D^{21}$ = + 31.5 (c = 1.0, $CHCl_3$). HRMS m/z $[M+H]^+$ calculated for $[C_{31}H_{23}NO_4+H^+]$: 474.1700; found 474.1700.

^{1}H NMR (700 MHz, $CDCl_3$) δ 10.08 (s, 1H), 7.66 – 7.63 (m, 1H), 7.61 (d, *J* = 8.2 Hz, 1H), 7.51 (d, *J* = 2.0 Hz, 1H), 7.36 (t, *J* = 7.7 Hz, 1H), 7.32 (d, *J* = 8.2 Hz, 1H), 7.24 (d, *J* = 7.7 Hz, 2H), 7.12 (t, *J* = 7.6 Hz, 2H), 7.06 (t, *J* = 7.5 Hz, 1H), 6.92 (t, *J* = 7.5 Hz, 1H), 6.89 (s, 1H), 6.84 (d, *J* = 7.5 Hz, 1H), 6.77 (d, *J* = 2.0 Hz, 1H), 6.70 (d, *J* = 7.6 Hz, 1H), 5.21 (d, *J* = 11.0 Hz, 1H), 4.10 (td, *J* = 10.3, 5.2 Hz, 1H), 3.43 (dd, *J* = 13.3, 10.3 Hz, 1H), 3.29 (dd, *J* = 13.3, 5.2 Hz, 1H), 2.18 (s, 3H).

$^{13}C\{^1H\}$ NMR (176 MHz, $CDCl_3$) δ 185.0, 163.6, 162.1, 156.6, 153.4, 142.8, 138.5, 138.4, 138.1, 134.9, 128.8, 128.7 (2C), 128.6, 128.5, 128.4 (2C), 127.4, 126.0, 125.5, 125.3, 123.6, 118.0, 117.6, 113.5, 110.1, 103.0, 50.4, 49.6, 37.7, 21.4.

4-((2*S*,3*R*)-3-(3-Formylfuran-2-yl)-3-phenyl-2-(*m*-tolyl)propyl)-2-oxo-2*H*-chromene-3-carbonitrile minor – 3h

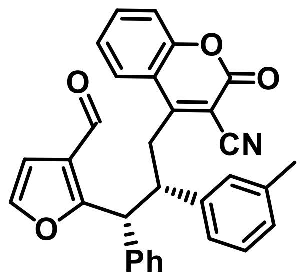

Minor diastereoisomer **3h** was isolated as light-orange oil (10.3 mg). The er was determined by UPC[2] using a chiral Chiralpack IA column gradient from 100% CO_2 up to 40%; acetonitrile, 2.5 mL/min; t_R = 3.665 (major), t_R = 4.099 (minor) : 97:3 er. $[\alpha]_D^{21}$ = - 44.5 (c = 1.0, $CHCl_3$). HRMS m/z $[M+H]^+$ calculated for $[C_{31}H_{23}NO_4+H^+]$: 474.1700; found 474.1700.

^{1}H NMR (700 MHz, $CDCl_3$) δ 9.84 (s, 1H), 7.72 – 7.70 (m, 2H), 7.64 – 7.62 (m, 1H), 7.47 – 7.45 (m, 2H), 7.40 – 7.37 (m, 2H), 7.34 – 7.32 (m, 1H), 7.30 – 7.28 (m, 1H), 7.17 (d, *J* = 2.0 Hz, 1H), 6.96 – 6.94 (m, 2H), 6.88 – 6.87 (m, 1H), 6.75 (s, 1H), 6.39 (d, *J* = 2.0 Hz, 1H), 5.15 – 5.14 (m, 1H), 4.04 – 4.01 (m, 1H), 3.32 – 3.29 (m, 1H), 3.27 – 3.25 (m, 1H), 2.20 (s, 3H).

$^{13}C\{^1H\}$ NMR (176 MHz, $CDCl_3$) δ 184.8, 163.6, 161.8, 156.5, 153.4, 142.3, 138.6, 138.4, 138.2, 134.8, 129.5 (2C), 128.8 (2C), 128.7, 126.6, 128.2, 126.1, 125.2, 124.9, 122.4, 118.0, 117.4, 113.5, 109.0, 103.2, 50.4, 50.3, 37.7 , 21.4.

Following the general procedure product **3i** (1.6:1 dr in a crude reaction mixture) was isolated after 7 days in 69% yield (32.7 mg) by column chromatography on silica gel (hexane : ethyl acetate 4:1 to 3:2).

4-((2*R*,3*R*)-3-(3-Formylfuran-2-yl)-3-phenyl-2-(*p*-tolyl)propyl)-2-oxo-2*H*-chromene-3-carbonitrile major – 3i

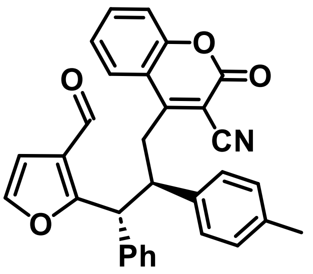

Major diasteroisomer **3i** was isolated as yellow oil (20.1 mg). The er was determined by UPC[2] using a chiral Chiralpack IB column gradient from 100% CO_2 up to 40%; *i*-PrOH, 2.5 mL/min; t_R = 4.341 (major), t_R = 4.650 (minor) : 99:1 er. $[\alpha]_D^{21}$ = + 81.2 (c = 1.0, $CHCl_3$). HRMS m/z $[M+H]^+$ calculated for $[C_{31}H_{23}NO_4+H^+]$: 474.1700; found 474.1696.

^{1}H NMR (700 MHz, $CDCl_3$) δ 10.08 (s, 1H), 7.66 – 7.64 (m, 1H), 7.60-7.59 (m, 1H), 7.50 (d, *J* = 2.0 Hz, 1H), 7.37 – 7.35 (m, 1H), 7.32 – 7.31 (m, 1H), 7.25 – 7.23 (m, 2H), 7.13 – 7.11 (m, 2H), 7.08 – 7.05 (m, 1H), 6.89 – 6.88 (m, 4H), 6.77 (d, *J* = 2.0 Hz, 1H), 5.21 – 5.19 (m, 1H), 4.14 – 4.10 (m, 1H), 3.44 – 3.40 (m, 1H), 3.30 – 3.28 (m, 1H), 2.15 (s, 3H).

$^{13}C\{^1H\}$ NMR (176 MHz, $CDCl_3$) δ 185.0, 163.6, 162.2, 156.6, 153.4, 142.8 (2C), 138.5, 137.4, 135.1, 134.9, 129.5 (2C), 128.7 (2C), 128.5 (2C), 128.0, 127.3, 126.1, 125.3, 123.6, 118.0, 117.6, 113.5, 110.1, 103.0, 50.1, 49.7, 37.9, 21.1.

4-((2*S*,3*R*)-3-(3-Formylfuran-2-yl)-3-phenyl-2-(*p*-tolyl)propyl)-2-oxo-2*H*-chromene-3-carbonitrile minor – 3i

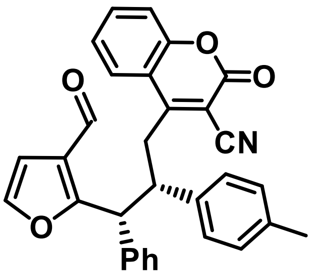

Minor diastereoisomer **3i** was isolated as light-orange oil (12.6 mg). The er was determined by UPC[2] using a chiral Chiralpack IA column gradient from 100% CO_2 up to 40%; *i*-PrOH, 2.5 mL/min; t_R = 4.125 (major), t_R = 4.593 (minor) : 97:3 er. $[\alpha]_D^{21}$ = - 96.3 (c = 1.0, $CHCl_3$). HRMS m/z $[M+H]^+$ calculated for $[C_{31}H_{23}NO_4+H^+]$: 474.1700; found 474.1699.

^{1}H NMR (700 MHz, $CDCl_3$) δ 9.85 (s, 1H), 7.72 – 7.69 (m, 2H), 7.64 – 7.62 (m, 1H), 7.47 – 7.45 (m, 2H), 7.39 – 7.37 (m, 2H), 7.34 – 7.31 (m, 1H), 7.30 – 7.28 (m, 1H), 7.17 (d, *J* = 2.0 Hz, 1H), 6.93 – 6.91 (m, 2H), 6.91 – 6.90 (m, 2H), 6.39 (d, *J* = 2.0 Hz, 1H), 5.15 – 5.13 (m, 1H), 4.06 – 4.02 (m, 1H), 3.31 – 3.27 (m, 1H), 3.26 – 7.24 (m, 1H), 2.17 (s, 3H).

$^{13}C\{^1H\}$ NMR (176 MHz, $CDCl_3$) δ 184.9, 163.7, 162.0, 156.5, 153.4, 142.3 (2C), 138.5, 137.7, 135.2, 134.8, 129.6 (2C), 129.5 (2C), 128.8 (2C), 128.6, 127.5, 126.1, 125.2, 122.4, 118.0, 117.4, 113.5, 109.0, 103.2, 50.4, 50.0, 37.8, 21.1.

Following the general procedure product **3j** (2:1 dr in a crude reaction mixture) was isolated after 5 days in 37% yield (18.1 mg) by column chromatography on silica gel (hexane : ethyl acetate 4:1 to 3:2).

4-((2*R*,3*R*)-3-(3-Formylfuran-2-yl)-2-(4-methoxyphenyl)-3-phenylpropyl)-2-oxo-2*H*-chromene-3-carbonitrile major – 3j

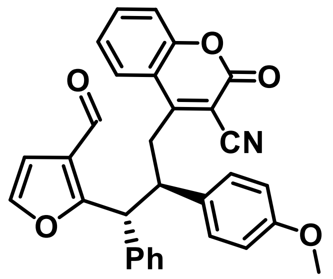

Major diasteroisomer **3j** was isolated as yellow oil (12.1 mg). The er was determined by UPC^2 using a chiral Chiralpack IB column gradient from 100% CO_2 up to 40%; *i*-PrOH, 2.5 mL/min; t_R = 4.563 (major), t_R = 4.992 (minor) : 90:10 er. $[\alpha]_D^{21}$ = + 9.1 (c = 1.0, $CHCl_3$). HRMS m/z $[M+H]^+$ calculated for $[C_{31}H_{23}NO_5+H^+]$: 490.1649; found 490.1643.

^{1}H NMR (700 MHz, $CDCl_3$) δ 10.08 (s, 1H), 7.66 – 7.64 (m, 1H), 7.61 – 7.60 (m, 1H), 7.51 (d, *J* = 2.0 Hz, 1H), 7.38 – 7.35 (m, 1H), 7.33 – 7.31 (m, 1H), 7.25 – 7.22 (m, 2H), 7.14 – 7.11 (m, 2H), 7.08 – 7.05 (m, 1H), 6.92 – 6.91 (m, 2H), 6.77 (d, *J* = 2.0 Hz, 1H), 6.62 – 6.59 (m, 2H), 5.18 – 5.17 (m, 1H), 4.12 – 4.08 (m, 1H), 3.66 (s, 3H), 3.43 – 3.39 (m, 1H), 3.29 – 3.27 (m, 1H).

^{13}C{^{1}H} NMR (176 MHz, $CDCl_3$) δ 185.1, 163.7, 162.2, 158.9, 156.6, 153.4, 142.9, 138.5, 134.9, 130.7, 130.2, 129.3, 128.7 (2C), 128.5 (2C), 127.4, 126.1, 125.3, 123.6, 118.0, 117.6, 114.2 (2C), 113.5, 110.1, 103.0, 55.2, 49.9, 49.8, 37.9.

4-((2*S*,3*R*)-3-(3-Formylfuran-2-yl)-2-(4-methoxyphenyl)-3-phenylpropyl)-2-oxo-2*H*-chromene-3-carbonitrile minor – 3j

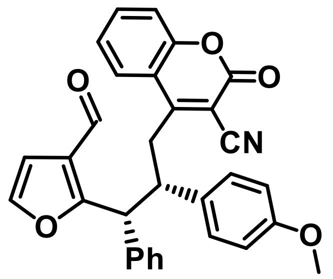

Minor diastereoisomer **3j** was isolated as light-orange oil (6.0 mg). The er was determined by UPC^2 using a chiral Chiralpack IB column gradient from 100% CO_2 up to 40%; *i*-PrOH, 2.5 mL/min; t_R = 4.519 (major), t_R = 4.665 (minor) : 97:3 er. $[\alpha]_D^{21}$ = - 16.0 (c = 1.0, $CHCl_3$). HRMS m/z $[M+H]^+$ calculated for $[C_{31}H_{23}NO_5+H^+]$: 490.1649; found 490.1647.

^{1}H NMR (700 MHz, $CDCl_3$) δ 9.84 (s, 1H), 7.71 – 7.69 (m, 2H), 7.64 – 7.62 (m, 2H), 7.47 – 7.45 (m, 3H), 7.38 – 7.36 (m, 3H), 7.33 – 7.31 (m, 1H), 7.29 – 7.28 (m, 1H), 7.17 (d, *J* = 1.9 Hz, 1H), 6.98 – 6.96 (m, 1H), 6.63 – 6.62 (m, 2H), 6.40 (d, *J* = 1.9 Hz, 1H), 3.67 (s, 3H), 3.27 – 3.26 (m, 1H), 3.26 – 3.25 (m, 1H).

^{13}C{^{1}H} NMR (176 MHz, $CDCl_3$) δ 185.1, 163.7, 162.2, 158.9, 156.6, 153.4, 142.9, 138.5, 134.9, 130.7, 130.2, 129.3, 128.7 (2C), 128.5 (2C), 127.4, 126.1, 125.3, 123.6, 118.0, 117.6, 114.2 (2C), 113.5, 110.1, 103.0, 55.2, 49.9, 49.8, 37.9.

Following the general procedure product **3k** (1.4:1 dr in a crude reaction mixture) was isolated after 7 days in 42% yield (21.4 mg) by column chromatography on silica gel (hexane : ethyl acetate 4:1 to 3:2).

4-((2*R*,3*R*)-3-(3-Formylfuran-2-yl)-2-(naphthalen-2-yl)-3-phenylpropyl)-2-oxo-2*H*-chromene-3-carbonitrile major – 3k

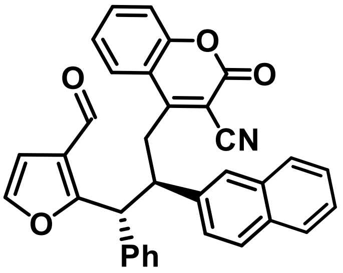

Major diasteroisomer **3k** was isolated as yellow oil (12.5 mg). The er was determined by UPC^2 using a chiral Chiralpack IB column gradient from 100% CO_2 up to 40%; *i*-PrOH, 2.5 mL/min; t_R = 4.996 (major), t_R = 5.545 (minor) : 98:2 er. $[\alpha]_D^{21}$ = + 49.6 (c = 1.0, $CHCl_3$). HRMS m/z $[M+H]^+$ calculated for $[C_{34}H_{23}NO_4+H^+]$: 510.1700; found 510.1700.

^{1}H NMR (700 MHz, $CDCl_3$) δ 10.10 (s, 1H), 7.67 – 7.64 (m, 2H), 7.64 – 7.62 (m, 1H), 7.62 – 7.60 (m, 1H), 7.60 – 7.58 (m, 1H), 7.52 (d, *J* = 1.9 Hz, 1H), 7.41 – 7.40 (m, 1H), 7.38 – 7.37 (m, 1H), 7.37 – 7.36 (m, 1H), 7.33 – 7.30 (m, 1H), 7.28 – 7.27 (m, 1H), 7.27 – 7.26 (m, 2H), 7.26 – 7.25 (m, 1H), 7.07 – 7.05 (m, 2H), 7.00 – 6.97 (m, 1H), 6.77 (d, *J* = 1.9 Hz, 1H), 5.36 – 5.35 (m, 1H), 4.38 – 4.34 (m, 1H), 3.57 – 3.54 (m, 1H), 3.41 – 3.38 (m, 1H).

$^{13}C\{^1H\}$ NMR (176 MHz, $CDCl_3$) δ 185.2, 163.5, 161.8, 156.5, 153.4, 142.9, 138.3, 135.9, 134.9, 133.1, 132.7, 128.8, 128.7 (2C), 128.5 (2C), 127.8, 127.7, 127.6, 127.4, 126.4, 126.1, 126.0, 125.5, 125.3, 123.6, 118.0, 117.6, 113.5, 110.3, 102.9, 50.5, 49.7, 38.7.

4-((2*S*,3*R*)-3-(3-Formylfuran-2-yl)-2-(naphthalen-2-yl)-3-phenylpropyl)-2-oxo-2*H*-chromene-3-carbonitrile minor – 3k

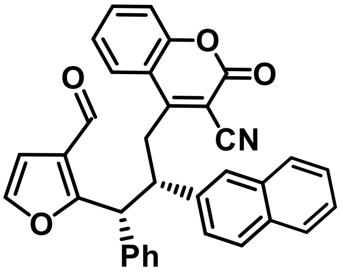

Minor diastereoisomer **3k** was isolated as light-orange oil (8.9 mg). The er was determined by UPC^2 using a chiral Chiralpack IA column gradient from 100% CO_2 up to 40%; *i*-PrOH, 2.5 mL/min; t_R = 4.705 (major), t_R = 5.088 (minor) : 97:3 er. $[\alpha]_D^{21}$ = - 81.0 (c = 1.0, $CHCl_3$). HRMS m/z $[M+H]^+$ calculated for $[C_{34}H_{23}NO_4+H^+]$: 510.1700; found 510.1694.

^{1}H NMR (700 MHz, $CDCl_3$) δ 9.82 (s, 1H), 7.77 – 7.75 (m, 2H), 7.69 – 7.67 (m, 1H), 7.66 – 7.64 (m, 1H), 7.64 – 7.62 (m, 1H), 7.6 – 7.59 (m, 1H), 7.49 – 7.46 (m, 2H), 7.44 – 7.42 (m, 1H), 7.39 – 7.38 (m, 2H), 7.38 – 7.37 (m, 2H), 7.32 – 7.29 (m, 2H), 7.25 – 7.24 (m, 1H), 7.13 (d, *J* = 1.9 Hz, 1H), 6.28 (d, *J* = 1.9 Hz, 1H), 5.35 – 5.33 (m, 1H), 4.31 – 4.27 (m, 1H), 3.45 – 3.41 (m, 1H), 3.38 – 3.35 (m, 1H).

$^{13}C\{^1H\}$ NMR (176 MHz, $CDCl_3$) δ 185.0, 163.6, 161.5, 156.4, 153.4, 142.4, 138.4, 135.9, 134.9, 133.2, 132.9, 129.5 (C), 128.9 (2C), 128.7 (2C), 127.8, 127.7, 127.1, 126.5, 126.2, 126.0, 125.2, 125.0, 122.3, 118.0, 117.4, 113.4, 109.3, 103.2, 50.4, 50.5, 37.6.

Following the general procedure product **3l** (1:1 dr in a crude reaction mixture) was isolated after 7 days in 56% yield (30.1 mg) by column chromatography on silica gel (hexane : ethyl acetate 4:1 to 3:2).

6-Bromo-4-((2*R*,3*R*)-3-(3-formylfuran-2-yl)-2,3-diphenylpropyl)-2-oxo-2*H*-chromene-3-carbonitrile major – 3l

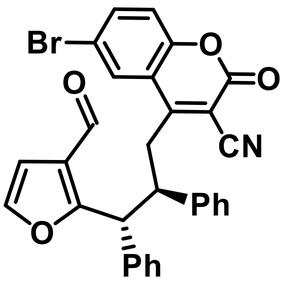

Major diasteroisomer **3l** was isolated as yellow oil (15.2 mg) The er was determined by UPC[2] using a chiral Chiralpack IB column gradient from 100% CO_2 up to 40%; *i*-PrOH, 2.5 mL/min; t_R = 5.099 (major), t_R = 5.400 (minor) : 99:1 er. $[\alpha]_D^{21}$ = + 12.2 (c = 1.0, $CHCl_3$). HRMS m/z $[M+H]^+$ calculated for $[C_{30}H_{20}BrNO_4+H^+]$: 538.0649; found 538.0647.

^{1}H NMR (700 MHz, $CDCl_3$) δ 10.12 (s, 1H), 7.74 – 7.73 (m, 1H), 7.72 – 7.71 (m, 1H), 7.61 (d, *J* = 2.0 Hz, 1H), 7.26 – 7.24 (m, 2H), 7.21 – 7.20 (m, 1H), 7.13 – 7.12 (m, 2H), 7.11 – 7.10 (m, 2H), 7.07 – 7.04 (m, 2H), 7.02 – 7.01 (m, 2H), 6.80 (d, *J* = 2.0 Hz, 1H), 5.29 – 5.27 (m, 1H), 4.07 – 4.04 (m, 1H), 3.41 – 3.38 (m, 1H), 3.23 – 3.20 (m, 1H).

^{13}C{^{1}H} NMR (176 MHz, $CDCl_3$) δ 185.2, 162.3, 161.8, 155.8, 152.2, 143.2, 138.1, 137.8, 137.6, 128.9 (2C), 128.8 (2C), 128.7, 128.5 (2C), 128.4 (2C), 128.1, 127.5, 123.7, 119.6, 119.0, 118.3, 112.8, 110.2, 103.9, 50.6, 49.4, 37.9.

6-Bromo-4-((2*S*,3*R*)-3-(3-formylfuran-2-yl)-2,3-diphenylpropyl)-2-oxo-2*H*-chromene-3-carbonitrile minor – 3l

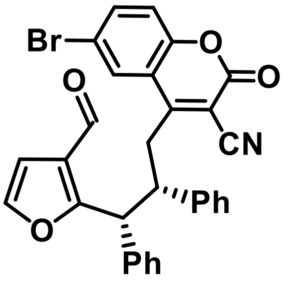

Minor diastereoisomer **3l** was isolated as light-orange oil (14.9 mg). The er was determined by UPC[2] using a chiral Chiralpack IB column gradient from 100% CO_2 up to 40%; *i*-PrOH, 2.5 mL/min; t_R = 5.379 (major), t_R = 5.176 (minor) : 98:2 er. $[\alpha]_D^{21}$ = - 26.9 (c = 1.0, $CHCl_3$). HRMS m/z $[M+H]^+$ calculated for $[C_{30}H_{20}BrNO_4+H^+]$: 538.0649; found 538.0648.

^{1}H NMR (700 MHz, $CDCl_3$) δ 9.85 (s, 1H), 8.10 – 8.09 (m, 1H), 7.74 – 7.43 (m, 2H), 7.71 – 7.69 (m, 1H), 7.53 – 7.71 (m, 2H), 7.51 – 7.48 (m, 2H), 7.41 – 7.39 (m, 1H), 7.19 – 7.17 (m, 1H), 7.15 – 7.13 (m, 1H), 7.11 – 7.10 (m, 1H), 6.40 – 6.39 (m, 1H), 5.41 – 5.32 (m, 1H), 5.24 – 5.22 (m, 1H), 4.70 – 4.69 (m, 1H), 4.01 – 3.97 (m, 1H), 3.28 – 3.24 (m, 1H), 3.22 – 3.19 (m, 1H).

^{13}C{^{1}H} NMR (176 MHz, $CDCl_3$) δ 185.1, 165.4, 162.4, 161.3, 155.7, 152.2, 142.5, 138.3, 138.0, 137.5, 133.9, 130.0, 129.9 (2C), 129.0, 128.7 (2C), 128.6, 128.3, 127.8, 122.4, 119.6, 118.8, 118.3, 112.9, 109.2, 104.1, 50.6, 50.0, 37.6.

Following the general procedure product **3m** (2:1 dr in a crude reaction mixture) was isolated after 5 days in 41% yield (20.1 mg) by column chromatography on silica gel (hexane : ethyl acetate 4:1 to 3:2).

4-((2*R*,3*R*)-3-(3-Formylfuran-2-yl)-2,3-diphenylpropyl)-7-methoxy-2-oxo-2*H*-chromene-3-carbonitrile major – 3m

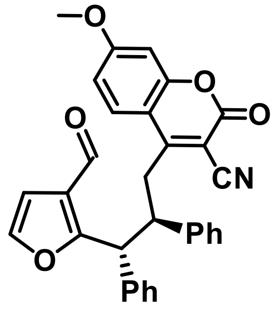

Major diasteroisomer **3m** was isolated as yellow oil (13.4 mg). The er was determined by UPC^2 using a chiral Chiralpack IB column gradient from 100% CO_2 up to 40%; *i*-PrOH, 2.5 mL/min; t_R = 5.001 (major), t_R = 5.308 (minor) : 99:1 er. $[\alpha]_D^{21}$ = + 12.7 (c = 1.0, $CHCl_3$). HRMS m/z $[M+H]^+$ calculated for $[C_{31}H_{23}NO_5+H^+]$: 490.1649; found 490.1648.

^{1}H NMR (700 MHz, $CDCl_3$) δ 10.09 (s, 1H), 7.52 – 7.50 (m, 1H), 7.47 – 7.46 (m, 1H), 7.25 – 7.24 (m, 2H), 7.12 – 7.10 (m, 2H), 7.08 – 7.06 (m, 2H), 7.05 – 7.04 (m, 2H), 7.02 – 7.01 (m, 2H), 6.89 – 6.87 (m, 1H), 6.76 – 6.74 (m, 2H), 5.23 – 5.21 (m, 1H), 4.15 – 4.11 (m, 1H), 3.90 (s, 3H), 3.39 – 3.36 (m, 1H), 3.25 – 3.22 (m, 1H).

^{13}C{^{1}H} NMR (176 MHz, $CDCl_3$) δ 185.1, 165.2, 163.4, 162.0, 157.3, 155.8, 142.9, 138.4, 138.3, 128.8 (2C), 128.7 (2C), 128.6 (2C), 128.3 (2C), 127.8, 127.3 (2C), 123.6, 114.0, 113.9, 111.3, 110.1, 101.3, 99.1, 56.3, 50.5, 49.6, 37.7.

4-((2*S*,3*R*)-3-(3-Formylfuran-2-yl)-2,3-diphenylpropyl)-7-methoxy-2-oxo-2*H*-chromene-3-carbonitrile minor – 3m

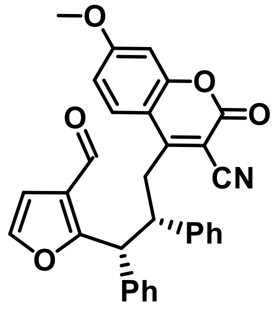

Minor diastereoisomer **3m** was isolated as light-orange oil (6.7 mg). The er was determined by UPC^2 using a chiral Chiralpack IA column gradient from 100% CO_2 up to 40%; *i*-PrOH, 2.5 mL/min; t_R = 4.312 (major), t_R = 4.762 (minor) : 99:1 er. $[\alpha]_D^{21}$ = - 25.5 (c = 1.0, $CHCl_3$). HRMS m/z $[M+H]^+$ calculated for $[C_{31}H_{23}NO_5+H^+]$: 490.1649; found 490.1647.

^{1}H NMR (700 MHz, $CDCl_3$) δ 9.84 (s, 1H), 7.72 – 7.70 (m, 2H), 7.48 – 7.44 (m, 2H), 7.39 – 7.37 (m, 1H), 7.25 – 7.22 (m, 1H), 7.16 (d, *J* = 1.9 Hz, 1H), 7.13 – 7.12 (m, 2H), 7.11 – 7.09 (m, 1H), 7.09 – 7.05 (m, 2H), 6.87 – 6.83 (m, 1H), 6.72 – 6.71 (m, 1H), 6.38 (d, *J* = 1.9 Hz, 1H), 5.18 – 5.15 (m, 1H), 4.11 – 4.01 (m, 1H), 3.90 (s, 3H), 3.24 – 3.23 (s, 1H), 3.22 – 3.19 (m, 1H).

^{13}C{^{1}H} NMR (176 MHz, $CDCl_3$) δ 185.2, 165.2, 163.4, 162.0, 157.3, 155.8, 142.9, 138.4, 138.3 128.7 (2C), 128.7 (2C), 128.6 (2C), 128.3 (2C), 127.8, 127.3 (2C), 123.6, 114.0, 113.9, 111.3, 110.1, 101.3, 99.0, 56.3, 50.4, 49.6, 37.7.

Following the general procedure product **3n** (1.5:1 dr in a crude reaction mixture) was isolated after 3 days in 78% yield (34.3 mg) by column chromatography on silica gel (hexane : ethyl acetate 4:1 to 3:2).

4-((*R*)-2-((*R*)-(3-formylfuran-2-yl)(phenyl)methyl)hexyl)-2-oxo-*2H*-chromene-3-carbonitrile major – 3n

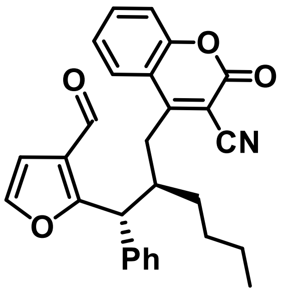

Major diasteroisomer **3m** was isolated as yellow oil (20.6 mg). The er was determined by UPC[2] using a chiral Chiralpack IB column gradient from 100% CO_2 up to 40%; *i*-PrOH, 2.5 mL/min; t_R = 3.941 (major), t_R = 4.107 (minor) : 99:1 er. $[\alpha]_D^{21}$ = + 66.3 (c = 1.0, $CHCl_3$). HRMS m/z $[M+H]^+$ calculated for $[C_{28}H_{25}NO_4+H^+]$: 440.1857; found 440.1853.

^{1}H NMR (700 MHz, $CDCl_3$) δ 10.04 (s, 1H), 7.67 – 7.64 (m, 1H), 7.42 – 7.40 (m, 2H), 7.40 – 7.39 (m, 1H), 7.38 (d, *J* = 2.0 Hz, 1H), 7.34 – 7.31 (m, 2H), 7.26 – 7.24 (m, 2H), 7.24 – 7.22 (m, 1H), 6.69 (d, *J* = 2.0 Hz, 1H), 4.65 – 4.63 (m, 1H), 3.13 – 3.07 (m, 2H), 3.04 – 3.01 (m, 1H), 1.38 – 1.30 (m, 2H), 1.25 – 1.20 (m, 2H), 1.17 – 1.10 (m, 2H), 0.77 – 0.75 (m, 3H).

^{13}C{^{1}H} NMR (176 MHz, $CDCl_3$) δ 184.7, 165.0, 162.9, 156.5, 153.5, 142.6, 138.6, 135.0, 129.0 (2C), 128.8 (2C), 128.4, 126.1, 125.2, 122.8, 118.0, 117.7, 113.8, 109.2, 103.2, 48.9, 42.7, 35.7, 32.1, 27.9, 22.9, 13.8.

4-((*S*)-2-((*R*)-(3-formylfuran-2-yl)(phenyl)methyl)hexyl)-2-oxo-2*H*-chromene-3-carbonitrile minor – 3n

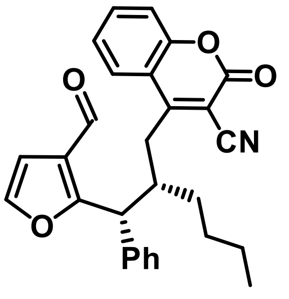

Minor diastereoisomer **3n** was isolated as yellow oil (13.7 mg). The er was determined by UPC[2] using a chiral Chiralpack IA column gradient from 100% CO_2 up to 40%; *i*-PrOH, 2.5 mL/min; t_R = 3.657 (major), t_R = 3.769 (minor) : 99:1 er. $[\alpha]_D^{21}$ = - 41.3 (c = 1.0, $CHCl_3$). HRMS m/z $[M+H]^+$ calculated for $[C_{28}H_{25}NO_4+H^+]$: 440.1857; found 440.1854.

^{1}H NMR (700 MHz, $CDCl_3$) δ 9.84 (s, 1H), 7.59 – 7.55 (m, 1H), 7.31 – 7.28 (m, 2H), 7.28 – 7.26 (m, 2H) 7.25 – 7.23 (m, 2H), 7.22 – 7.20 (m, 2H), 7.19 – 7.18 (m, 1H), 6.56 – 6.55 (m, 1H), 4.76 – 4.74 (m, 1H), 3.25 – 3.22 (m, 1H), 3.07 – 3.04 (m 1H), 2.93 – 2.86 (m, 1H), 1.32 – 1.24 (m, 2H), 1.23 – 1.15 (m, 2H), 1.11 – 1.03 (m, 2H), 0.69 – 0.66 (m, 3H).

^{13}C{^{1}H} NMR (176 MHz, $CDCl_3$) δ 184.6, 164.8, 162.3, 156.7, 153.5, 142.3, 139.3, 135.0, 129.1 (2C), 128.4 (2C), 127.7, 126.0, 125.4, 123.5, 118.0, 117.8, 113.8, 110.0, 102.8, 47.5, 43.6, 35.4, 31.9, 28.3, 22.7, 13.9.

Following the general procedure product **3o** (1.8:1 dr in a crude reaction mixture) was isolated after 3 days in 63% yield (33.2 mg) by column chromatography on silica gel (hexane : ethyl acetate 4:1 to 3:2).

4-((2*R*,3*R*)-3-(3-Formylfuran-2-yl)-2-phenyl-3-(3-(trifluoromethyl)phenyl)propyl)-2-oxo-2*H*-chromene-3-carbonitrile major – 3o

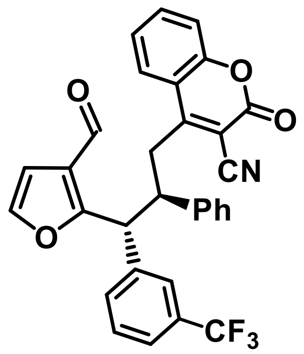

Major diasteroisomer **3o** was isolated as yellow oil (21.3 mg). The er was determined by UPC^2 using a chiral Chiralpack IB column gradient from 100% CO_2 up to 40%; *i*-PrOH, 2.5 mL/min; t_R = 3.936 (major), t_R = 4.172 (minor) : 99:1 er. $[\alpha]_D^{21}$ = + 51.5 (c = 1.0, $CHCl_3$). HRMS m/z $[M+H]^+$ calculated for $[C_{31}H_{20}F_3NO_4+H^+]$: 528.1417; found 528.1409.

^{1}H NMR (700 MHz, CDCl$_3$) δ 10.07 (s, 1H), 7.67 – 7.63 (m, 1H), 7.60 – 7.57 (m, 1H), 7.52 (d, *J* = 2.0 Hz, 1H), 7.45 (s, 1H), 7.42 (d, *J* = 7.9 Hz, 1H), 7.37 (t, *J* = 7.6 Hz, 1H), 7.32 (d, *J* = 8.3 Hz, 1H), 7.30 (d, *J* = 7.8 Hz, 1H), 7.22 (t, *J* = 7.8 Hz, 1H), 7.09 (t, *J* = 7.4 Hz, 2H), 7.04 (d, *J* = 7.2 Hz, 1H), 7.02 (d, *J* = 8.1 Hz, 2H), 6.79 (d, *J* = 2.0 Hz, 1H), 5.37 (d, *J* = 11.4 Hz, 1H), 4.18 – 4.09 (m, 1H), 3.46 (dd, *J* = 13.4, 9.8 Hz, 1H), 3.31 (dd, *J* = 13.4, 5.4 Hz, 1H).

^{13}C{^{1}H} NMR (176 MHz, CDCl$_3$) δ 185.5, 163.2, 160.2, 156.5, 153.4, 143.2, 139.4, 137.9, 135.1, 132.1, 130.8 (q, *J* = 32.4 Hz), 129.1, 129.0 (2C), 128.2 (2C), 128.1, 126.0, 125.4, 125.3 (q, *J* = 3.9 Hz), 124.2 (q, *J* = 3.9 Hz), 123.8 (q, *J* = 273.5 Hz), 123.7, 118.0, 117.5, 113.4, 110.7, 102.9, 50.3, 49.4, 37.6.

4-((2*S*,3*R*)-3-(3-Formylfuran-2-yl)-2-phenyl-3-(3-(trifluoromethyl)phenyl)propyl)-2-oxo-2*H*-chromene-3-carbonitrile minor – 3o

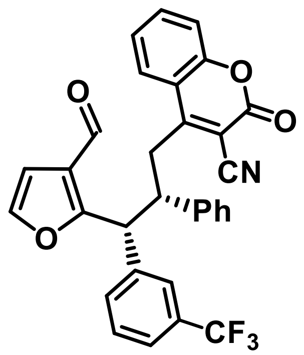

Minor diastereoisomer **3o** was isolated as light-orange oil (11.9 mg). The er was determined by UPC^2 using a chiral Chiralpack IB column gradient from 100% CO_2 up to 40%; *i*-PrOH, 2.5 mL/min; t_R = 4.417 (major), t_R = 3.947 (minor) : 99:1 er. $[\alpha]_D^{21}$ = - 75.9 (c = 1.0, $CHCl_3$). HRMS m/z $[M+H]^+$ calculated for $[C_{31}H_{20}F_3NO_4+H^+]$: 528.1417; found 528.1415.

^{1}H NMR (700 MHz, CDCl$_3$) δ 9.81 (s, 1H), 8.00 (s, 1H), 7.95 (d, *J* = 7.5 Hz, 1H), 7.67 – 7.61 (m, 3H), 7.32 (d, *J* = 4.4 Hz, 2H), 7.30 (d, *J* = 8.3 Hz, 1H), 7.19 (d, *J* = 1.9 Hz, 1H), 7.12 (d, *J* = 7.2 Hz, 2H), 7.09 (dd, *J* = 15.1, 8.1 Hz, 3H), 6.40 (d, *J* = 2.0 Hz, 1H), 5.37 (d, *J* = 11.7 Hz, 1H), 4.08 (td, *J* = 11.3, 4.4 Hz, 1H), 3.33 (dd, *J* = 13.3, 11.0 Hz, 1H), 3.20 (dd, *J* = 13.3, 4.5 Hz, 1H).

^{13}C{^{1}H} NMR (176 MHz, CDCl$_3$) δ 185.3, 163.1, 159.9, 156.3, 153.4, 142.7, 139.5, 137.9, 135.1, 132.9, 131.9 (q, *J* = 32.6 Hz), 130.2, 129.0 (2C), 128.3, 127.7 (2C), 125.7, 125.4 (q, *J* = 3.4 Hz), 125.3, 125.0 (q, *J* = 3.6 Hz), 124.0 (q, *J* = 272.7 Hz), 122.4, 118.1, 117.2, 113.3, 109.6, 103.3, 50.3, 49.8, 37.5.

Following the general procedure product **3p** (1.5:1 dr in a crude reaction mixture) was isolated after 3 days in 73% yield (34.8 mg) by column chromatography on silica gel (hexane : ethyl acetate 4:1 to 3:2).

4-((2*R*,3*R*)-3-(4-Fluorophenyl)-3-(3-formylfuran-2-yl)-2-phenylpropyl)-2-oxo-2*H*-chromene-3-carbonitrile major – 3p

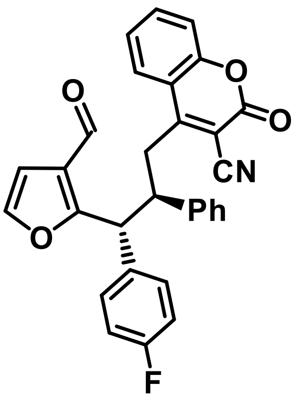

Major diasteroisomer **3p** was isolated as yellow oil (20.9 mg). The er was determined by UPC^2 using a chiral Chiralpack IB column gradient from 100% CO_2 up to 40%; *i*-PrOH, 2.5 mL/min; t_R = 4.334 (major), t_R = 4.609 (minor) : >99:1 er. $[\alpha]_D^{21}$ = + 33.5 (c = 1.0, $CHCl_3$). HRMS m/z $[M+H]^+$ calculated for $[C_{30}H_{20}FNO_4+H^+]$: 478.1449; found 478.1448.

^{1}H NMR (700 MHz, $CDCl_3$) δ 10.07 (s, 1H), 7.64 (t, *J* = 7.6 Hz, 1H), 7.59 (d, *J* = 7.6 Hz, 1H), 7.51 – 7.50 (m, 1H), 7.35 (t, *J* = 7.6 Hz, 1H), 7.32 – 7.31 (m, 1H), 7.21 – 7.19 (m, 2H), 7.11 – 7.09 (m, 2H), 7.09 – 7.05 (m, 1H), 7.04 – 7.01 (m, 2H), 6.80 – 6.77 (m, 3H), 5.28 – 5.26 (m, 1H), 4.13 – 4.10 (m, 1H), 3.44 – 3.41 (m, 1H), 3.30 – 3.27 (m, 1H).

^{13}C{^{1}H} NMR (176 MHz, $CDCl_3$) δ 185.4, 163.4, 161.8 (d, *J* = 246.1 Hz), 161.3, 156.5, 153.4, 142.9, 138.2, 135.0, 135.0, 134.2, (d, *J* = 2.6 Hz), 130.1 (d, *J* = 7.9 Hz, 2C), 128.9, 128.2 (2C), 127.9, 126.0, 125.3, 123.4, 118.0, 117.5, 115.6 (d, *J* = 21.3 Hz, 2C), 113.4, 110.5, 102.9, 50.4, 48.8, 37.7.

4-((2*S*,3*R*)-3-(4-Fluorophenyl)-3-(3-formylfuran-2-yl)-2-phenylpropyl)-2-oxo-2*H*-chromene-3-carbonitrile minor – 3p

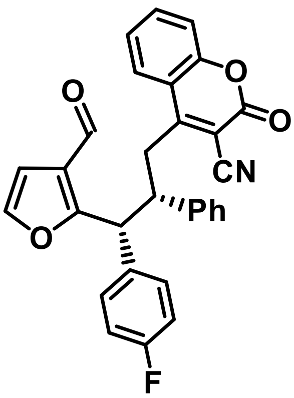

Minor diastereoisomer **3p** was isolated as light-orange oil (13.9 mg). The er was determined by UPC^2 using a chiral Chiralpack IB column gradient from 100% CO_2 up to 40%; *i*-PrOH, 2.5 mL/min; t_R = 4.904 (major), t_R = 4.393 (minor) : 98:2 er. $[\alpha]_D^{21}$ = - 54.5 (c = 1.0, $CHCl_3$). HRMS m/z $[M+H]^+$ calculated for $[C_{30}H_{20}FNO_4+H^+]$: 478.1449; found 478.1449.

^{1}H NMR (700 MHz, $CDCl_3$) δ 9.81 (s, 1H), 7.73 – 7.69 (m, 2H), 7.64 – 7.61 (m, 1H), 7.36 – 7.35 (m, 1H), 7.32 – 7.28 (m, 3H), 7.17 – 7.14 (m, 3H), 7.11 – 7.07 (m, 2H), 7.07 – 7.02 (m, 2H), 6.39 (d, *J* = 2.0 Hz, 1H), 5.25 (d, *J* = 11.4 Hz, 1H), 4.05 (td, *J* = 11.4, 4.9 Hz, 1H), 3.31 – 3.28 (m, 1H), 3.27 – 3.24 (m, 1H).

^{13}C{^{1}H} NMR (176 MHz, $CDCl_3$) δ 185.2, 163.6, 162.7 (d, *J* = 248.0 Hz), 161.1, 156.4, 153.4, 142.4, 138.2, 135.0, 134.1 (d, *J* = 3.2 Hz), 130.5 (d, *J* = 8.1 Hz, 2C), 128.9 (2C), 128.1, 127.7 (2C), 125.9, 125.2, 122.2, 118.0, 117.4, 116.5 (d, *J* = 21.4 Hz, 2C), 113.4, 109.4, 103.2, 50.4, 49.4, 37.5.

Following the general procedure product **3q** (2:1 dr in a crude reaction mixture) was isolated after 5 days in 81% yield (38.4 mg) by column chromatography on silica gel (hexane : ethyl acetate 4:1 to 3:2).

4-((2*R*,3*R*)-3-(3-Formylfuran-2-yl)-2-phenyl-3-(*p*-tolyl)propyl)-2-oxo-2*H*-chromene-3-carbonitrile major – 3q

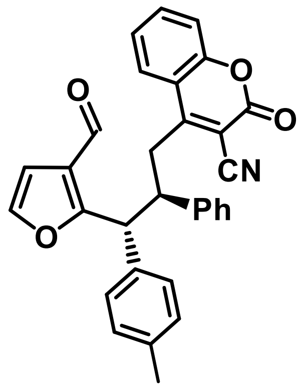

Major diasteroisomer **3q** was isolated as yellow oil (25.6 mg). The er was determined by UPC^2 using a chiral Chiralpack IB column gradient from 100% CO_2 up to 40%; *i*-PrOH, 2.5 mL/min; t_R = 4.485 (major), t_R = 4.767 (minor) : >99:1 er. $[\alpha]_D^{21}$ = + 64.3 (c = 1.0, $CHCl_3$). HRMS m/z $[M+H]^+$ calculated for $[C_{31}H_{23}NO_4+H^+]$: 474.1700; found 474.1692.

^{1}H NMR (700 MHz, $CDCl_3$) δ 10.10 (s, 1H), 7.65 (t, *J* = 7.8 Hz, 1H), 7.61 (d, *J* = 7.8 Hz, 1H), 7.50 (d, *J* = 1.8 Hz, 1H), 7.37 (t, *J* = 7.8 Hz, 1H), 7.31 (d, *J* = 8.3 Hz, 1H), 7.14 (d, *J* = 7.8 Hz, 2H), 7.10 – 7.08 (m, 2H), 7.04 – 7.03 (m, 2H), 7.03 – 7.02 (m, 1H), 6.91 (d, *J* = 7.8 Hz, 2H), 6.76 (d, *J* = 1.8 Hz, 1H), 5.22 (d, *J* = 11.0 Hz, 1H), 4.15 (td, *J* = 10.3, 5.0 Hz, 1H), 3.43 (dd, *J* = 13.3, 10.3 Hz, 1H), 3.31 (dd, *J* = 13.3, 5.0 Hz, 1H), 2.15 (s, 3H).

$^{13}C\{^1H\}$ NMR (176 MHz, $CDCl_3$) δ 185.1, 163.6, 162.4, 156.5, 153.3, 142.8, 138.4, 137.0, 135.2, 135.0, 129.4 (2C), 128.7 (2C), 128.3 (2C), 128.2 (2C), 127.7, 126.0, 125.3, 123.5, 117.9, 117.5, 113.4, 110.0, 102.8, 50.3, 49.0, 37.8, 21.01.

4-((2*S*,3*R*)-3-(3-Formylfuran-2-yl)-2-phenyl-3-(*p*-tolyl)propyl)-2-oxo-2*H*-chromene-3-carbonitrile minor – 3q

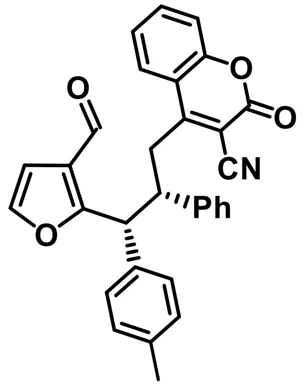

Minor diastereoisomer **3q** was isolated as light-orange oil (12.8 mg). The er was determined by UPC^2 using a chiral Chiralpack IB column gradient from 100% CO_2 up to 40%; *i*-PrOH, 2.5 mL/min; t_R = 4.845 (major), t_R = 4.573 (minor) : >99:1 er. $[\alpha]_D^{21}$ = - 94.5 (c = 1.0, $CHCl_3$). HRMS m/z $[M+H]^+$ calculated for $[C_{31}H_{23}NO_4+H^+]$: 474.1700; found 474.1694.

^{1}H NMR (700 MHz, $CDCl_3$) δ 9.84 (s, 1H), 7.64 (t, *J* = 7.7 Hz, 1H), 7.57 (d, *J* = 7.7 Hz, 2H), 7.43 (d, *J* = 7.7 Hz, 1H), 7.35 – 7.33 (m, 1H), 7.29 – 7.28 (m, 1H), 7.25 – 7.24 (m, 2H), 7.15 (d, *J* = 1.9 Hz, 1H), 7.12 – 7.10 (m, 2H), 7.09 – 7.07 (m, 2H), 7.07 – 7.06 (m, 1H), 6.37 (d, *J* = 1.9 Hz, 1H), 5.14 (d, *J* = 11.6 Hz, 1H), 4.09 – 4.06 (m, 1H), 3.34 – 3.32 (m, 1H), 3.30 – 3.28 (m, 1H), 2.37 (s, 3H).

$^{13}C\{^1H\}$ NMR (176 MHz, $CDCl_3$) δ 184.9, 163.7, 162.1, 156.4, 153.4, 142.3, 138.6, 138.5, 135.2, 134.9, 130.1 (2C), 128.8 (2C), 128.7 (2C), 128.0, 127.6 (2C), 126.1, 125.2, 122.2, 117.9, 117.4, 113.4, 108.9, 103.1, 50.4, 49.9, 37.7, 21.2.

Following the general procedure product **3r** (2.3:1 dr in a crude reaction mixture) was isolated after 3 days in 72% yield (35.2 mg) by column chromatography on silica gel (hexane : ethyl acetate 4:1 to 3:2).

4-((2*R*,3*R*)-3-(3-Formylfuran-2-yl)-3-(3-methoxyphenyl)-2-phenylpropyl)-2-oxo-2*H*-chromene-3-carbonitrile major – 3r

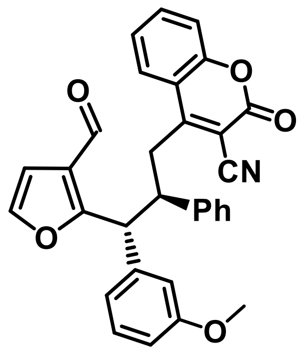

Major diasteroisomer **3r** was isolated as yellow oil (24.5 mg). The er was determined by UPC[2] using a chiral Chiralpack IA column gradient from 100% CO_2 up to 40%; *i*-PrOH, 2.5 mL/min; t_R = 4.398 (major), t_R = 4.137 (minor) : >99:1 er. $[\alpha]_D^{21}$ = + 12.9 (c = 1.0, $CHCl_3$). HRMS m/z $[M+H]^+$ calculated for $[C_{31}H_{23}NO_5+H^+]$: 490.1649; found 490.1647.

^{1}H NMR (700 MHz, $CDCl_3$) δ 10.08 (s, 1H), 7.66 – 7.63 (m, 1H), 7.60 (d, *J* = 7.8 Hz, 1H), 7.50 (d, *J* = 2.0 Hz, 1H), 7.36 (t, *J* = 7.8 Hz, 1H), 7.32 – 7.31 (m, 1H), 7.10 (t, *J* = 7.8 Hz, 2H), 7.06 – 7.05 (m, 1H), 7.04 – 7.03 (m, 1H), 7.03 – 7.00 (m, 2H), 6.83 – 6.81 (m, 1H), 6.79 – 6.67 (m, 1H), 6.77 (d, *J* = 2.0 Hz, 1H), 6.60 – 6.57 (m, 1H), 5.21 (d, *J* = 11.1 Hz, 1H), 4.13 (td, *J* = 10.3, 5.2 Hz, 1H), 3.66 (s, 3H), 3.43 (dd, *J* = 13.3, 10.3 Hz, 1H), 3.30 (dd, *J* = 13.3, 5.2 Hz, 1H).

^{13}C{^{1}H} NMR (176 MHz, $CDCl_3$) δ 185.1, 163.5, 161.8, 159.6, 156.5, 153.4, 142.9, 139.8, 138.3, 135.0, 129.7, 128.8 (2C), 128.2 (2C), 127.9, 126.0, 125.3, 123.7, 121.0, 118.0, 117.5, 114.7, 113.4, 112.4, 110.2, 102.9, 55.3, 50.3, 49.5, 37.8.

4-((2*S*,3*R*)-3-(3-Formylfuran-2-yl)-3-(3-methoxyphenyl)-2-phenylpropyl)-2-oxo-2*H*-chromene-3-carbonitrile minor – 3r

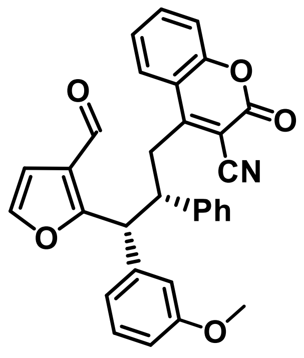

Minor diastereoisomer **3r** was isolated as light-orange oil (10.7 mg). The er was determined by UPC[2] using a chiral Chiralpack IA column gradient from 100% CO_2 up to 40%; *i*-PrOH, 2.5 mL/min; t_R = 4.231 (major), t_R = 4.404 (minor) : 98:2 er. $[\alpha]_D^{21}$ = - 37.1 (c = 1.0, $CHCl_3$). HRMS m/z $[M+H]^+$ calculated for $[C_{31}H_{23}NO_5+H^+]$: 490.1649; found 490.1648.

^{1}H NMR (700 MHz, $CDCl_3$) δ 9.84 (s, 1H), 7.73 – 7.69 (m, 2H), 7.48 – 7.43 (m, 2H), 7.40 – 7.35 (m, 1H), 7.25 – 7.21 (m, 1H), 7.17 – 7.16 (m, 1H), 7.14 – 7.10 (m, 2H), 7.10 – 7.08 (m, 2H), 7.07 – 7.04 (m, 1H), 6.87 – 6.83 (m, 1H), 6.72 – 6.71 (m, 1H), 6.40 – 6.37 (m, 1H), 5.18 – 5.15 (m, 1H), 4.10 – 4.02 (m, 1H), 3.90 (s, 3H), 3.29 – 3.24 (s, 1H), 3.23 – 3.17 (m, 1H).

^{13}C{^{1}H} NMR (176 MHz, $CDCl_3$) δ 184.9, 163.5, 162.0, 158.7, 156.4, 153.3, 142.7, 138.3, 134.8, 130.5, 130.0, 129.1 (2C), 128.5 (2C), 128.4 (2C), 127.2, 125.9, 125.1, 123.4, 117.8, 117.4, 114.0, 113.3, 109.9, 102.8, 55.1, 49.7, 49.6, 37.0.

Following the general procedure product **3s** (1.2:1 dr in a crude reaction mixture) was isolated after 3 days in 71% yield (32.6 mg) by column chromatography on silica gel (hexane : ethyl acetate 4:1 to 3:2).

4-((2*R*,3*R*)-3-(5-Formylfuran-2-yl)-2,3-diphenylpropyl)-2-oxo-2*H*-chromene-3-carbonitrile major – 3s

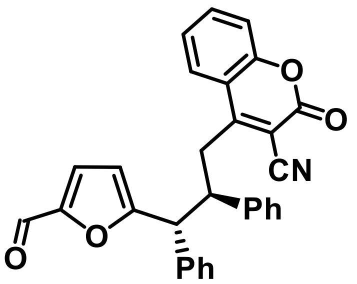

Major diasteroisomer **3s** was isolated as yellow oil (17.8 mg). The er was determined by UPC^2 using a chiral Chiralpack IA column gradient from 100% CO_2 up to 40%; acetonitrile, 2.5 mL/min; t_R = 4.307 (major), t_R = 4.033 (minor) : 63:37 er. $[\alpha]_D^{21}$ = + 59.3 (c = 1.0, $CHCl_3$). HRMS m/z $[M+H]^+$ calculated for $[C_{30}H_{21}NO_4+H^+]$: 460.1543; found 460.1543.

^{1}H NMR (700 MHz, $CDCl_3$) δ 9.62 (s, 1H), 7.97 – 7.95 (m, 1H), 7.72 – 7.69 (m, 1H), 7.61 – 7.59 (m, 1H), 7.33 – 7.32 (m, 1H), 7.17 – 7.15 (m, 2H), 7.12 – 7.10 (m, 1H), 7.10 – 7.08 (m, 2H), 7.08 – 7.06 (m, 2H), 7.05 – 7.02 (m, 2H), 6.97 – 6.92 (m, 2H) 6.62 – 6.61 (m, 1H), 4.57 – 4.56 (m, 1H), 4.13 – 4.09 (m, 1H), 3.46 – 3.43 (m, 1H), 3.36 – 3.34 (m, 1H).

^{13}C{^{1}H} NMR (176 MHz, $CDCl_3$) δ 184.9, 163.3, 161.7, 156.4, 153.2, 142.7, 138.1, 134.8, 134.2, 134.1, 128.6 (2C), 128.5 (2C), 128.4 (2C), 128.1 (2C), 127.7, 127.2, 125.8, 125.1, 123.4, 117.8, 117.3, 113.2, 109.9, 50.2, 49.3, 37.5.

4-((2*S*,3*R*)-3-(5-Formylfuran-2-yl)-2,3-diphenylpropyl)-2-oxo-2*H*-chromene-3-carbonitrile minor – 3s

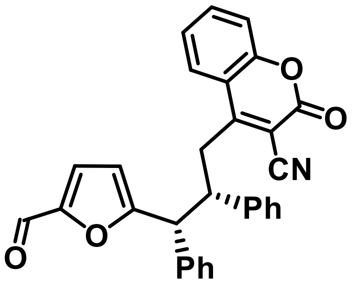

Minor diastereoisomer **3s** was isolated as light-orange oil (14.8 mg). The er was determined by UPC^2 using a chiral Chiralpack IB column gradient from 100% CO_2 up to 40%; *i*-PrOH, 2.5 mL/min; t_R = 4.801 (major), t_R = 4.667 (minor) : 63:37 er. $[\alpha]_D^{21}$ = - 72.1 (c = 1.0, $CHCl_3$). HRMS m/z $[M+H]^+$ calculated for $[C_{30}H_{21}ClNO_4+H^+]$: 460.1543; found 460.1543.

^{1}H NMR (700 MHz, $CDCl_3$) δ 9.38 (s, 1H), 7.66 – 7.65 (m, 1H), 7.64 – 7.62 (m, 2H), 7.48 – 7.45 (m, 2H), 7.41 – 7.36 (m, 1H), 7.35 – 7.34 (m, 1H), 7.33 – 7.32 (m, 1H), 7.17 – 7.15 (m, 2H), 7.14 – 7.12 (m, 2H), 7.07 – 7.06 (m, 2H), 6.88 (d, *J* = 3.6 Hz, 1H), 6.07 (d, *J* = 3.6 Hz, 1H), 4.54 (d, *J* = 11.3 Hz, 1H), 3.95 – 3.91 (m, 1H), 3.31 – 3.28 (m, 1H), 3.28 – 3.27 (m, 1H).

^{13}C{^{1}H} NMR (176 MHz, $CDCl_3$) δ 177.0, 163.4, 161.5, 156.4, 153.4, 152.1, 138.6, 138.5, 135.0, 129.5 (2C), 129.0 (2C), 128.8 (2C), 128.6, 128.1, 127.9 (2C), 126.0, 125.5, 118.0, 117.2, 113.5, 110.5, 103.3, 52.5, 50.8, 37.9.

4. **Synthesis of 4-((2*R*,3*R*)-3-(3-formylfuran-2-yl)-2,3-diphenylpropyl)-2-oxo-2*H*-chromene-3-carbonitrile major – 3a** and **4-((2*S*,3*R*)-3-(3-formylfuran-2-yl)-2,3-diphenylpropyl)-2-oxo-2*H*-chromene-3-carbonitrile minor – 3a on a 1 mmol scale**

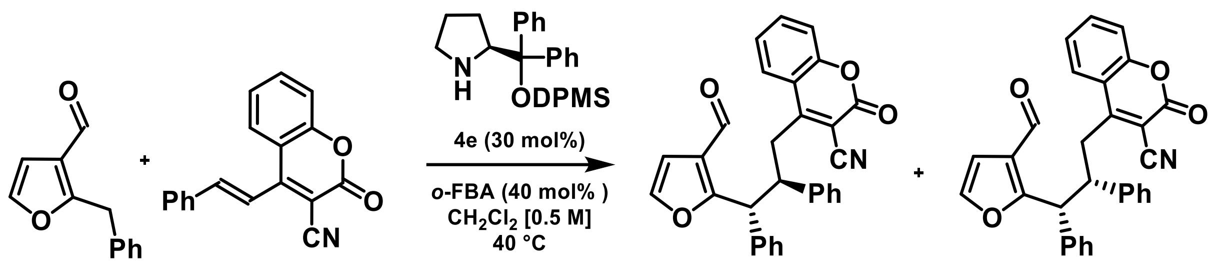

1a **2a** **major-3a** **minor-3a**

In an ordinary 10 mL flask with a magnetic stirring bar 2-benzyl-3-furfural **1a** (1.0 mmol, 1.0 equiv.) and 4-(alk-1-en-1-yl)-3-cyanocoumarin **2a** (1.0 mmol, 1.0 equiv.) were placed. The starting materials were dissolved in CH_2Cl_2 (2.0 mL). Catalyst – (*S*)-2-(((methyldiphenylsilyl)oxy)diphenylmethyl)pyrrolidine **4e** (0.3 mmol, 0.3 equiv.) and *o*-fluorobenzoic acid (0.4 mmol, 0.4 equiv.) were added. The resulting mixture was stirred for 72 hours in 40°C, and conversion of the starting material **1a** was controlled by ^{1}H NMR spectroscopy. Then, the reaction mixture was directly subjected to column chromatography on silica gel (hexane - ethyl acetate 4:1 to 3:2) to obtain pure product **3a** (2.2:1 dr in a crude reaction mixture) was isolated after 3 days with 74% yield: major-**3a** as a light-yellow oil (233.3 mg) and minor-**3a** as a light-yellow-oil (106.1 mg). NMR and HPLC data were in accordance with previously obtained results.

5. **Synthesis of 4-((2*S*,3*R*)-3-(3-(2,2-dibromovinyl)furan-2-yl)-3-phenyl-2-(*p*-tolyl)propyl)-2-oxo-2*H*-chromene-3-carbonitrile 5**

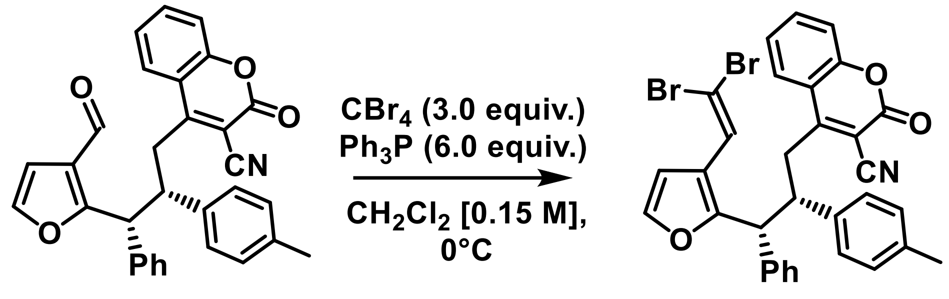

minor-3i **5** >20:1 dr

In a round-bottom flask, PPh_3 (6.0 equiv.) was dissolved in CH_2Cl_2 (1.7 M) and cooled to 0°C under argon atmosphere. A solution of CBr_4 (3.0 equiv.) in CH_2Cl_2 (0.7 M) was then added dropwise. The colorless solution turned yellow and then reddish. After stirring for 30 minutes at 0°C, **minor-3i** (0.1 mmol, 1.0 equiv.) was added dropwise and the reaction mixture was stirred at 0°C for 1.5 hours. The mixture was poured into water, the organic phase was separated, and the aqueous layer was extracted with methylene chloride (3 × 20 mL). The extracts were combined with the organic phase, dried over Na_2SO_4, and concentrated under reduced pressure. The residue was subjected to chromatography (hexane - ethyl acetate: 4:1) to isolate **5** with 74% yield (>20:1 dr).

4-((2*S*,3*R*)-3-(3-(2,2-dibromovinyl)furan-2-yl)-3-phenyl-2-(*p*-tolyl)propyl)-2-oxo-2*H*-chromene-3-carbonitrile 5

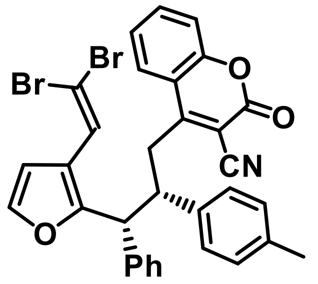

5 was isolated as light-orange oil (46.4 mg). $[\alpha]_D^{21}$ = - 49.1 (c = 1.0, $CHCl_3$). HRMS m/z $[M+H]^+$ calculated for $[C_{32}H_{23}Br_2NO_3+H^+]$: 628.0118; found 628.0117.

^{1}H NMR (700 MHz, $CDCl_3$) δ 7.67 – 7.65 (m, 2H), 7.64 – 7.62 (m, 1H), 7.46 – 7.44 (m, 2H), 7.40 – 7.39 (m, 1H), 7.37 – 7.33 (m, 2H), 7.29 – 7.28 (m, 1H), 7.15 (d, *J* = 2.0 Hz, 1H), 7.11 (s, 1H), 6.95 – 6.94 (m, 2H), 6.90 – 6.89 (m, 2H), 6.66 (d, *J* = 2.0 Hz, 1H), 4.36 – 4.34 (m, 1H), 3.96 – 3.92 (m, 1H), 3.32 – 3.20 (m, 2H), 2.21 (s, 3H).

^{13}C{^{1}H} NMR (176 MHz, $CDCl_3$) δ 163.9, 156.5, 153.4, 153.2, 141.2, 139.1, 137.5, 135.4, 134.8, 129.4 (2C), 129.3 (2C), 128.6 (2C), 128.3, 127.4 (2C), 127.3, 126.1, 125.2, 117.9, 117.6, 117.3, 113.6, 109.0, 103.1, 88.3, 50.5, 50.4, 37.9, 21.2.

6. Synthesis of 4-((6*S*,7*R*)-6,7-diphenyl-6,7-dihydrobenzofuran-5-yl)-2*H*-chromen-2-one 6

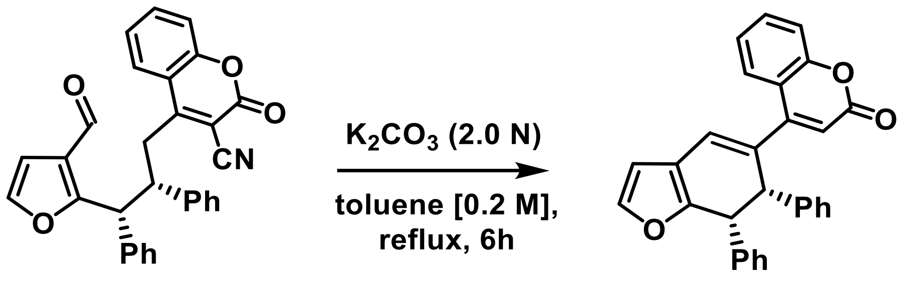

minor-3a

6
>20:1 dr

In a shlenk flask, with a magnetic stirring bar a **minor-3a** and K_2CO_3 (2.0 N) was dissolved in dry toluene [0.2 M] under argon atmosphere. The mixture was heated in reflux for 6 hours under argon atmosphere. Then, the mixture was poured into water, the organic phase was separated, and the aqueous layer was extracted with CH_2Cl_2 (3 × 20 mL). The extracts were combined with the organic phase, dried over Na_2SO_4, and concentrated under reduced pressure. The residue was subjected to chromatography (hexane - ethyl acetate: 4:1) to isolate **6** with 64% yield (>20:1 dr).

4-((6*S*,7*R*)-6,7-diphenyl-6,7-dihydrobenzofuran-5-yl)-2*H*-chromen-2-one 6

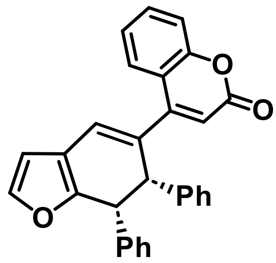

6 was isolated as orange oil (26.6 mg) $[\alpha]_D^{21}$ = - 102.2 (c = 1.0, $CHCl_3$). HRMS m/z $[M+H]^+$ calculated for $[C_{29}H_{20}O_3+H^+]$: 417.1485; found 417.1476

^{1}H NMR (700 MHz, $CDCl_3$) δ 7.90 – 7.89 (m, 1H), 7.53 – 7.51 (m, 1H), 7.38 (d, *J* = 1.9, 1H), 7.33 – 7.29 (m, 2H), 7.19 – 7.15 (m, 1H), 7.12 – 7.10 (m, 2H), 7.06 – 7.01 (m, 1H), 6.97 – 6.93 (m, 2H), 6.84 (s, 1H), 6.83 – 6.77 (m, 2H), 6.71 – 6.67 (m, 2H), 6.53 (d, *J* = 1.9 Hz, 1H), 6.01 (s, 1H), 4.93 (d, *J* = 8.5 Hz, 1H), 4.18 (d, *J* = 8.5 Hz, 1H).

^{13}C{^{1}H} NMR (176 MHz, $CDCl_3$) δ 160.9, 155.3, 154.2, 152.9, 143.0, 135.8, 135.5, 131.8, 131.7, 129.51 (2C), 129.5 (2C), 128.1 (2C), 128.0 (2C), 127.5, 127.4, 126.6, 125.2, 124.2, 119.1, 118.7, 117.6, 113.9, 108.5, 54.2, 48.1.

7. **Crystal and X-ray data for 3a – major and 3d - minor**

The crystal structure of the compound **4-((2*R*,3*R*)-3-(3-formylfuran-2-yl)-2,3-diphenylpropyl)-2-oxo-2*H*-chromene-3-carbonitrile major – 3a**, $C_{30}H_{21}NO_4$, was established by single-crystal X-ray diffraction at 100 K. The compound crystallizes in the non-centrosymmetric orthorhombic space group *P*-1 (Z = 2), with two crystallographically independent formula units per unit cell (Figure **1**). Method for crystal growth was liquid-liquid diffusion, solvent system was hexane:ethyl acetate 3:2.

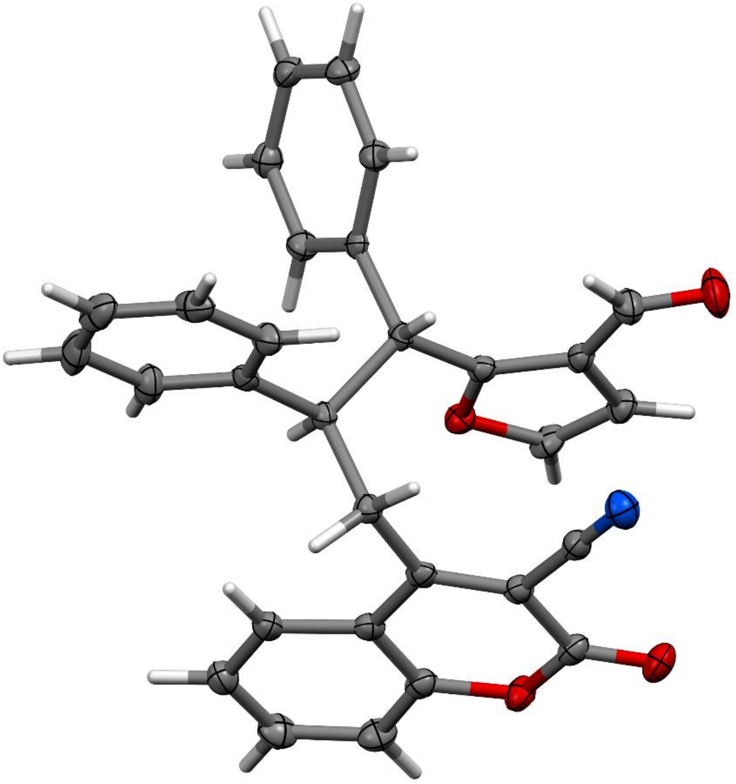

Figure **1**. The molecular structure of the major – **3a** compound K (one of two independent molecules), showing the atom-labelling scheme and displacement ellipsoids for the non-H atoms at the 50% probability level.

The crystal structure of the compound **4-((2*S*,3*R*)-2-(3-chlorophenyl)-3-(3-formylfuran-2-yl)-3-phenylpropyl)-2-oxo-2*H*-chromene-3-carbonitrile minor – 3d**, $C_{30}H_{20}ClNO_4$, was established by single-crystal X-ray diffraction at 100 K. The compound crystallizes in the non-centrosymmetric orthorhombic space group $P2_12_12_1$ (Z = 4), with one crystallographically independent formula unit per unit cell (Figure **2**). Method for crystal growth was liquid-liquid diffusion, solvent system was hexane:ethyl acetate 3:2.

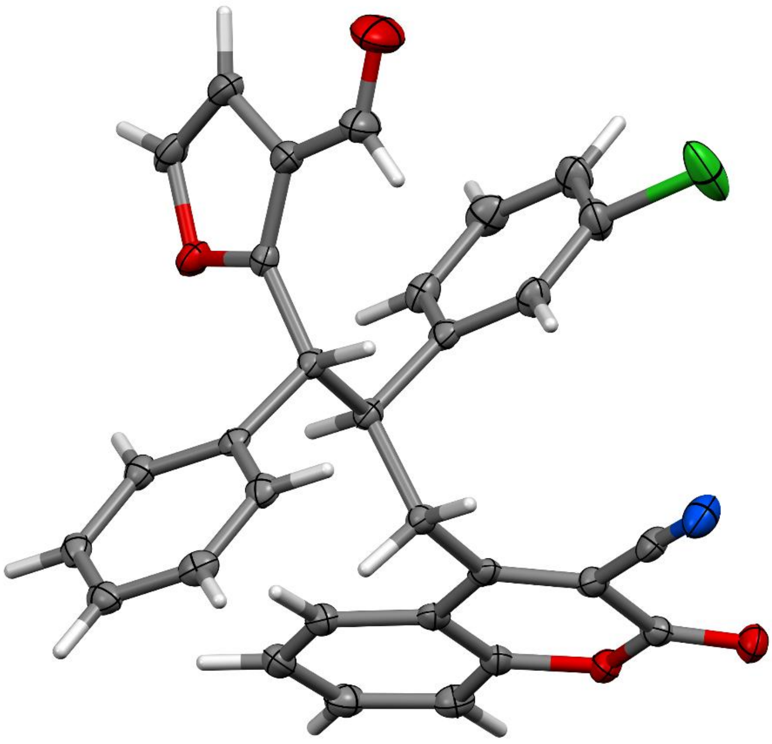

Figure **2**. The molecular structure of the minor – **3d** compound, showing the atom-labelling scheme and displacement ellipsoids for the non-H atoms at the 50% probability level.

Single crystal X-ray diffraction data were collected at 100 K by the ω-scan technique using a RIGAKU XtaLAB Synergy, Dualflex, Pilatus 300K diffractometer with PhotonJet micro-focus X-ray Source Cu-Kα (λ = 1.54184 Å).[4] Data collection, cell refinement, data reduction and absorption correction were performed using CrysAlis PRO software.[4] The crystal structure was solved using direct methods and the SHELXT 2018/2 program, with atomic scattering factors taken from the International Tables for X-ray Crystallography.[5] Positional parameters of non-H-atoms were refined by a full-matrix least-squares method on F^2 with anisotropic thermal parameters by using the SHELXL 2019/3 program.[6] All hydrogen atoms were found from the difference Fourier maps and were subsequently positioned geometrically in calculated positions (C–H = 0.95–1.00 Å) and constrained to ride on their parent atoms with isotropic displacement parameters set to 1.2 times the U_{eq} of the parent atom.

4-((2*R*,3*R*)-3-(3-Formylfuran-2-yl)-2,3-diphenylpropyl)-2-oxo-2*H*-chromene-3-carbonitrile major – 3a: Formula $C_{30}H_{21}NO_4$, triclinic, space group *P*-1, Z = 2, unit cell constants *a* = 8.6492(1), *b* = 11.8188(2), *c* = 12.0770(2) Å, α = 106.902(1), β = 95.494(1), γ = 102.213(1)°, *V* = 1138.05(3) Å^3. The integration of the data yielded a total of 28859 reflections with θ angles in the range of 3.88 to 67.73°, of which 7830 were unique (R_{int} = 2.50%). The final anisotropic full-matrix least-squares refinement on F^2 with 631 parameters. The final R_1 was 0.0282 (for I > 2σ(I)) and wR_2 was 0.0766 (all data). The largest peak in the final difference electron density synthesis was 0.201 eÅ^{-3} and the largest hole was -0.186 eÅ^{-3}. The goodness-of-fit was 1.013. The absolute configuration was established from anomalous scattering, by calculating the x Flack parameter [7] of 0.12(6) using 3535 quotients.

4-((2*S*,3*R*)-2-(3-Chlorophenyl)-3-(3-formylfuran-2-yl)-3-phenylpropyl)-2-oxo-2*H*-chromene-3-carbonitrile minor – 3d: Formula $C_{30}H_{20}ClNO_4$, orthorhombic, space group $P2_12_12_1$, Z = 4, unit cell constants *a* = 9.2409(1), *b* = 14.7391(1), *c* = 17.6264(1) Å, *V* = 2400.76(3) Å^3. The integration of the data yielded a total of 88281 reflections with θ angles in the range of 3.91 to 67.70°, of which 4346 were unique (R_{int} = 3.36%). The final anisotropic full-matrix least-squares refinement on F^2 with 325 parameters. The final R_1 was 0.0295 (for I > 2σ(I)) and wR_2 was 0.0746 (all data). The largest peak in the final difference electron density synthesis was 0.595 eÅ^{-3} and the largest hole was -0.685 eÅ^{-3}. The goodness-of-fit was 1.020. The absolute configuration was unambiguously established from anomalous scattering, by calculating the x Flack parameter [7] of 0.005(3) using 1820 quotients.

CCDC 2404483 and **2404536** contain the supplementary crystallographic data for this paper. These data can be obtained free of charge from The Cambridge Crystallographic Data Centre via www.ccdc.cam.ac.uk/structures

4. Rigaku OD. CrysAlis PRO. Rigaku Oxford Diffraction Ltd, Yarnton, Oxfordshire, England, **2019**.

5. Sheldrick, G.M. "SHELXT - integrated space-group and crystal-structure determination", *Acta Cryst.* **2015**, *A71*, 3-8.

6. Sheldrick, G.M. "Crystal structure refinement with SHELXL", *Acta Cryst.* **2015**, *C71*, 3-8.

7. Parsons, S.; Flack, H. D.; Wagner, T. "Use of intensity quotients and differences in absolute structure refinement" *Acta Cryst.* **2013**, *B69*, 249-259.

8. NMR data

4-((2*R*,3*R*)-3-(3-Formylfuran-2-yl)-2,3-diphenylpropyl)-2-oxo-2*H*-chromene-3-carbonitrile major – 3a

^{1}H NMR (700 MHz, $CDCl_3$)

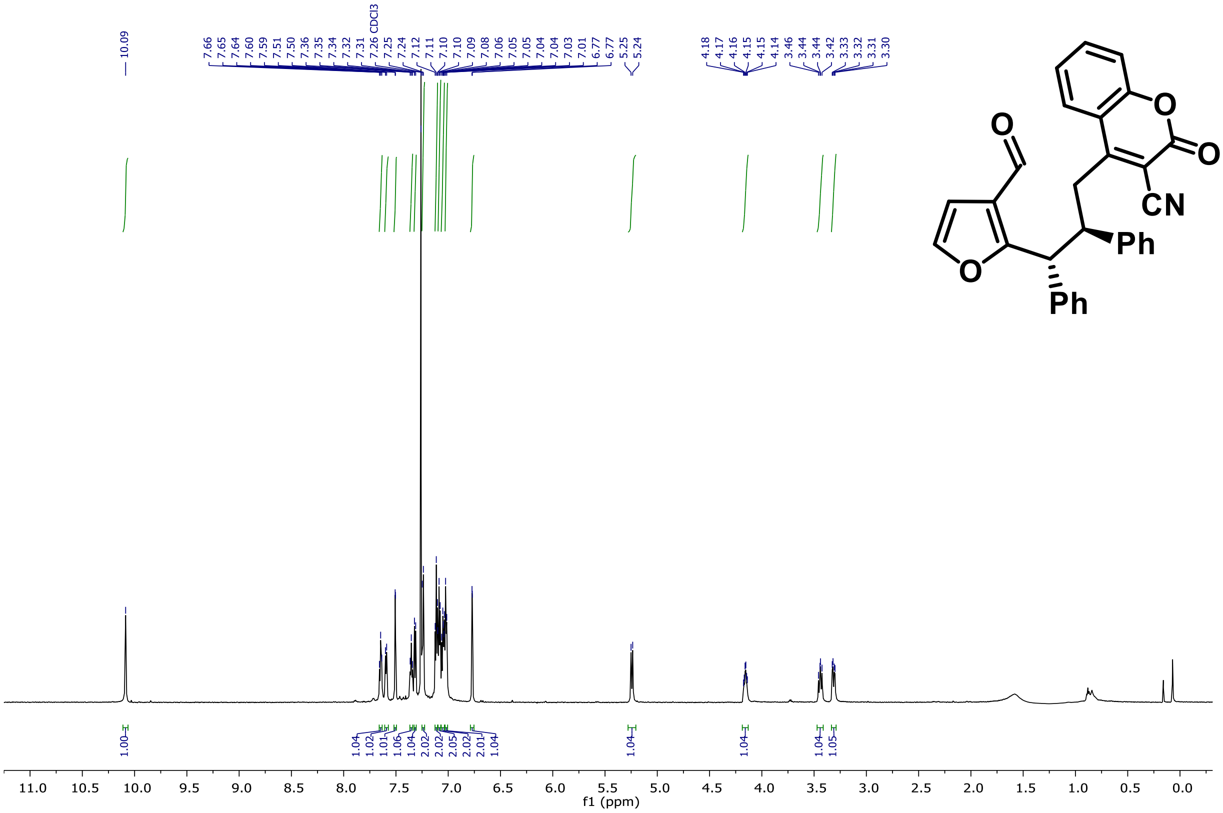

^{13}C{^{1}H} NMR (176 MHz, $CDCl_3$)

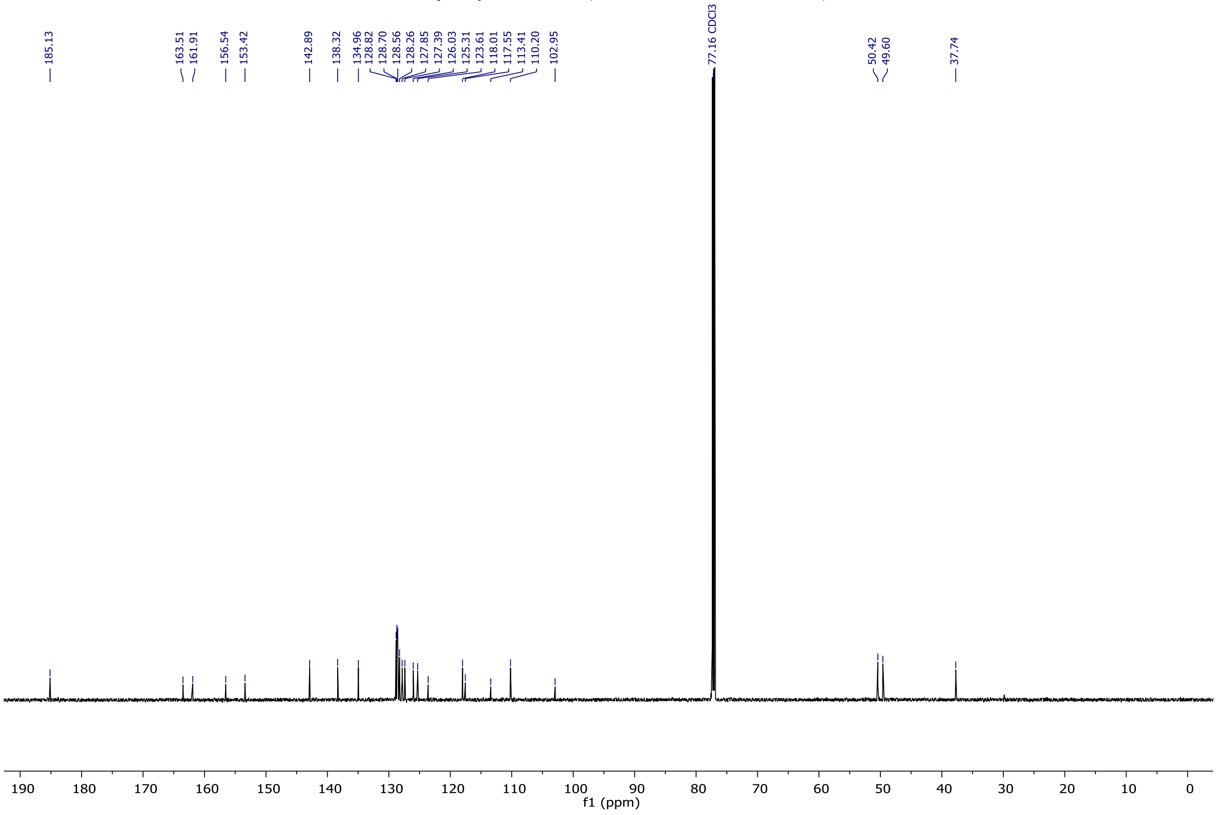

4-((2*S*,3*R*)-3-(3-Formylfuran-2-yl)-2,3-diphenylpropyl)-2-oxo-2*H*-chromene-3-carbonitrile minor – 3a

^{1}H NMR (700 MHz, CDCl$_3$)

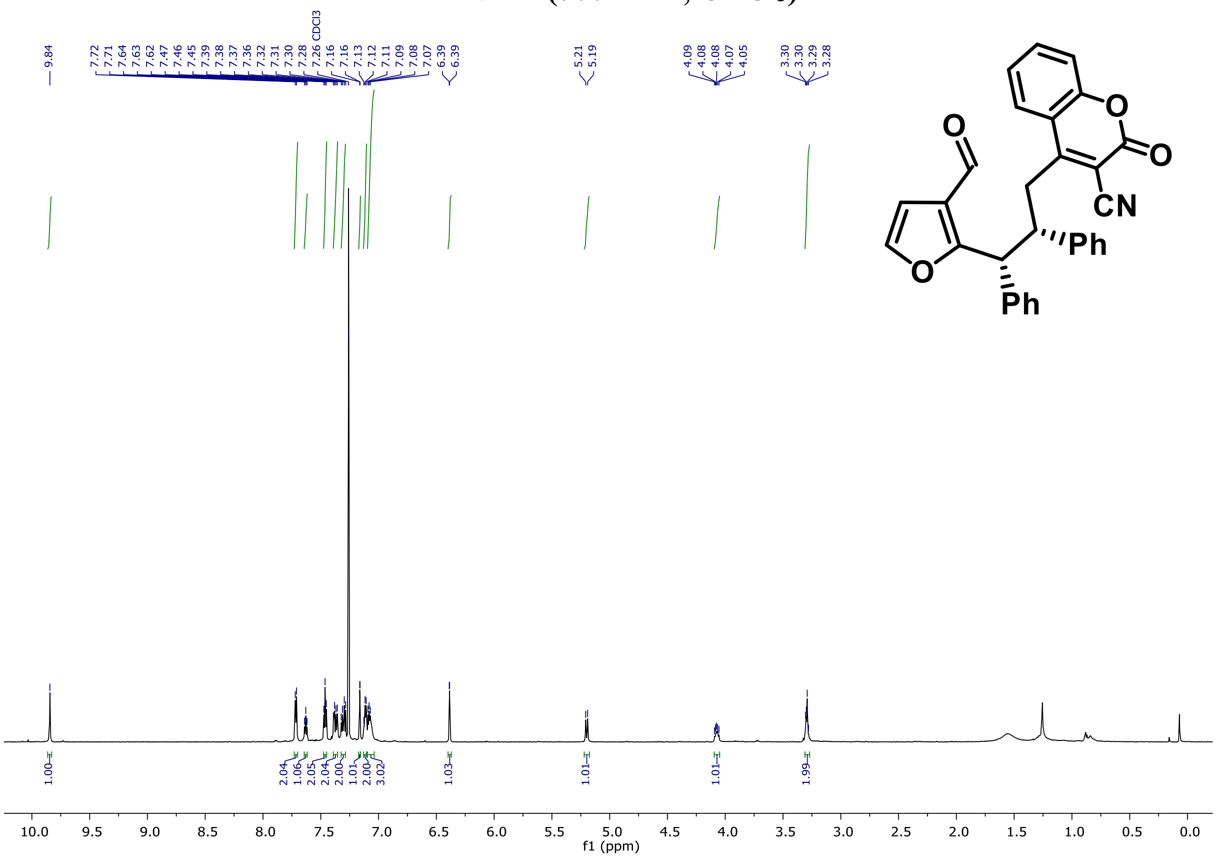

^{13}C{^{1}H} NMR (176 MHz, CDCl$_3$)

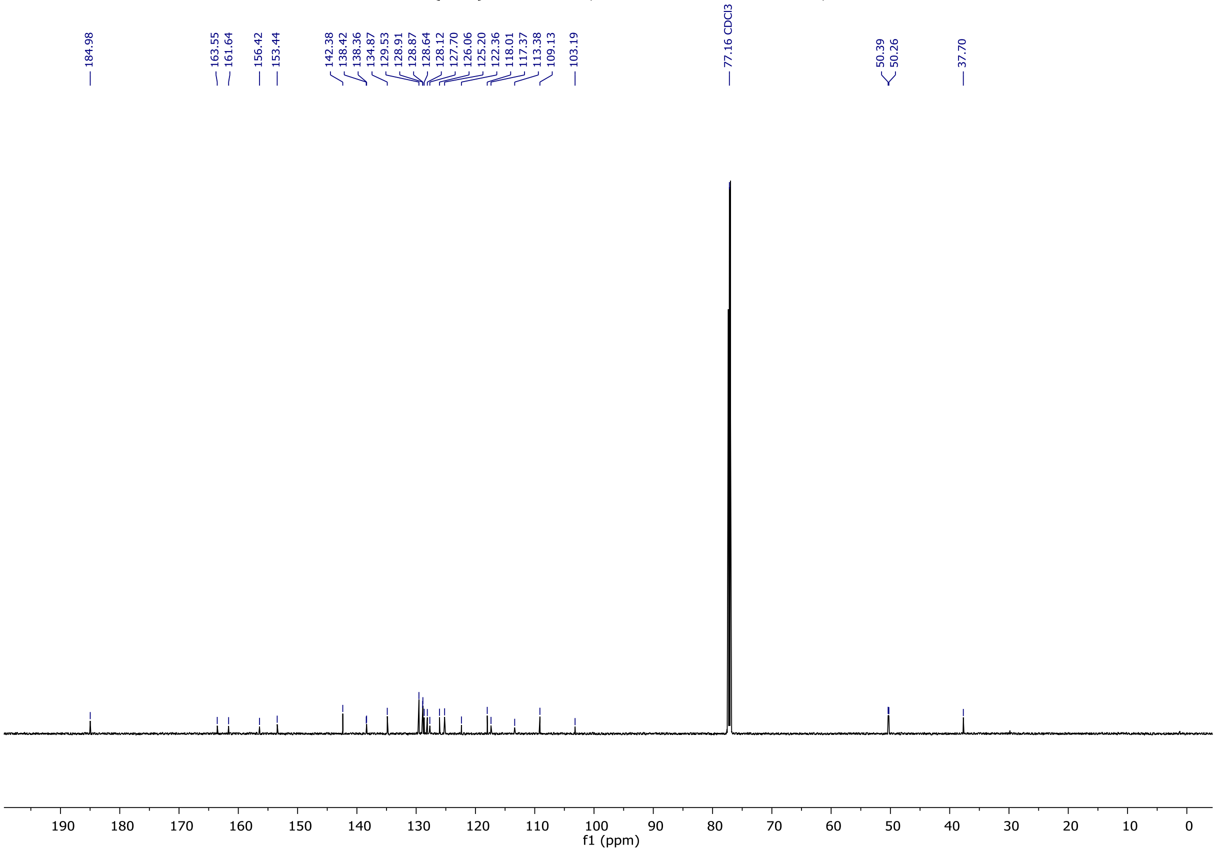

4-((2*R*,3*R*)-2-(3-Fluorophenyl)-3-(3-formylfuran-2-yl)-3-phenylpropyl)-2-oxo-2*H*-chromene-3-carbonitrile major – 3b

^{1}H NMR (700 MHz, $CDCl_3$)

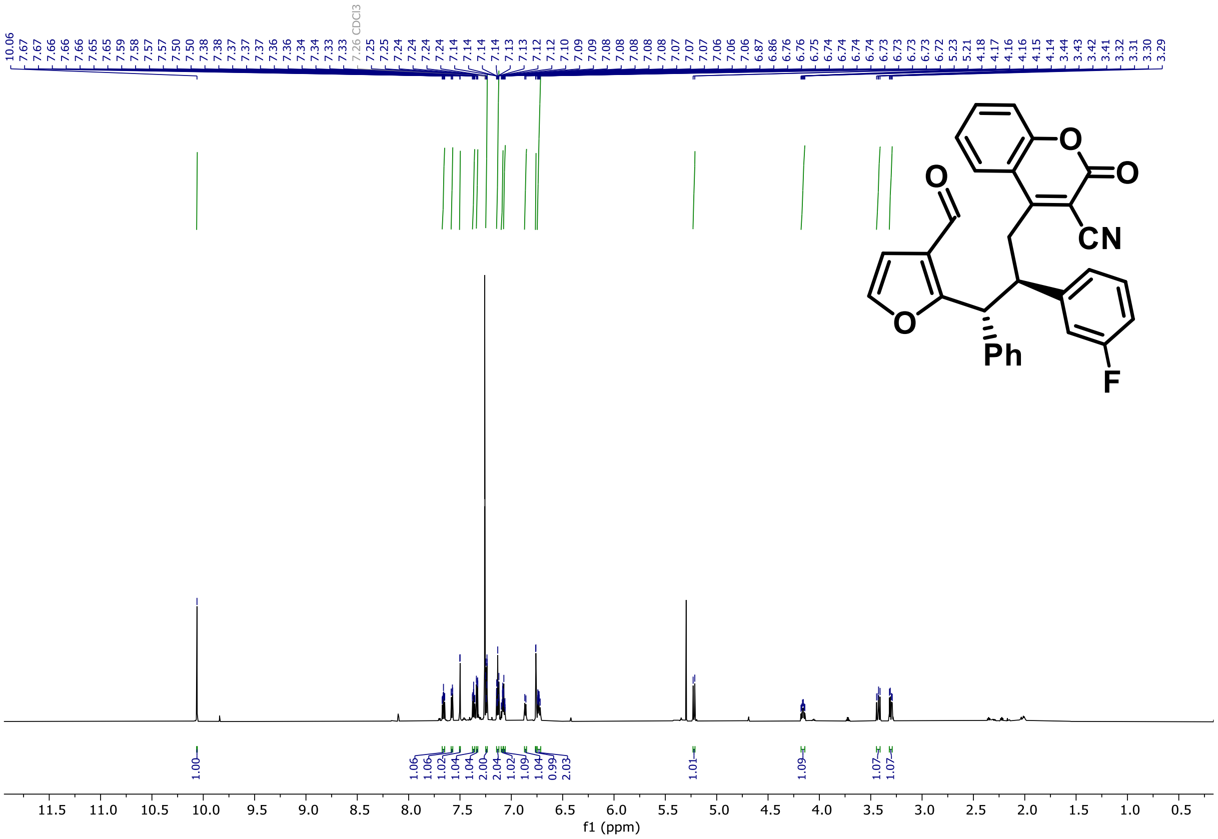

^{13}C{^{1}H} NMR (176 MHz, $CDCl_3$)

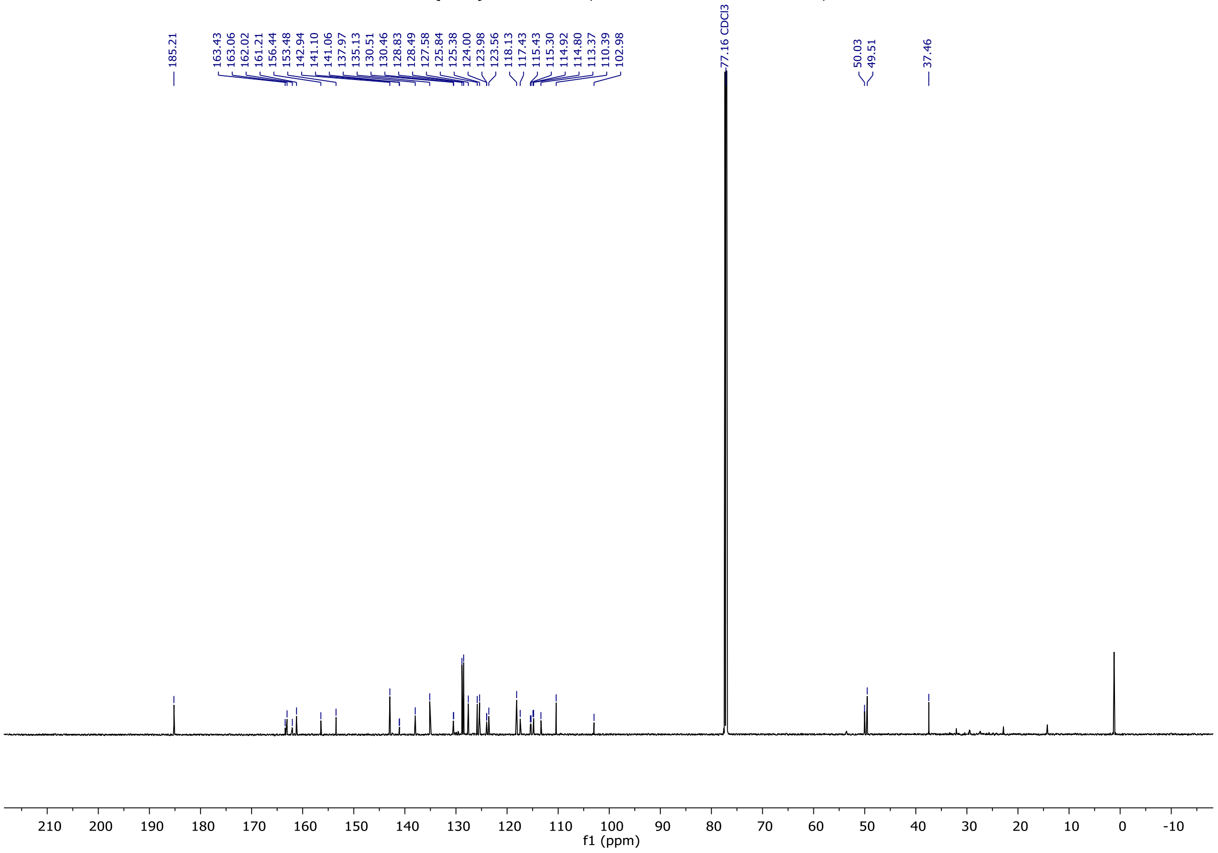

4-((2*S*,3*R*)-2-(3-Fluorophenyl)-3-(3-formylfuran-2-yl)-3-phenylpropyl)-2-oxo-2*H*-chromene-3-carbonitrile minor – 3b

^{1}H NMR (700 MHz, $CDCl_3$)

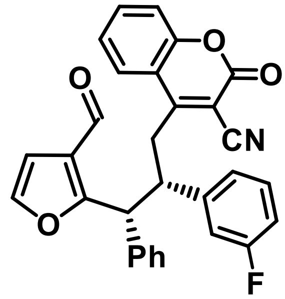

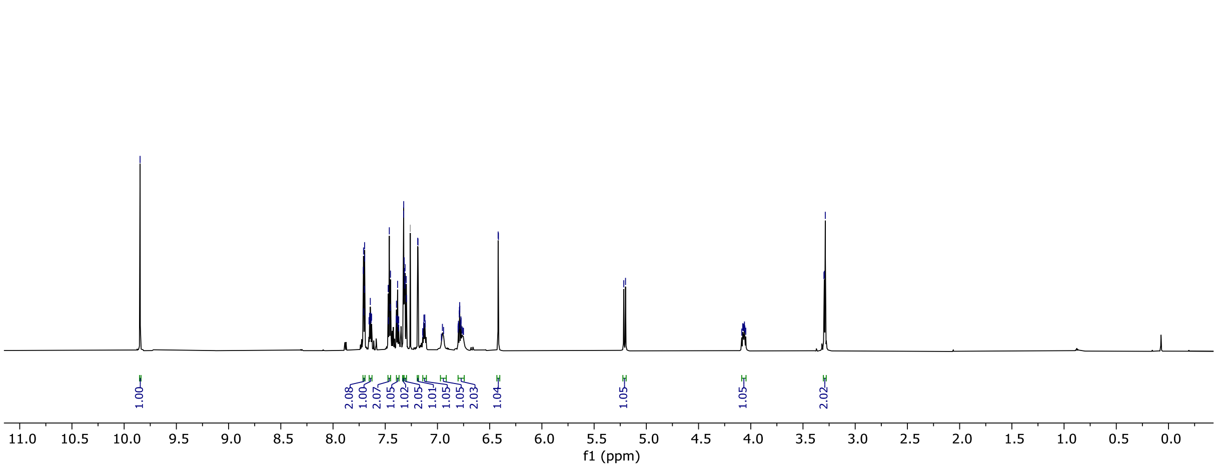

^{13}C{^{1}H} NMR (176 MHz, $CDCl_3$)

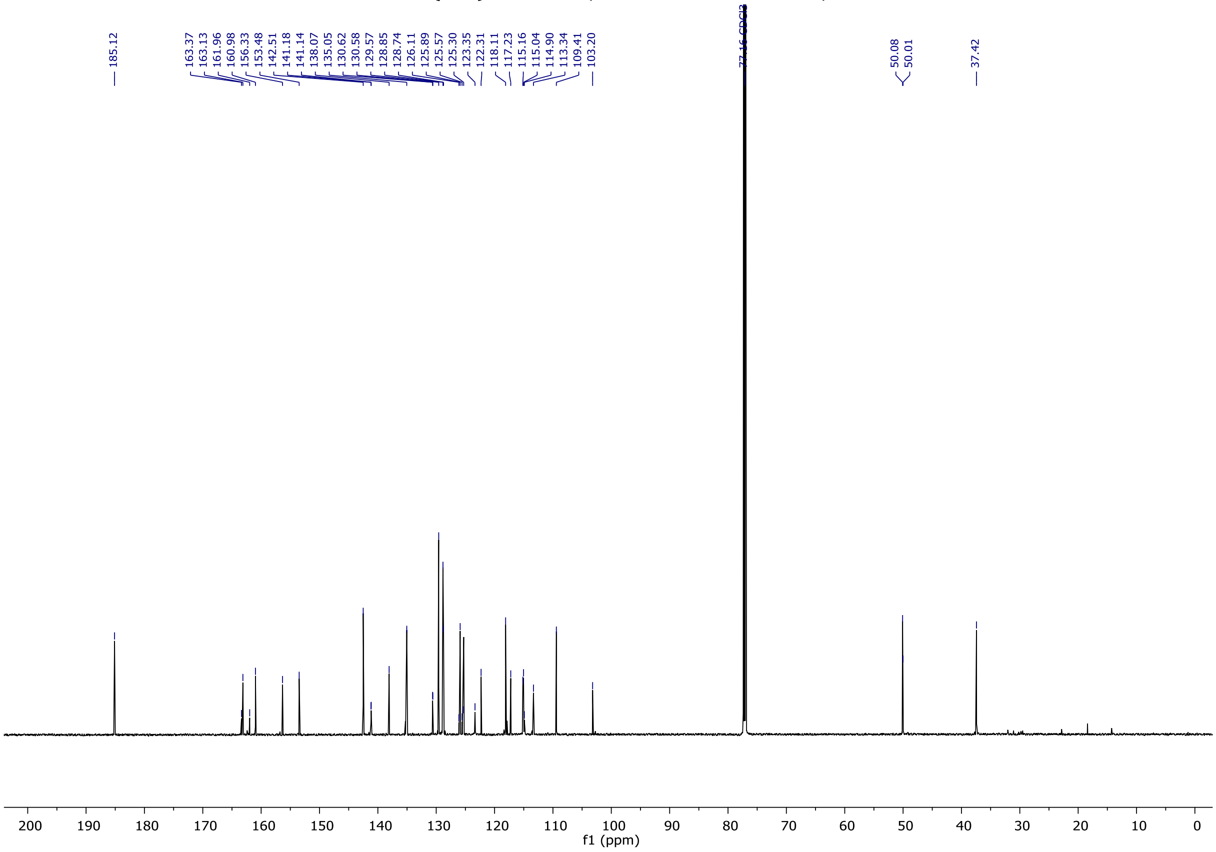

4-((2*R*,3*R*)-2-(2-Chlorophenyl)-3-(3-formylfuran-2-yl)-3-phenylpropyl)-2-oxo-2*H*-chromene-3-carbonitrile major – 3c

^{1}H NMR (700 MHz, CDCl$_3$)

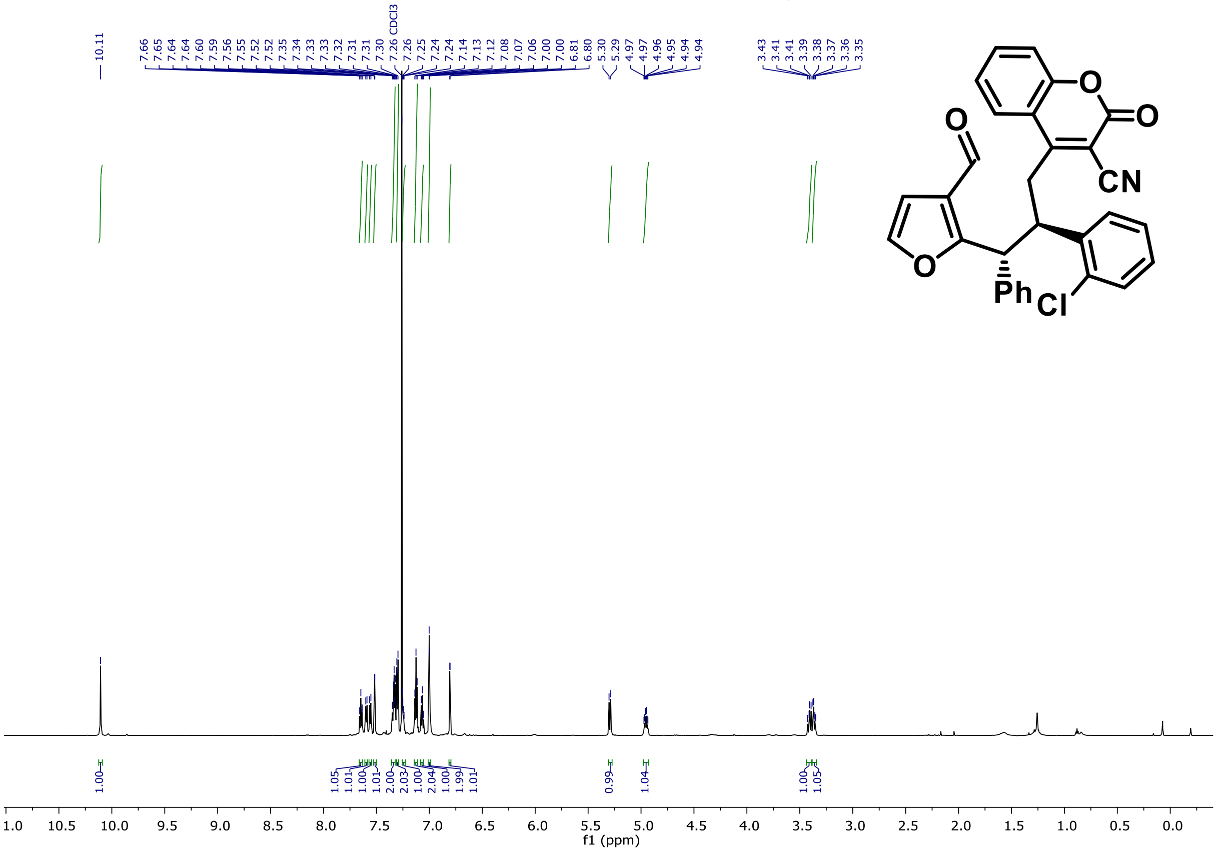

^{13}C{^{1}H} NMR (176 MHz, CDCl$_3$)

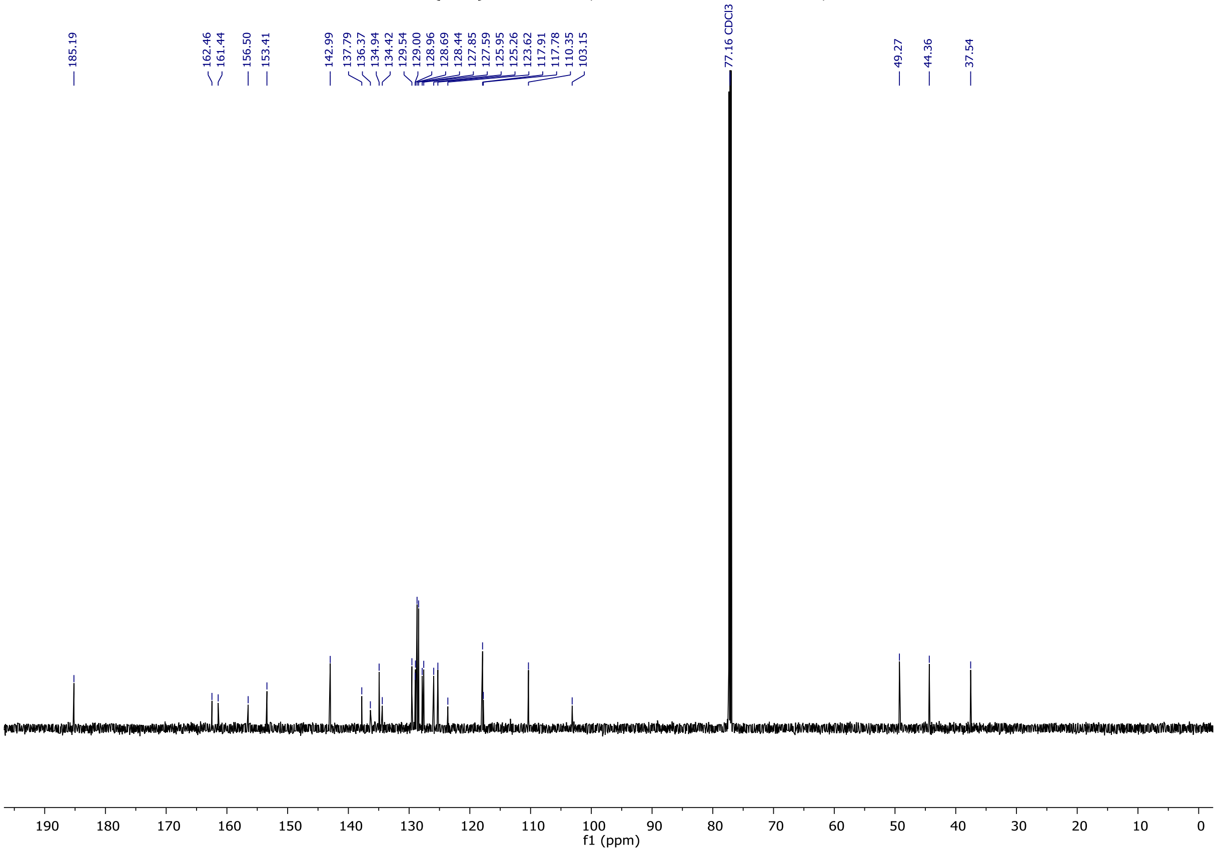

4-((2*S*,3*R*)-2-(2-Chlorophenyl)-3-(3-formylfuran-2-yl)-3-phenylpropyl)-2-oxo-2*H*-chromene-3-carbonitrile minor – 3c

^{1}H NMR (700 MHz, CDCl$_3$)

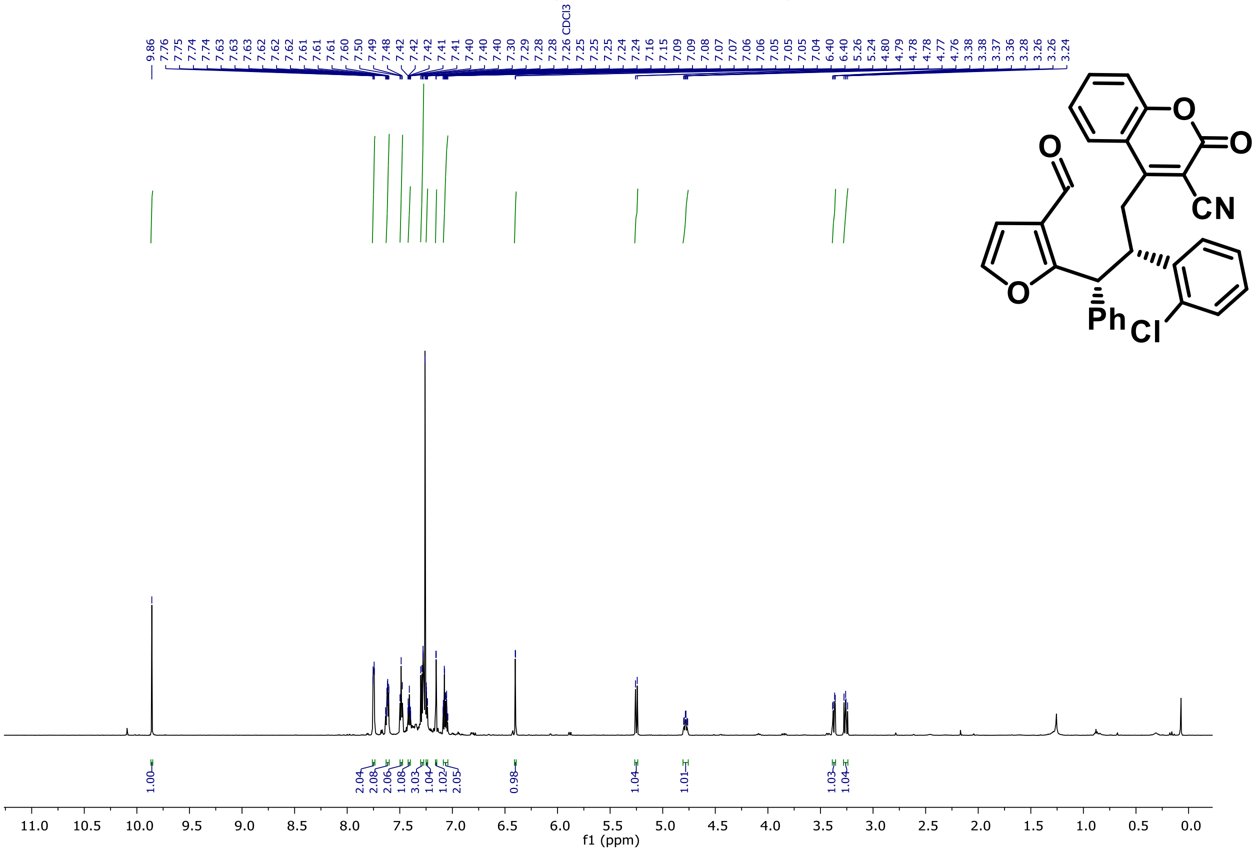

^{13}C{^{1}H} NMR (176 MHz, CDCl$_3$)

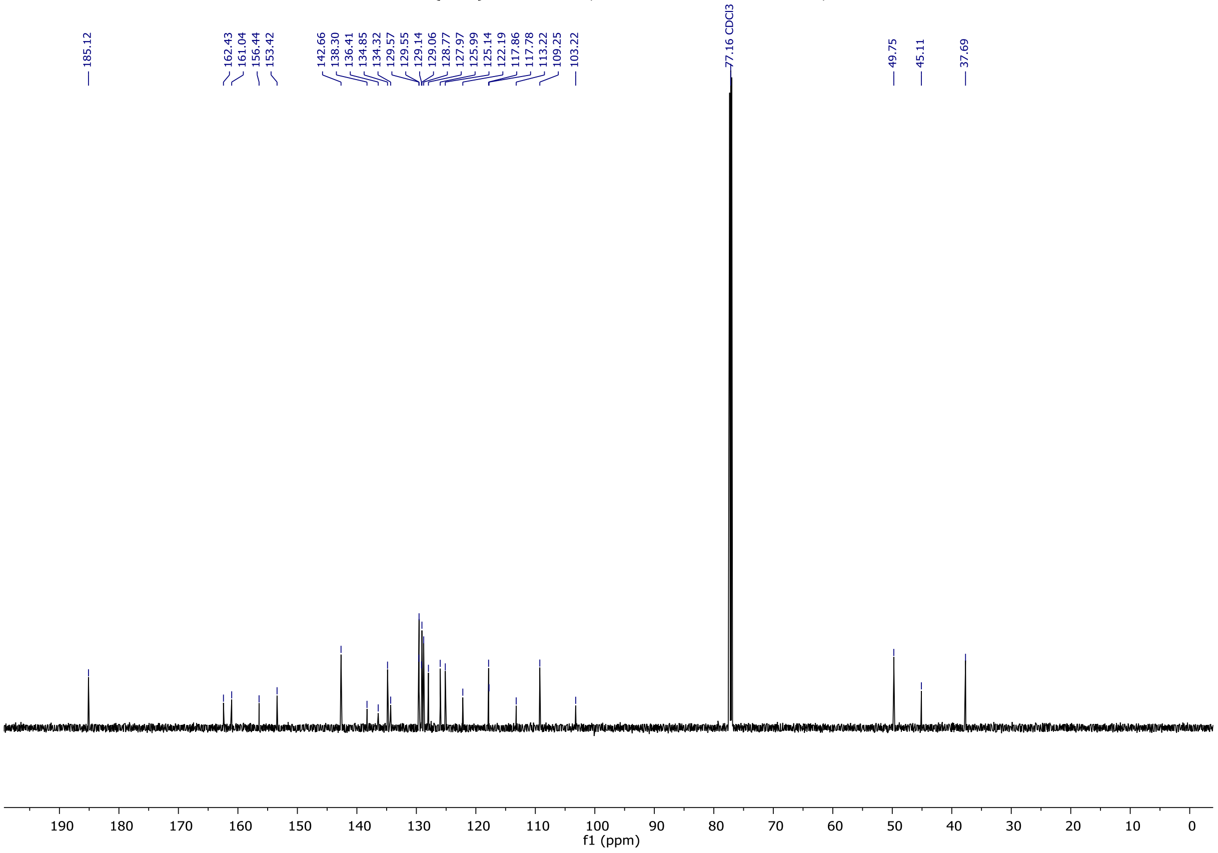

4-((2*R*,3*R*)-2-(3-Chlorophenyl)-3-(3-formylfuran-2-yl)-3-phenylpropyl)-2-oxo-2*H*-chromene-3-carbonitrile major – 3d

^{1}H NMR (700 MHz, $CDCl_3$)

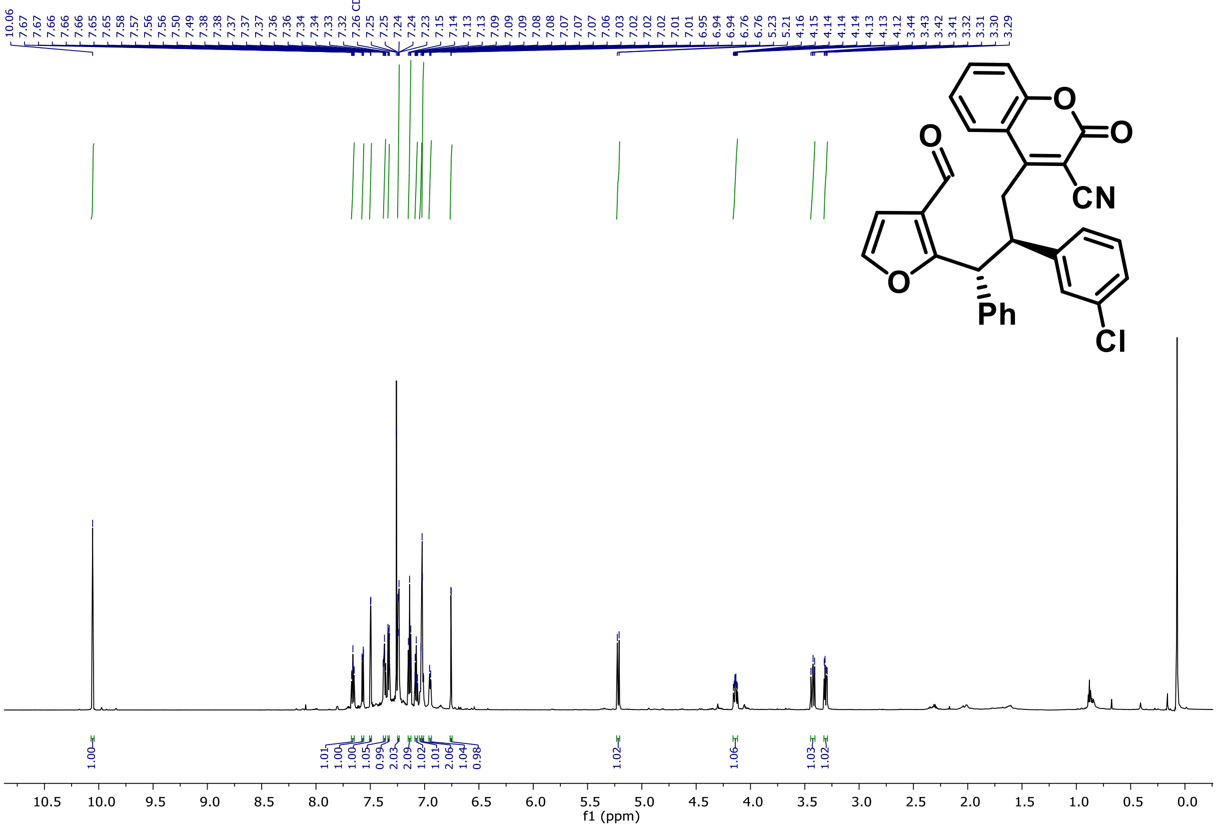

^{13}C{^{1}H} NMR (176 MHz, $CDCl_3$)

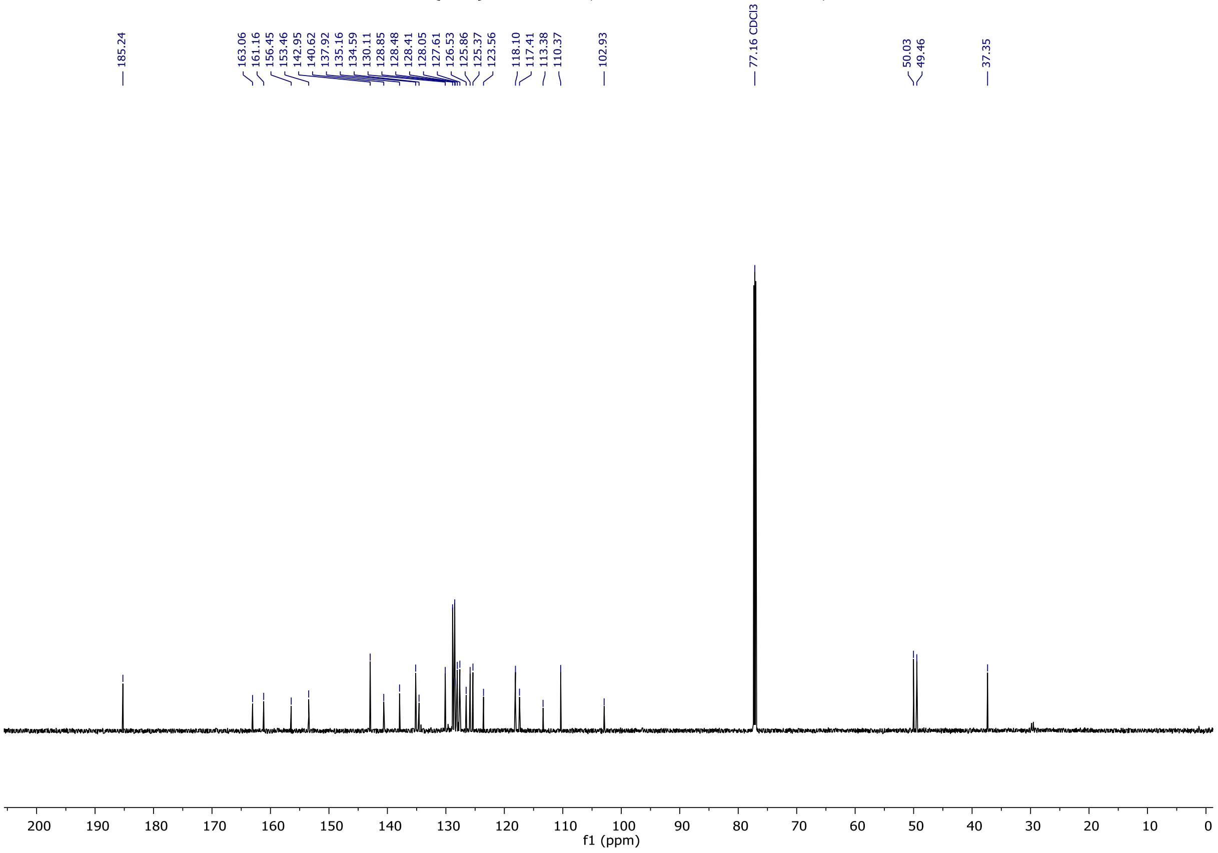

4-((2*S*,3*R*)-2-(3-Chlorophenyl)-3-(3-formylfuran-2-yl)-3-phenylpropyl)-2-oxo-2*H*-chromene-3-carbonitrile minor – 3d

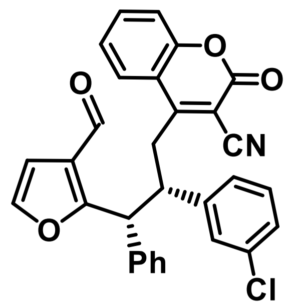

^{1}H NMR (700 MHz, CDCl₃)

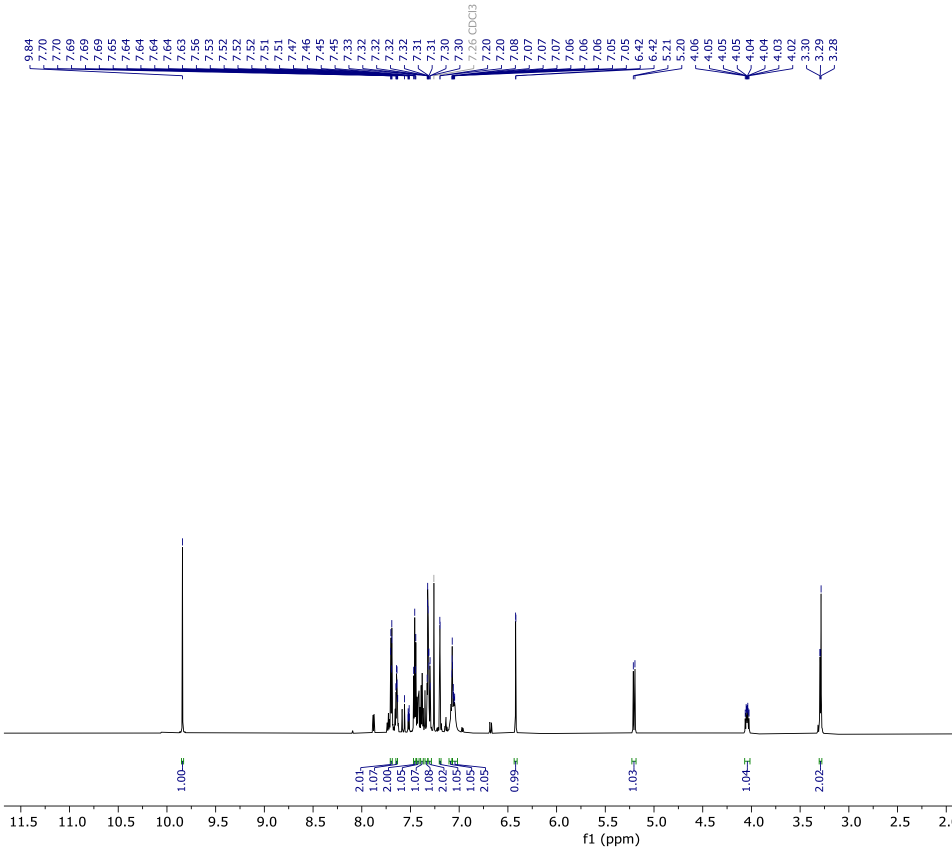

^{13}C{^{1}H} NMR (176 MHz, CDCl₃)

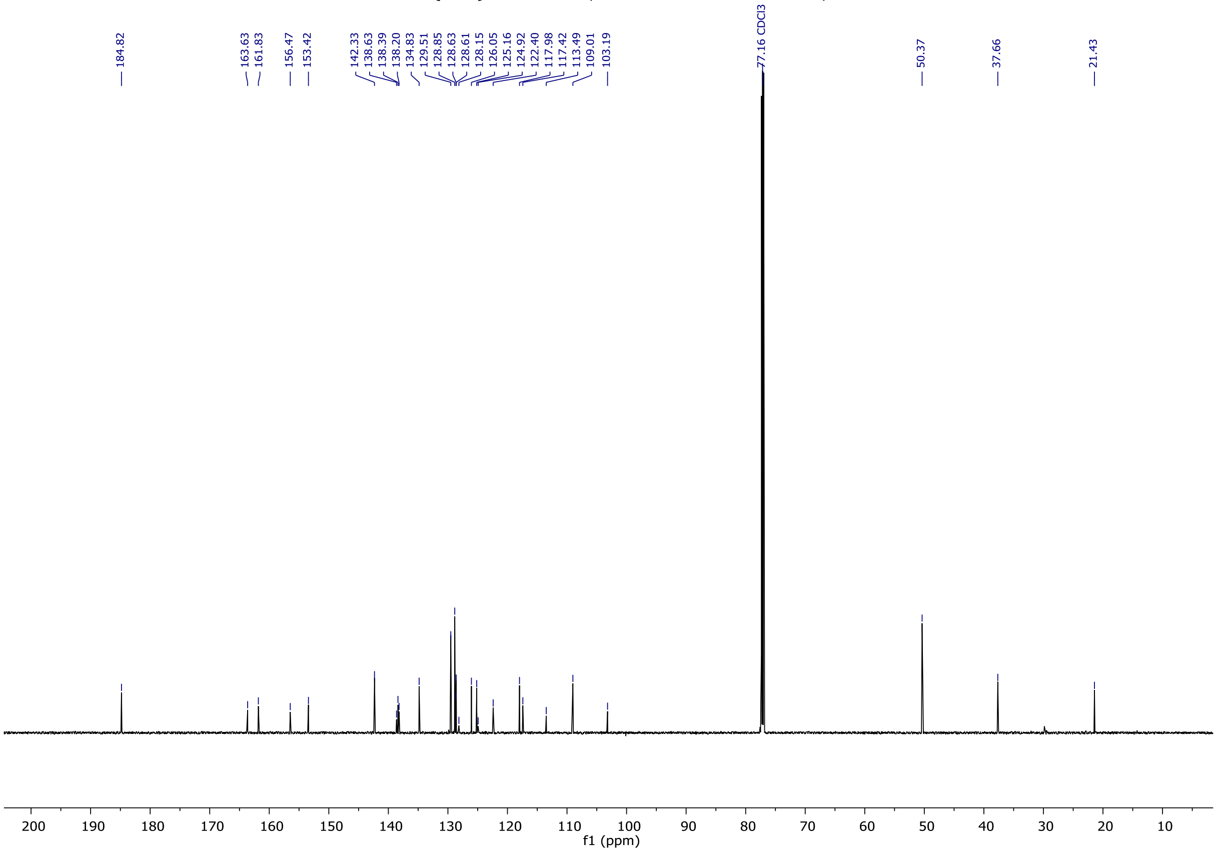

4-((2*R*,3*R*)-2-(4-Chlorophenyl)-3-(3-formylfuran-2-yl)-3-phenylpropyl)-2-oxo-2*H*-chromene-3-carbonitrile major – 3e

^{1}H NMR (700 MHz, $CDCl_3$)

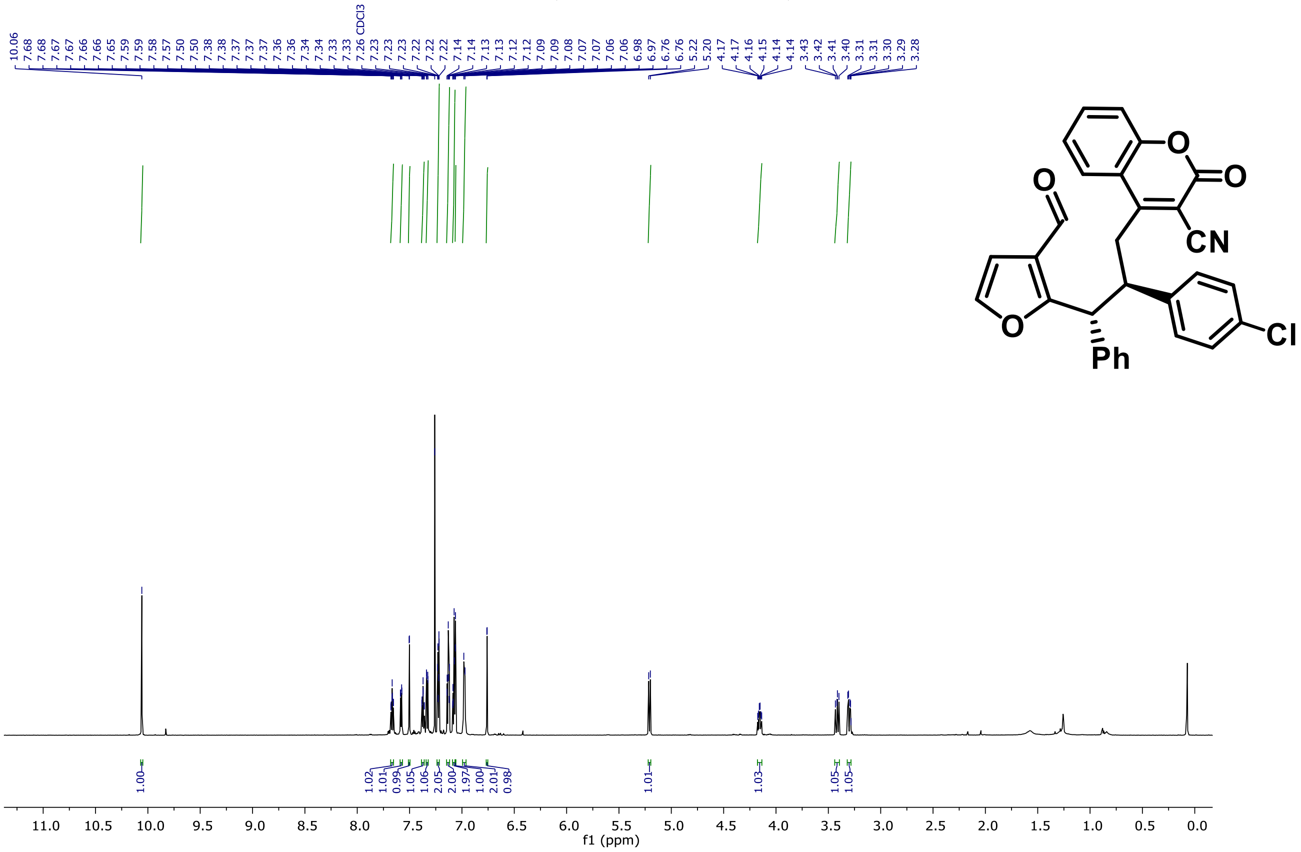

^{13}C{^{1}H} NMR (176 MHz, $CDCl_3$)

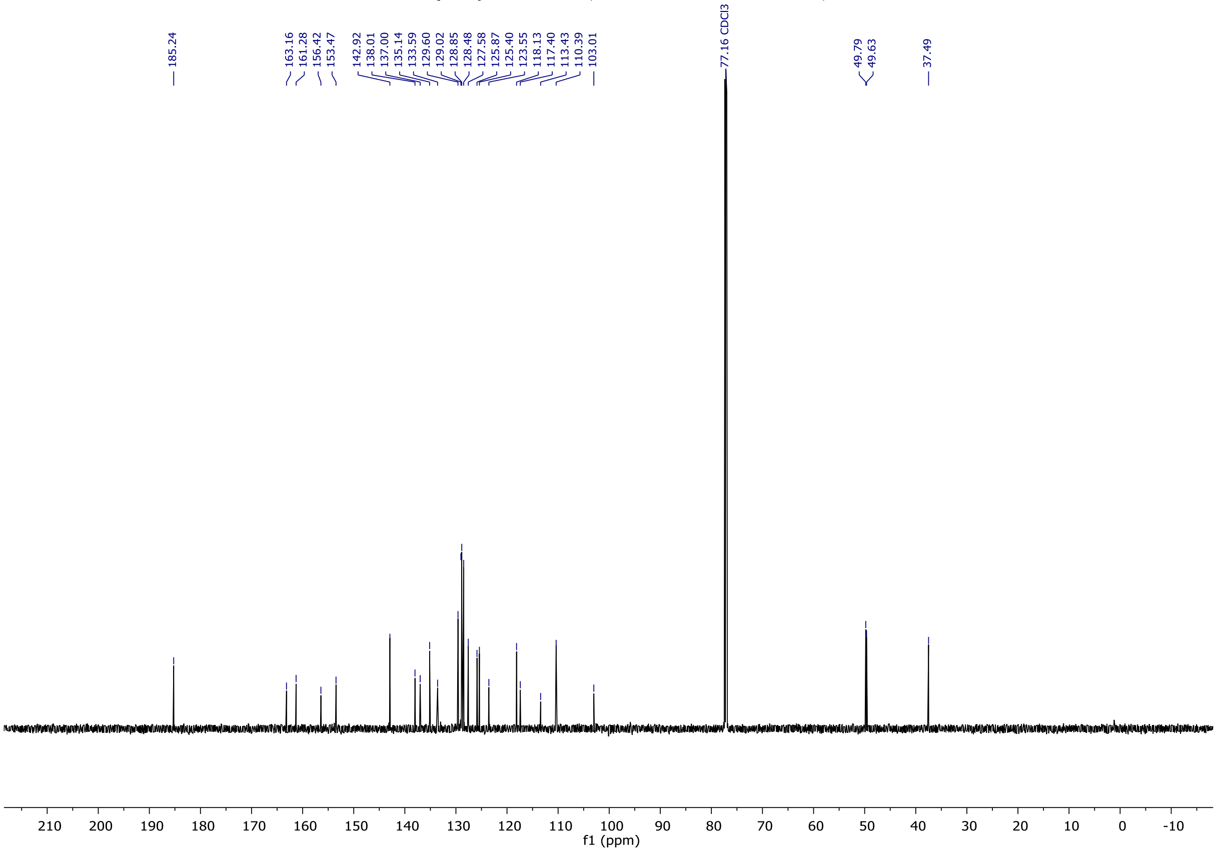

4-((2*S*,3*R*)-2-(4-Chlorophenyl)-3-(3-formylfuran-2-yl)-3-phenylpropyl)-2-oxo-2*H*-chromene-3-carbonitrile minor – 3e

^{1}H NMR (700 MHz, $CDCl_3$)

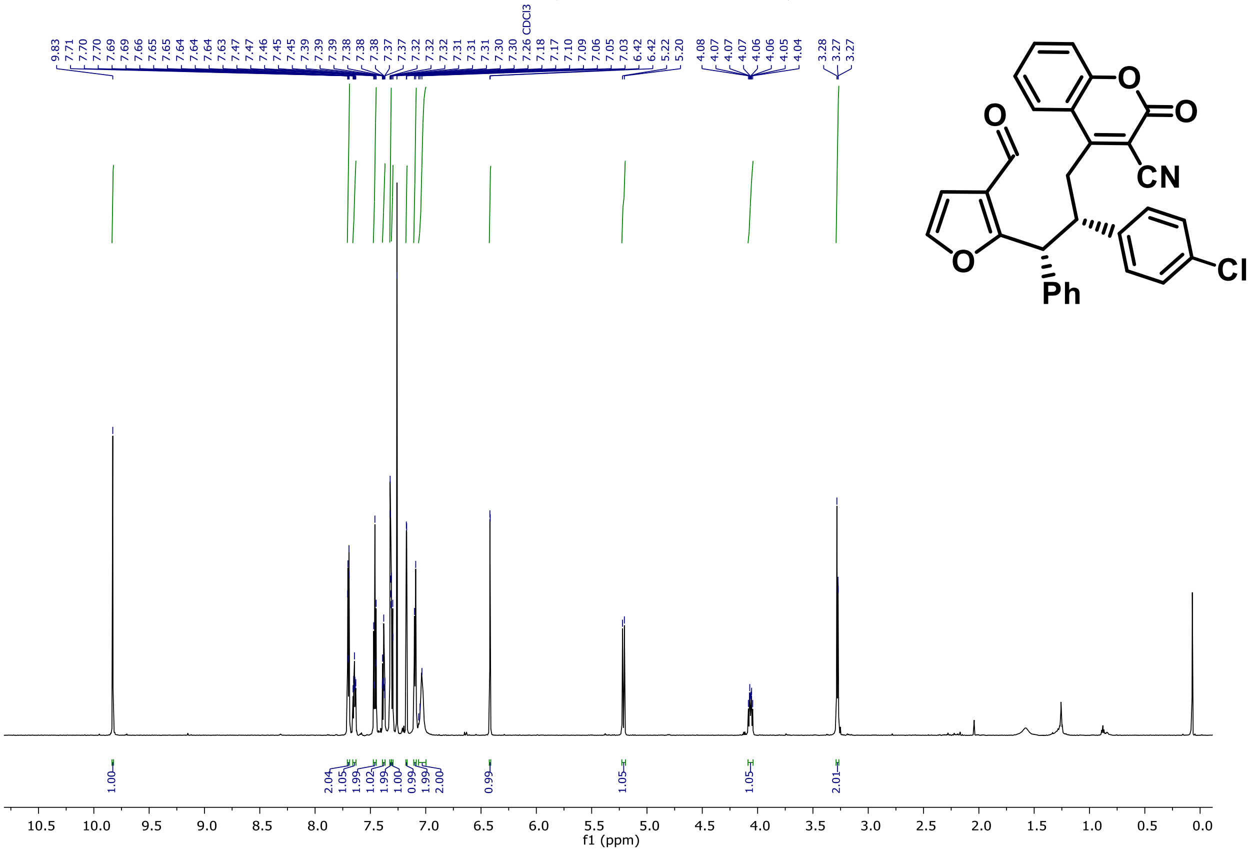

^{13}C{^{1}H} NMR (176 MHz, $CDCl_3$)

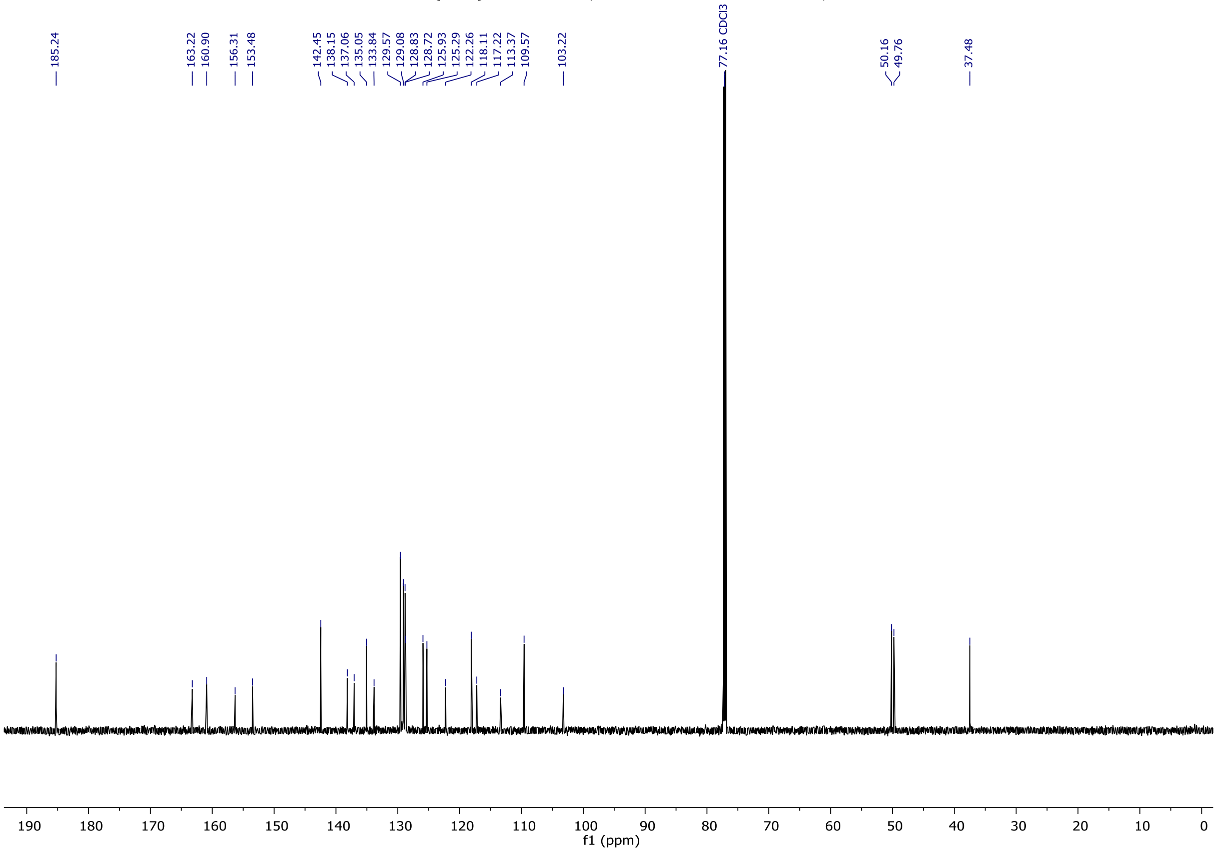

4-((2*R*,3*R*)-2-(4-Bromophenyl)-3-(3-formylfuran-2-yl)-3-phenylpropyl)-2-oxo-2*H*-chromene-3-carbonitrile major – 3f

^{1}H NMR (700 MHz, $CDCl_3$)

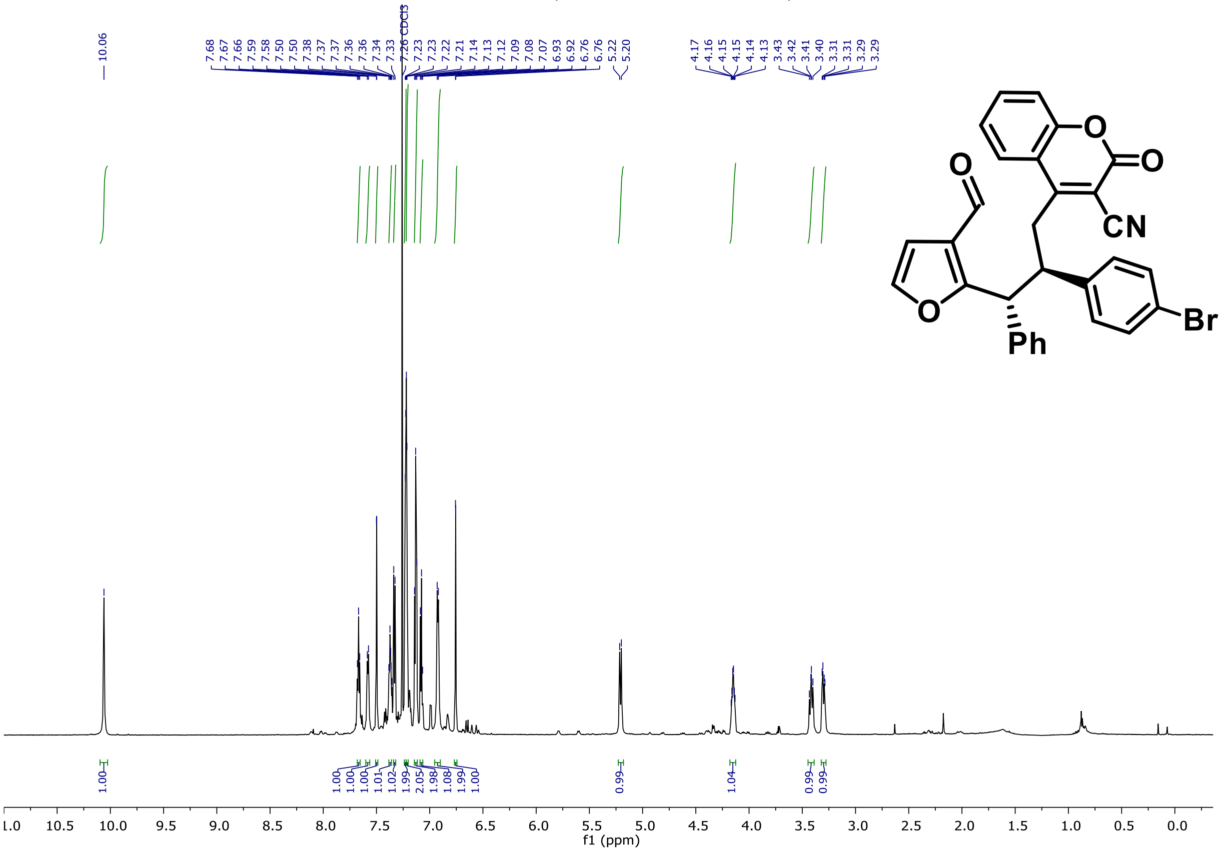

^{13}C{^{1}H} NMR (176 MHz, $CDCl_3$)

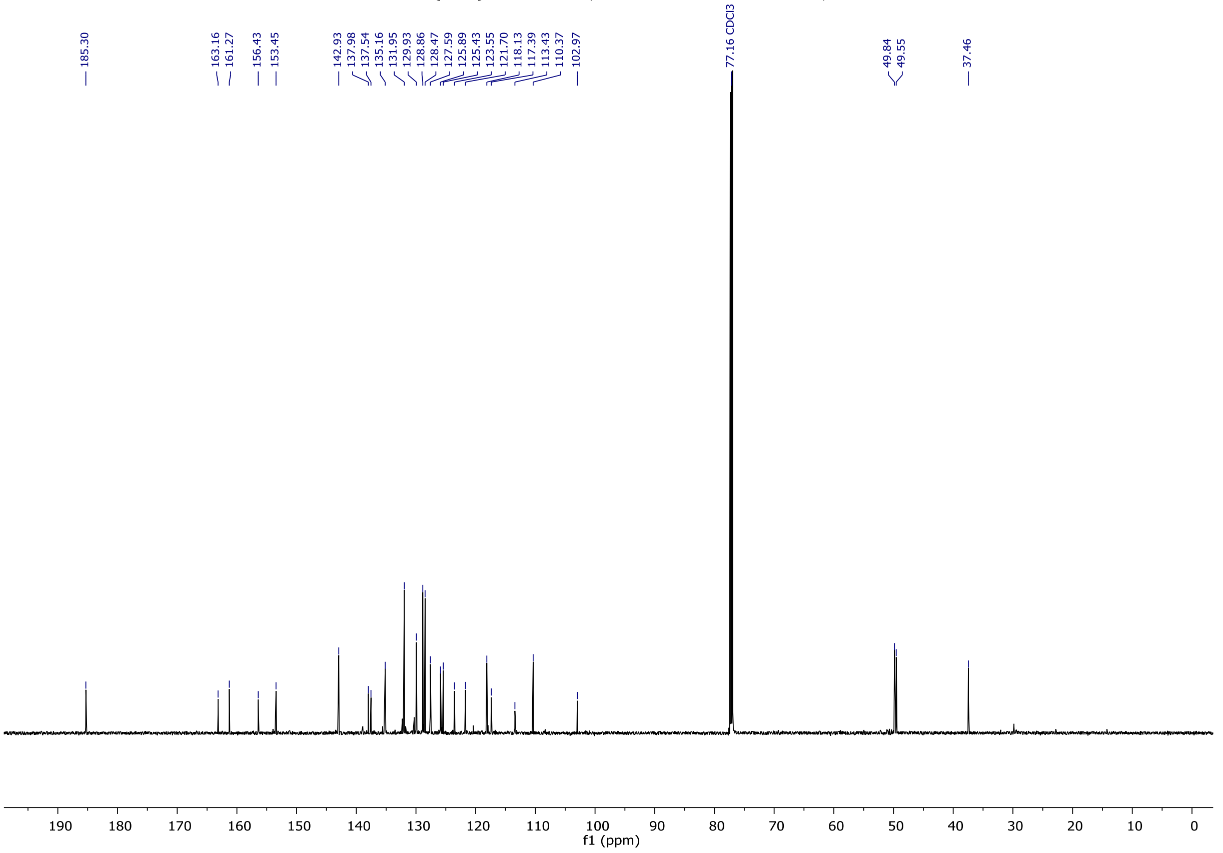

4-((2*S*,3*R*)-2-(4-Bromophenyl)-3-(3-formylfuran-2-yl)-3-phenylpropyl)-2-oxo-2*H*-chromene-3-carbonitrile minor – 3f

^{1}H NMR (700 MHz, CDCl$_3$)

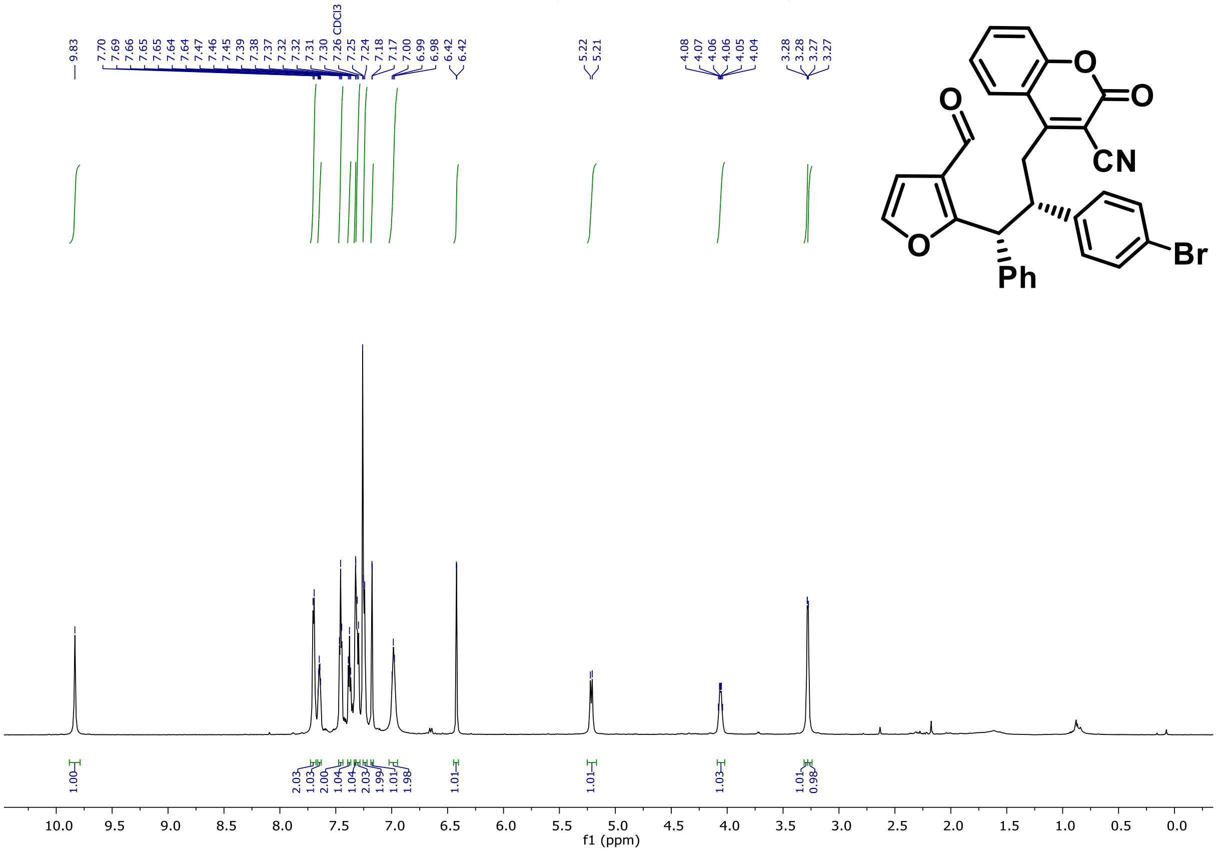

^{13}C{^{1}H} NMR (176 MHz, CDCl$_3$)

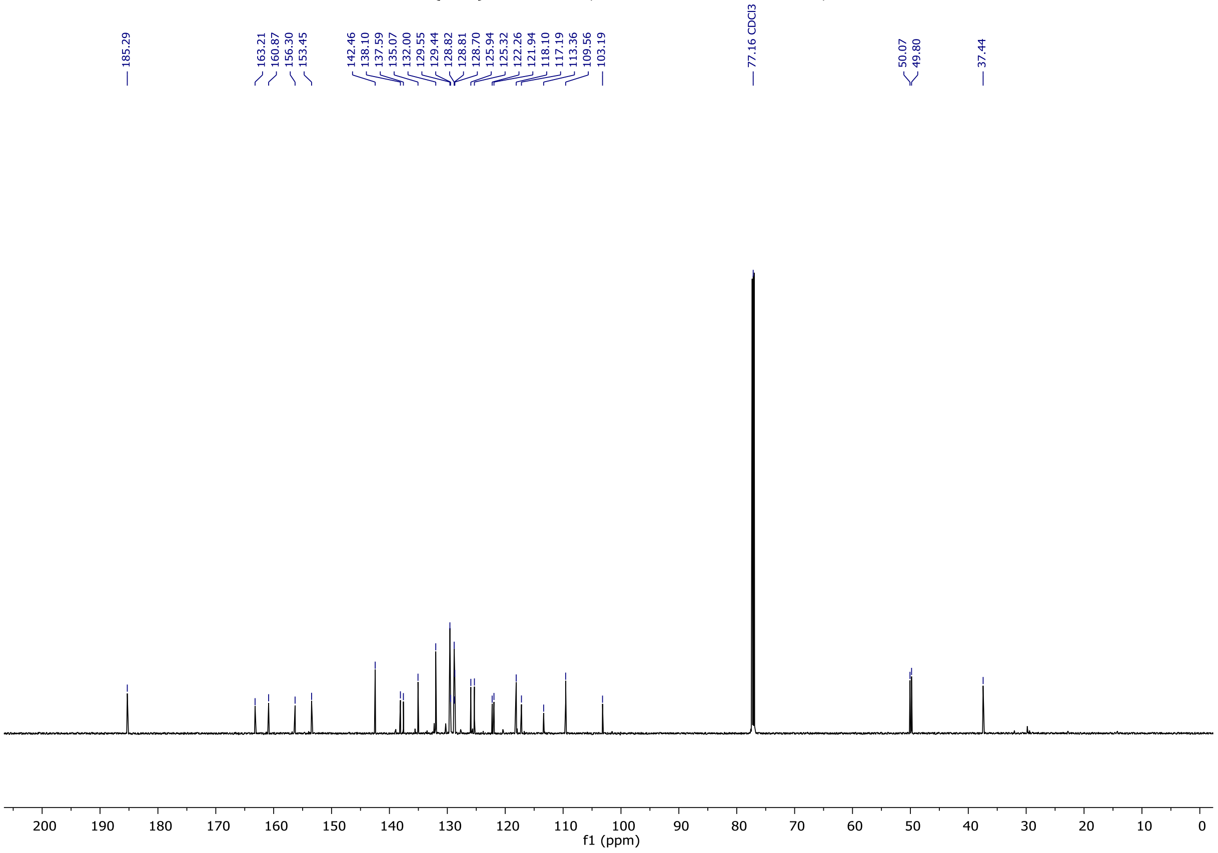

4-((2*R*,3*R*)-3-(3-Formylfuran-2-yl)-3-phenyl-2-(4-(trifluoromethyl)phenyl)propyl)-2-oxo-2*H*-chromene-3-carbonitrile major – 3g

^{1}H NMR (700 MHz, $CDCl_3$)

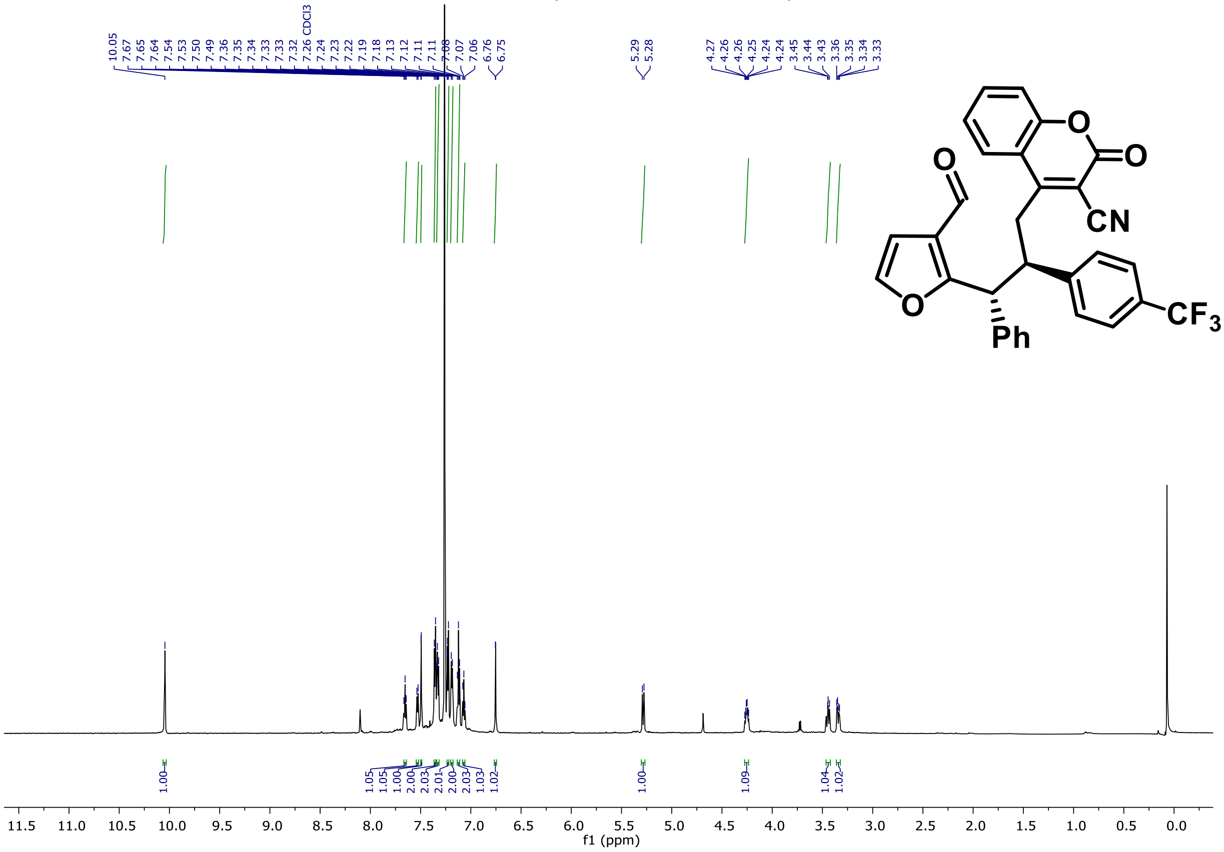

$^{13}C\{^{1}H\}$ NMR (176 MHz, $CDCl_3$)

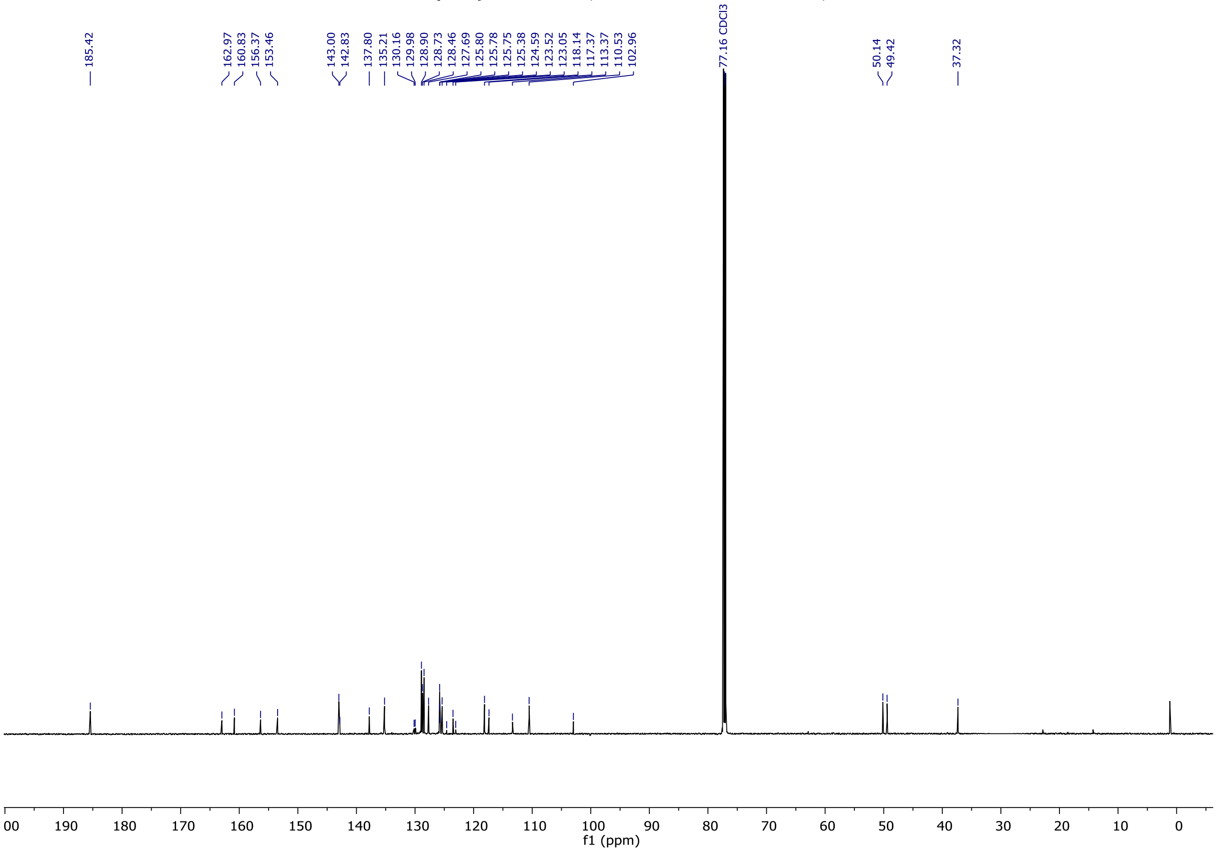

4-((2*S*,3*R*)-3-(3-Formylfuran-2-yl)-3-phenyl-2-(4-(trifluoromethyl)phenyl)propyl)-2-oxo-2*H*-chromene-3-carbonitrile minor – 3g

^{1}H NMR (700 MHz, CDCl$_3$)

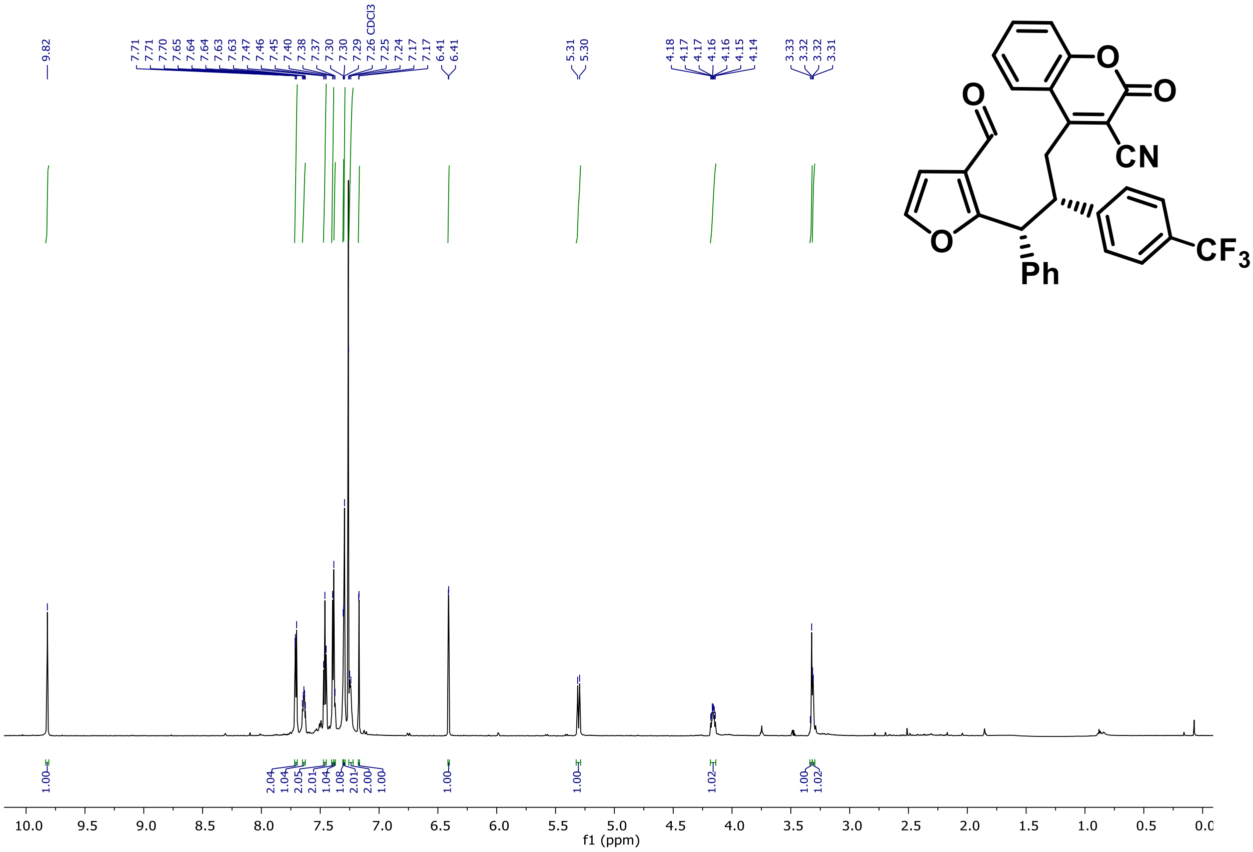

^{13}C{^{1}H} NMR (176 MHz, CDCl$_3$)

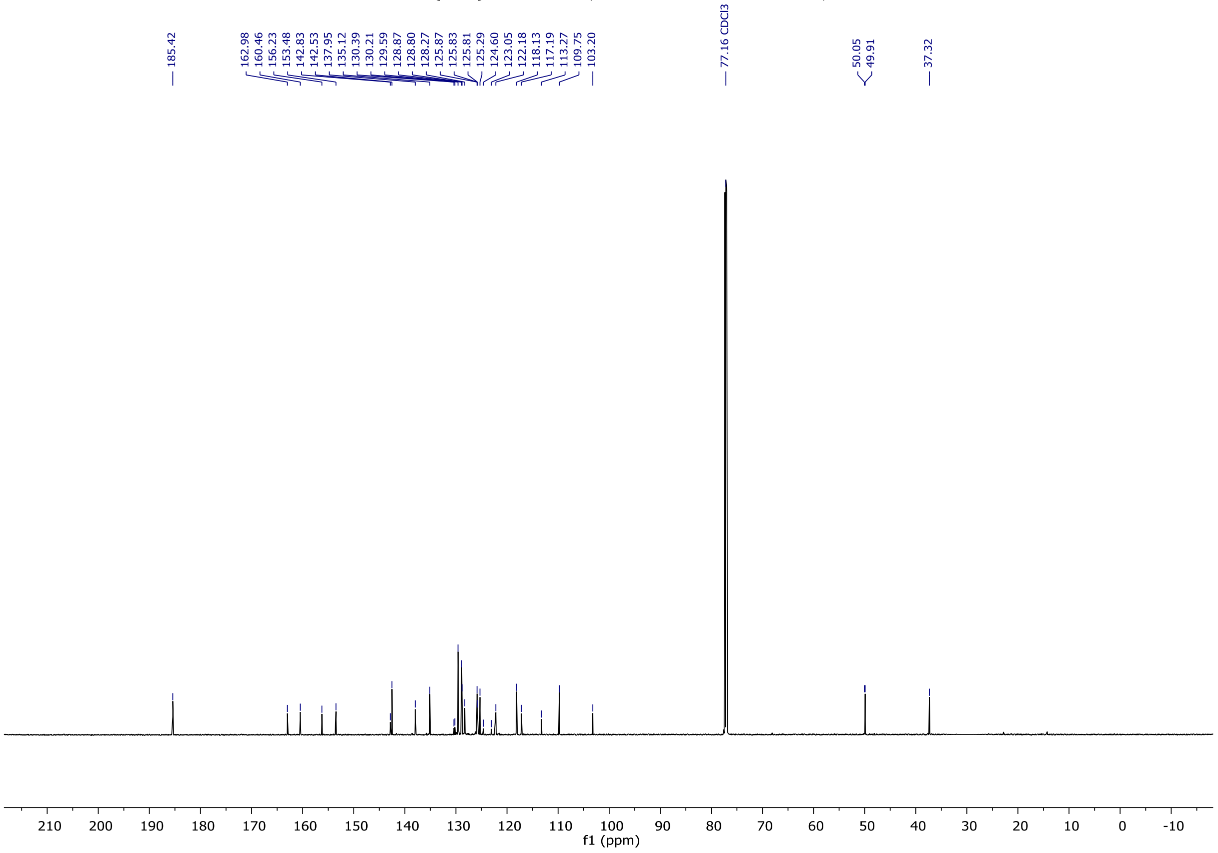

4-((2*R*,3*R*)-3-(3-Formylfuran-2-yl)-3-phenyl-2-(*m*-tolyl)propyl)-2-oxo-2*H*-chromene-3-carbonitrile major – 3h

^{1}H NMR (700 MHz, $CDCl_3$)

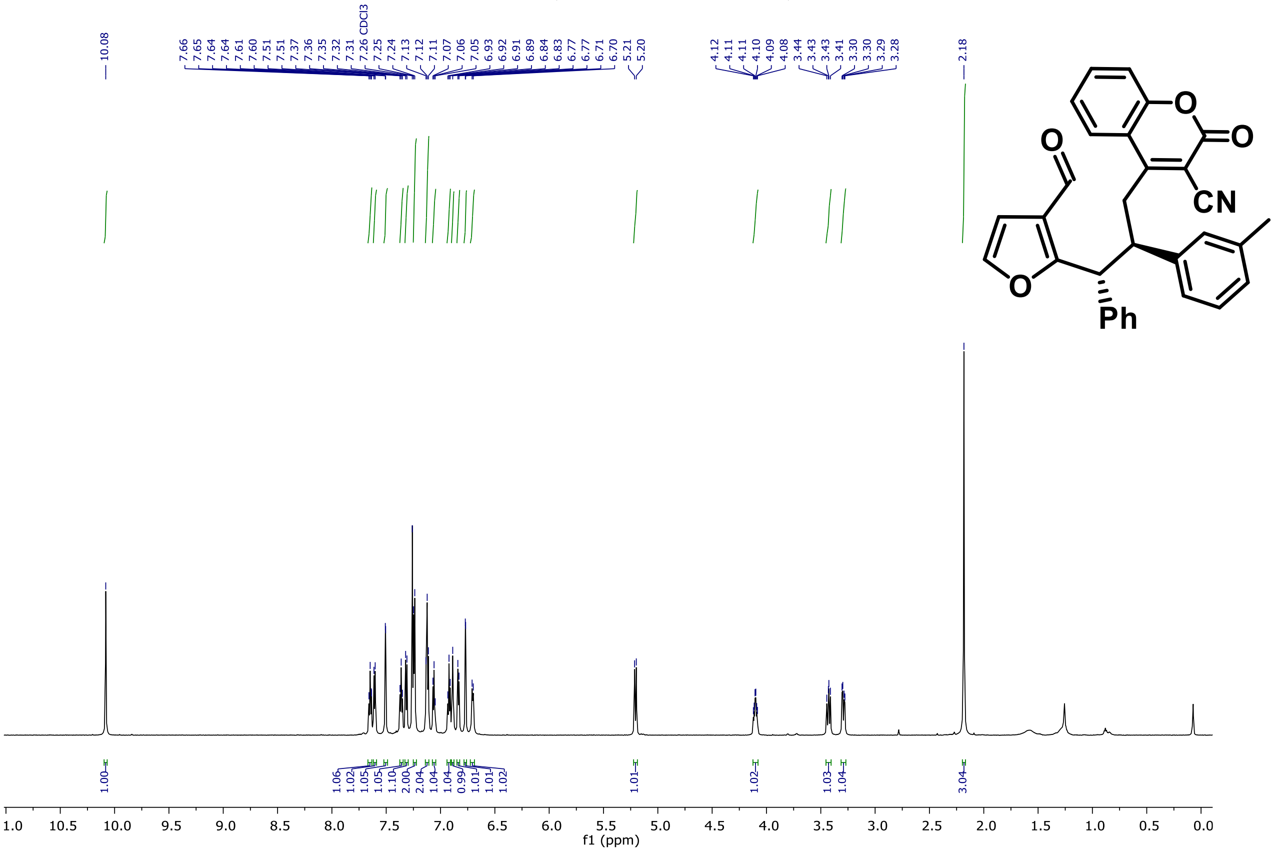

^{13}C{^{1}H} NMR (176 MHz, $CDCl_3$)

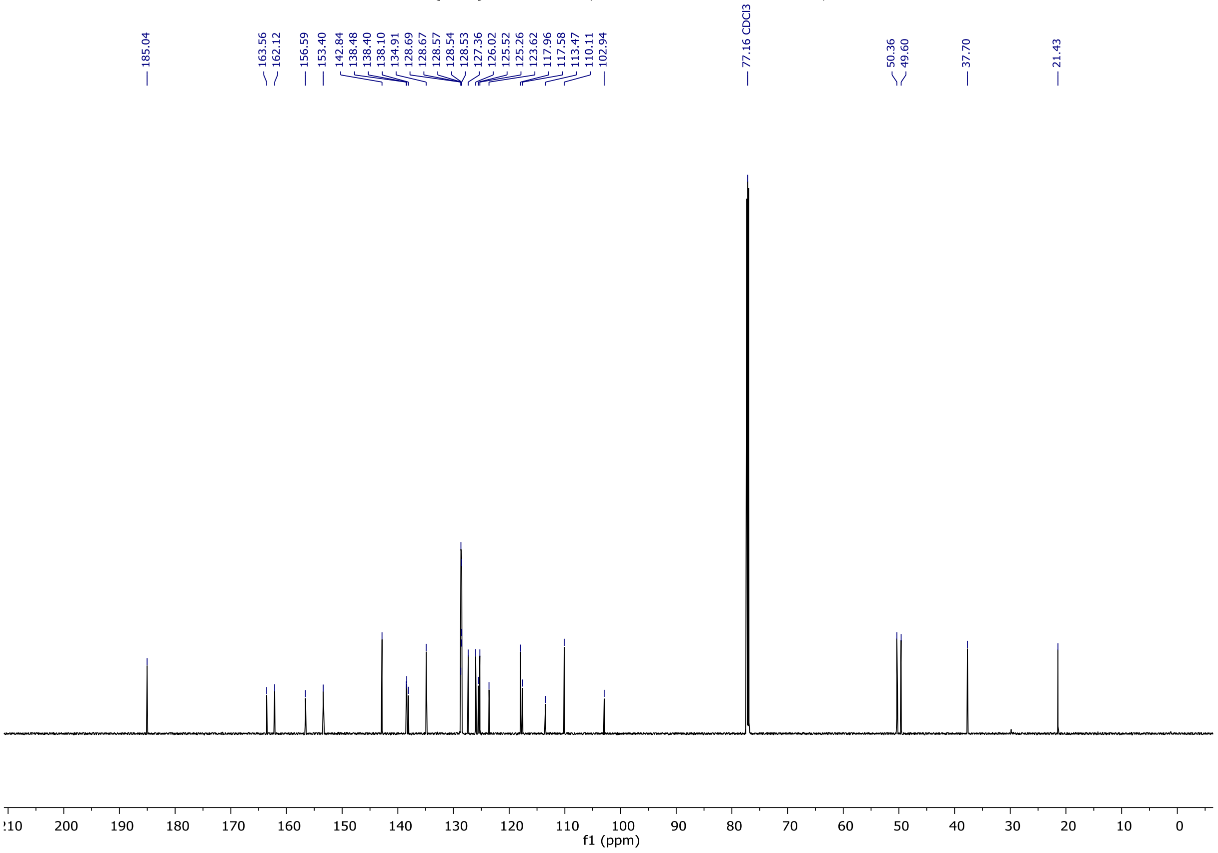

4-((2*S*,3*R*)-3-(3-Formylfuran-2-yl)-3-phenyl-2-(*m*-tolyl)propyl)-2-oxo-2*H*-chromene-3-carbonitrile minor – 3h

^{1}H NMR (700 MHz, $CDCl_3$)

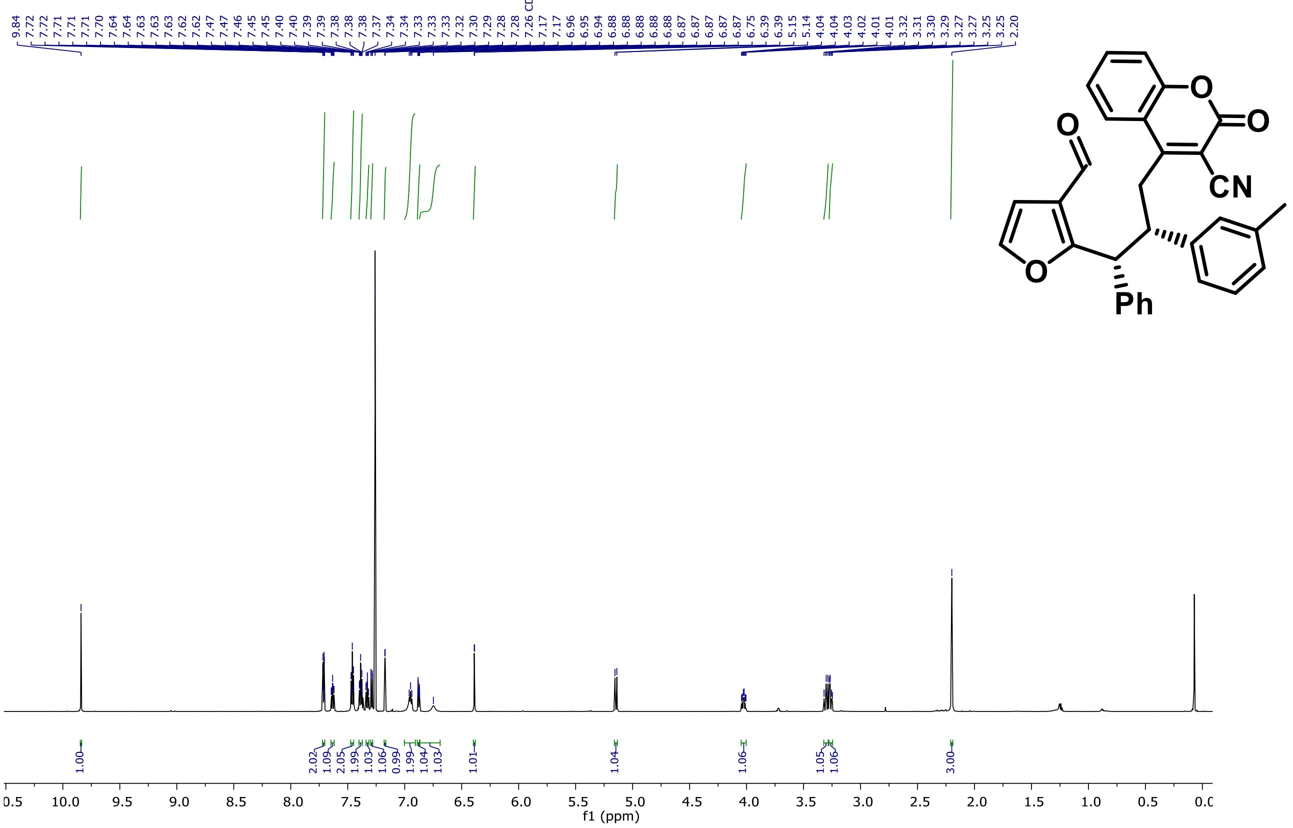

^{13}C{^{1}H} NMR (176 MHz, $CDCl_3$)

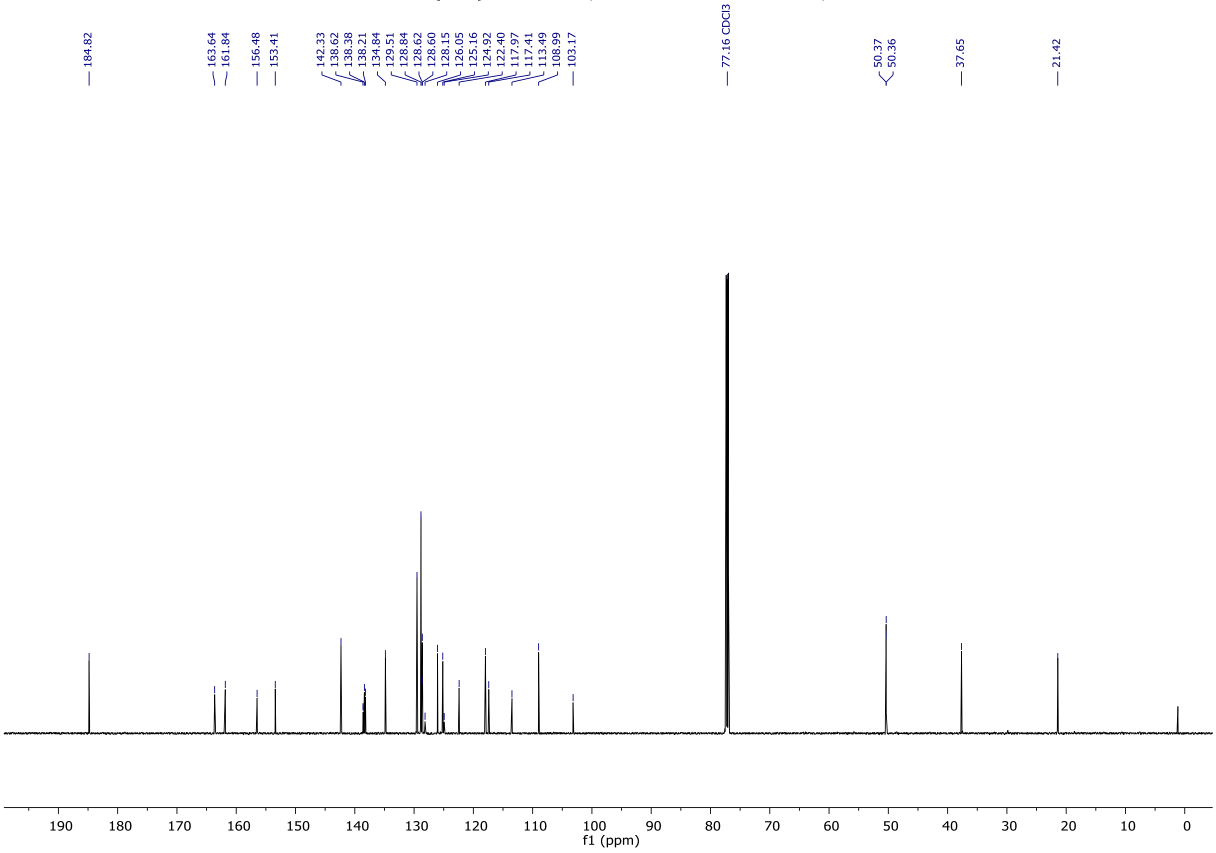

4-((2*R*,3*R*)-3-(3-Formylfuran-2-yl)-3-phenyl-2-(*p*-tolyl)propyl)-2-oxo-2*H*-chromene-3-carbonitrile major – 3i

^{1}H NMR (700 MHz, $CDCl_3$)

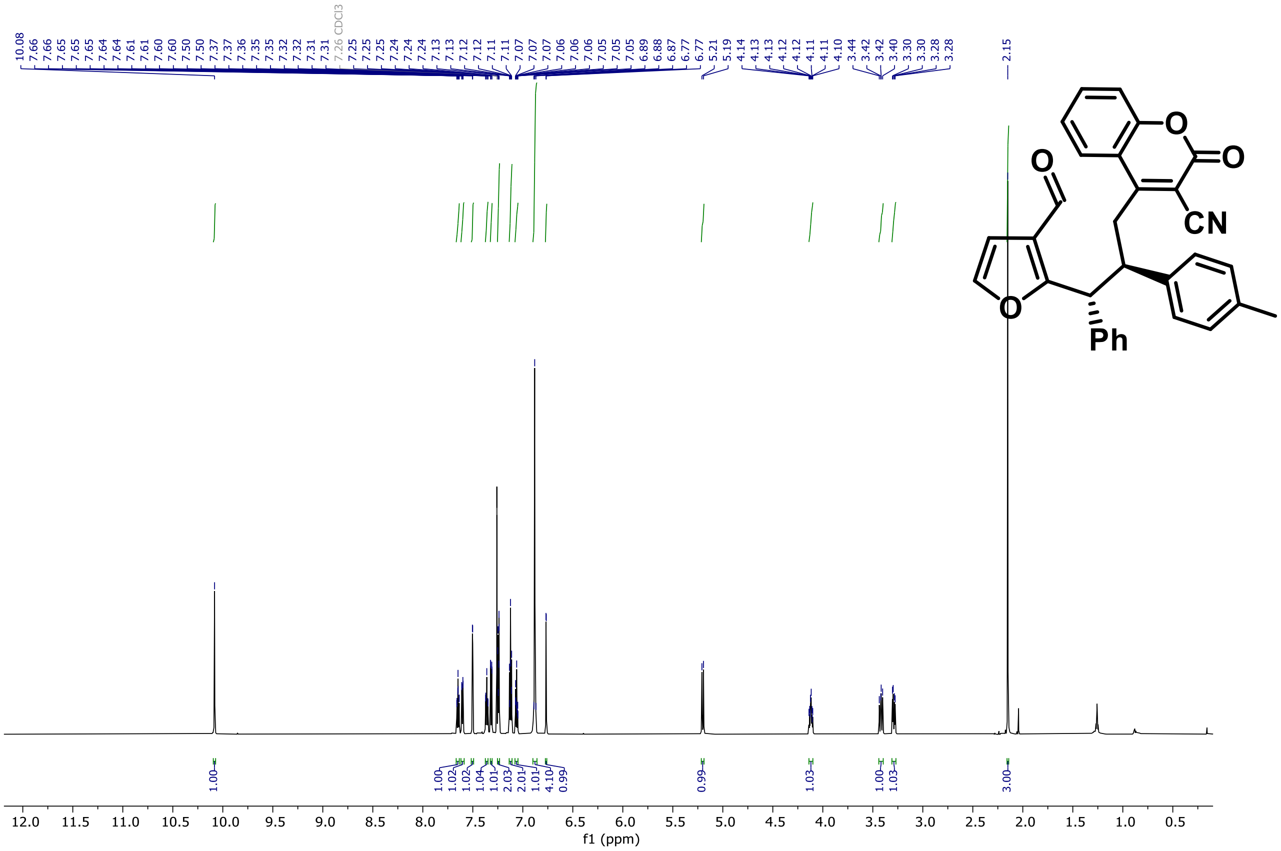

$^{13}C\{^{1}H\}$ NMR (176 MHz, $CDCl_3$)

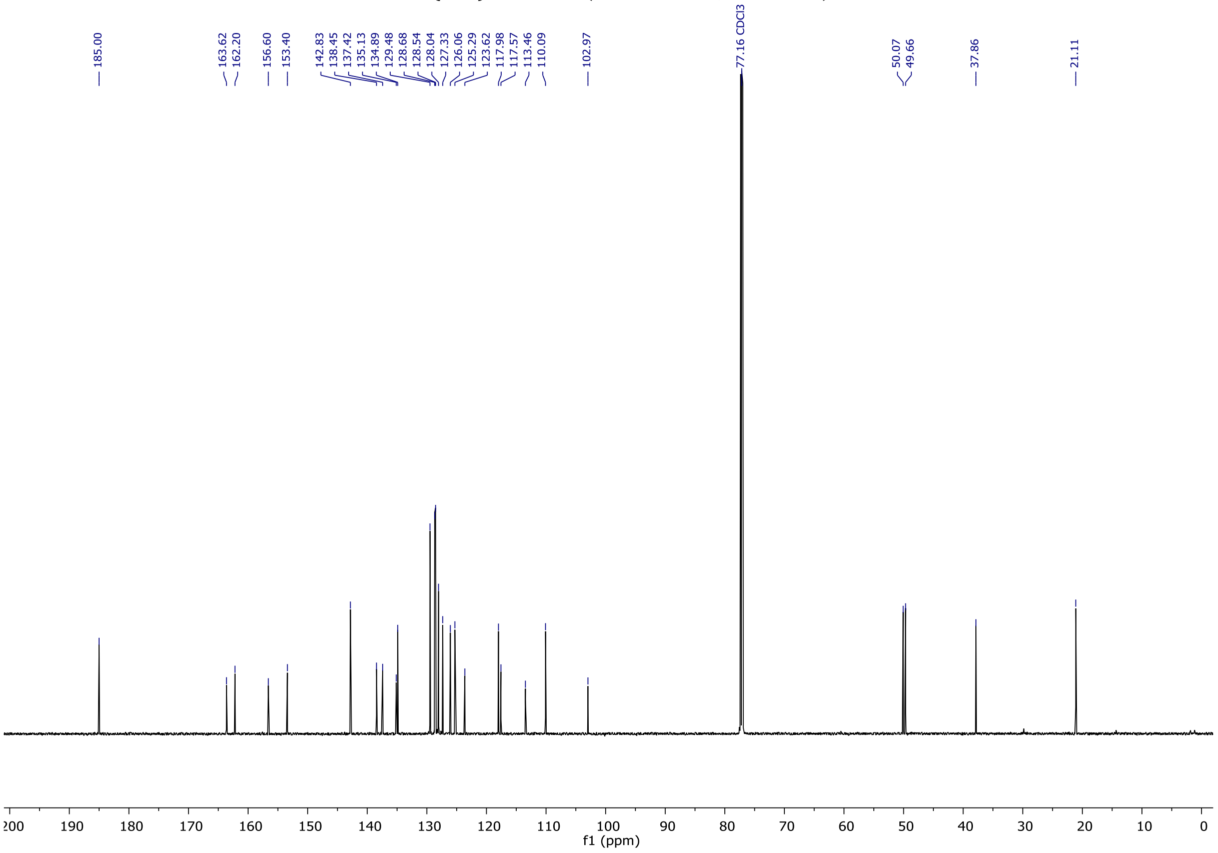

4-((2*S*,3*R*)-3-(3-Formylfuran-2-yl)-3-phenyl-2-(*p*-tolyl)propyl)-2-oxo-2*H*-chromene-3-carbonitrile minor – 3i

^{1}H NMR (700 MHz, CDCl$_3$)

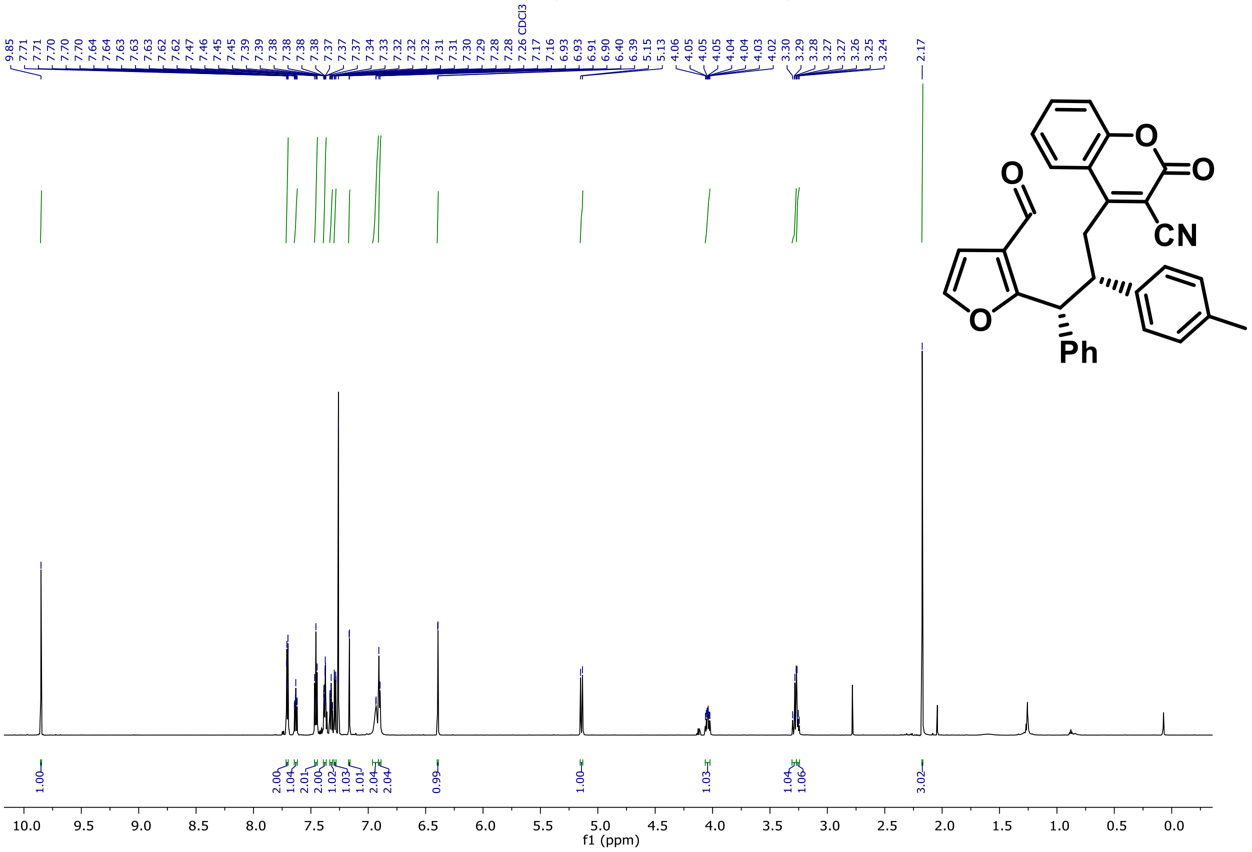

^{13}C{^{1}H} NMR (176 MHz, CDCl$_3$)

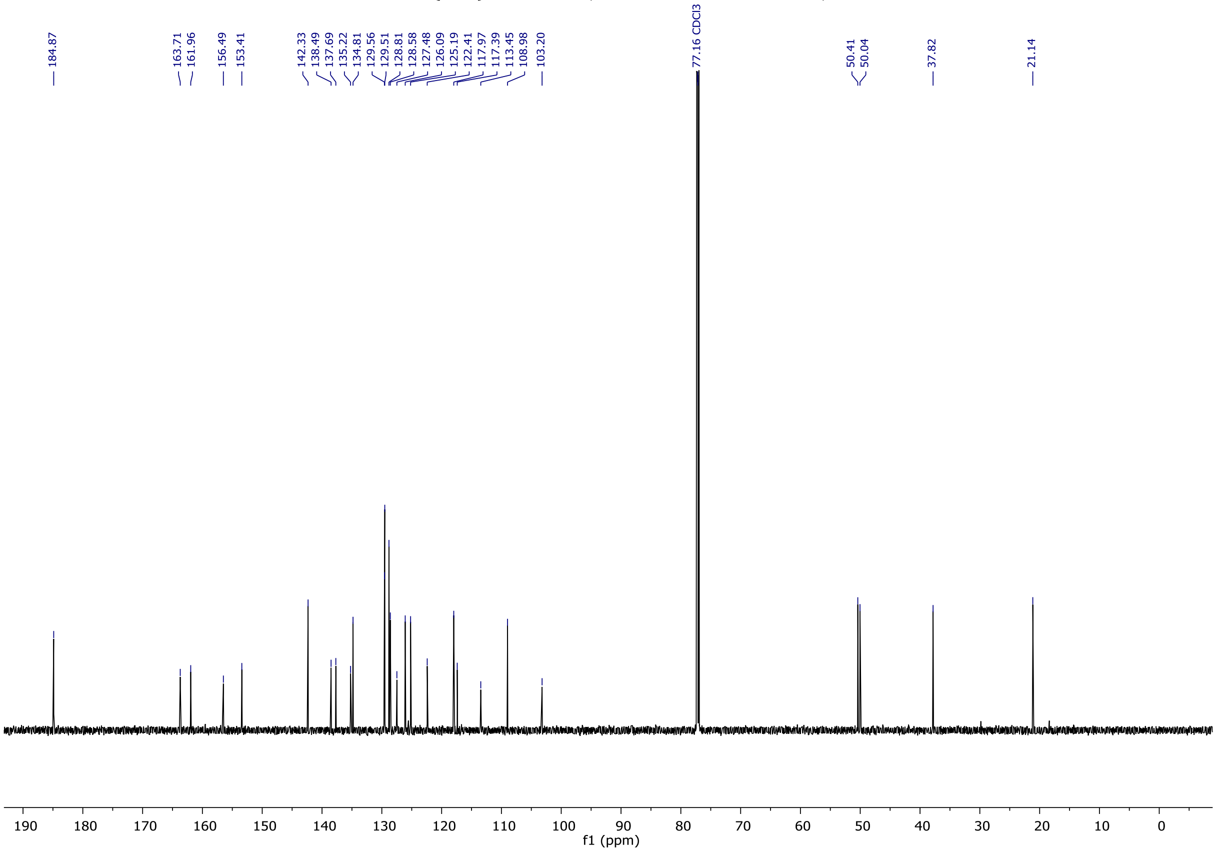

4-((2*R*,3*R*)-3-(3-Formylfuran-2-yl)-2-(4-methoxyphenyl)-3-phenylpropyl)-2-oxo-2*H*-chromene-3-carbonitrile major – 3j

^{1}H NMR (700 MHz, $CDCl_3$)

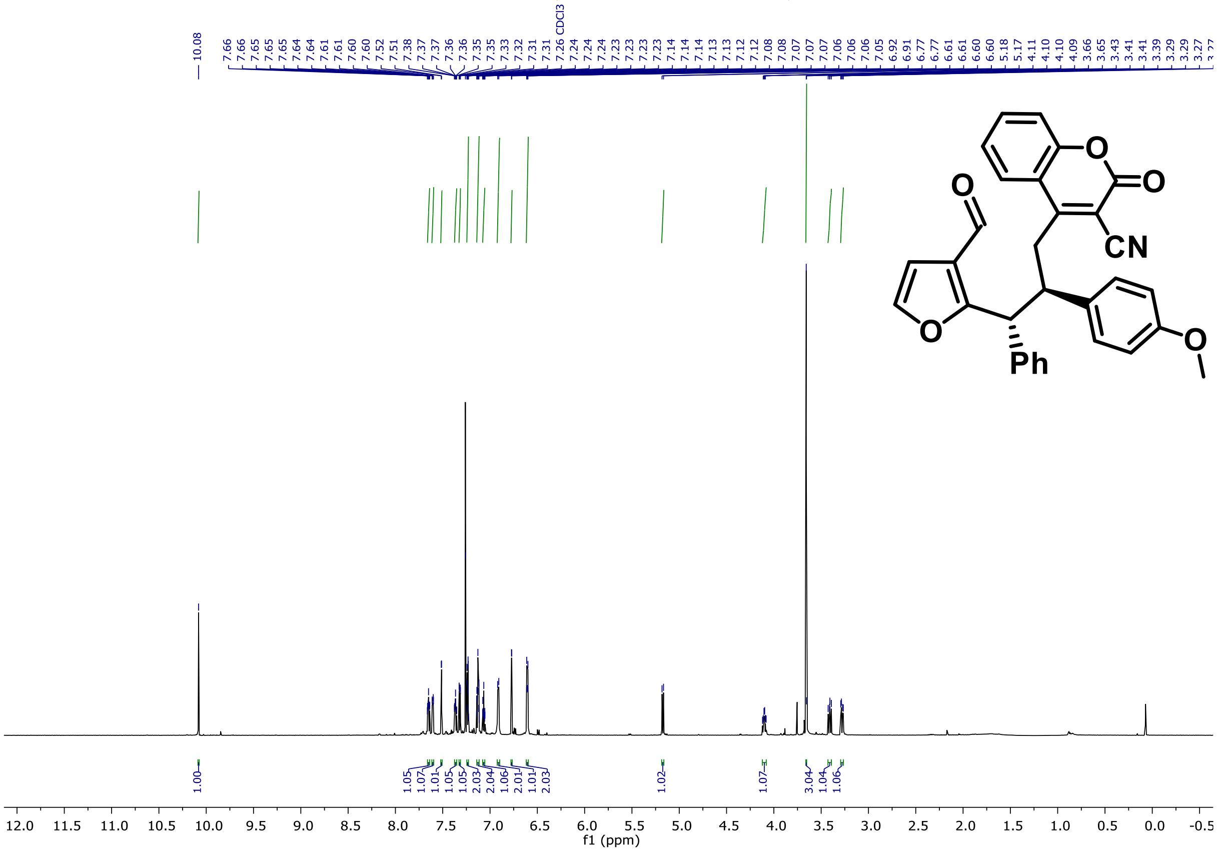

^{13}C{^{1}H} NMR (176 MHz, $CDCl_3$)

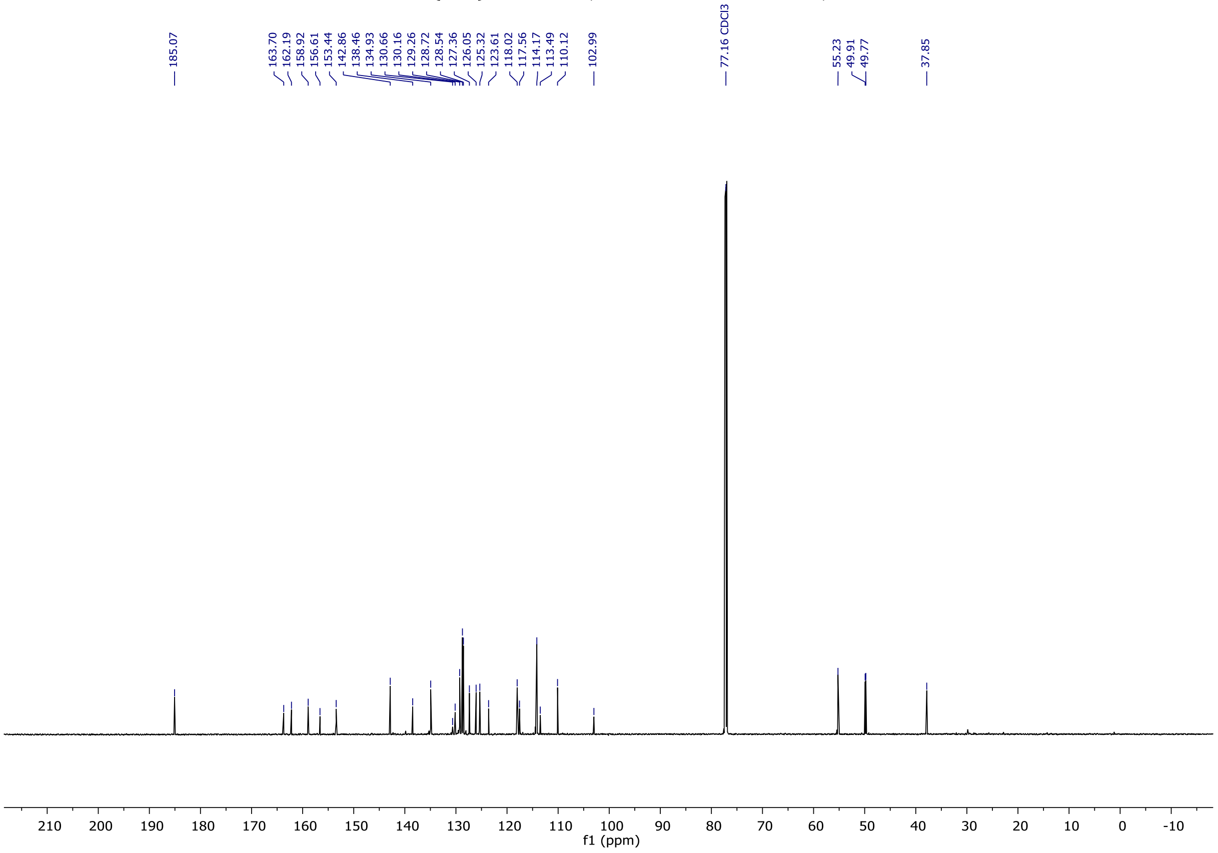

4-((2*S*,3*R*)-3-(3-Formylfuran-2-yl)-2-(4-methoxyphenyl)-3-phenylpropyl)-2-oxo-2*H*-chromene-3-carbonitrile minor – 3j

^{1}H NMR (700 MHz, CDCl$_3$)

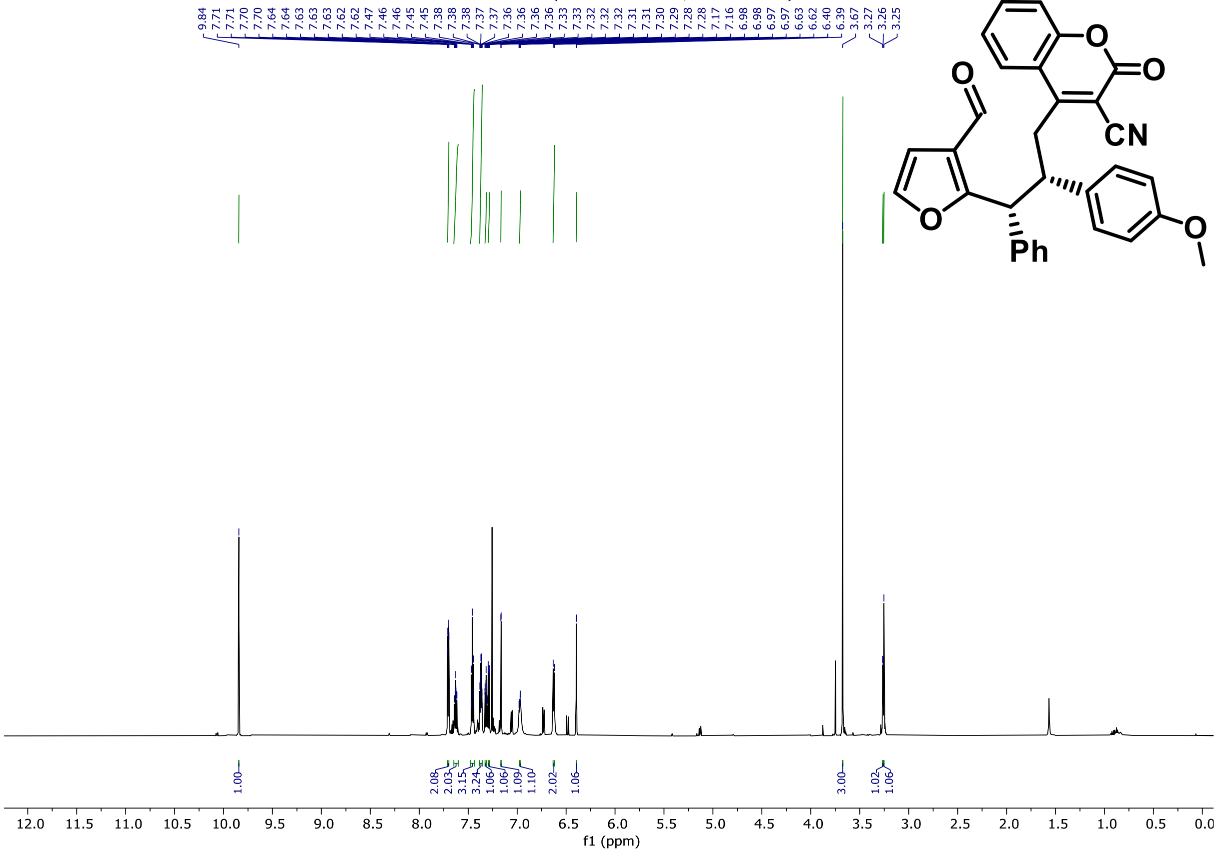

^{13}C{^{1}H} NMR (176 MHz, CDCl$_3$)

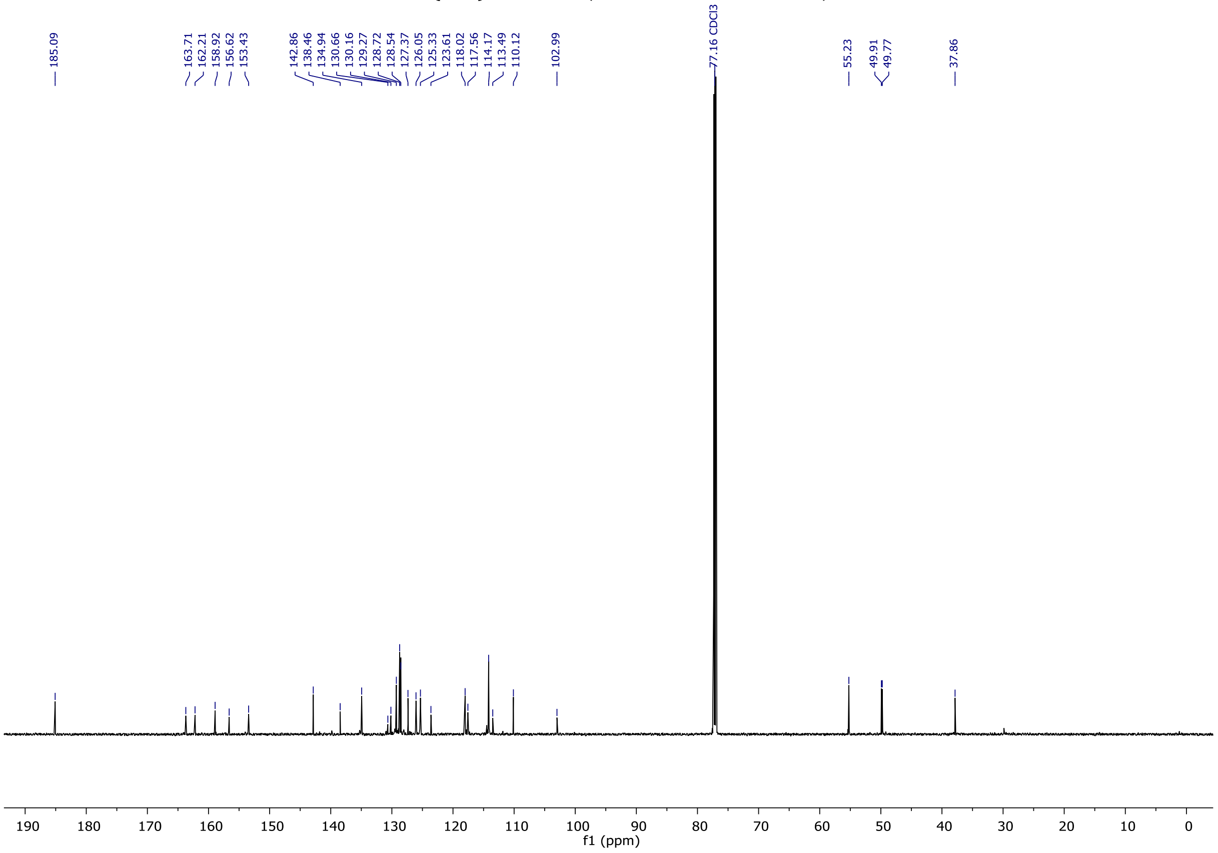

4-((2*R*,3*R*)-3-(3-Formylfuran-2-yl)-2-(naphthalen-2-yl)-3-phenylpropyl)-2-oxo-2*H*-chromene-3-carbonitrile major – 3k

^{1}H NMR (700 MHz, CDCl$_3$)

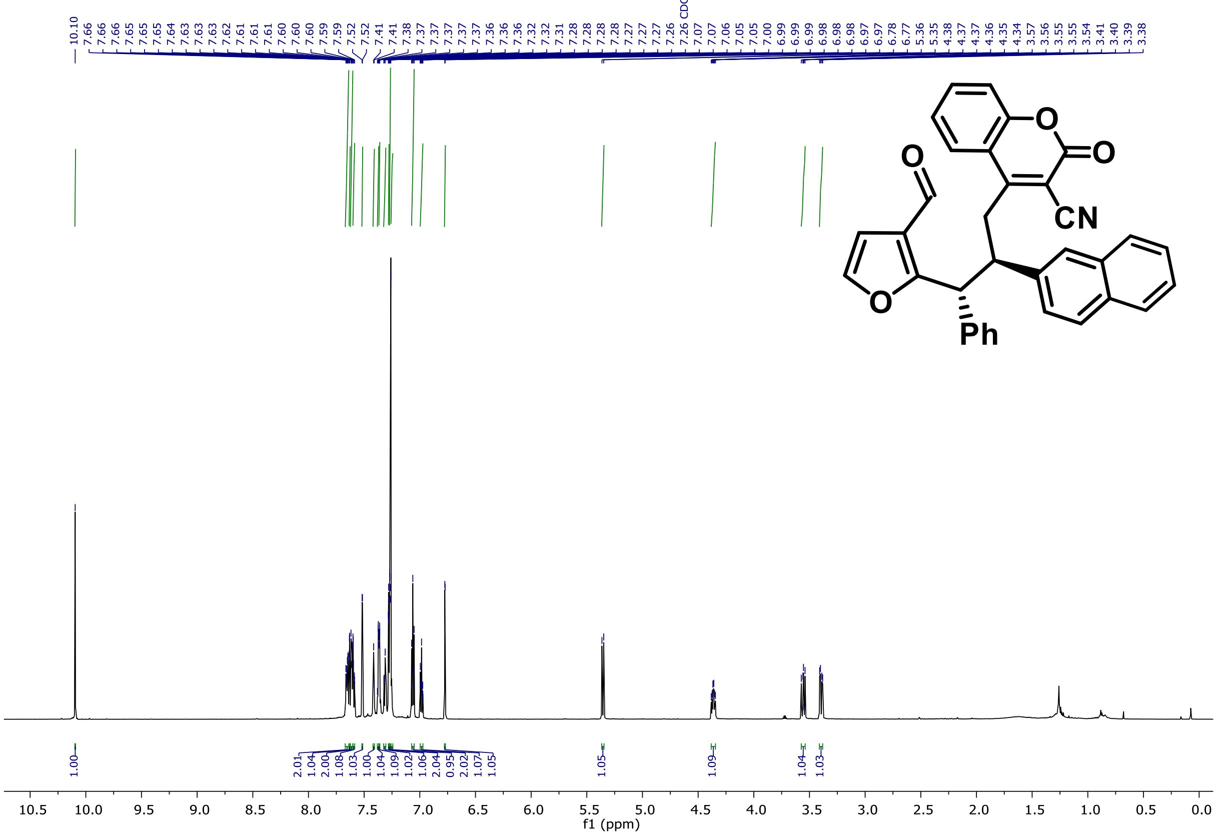

^{13}C{^{1}H} NMR (176 MHz, CDCl$_3$)

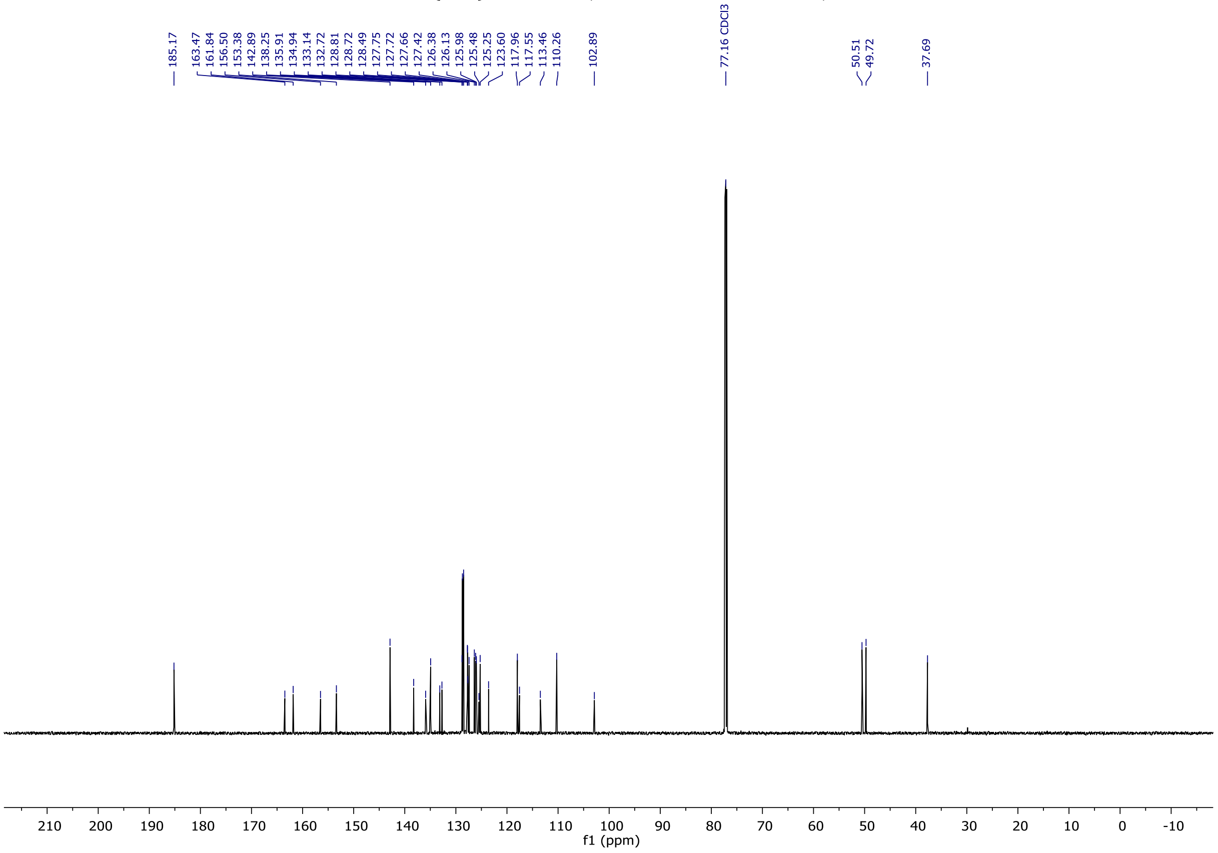

4-((2*S*,3*R*)-3-(3-Formylfuran-2-yl)-2-(naphthalen-2-yl)-3-phenylpropyl)-2-oxo-2*H*-chromene-3-carbonitrile minor – 3k

^{1}H NMR (700 MHz, CDCl$_3$)

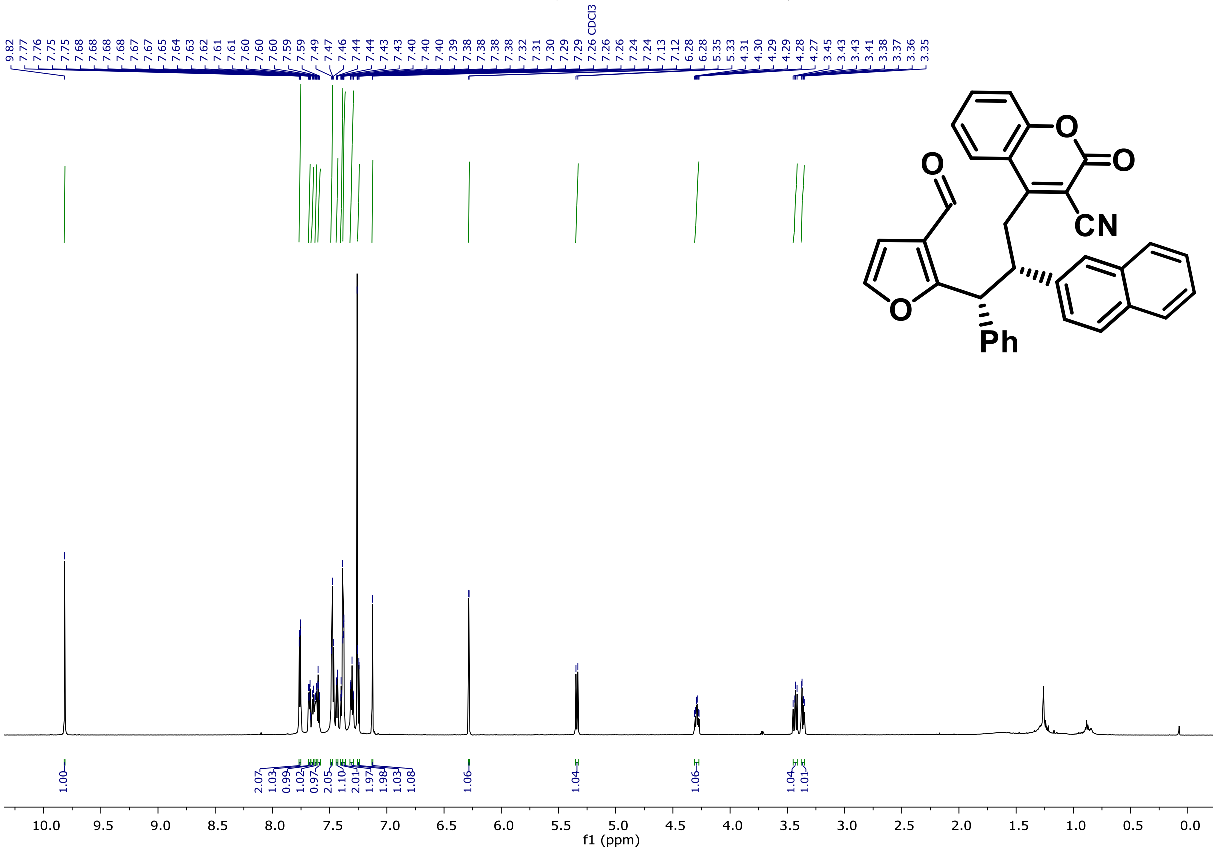

^{13}C{^{1}H} NMR (176 MHz, CDCl$_3$)

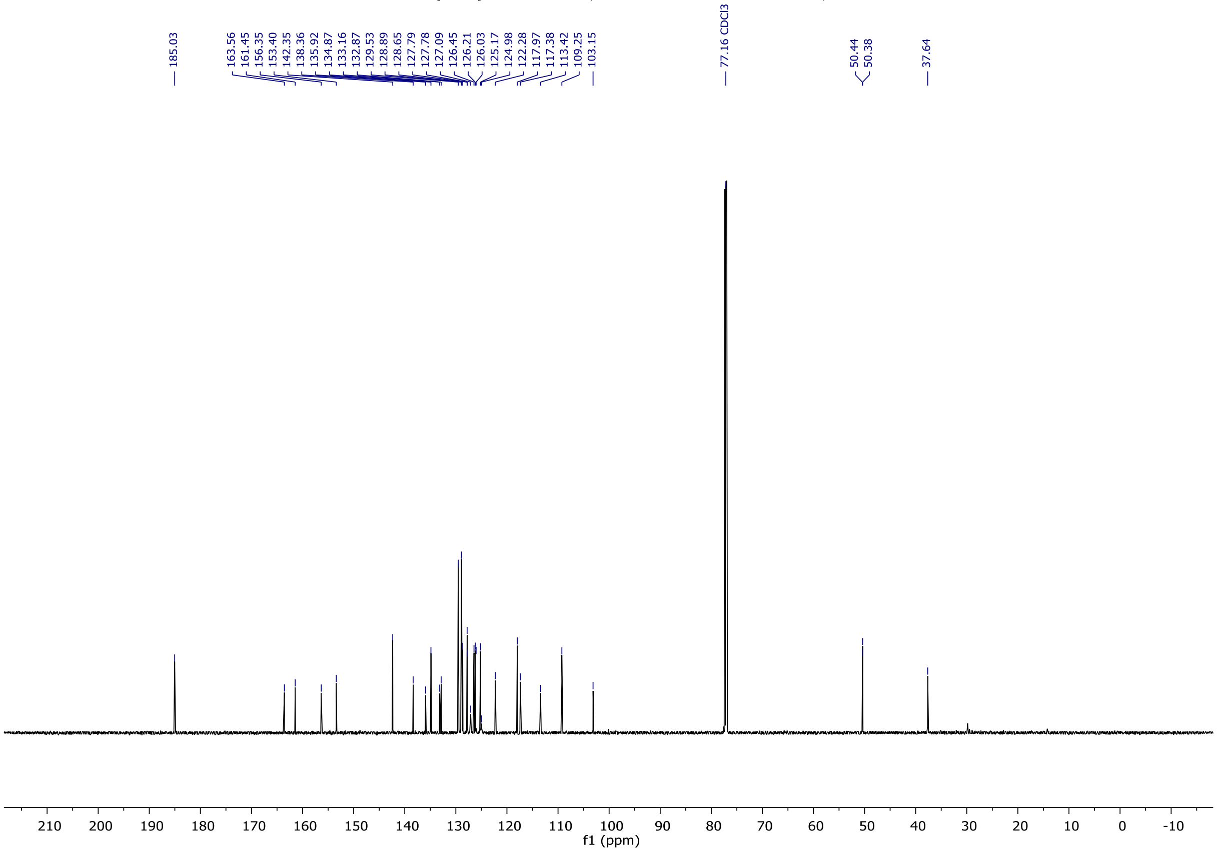

6-Bromo-4-((2*R*,3*R*)-3-(3-formylfuran-2-yl)-2,3-diphenylpropyl)-2-oxo-2*H*-chromene-3-carbonitrile major – 3l

^{1}H NMR (700 MHz, $CDCl_3$)

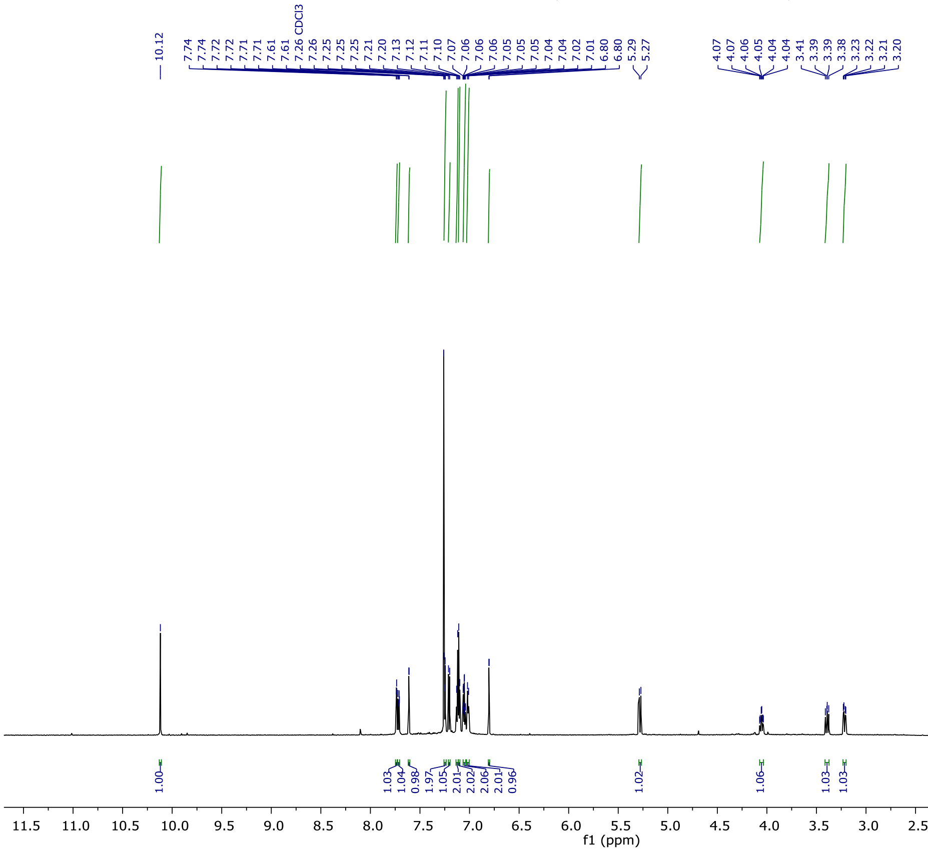

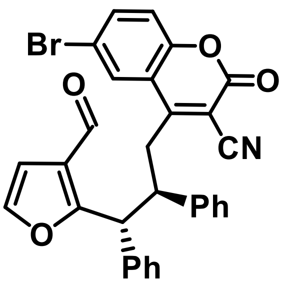

$^{13}C\{^1H\}$ NMR (176 MHz, $CDCl_3$)

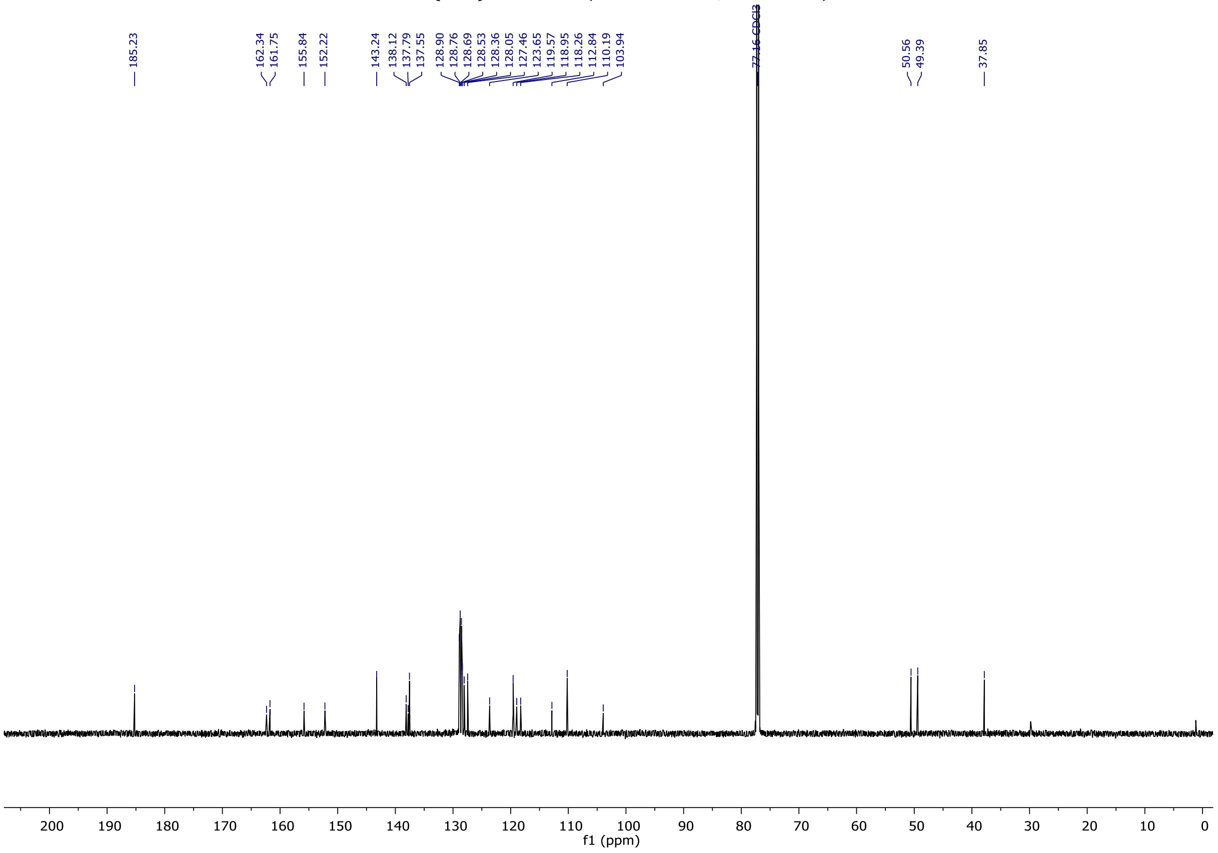

6-Bromo-4-((2*S*,3*R*)-3-(3-formylfuran-2-yl)-2,3-diphenylpropyl)-2-oxo-2*H*-chromene-3-carbonitrile minor – 3l

^{1}H NMR (700 MHz, $CDCl_3$)

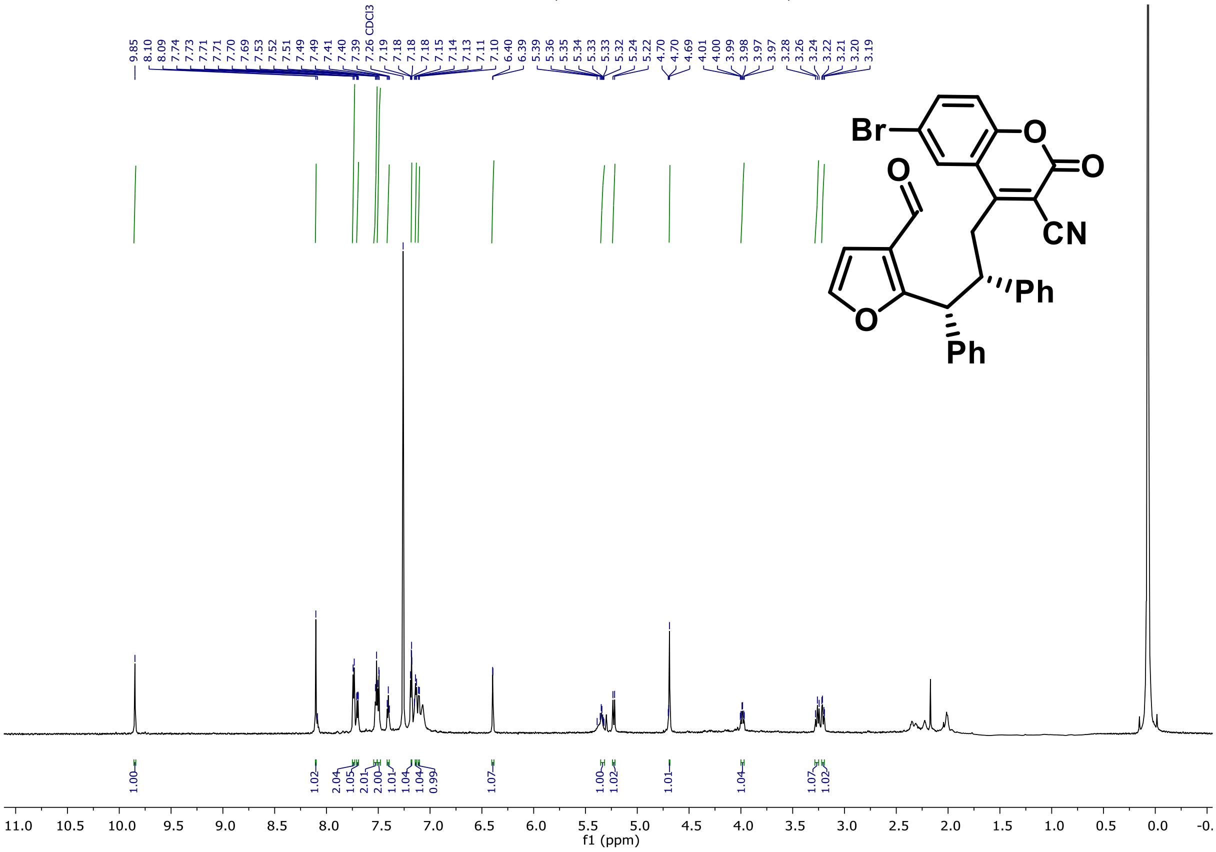

^{13}C{^{1}H} NMR (176 MHz, $CDCl_3$)

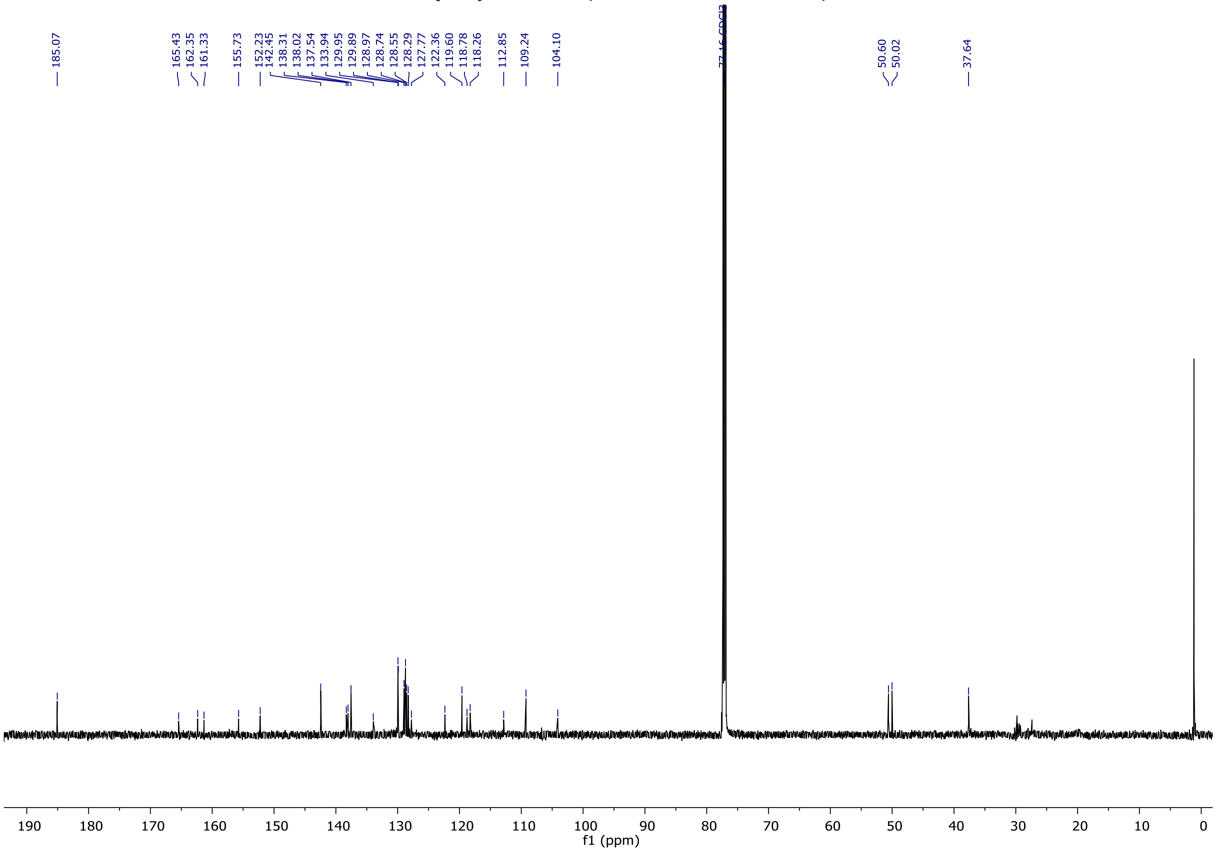

4-((2*R*,3*R*)-3-(3-Formylfuran-2-yl)-2,3-diphenylpropyl)-7-methoxy-2-oxo-2*H*-chromene-3-carbonitrile major – 3m

^{1}H NMR (700 MHz, CDCl$_3$)

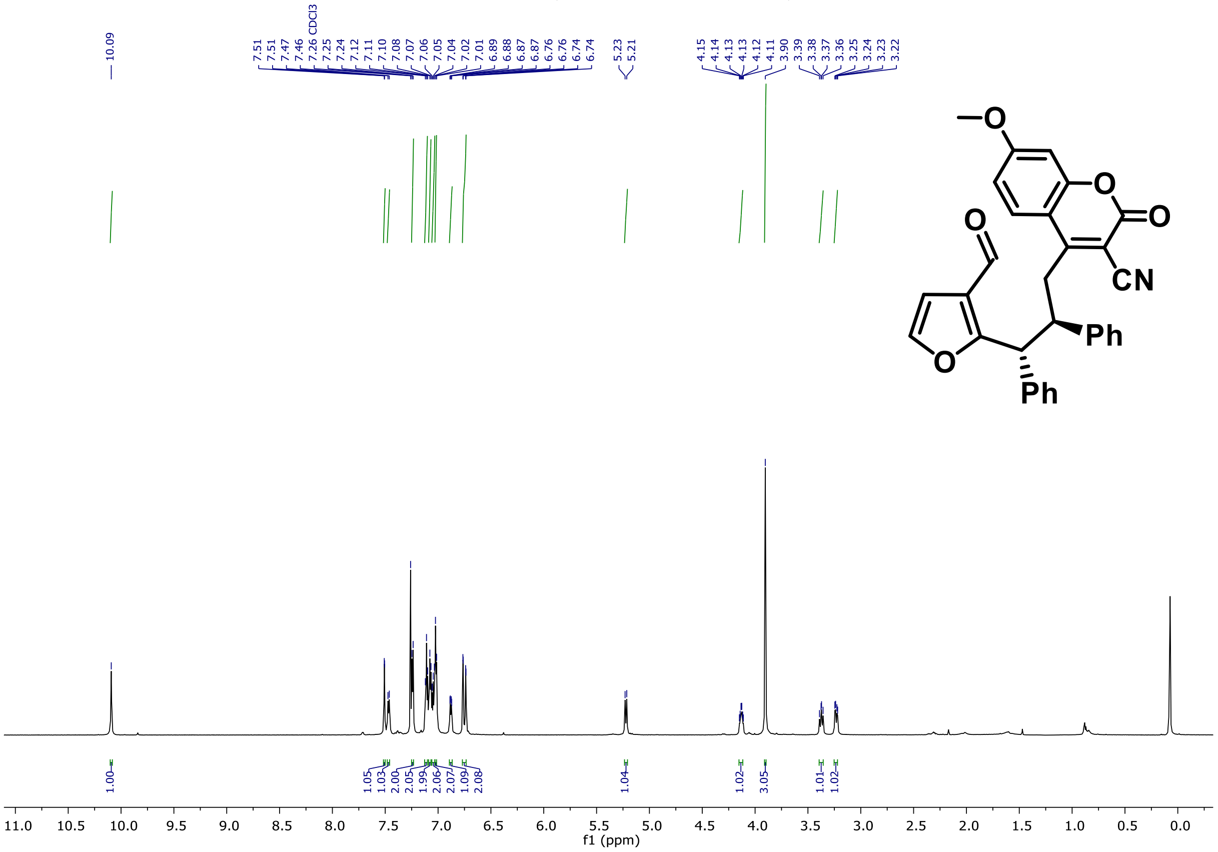

^{13}C{^{1}H} NMR (176 MHz, CDCl$_3$)

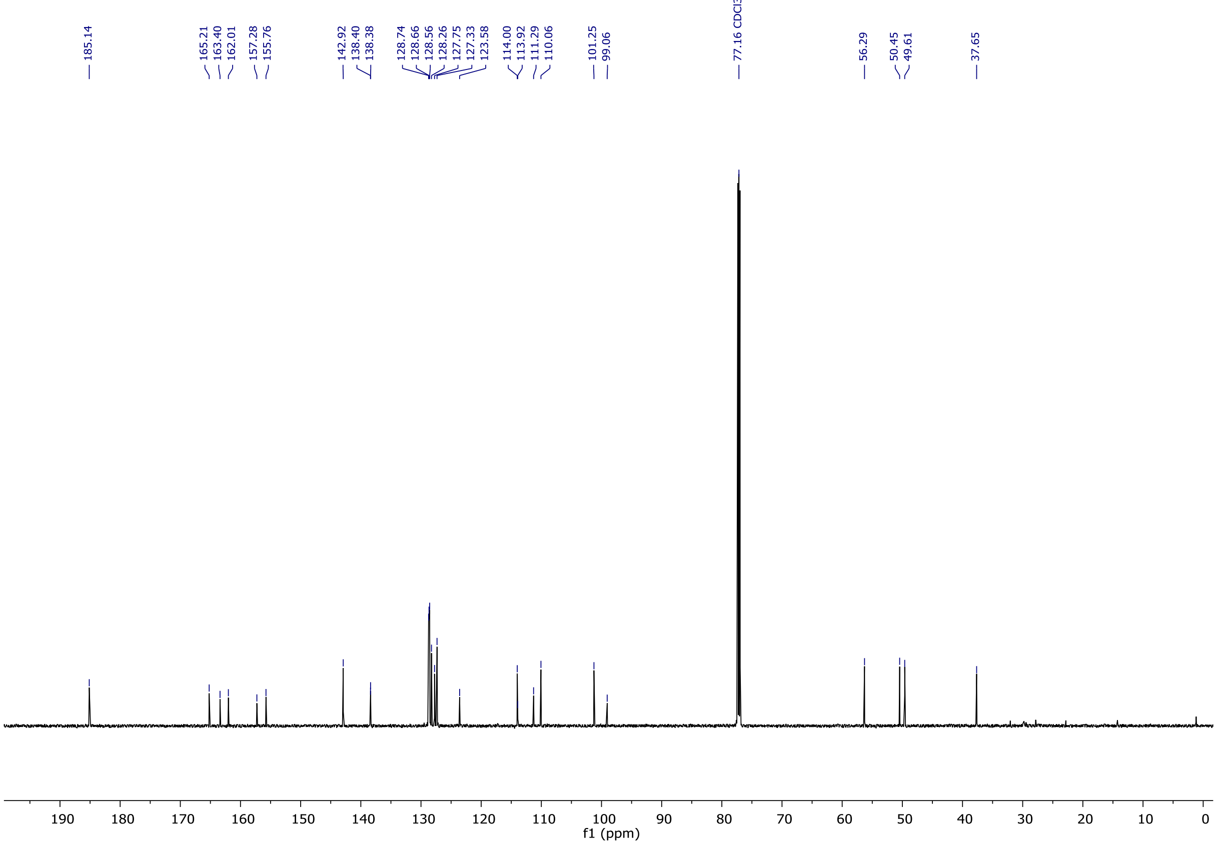

4-((2S,3R)-3-(3-Formylfuran-2-yl)-2,3-diphenylpropyl)-7-methoxy-2-oxo-2*H*-chromene-3-carbonitrile minor – 3m

^{1}H NMR (700 MHz, CDCl$_3$)

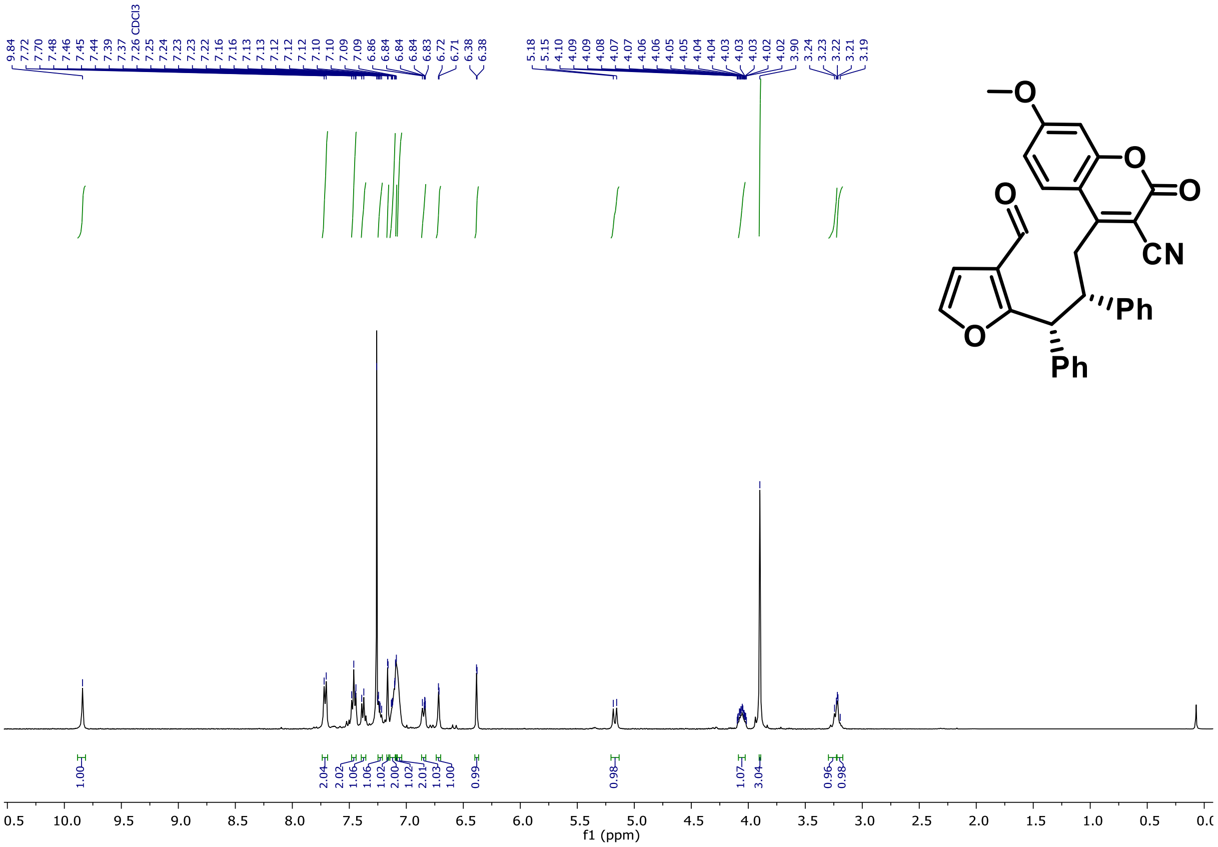

^{13}C{^{1}H} NMR (176 MHz, CDCl$_3$)

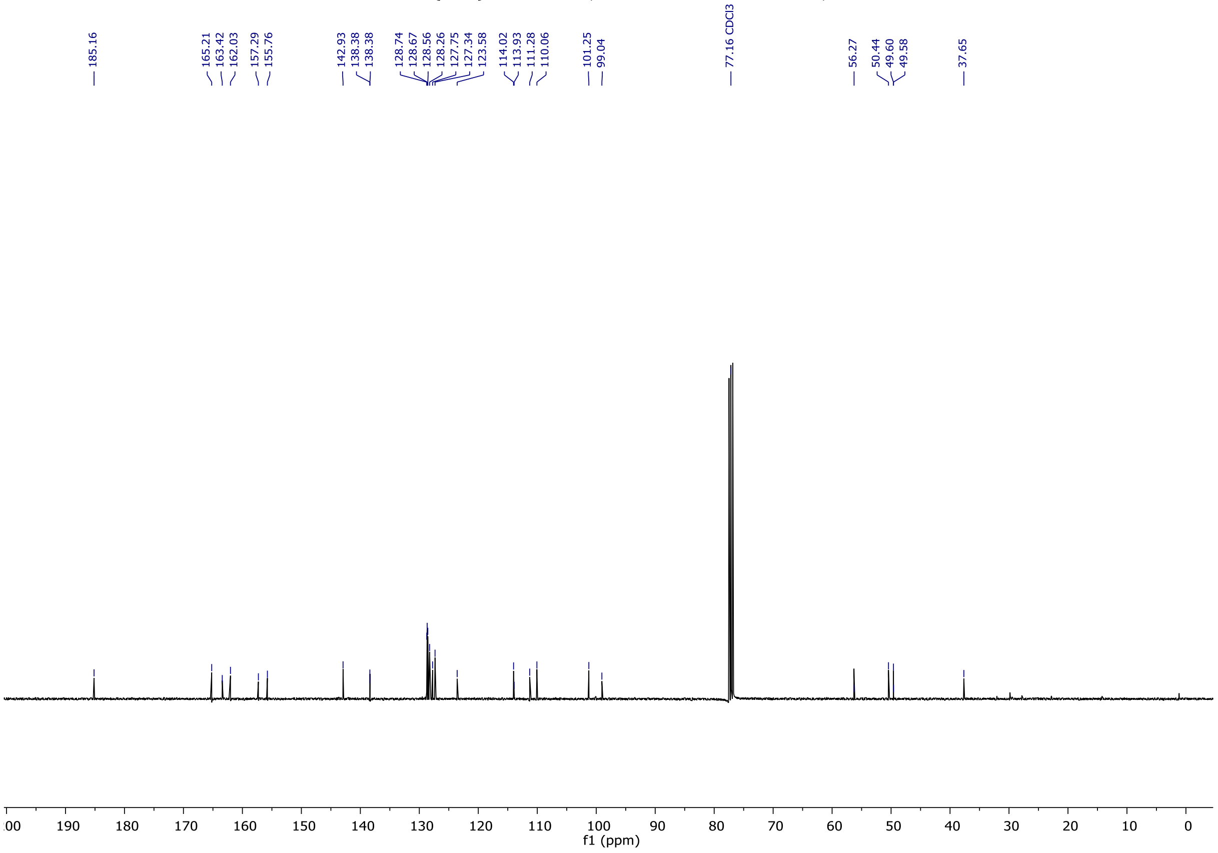

4-((*R*)-2-((*R*)-(3-formylfuran-2-yl)(phenyl)methyl)hexyl)-2-oxo-*2H*-chromene-3-carbonitrile major – 3n

^{1}H NMR (700 MHz, CDCl$_3$)

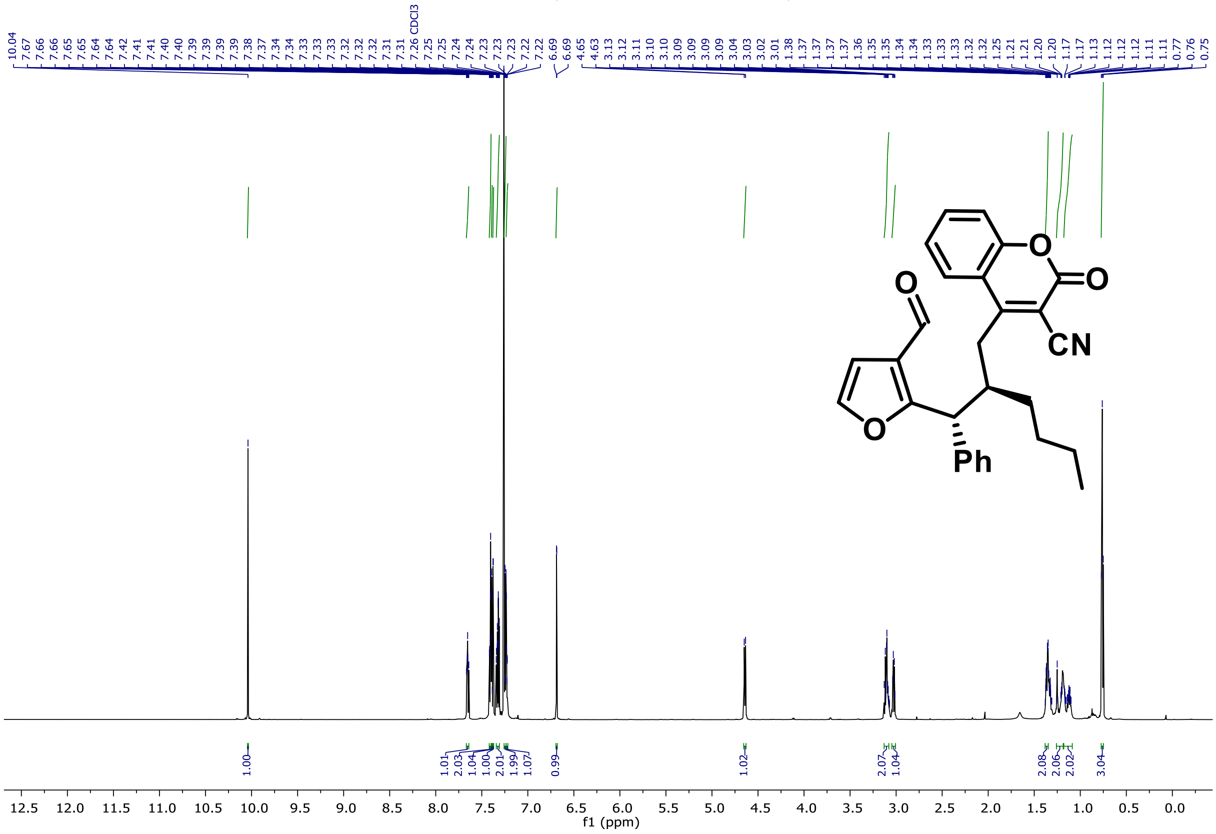

^{13}C{^{1}H} NMR (176 MHz, CDCl$_3$)

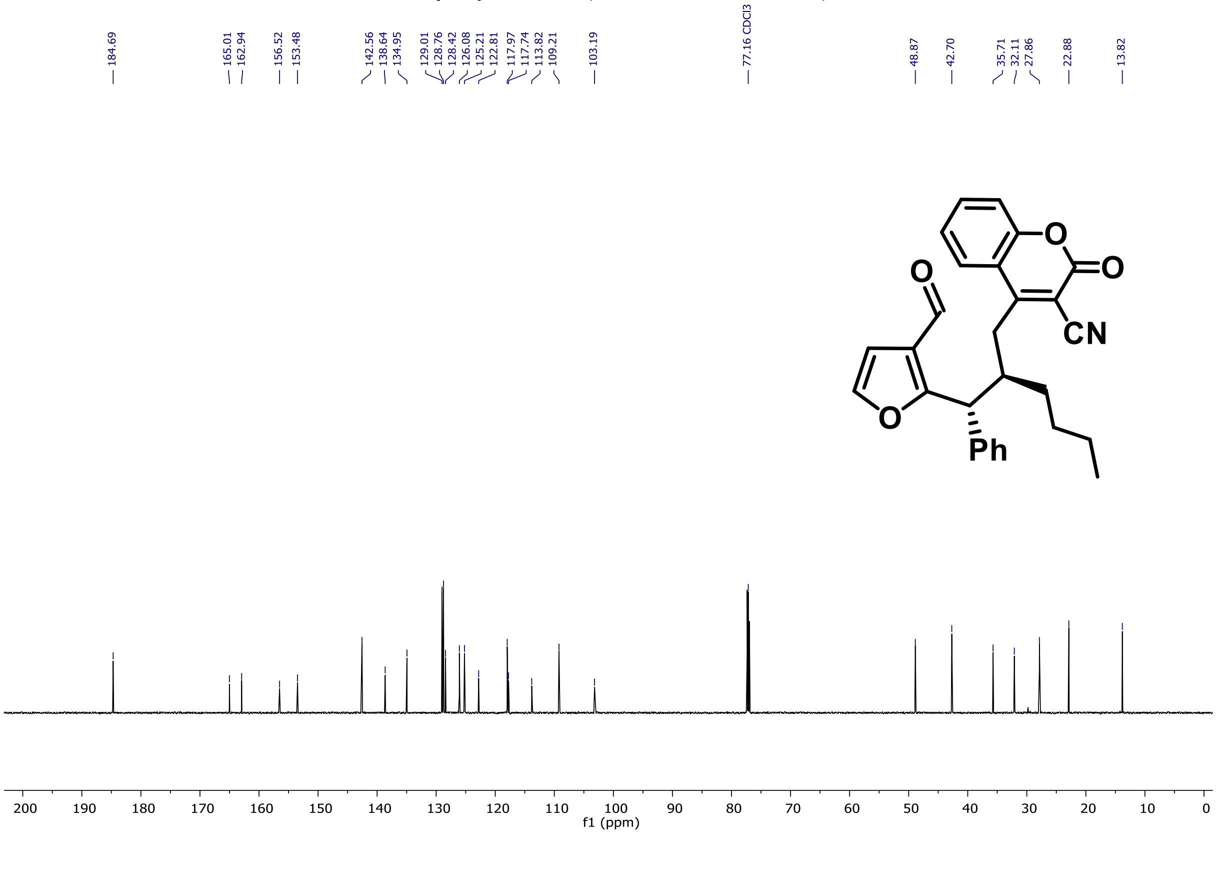

4-((S)-2-((R)-(3-formylfuran-2-yl)(phenyl)methyl)hexyl)-2-oxo-2H-chromene-3-carbonitrile minor – 3n

^{1}H NMR (700 MHz, $CDCl_3$)

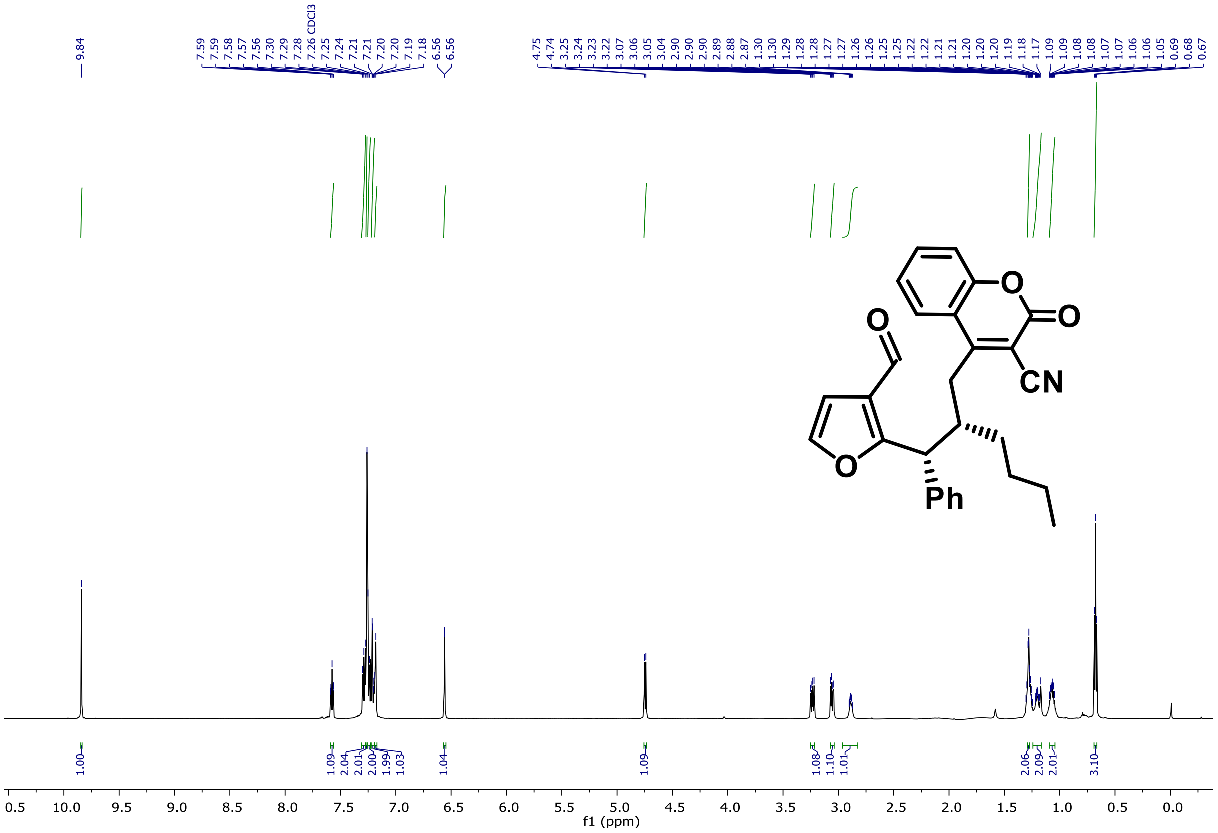

^{13}C{^{1}H} NMR (176 MHz, $CDCl_3$)

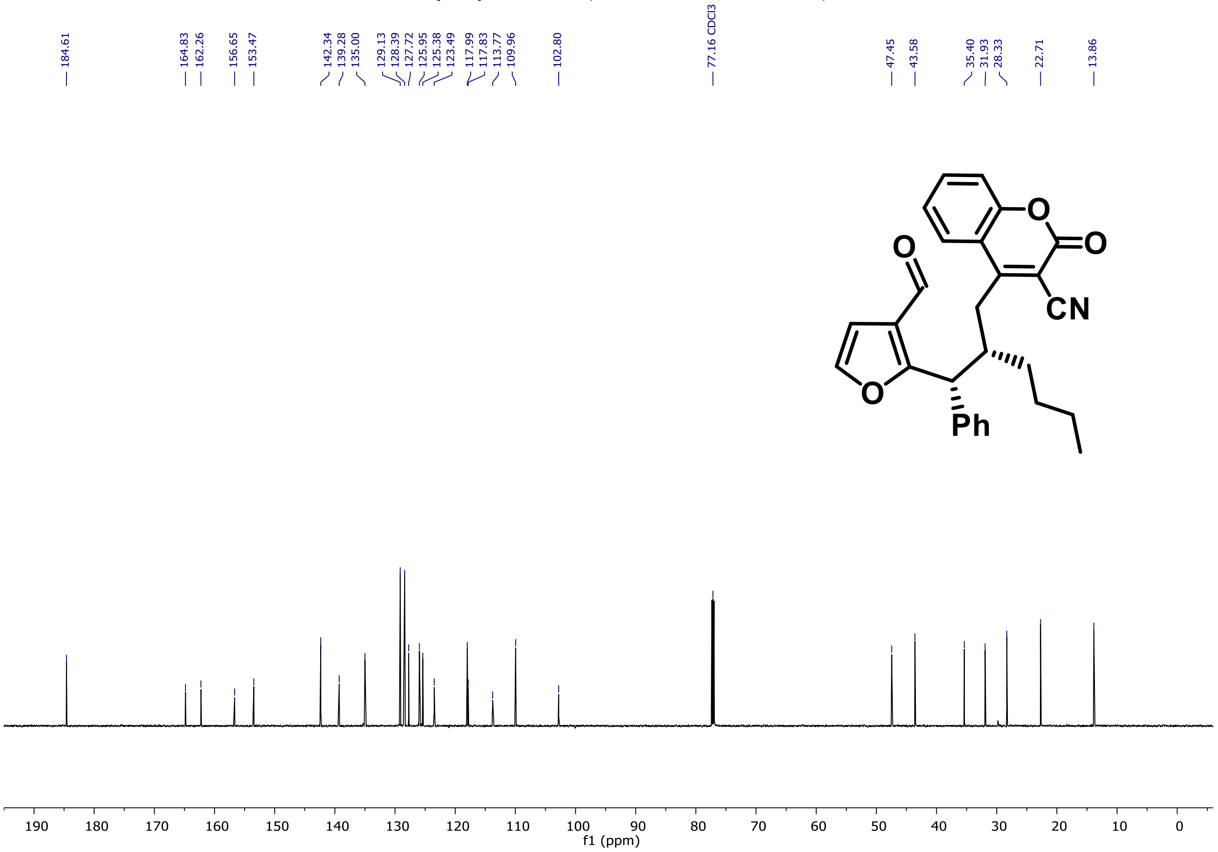

4-((2*R*,3*R*)-3-(3-Formylfuran-2-yl)-2-phenyl-3-(3-(trifluoromethyl)phenyl)propyl)-2-oxo-2*H*-chromene-3-carbonitrile major – 3o

^{1}H NMR (700 MHz, $CDCl_3$)

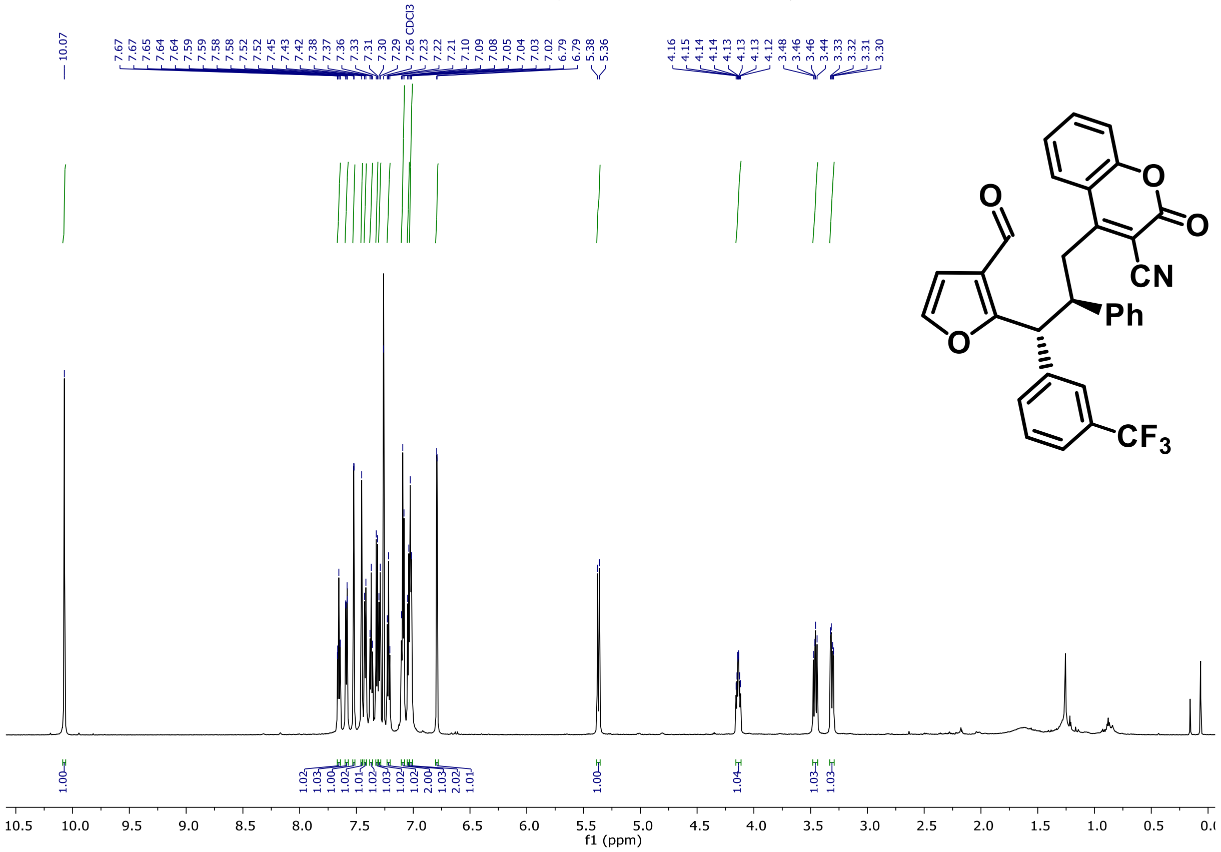

^{13}C{^{1}H} NMR (176 MHz, $CDCl_3$)

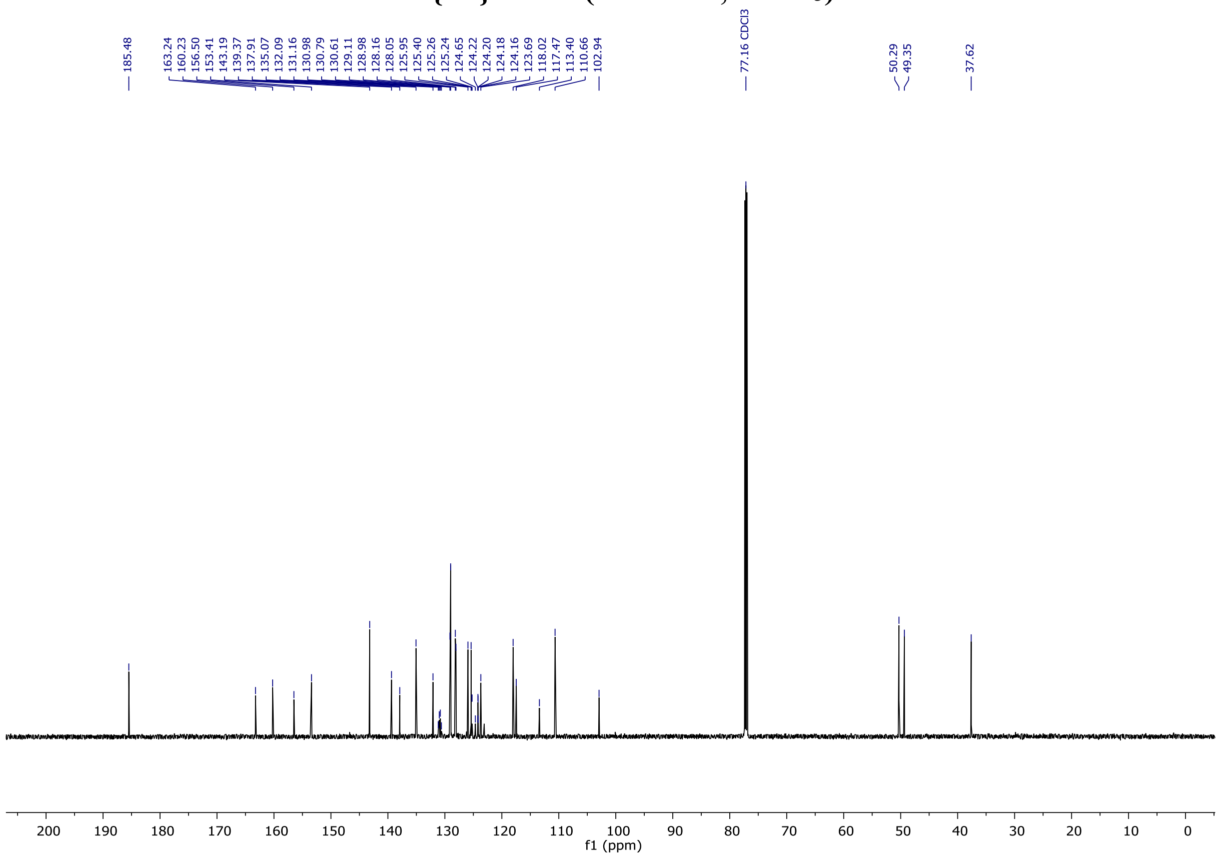

4-((2*S*,3*R*)-3-(3-Formylfuran-2-yl)-2-phenyl-3-(3-(trifluoromethyl)phenyl)propyl)-2-oxo-2*H*-chromene-3-carbonitrile minor – 3o

^{1}H NMR (700 MHz, CDCl$_3$)

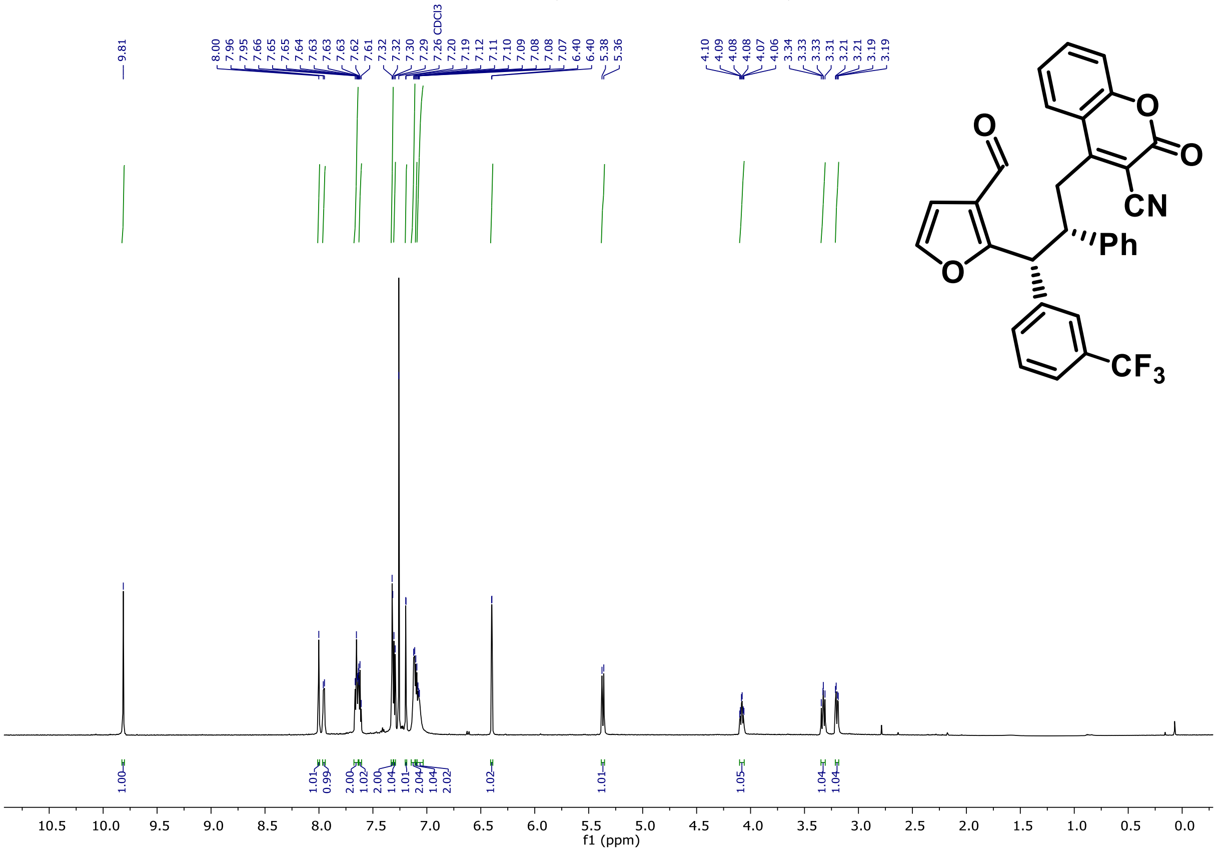

^{13}C{^{1}H} NMR (176 MHz, CDCl$_3$)

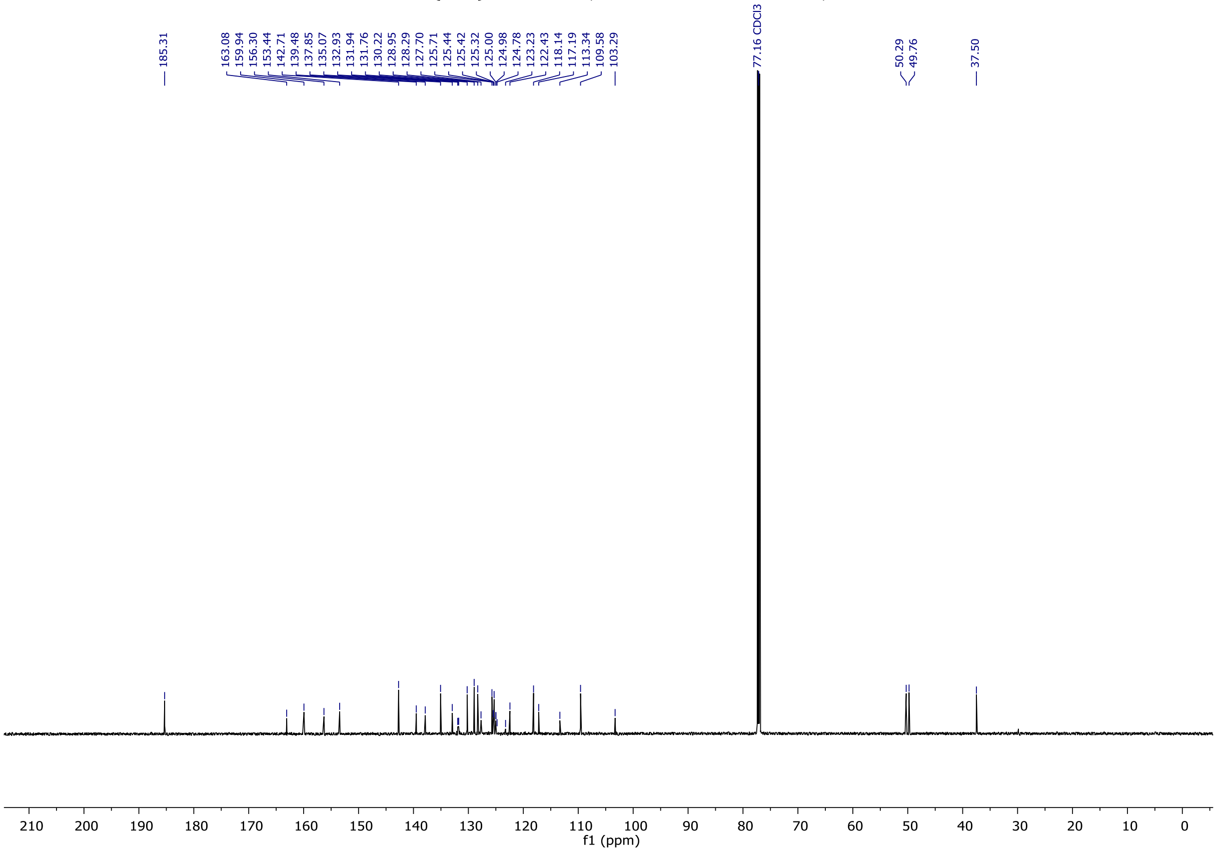

4-((2*R*,3*R*)-3-(4-Fluorophenyl)-3-(3-formylfuran-2-yl)-2-phenylpropyl)-2-oxo-2*H*-chromene-3-carbonitrile major – 3p

^{1}H NMR (700 MHz, $CDCl_3$)

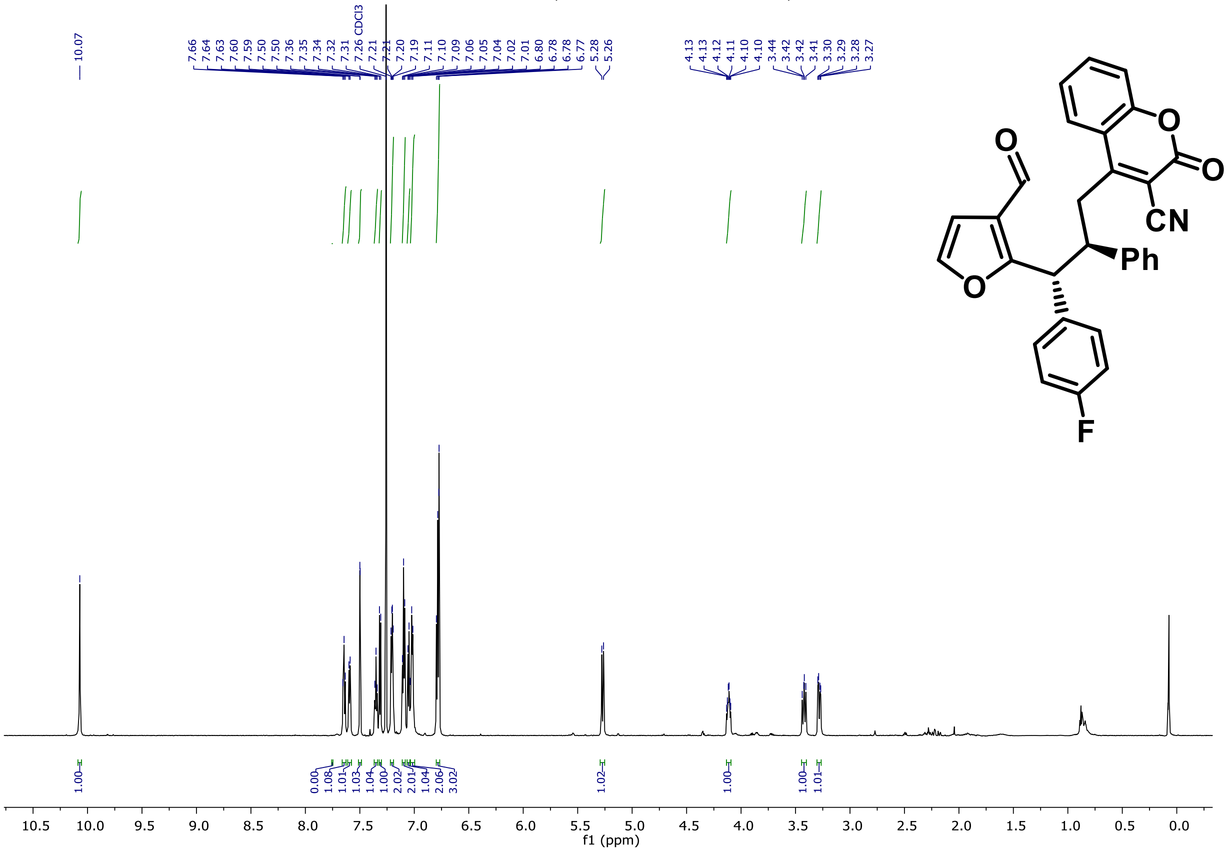

^{13}C{^{1}H} NMR (176 MHz, $CDCl_3$)

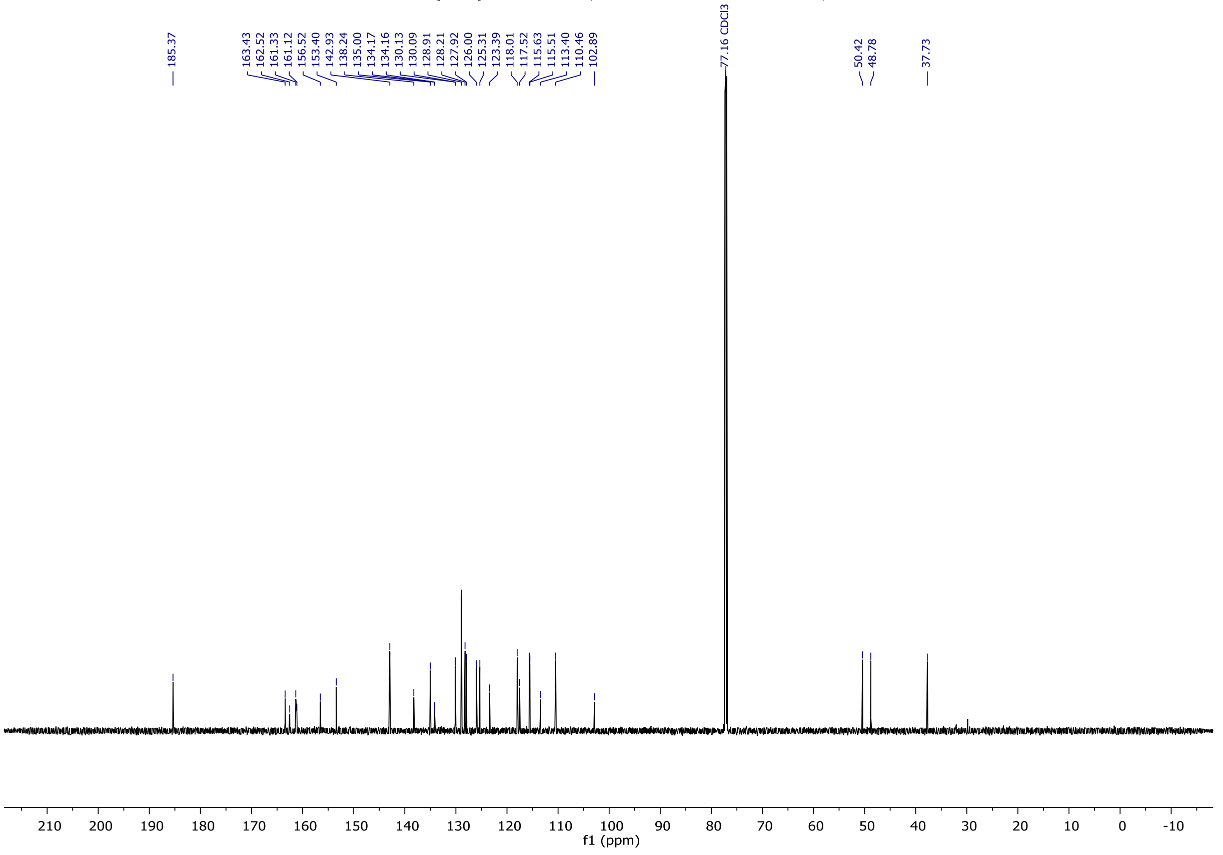

4-((2*S*,3*R*)-3-(4-Fluorophenyl)-3-(3-formylfuran-2-yl)-2-phenylpropyl)-2-oxo-2*H*-chromene-3-carbonitrile minor – 3p

^{1}H NMR (700 MHz, CDCl$_3$)

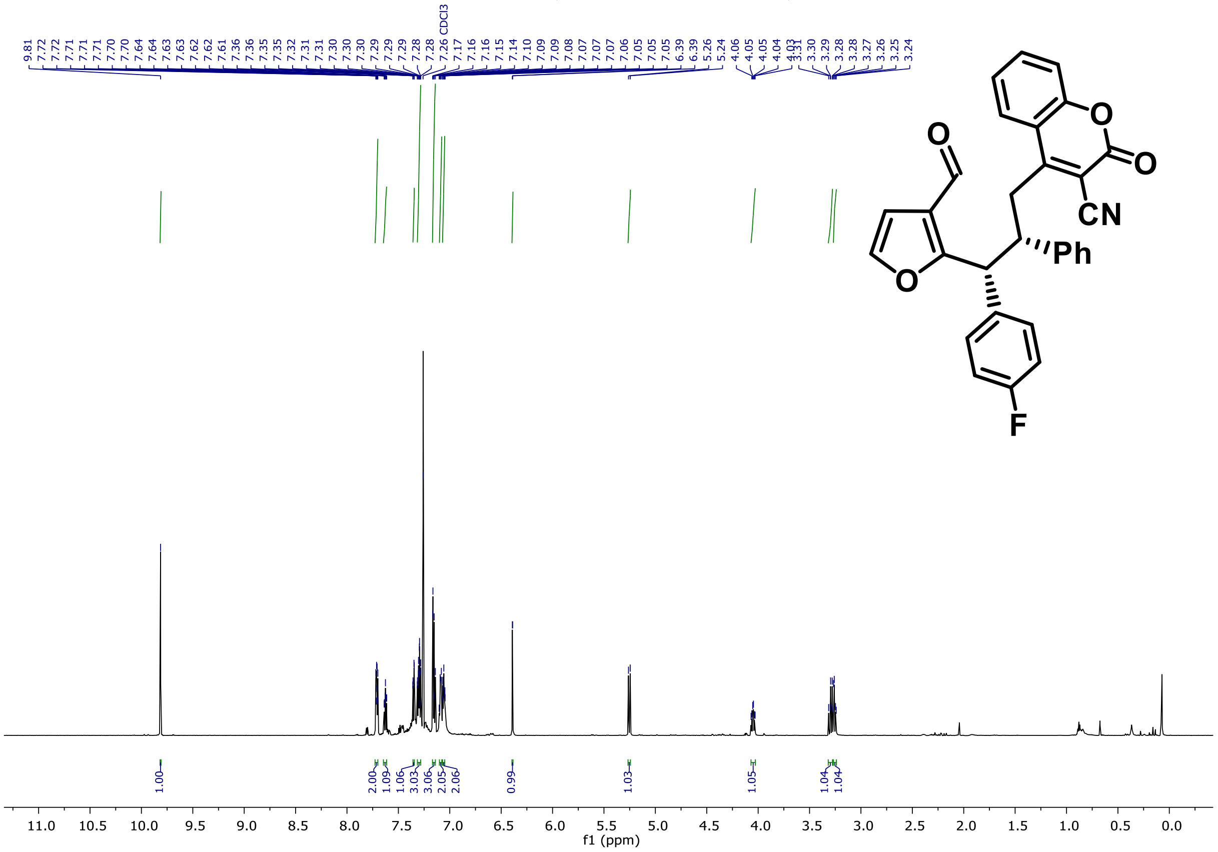

^{13}C{^{1}H} NMR (176 MHz, CDCl$_3$)

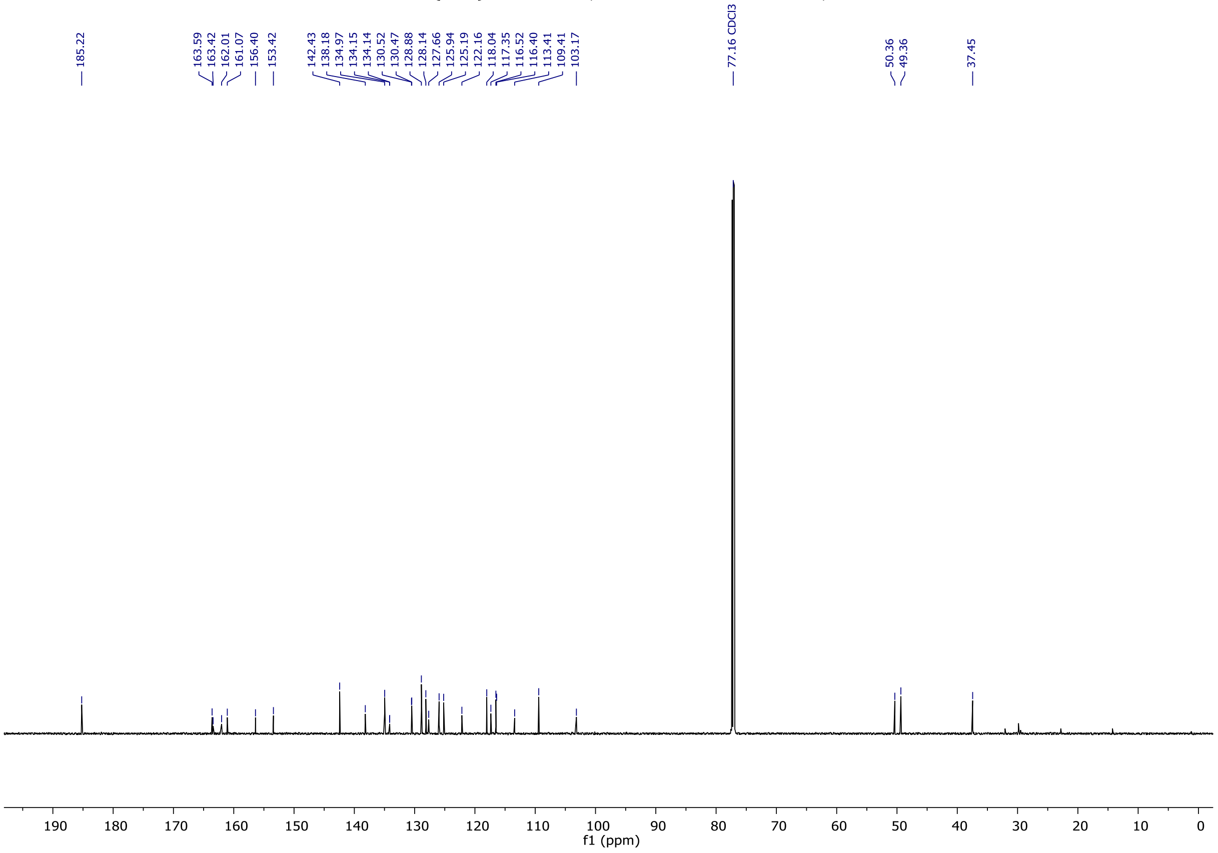

4-((2*R*,3*R*)-3-(3-Formylfuran-2-yl)-2-phenyl-3-(*p*-tolyl)propyl)-2-oxo-2*H*-chromene-3-carbonitrile major – 3q

^{1}H NMR (700 MHz, CDCl$_3$)

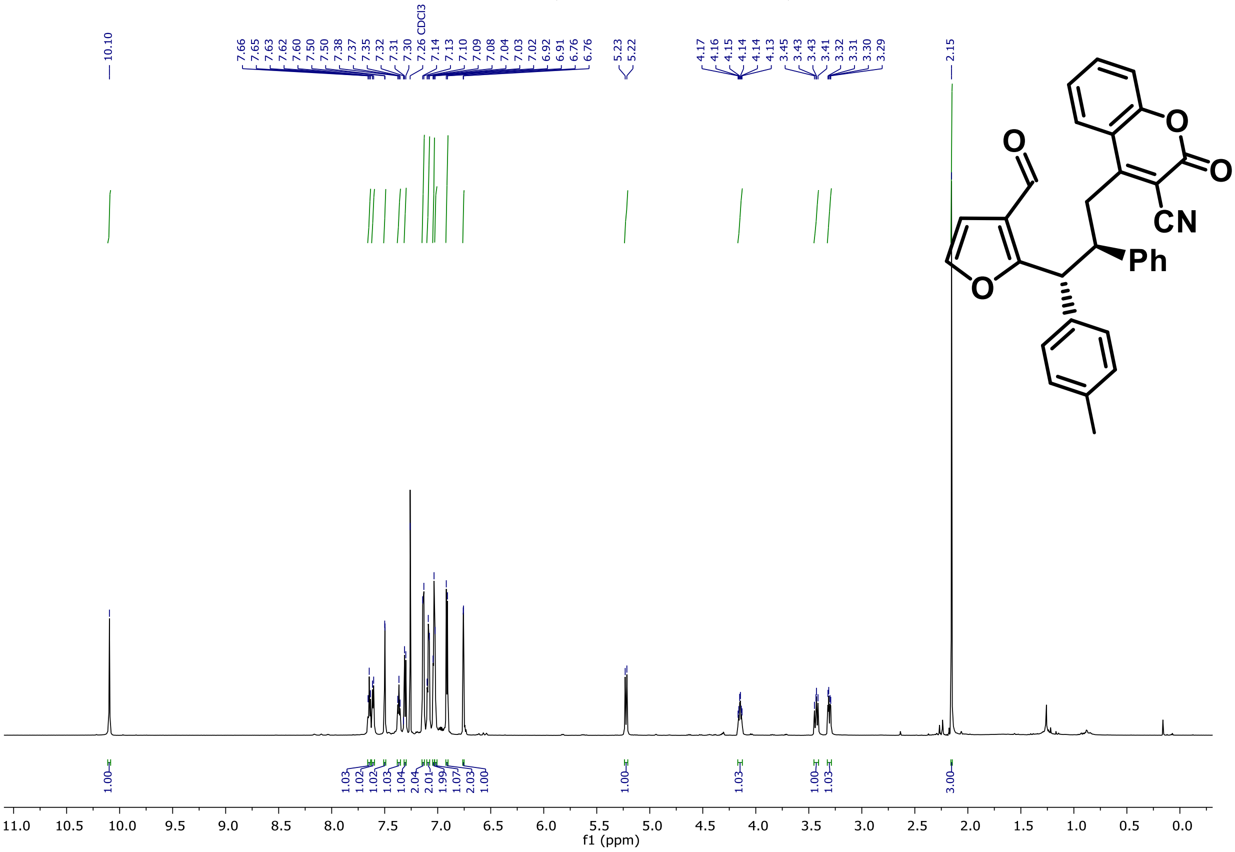

^{13}C{^{1}H} NMR (176 MHz, CDCl$_3$)

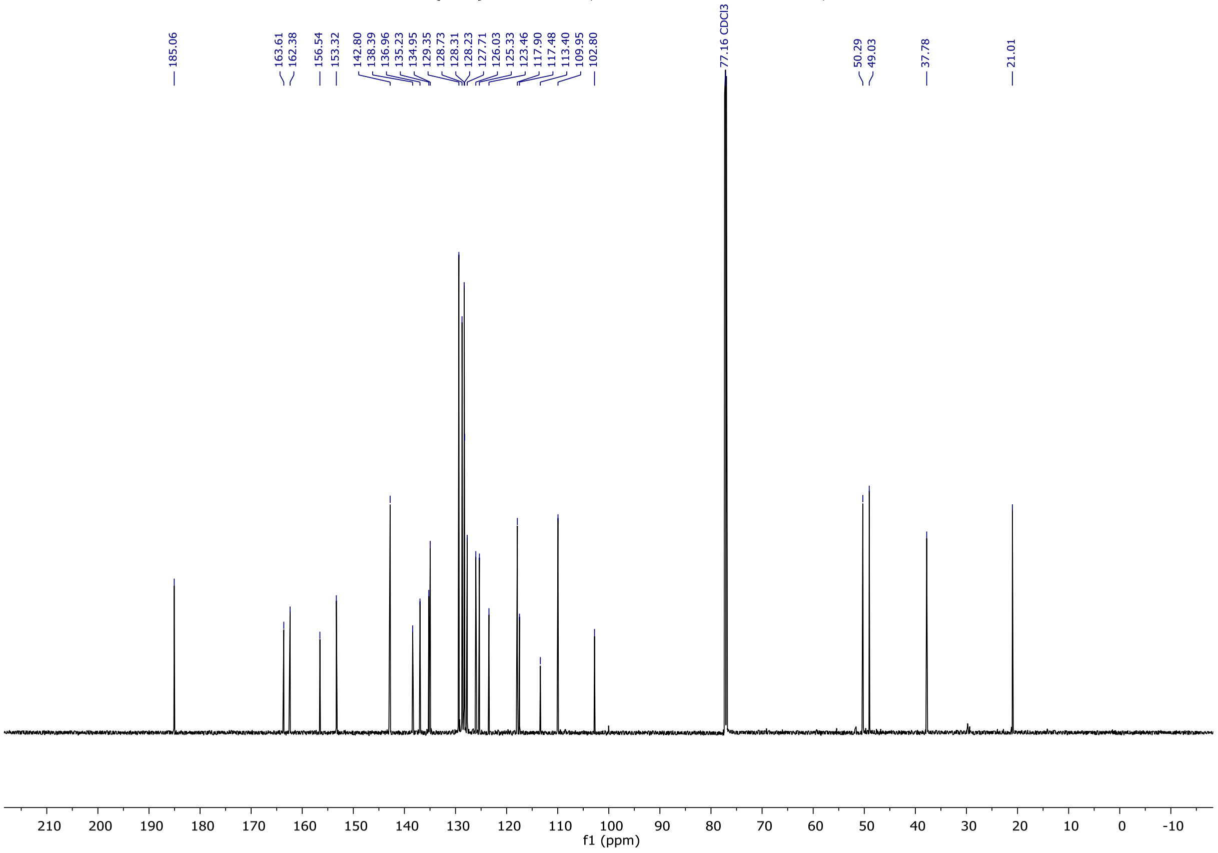

4-((2*S*,3*R*)-3-(3-Formylfuran-2-yl)-2-phenyl-3-(*p*-tolyl)propyl)-2-oxo-2*H*-chromene-3-carbonitrile minor – 3q

^{1}H NMR (700 MHz, CDCl$_3$)

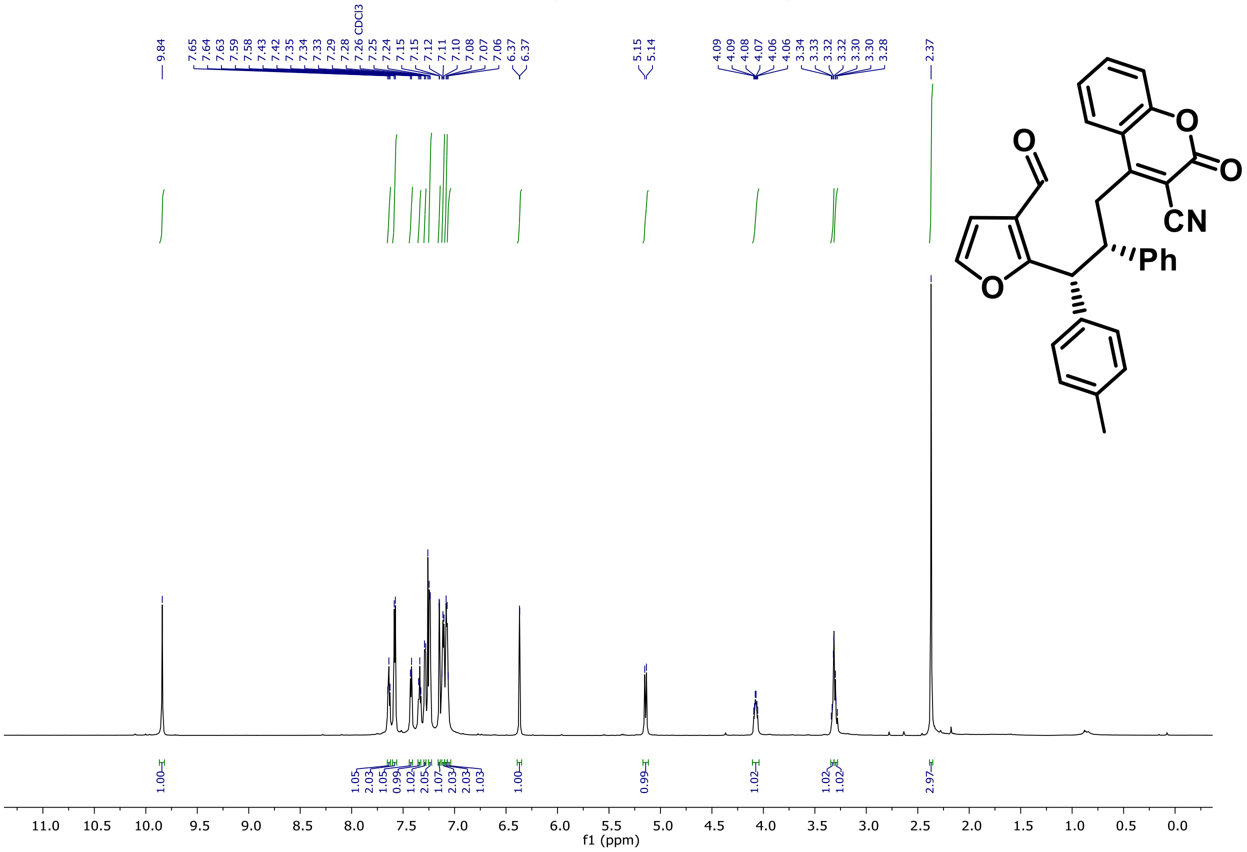

^{13}C{^{1}H} NMR (176 MHz, CDCl$_3$)

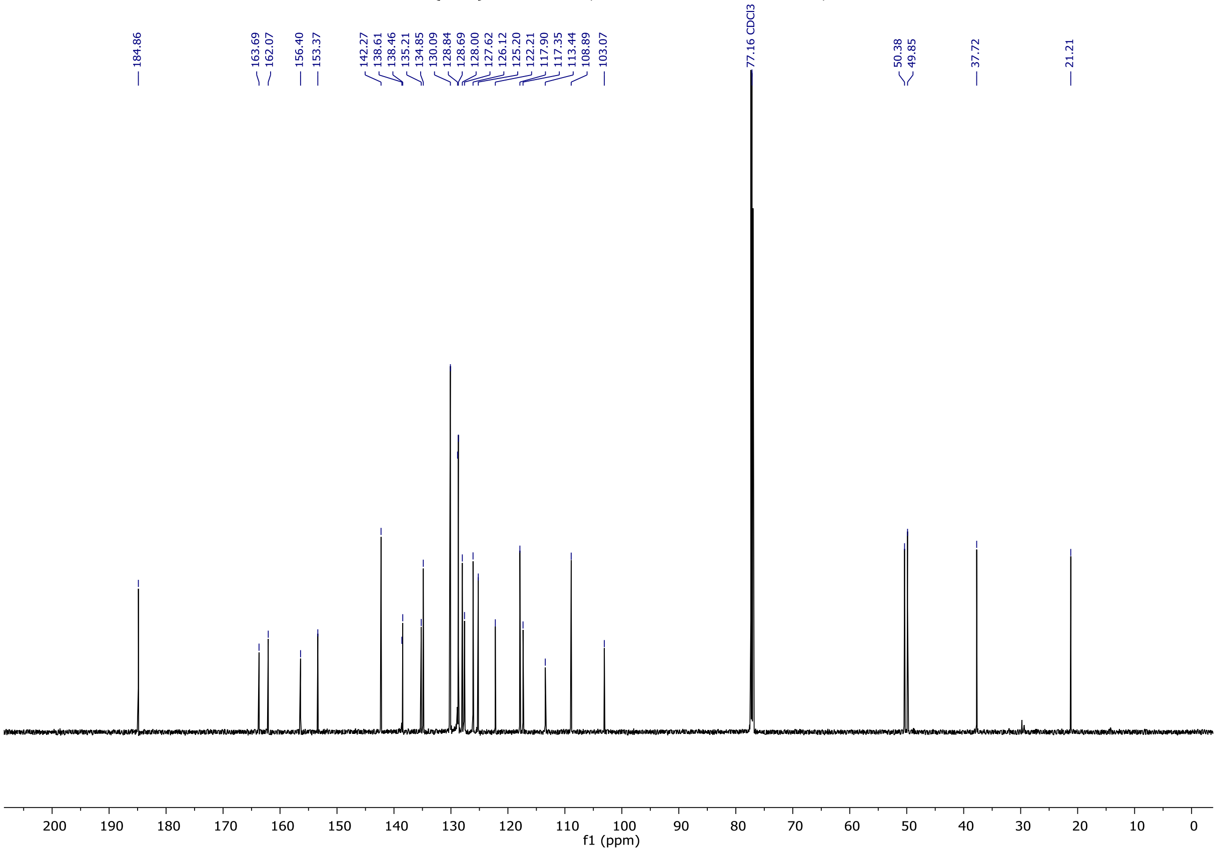

4-((2*R*,3*R*)-3-(3-Formylfuran-2-yl)-3-(3-methoxyphenyl)-2-phenylpropyl)-2-oxo-2*H*-chromene-3-carbonitrile major – 3r

^{1}H NMR (700 MHz, CDCl$_3$)

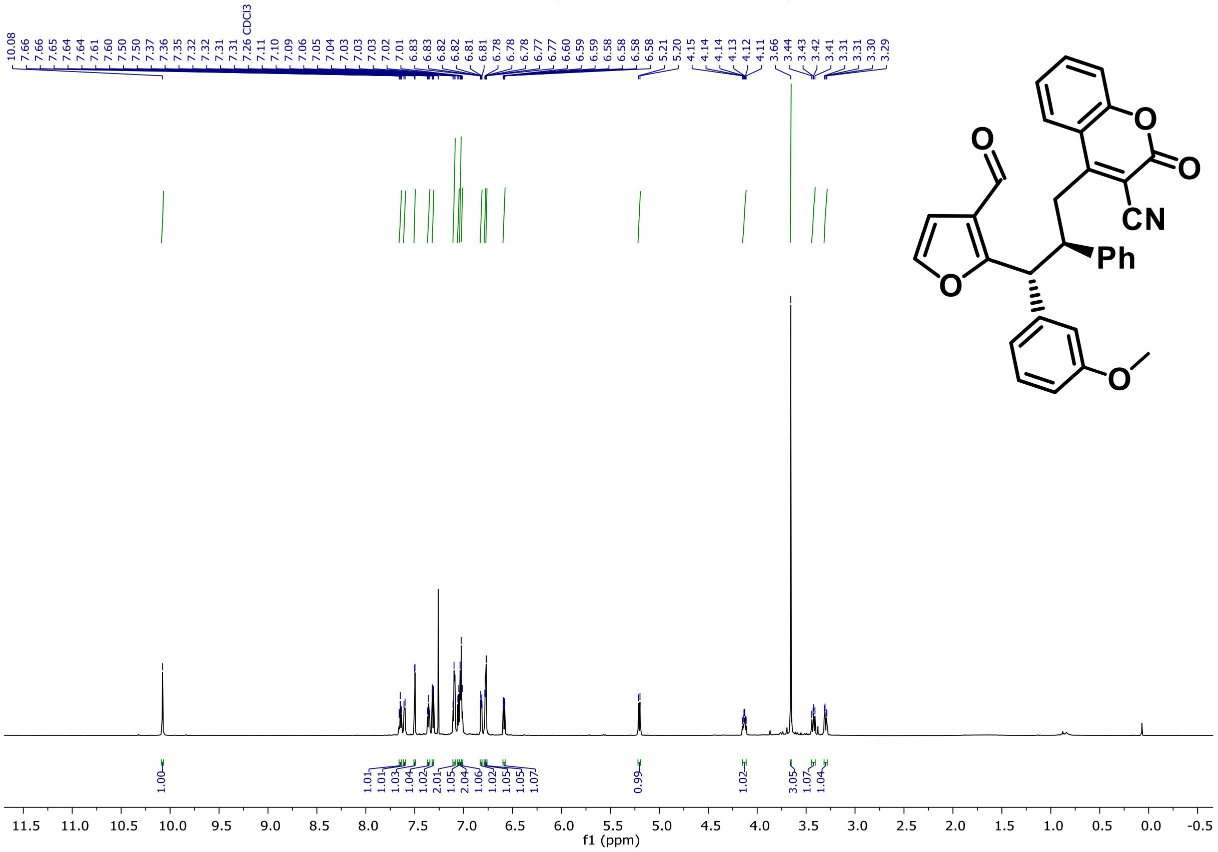

^{13}C{^{1}H} NMR (176 MHz, CDCl$_3$)

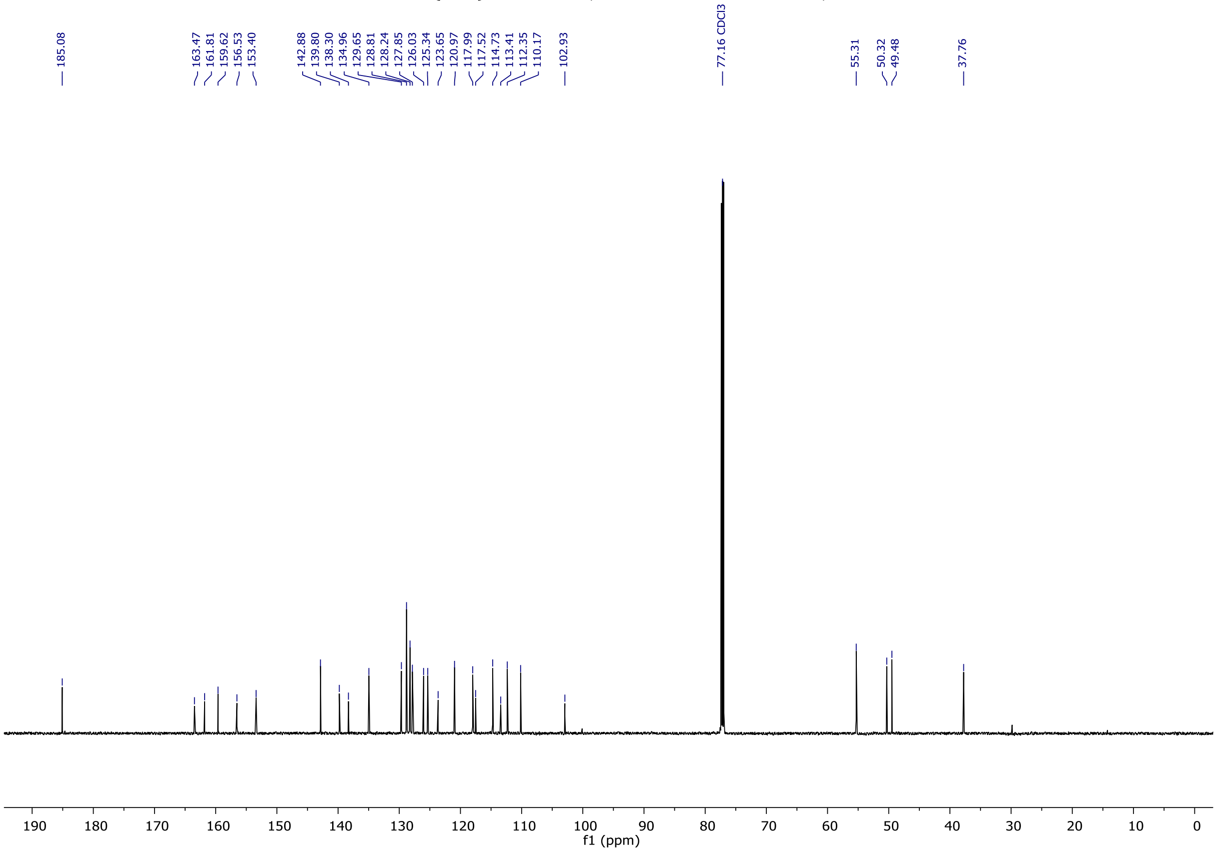

4-((2*S*,3*R*)-3-(3-Formylfuran-2-yl)-3-(3-methoxyphenyl)-2-phenylpropyl)-2-oxo-2*H*-chromene-3-carbonitrile minor – 3r

^{1}H NMR (700 MHz, CDCl$_3$)

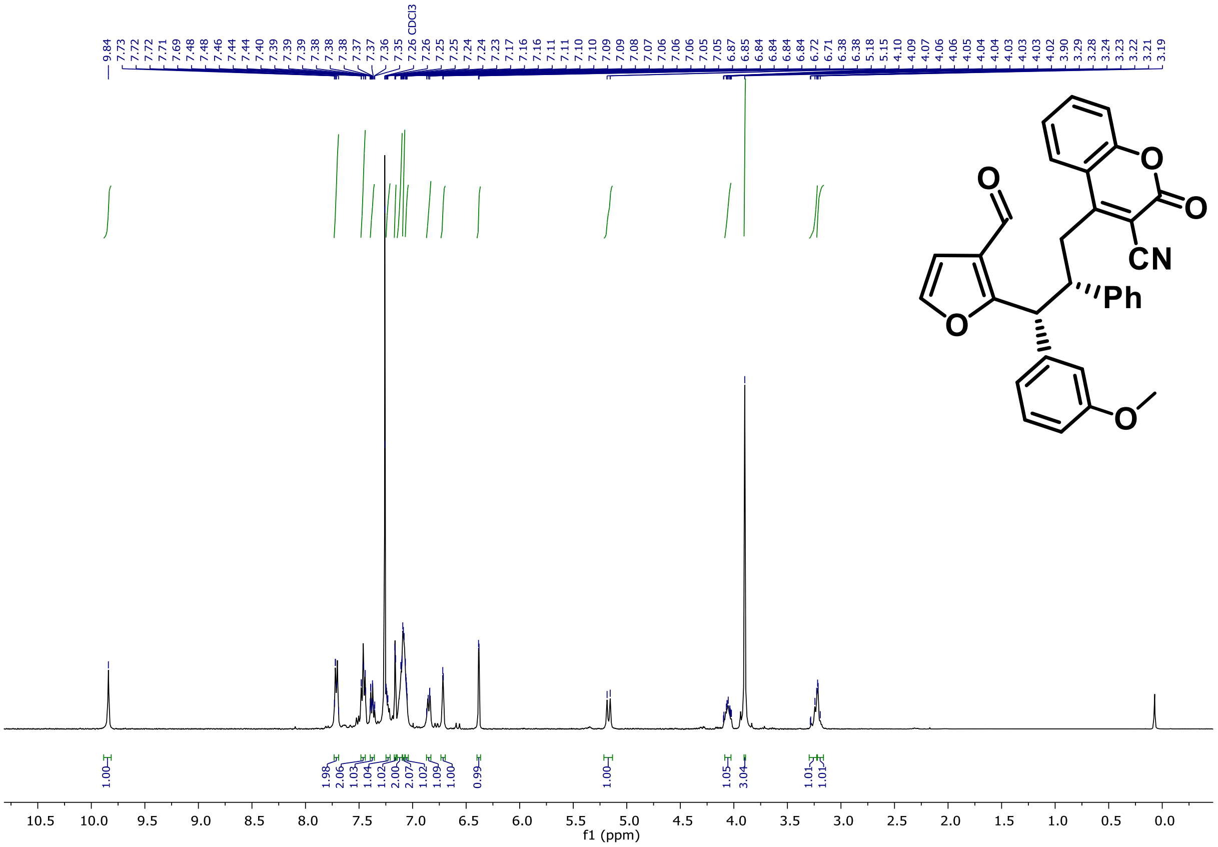

^{13}C{^{1}H} NMR (176 MHz, CDCl$_3$)

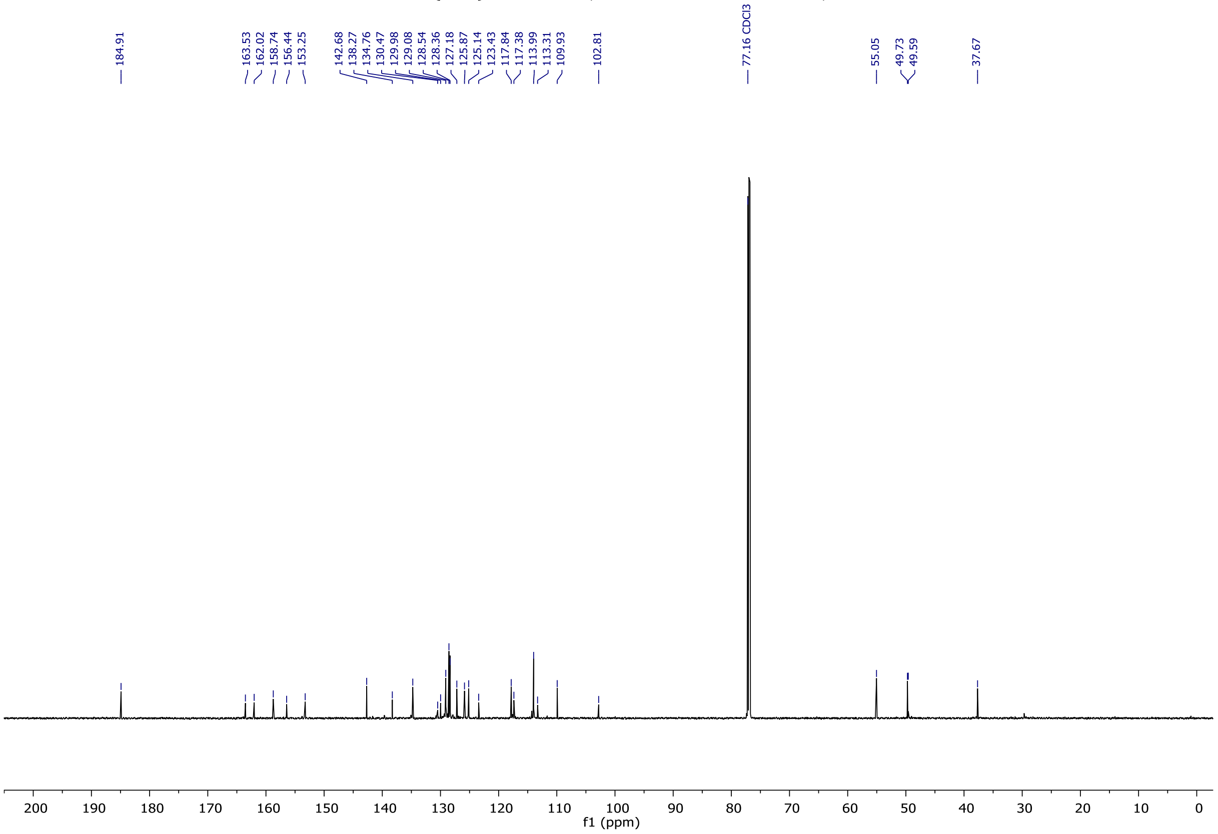

4-((2*R*,3*R*)-3-(5-Formylfuran-2-yl)-2,3-diphenylpropyl)-2-oxo-2*H*-chromene-3-carbonitrile major – 3s

^{1}H NMR (700 MHz, $CDCl_3$)

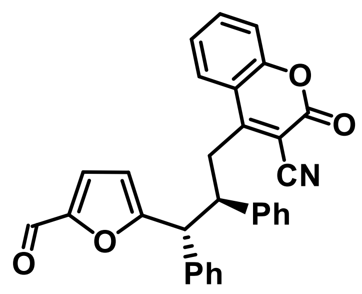

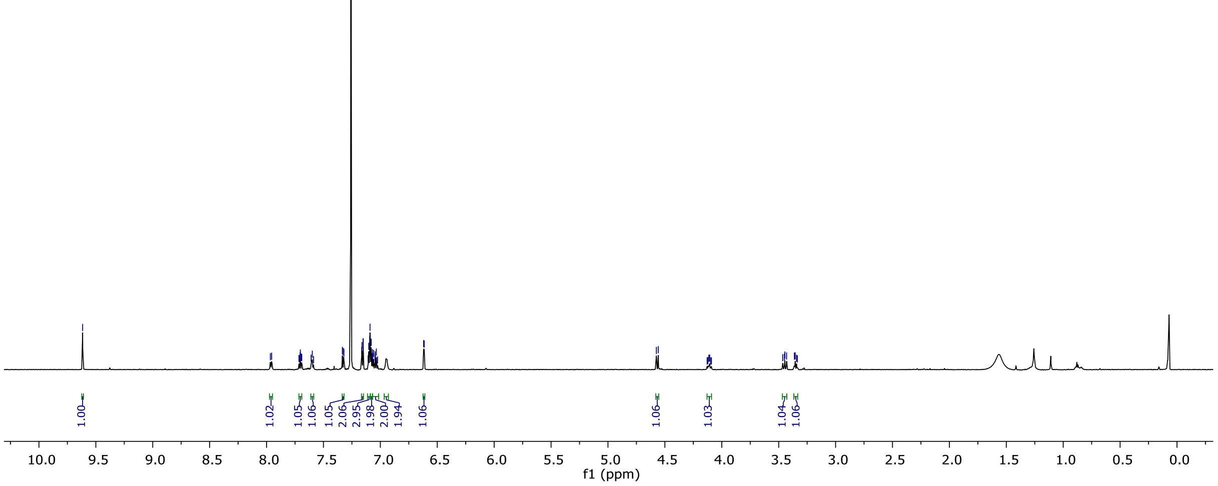

^{13}C{^{1}H} NMR (176 MHz, $CDCl_3$)

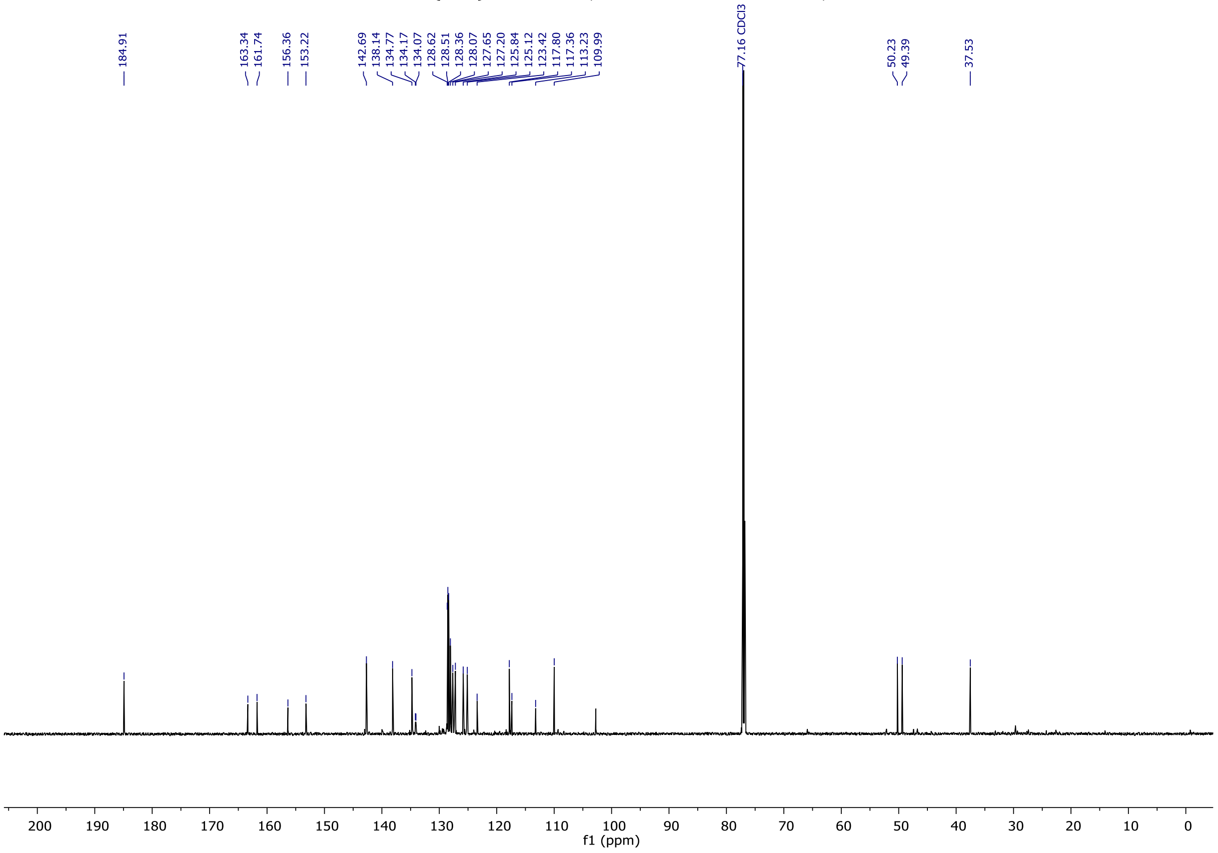

4-((2*S*,3*R*)-3-(5-Formylfuran-2-yl)-2,3-diphenylpropyl)-2-oxo-2*H*-chromene-3-carbonitrile minor – 3s

^{1}H NMR (700 MHz, CDCl$_3$)

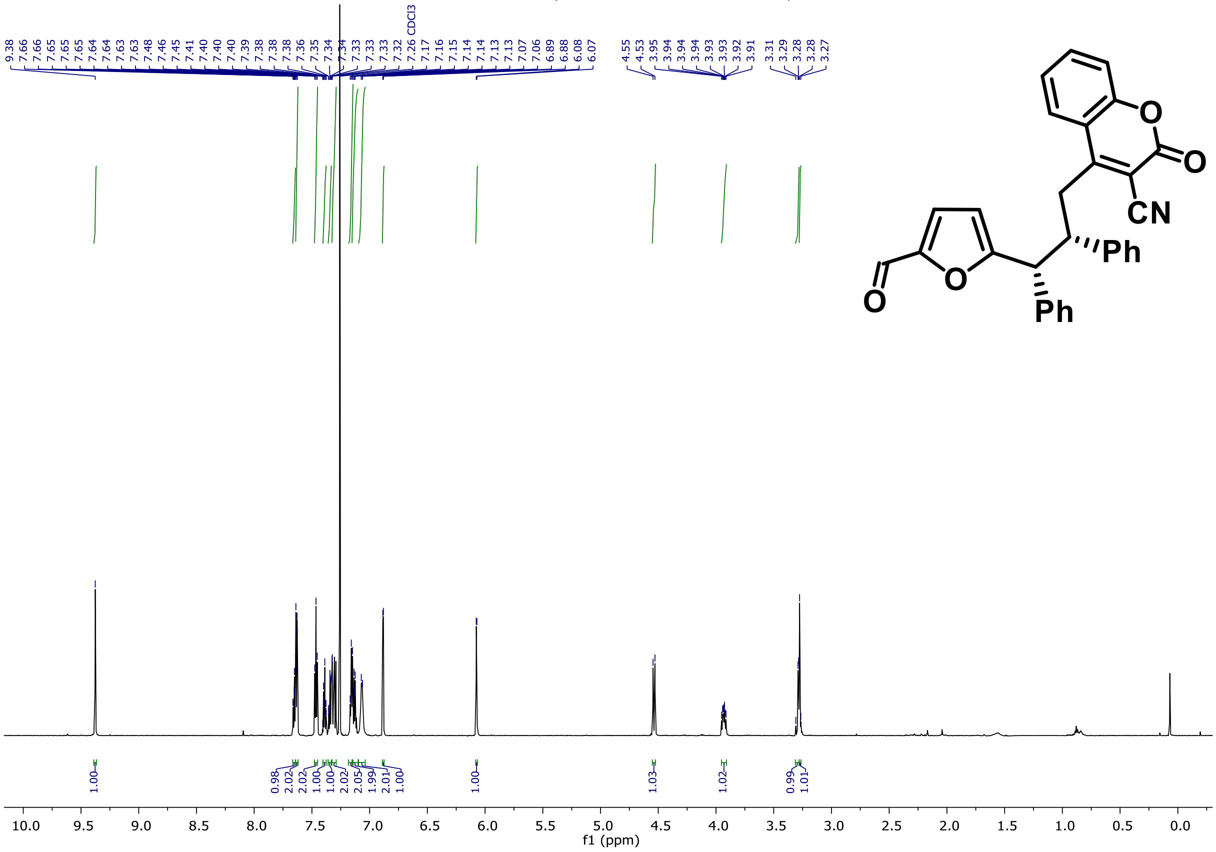

^{13}C{^{1}H} NMR (176 MHz, CDCl$_3$)

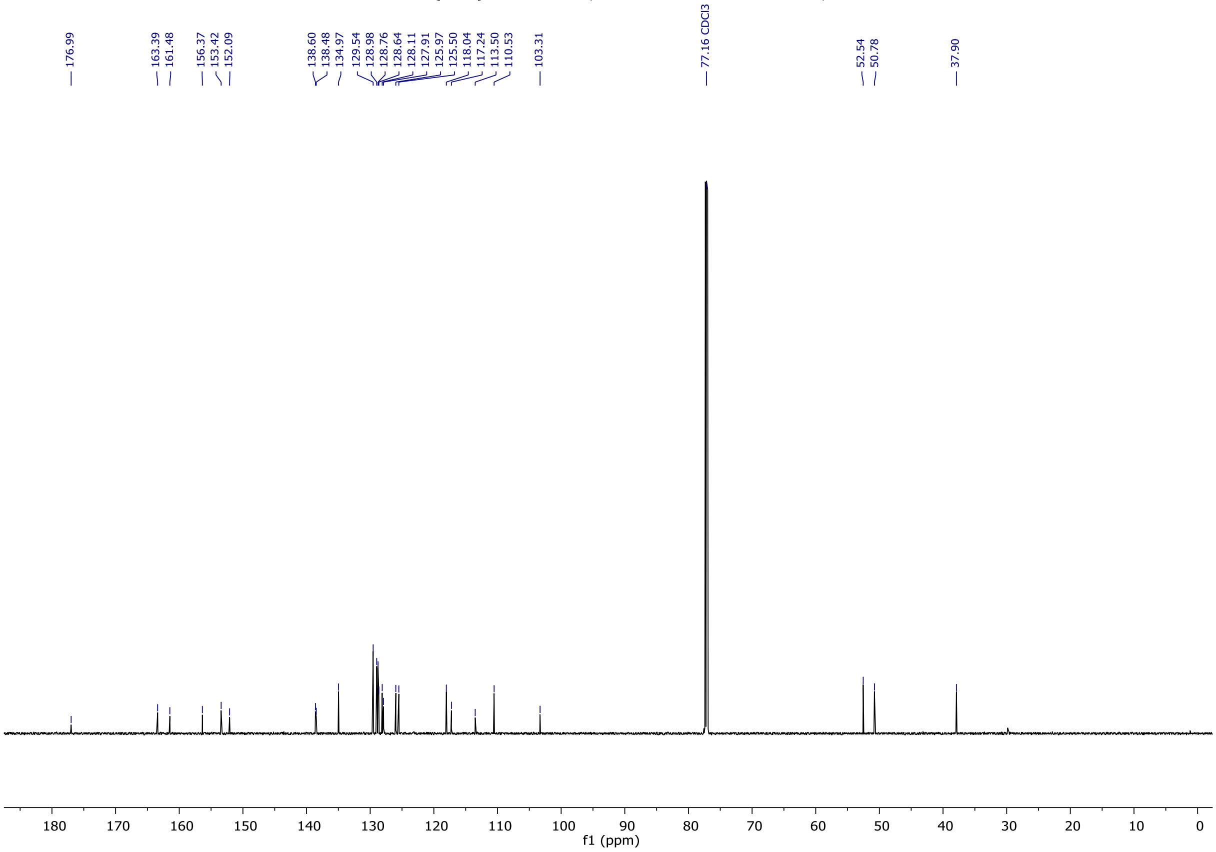

4-((2*S*,3*R*)-3-(3-(2,2-dibromovinyl)furan-2-yl)-3-phenyl-2-(*p*-tolyl)propyl)-2-oxo-2*H*-chromene-3-carbonitrile 5

^{1}H NMR (700 MHz, $CDCl_3$)

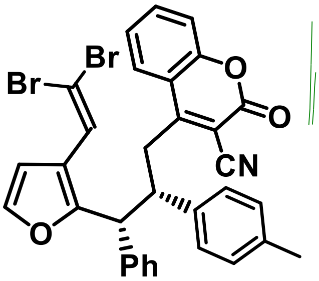

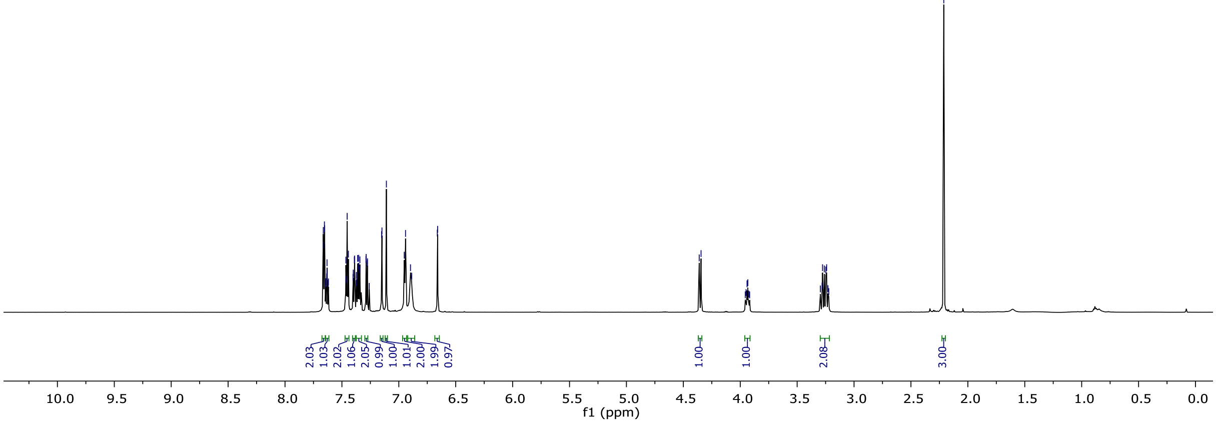

$^{13}C\{^1H\}$ NMR (176 MHz, $CDCl_3$)

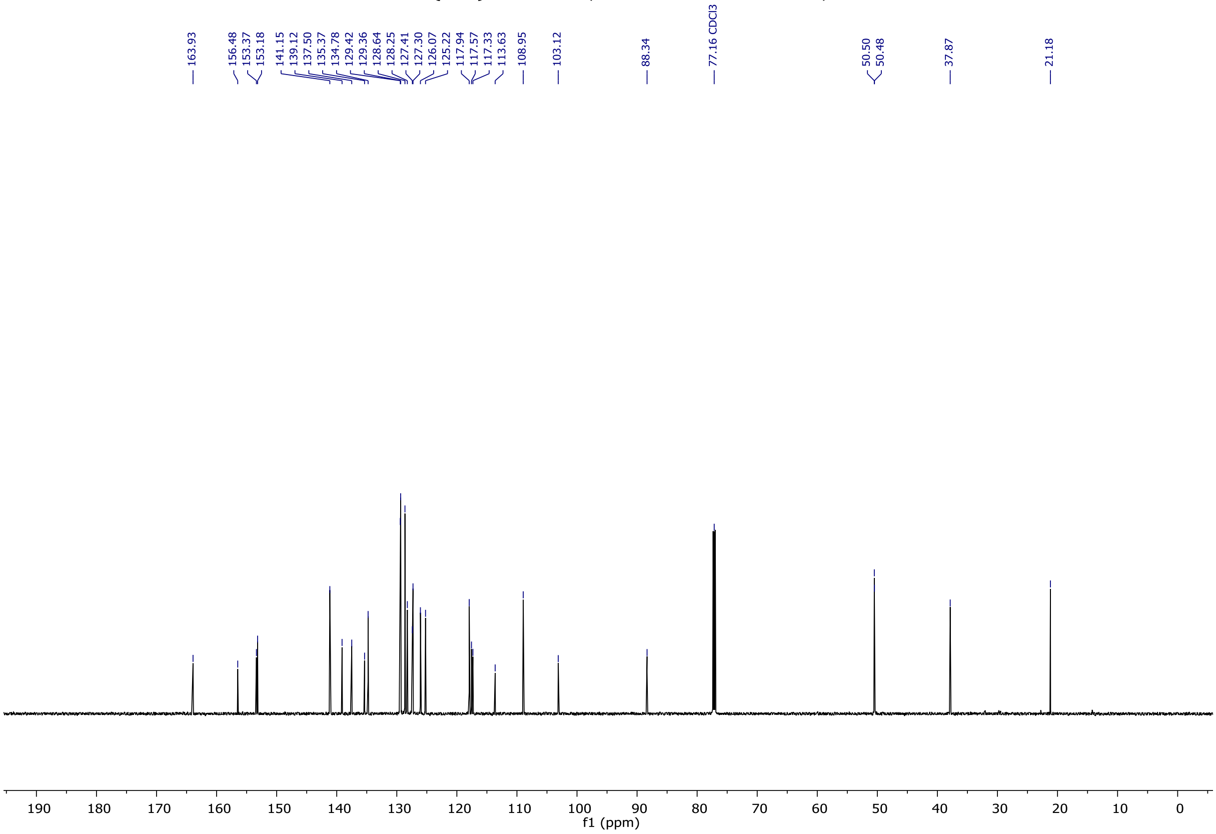

4-((6*S*,7*R*)-6,7-diphenyl-6,7-dihydrobenzofuran-5-yl)-2*H*-chromen-2-one 6

^{1}H NMR (700 MHz, $CDCl_3$)

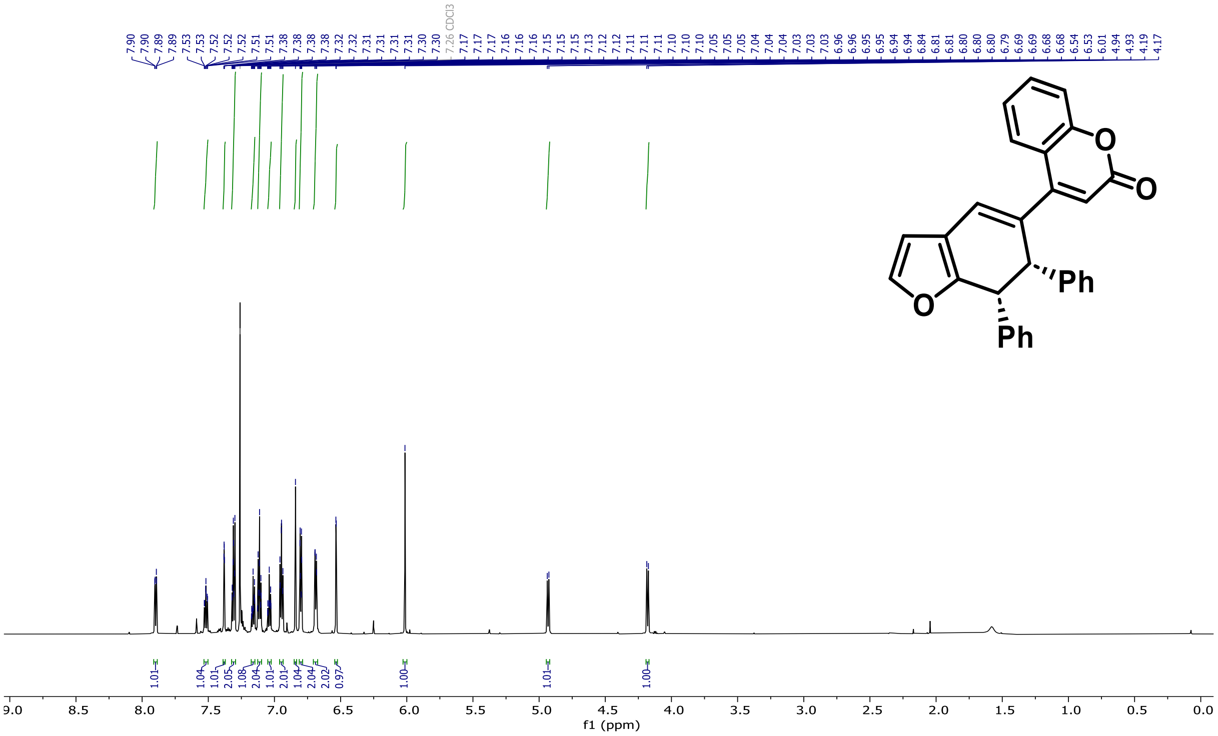

^{13}C{^{1}H} NMR (176 MHz, $CDCl_3$)

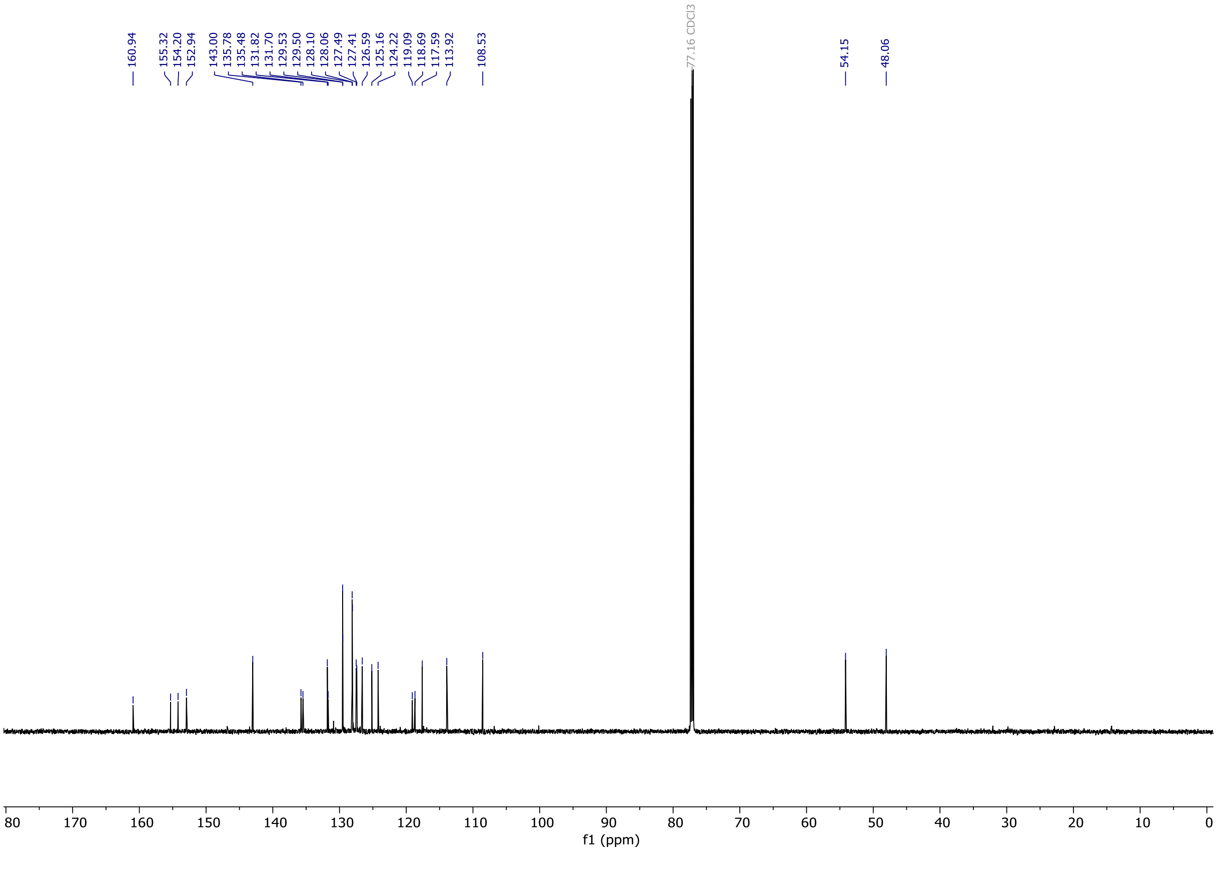

9. UPC² data

4-((2*R*,3*R*)-3-(3-formylfuran-2-yl)-2,3-diphenylpropyl)-2-oxo-2*H*-chromene-3-carbonitrile major – 3a

Racemic sample

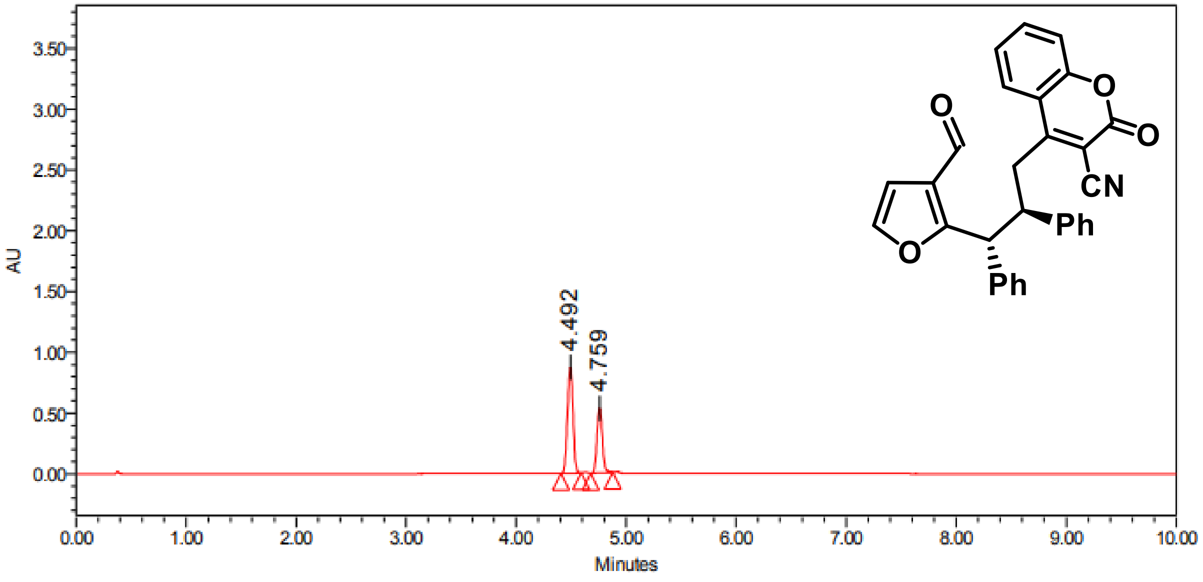

Peak Results

	RT	% Area
1	4.492	63.84
2	4.759	36.16

Enantiomerically enriched sample

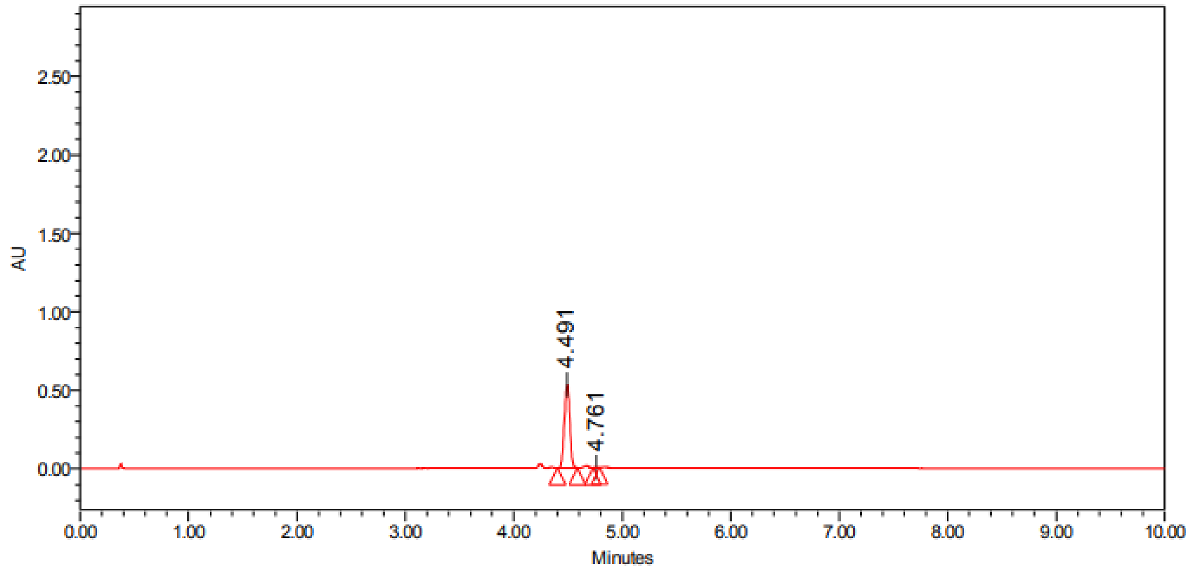

Peak Results

	RT	% Area
1	4.491	99.85
2	4.761	0.15

4-((2*S*,3*R*)-3-(3-formylfuran-2-yl)-2,3-diphenylpropyl)-2-oxo-2*H*-chromene-3-carbonitrile minor – 3a

Racemic sample

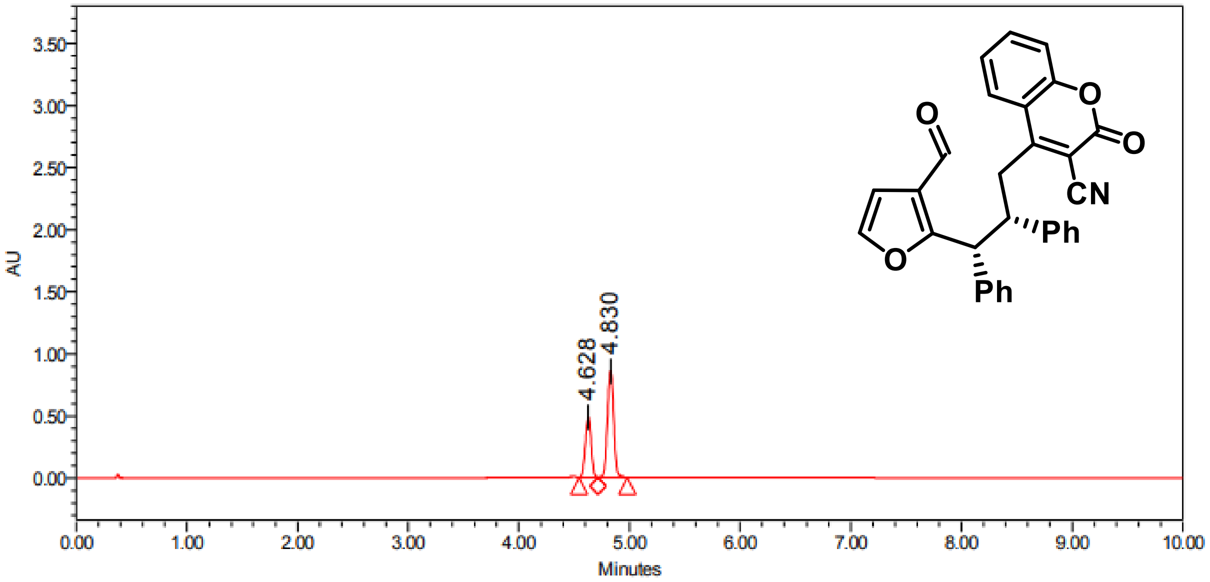

Peak Results

	RT	% Area
1	4.628	34.67
2	4.830	65.33

Enantiomerically enriched sample

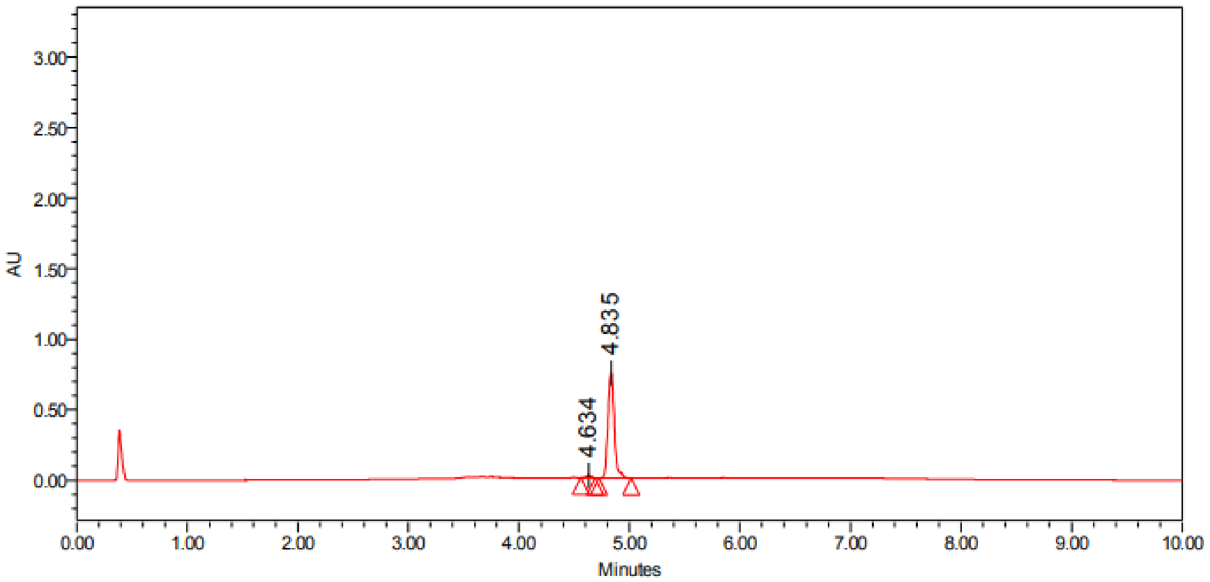

Peak Results

	RT	% Area
1	4.634	1.90
2	4.835	98.10

4-((2*R*,3*R*)-2-(3-fluorophenyl)-3-(3-formylfuran-2-yl)-3-phenylpropyl)-2-oxo-2*H*-chromene-3-carbonitrile major – 3b

Racemic sample

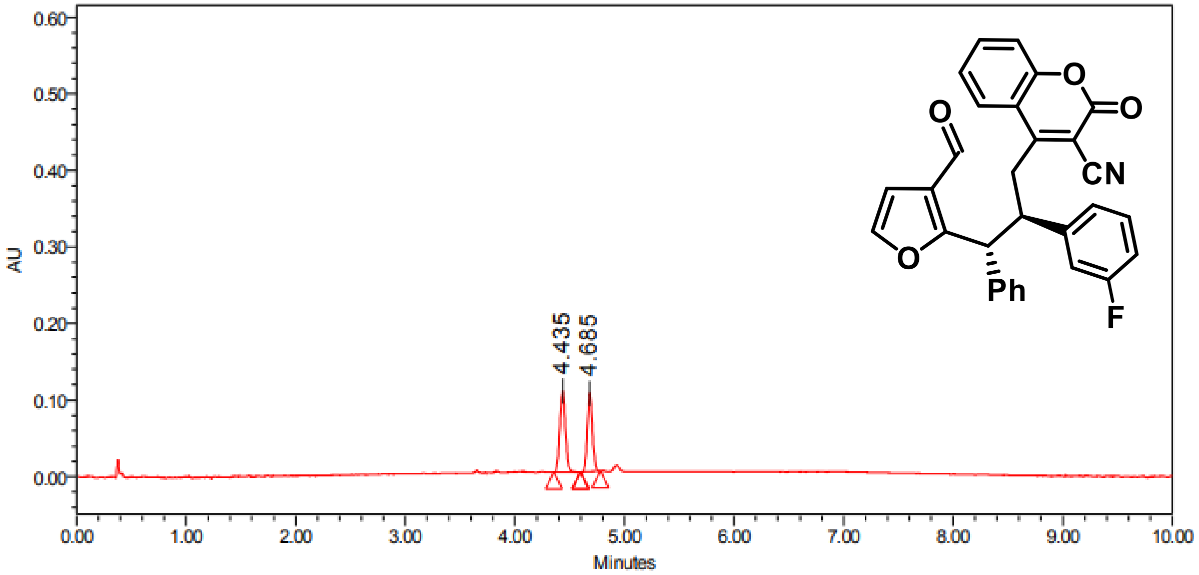

Peak Results

	RT	% Area
1	4.435	53.15
2	4.685	46.85

Enantiomerically enriched sample

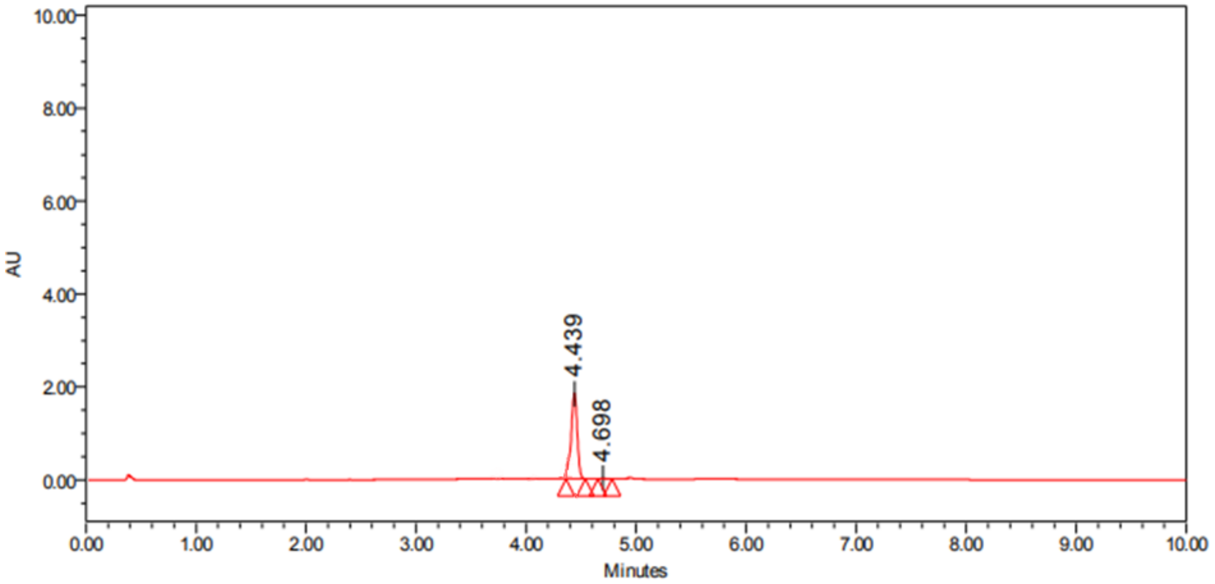

Peak Results

	RT	% Area
1	4.439	99.24
2	4.698	0.76

4-((2*S*,3*R*)-2-(3-fluorophenyl)-3-(3-formylfuran-2-yl)-3-phenylpropyl)-2-oxo-2*H*-chromene-3-carbonitrile minor – 3b

Racemic sample

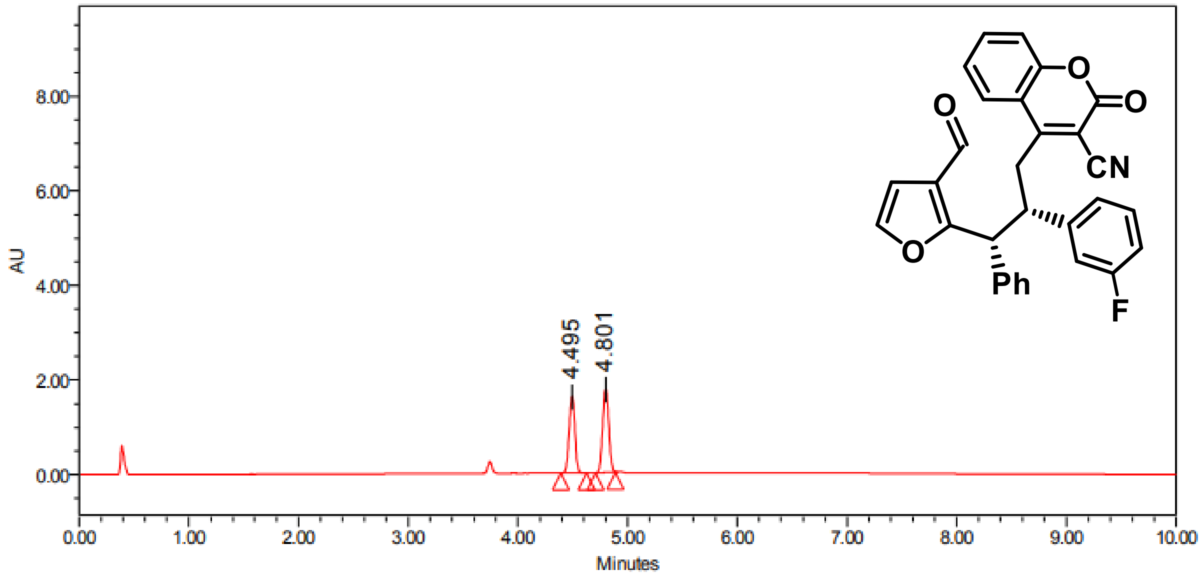

Peak Results

	RT	% Area
1	4.495	46.97
2	4.801	53.03

Enantiomerically enriched sample

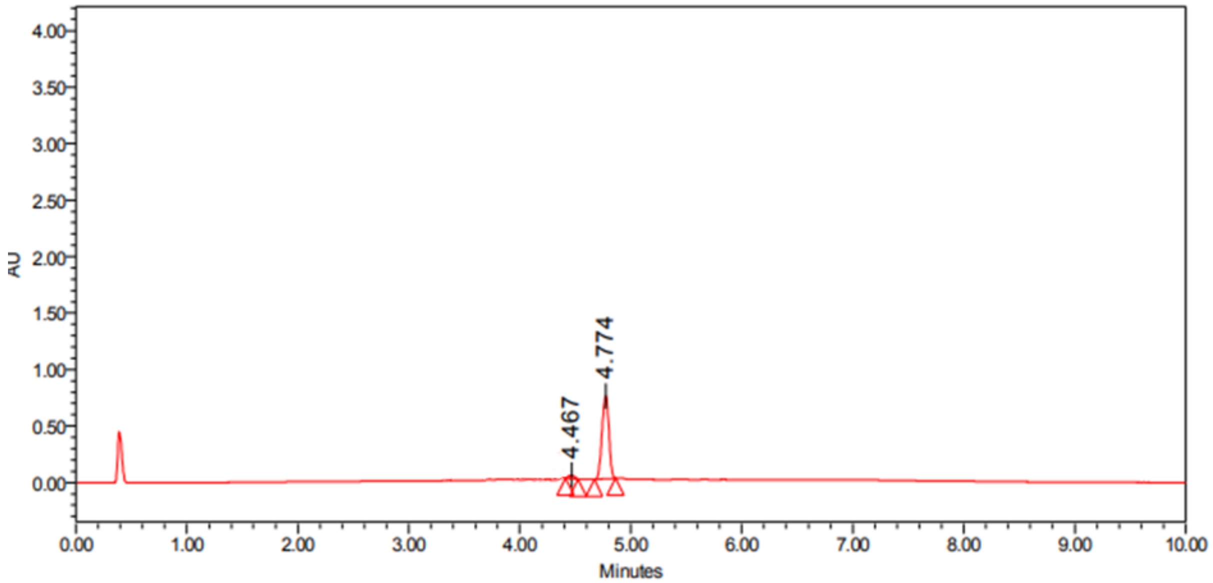

Peak Results

	RT	% Area
1	4.467	3.08
2	4.774	96.92

4-((2*R*,3*R*)-2-(2-chlorophenyl)-3-(3-formylfuran-2-yl)-3-phenylpropyl)-2-oxo-2*H*-chromene-3-carbonitrile major – 3c

Racemic sample

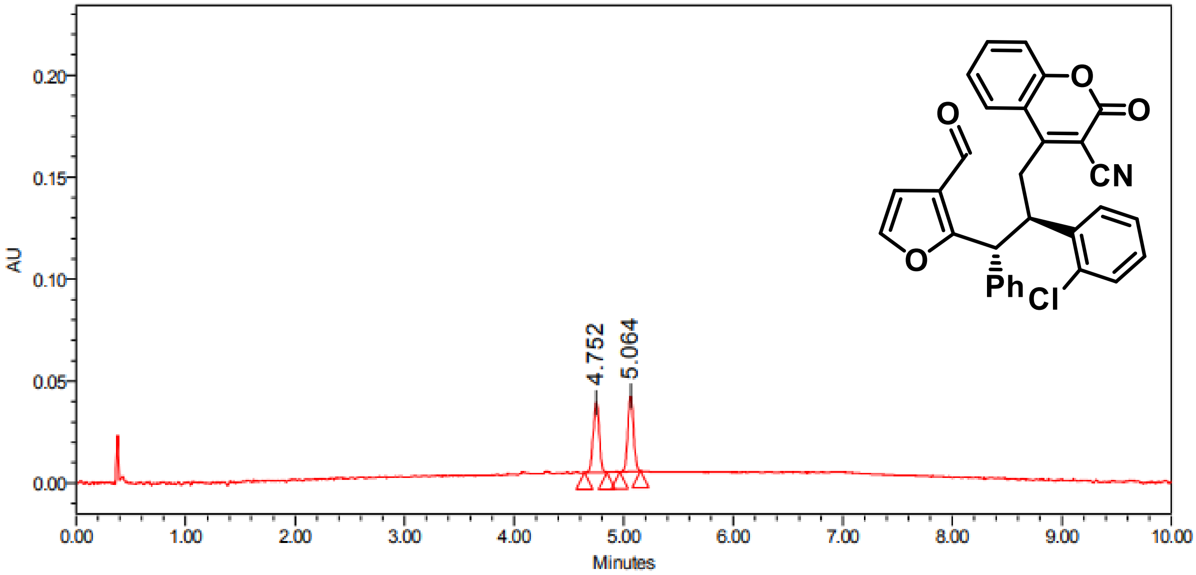

Peak Results

	RT	% Area
1	4.752	49.26
2	5.064	50.74

Enantiomerically enriched sample

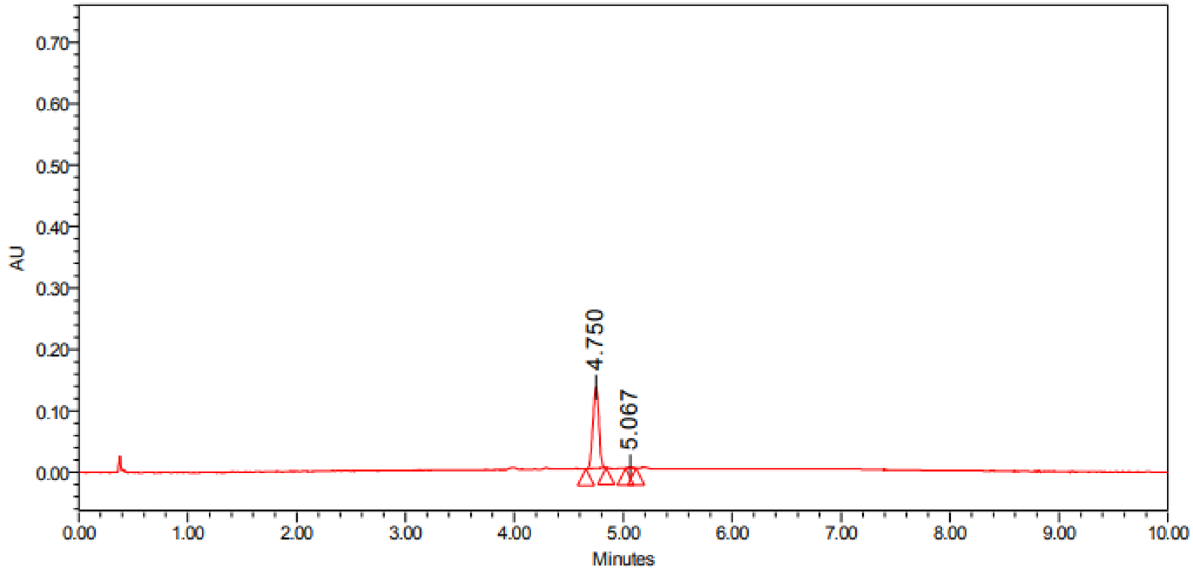

Peak Results

	RT	% Area
1	4.750	98.83
2	5.067	1.17

4-((2*S*,3*R*)-2-(2-chlorophenyl)-3-(3-formylfuran-2-yl)-3-phenylpropyl)-2-oxo-2*H*-chromene-3-carbonitrile minor – 3c

Racemic sample

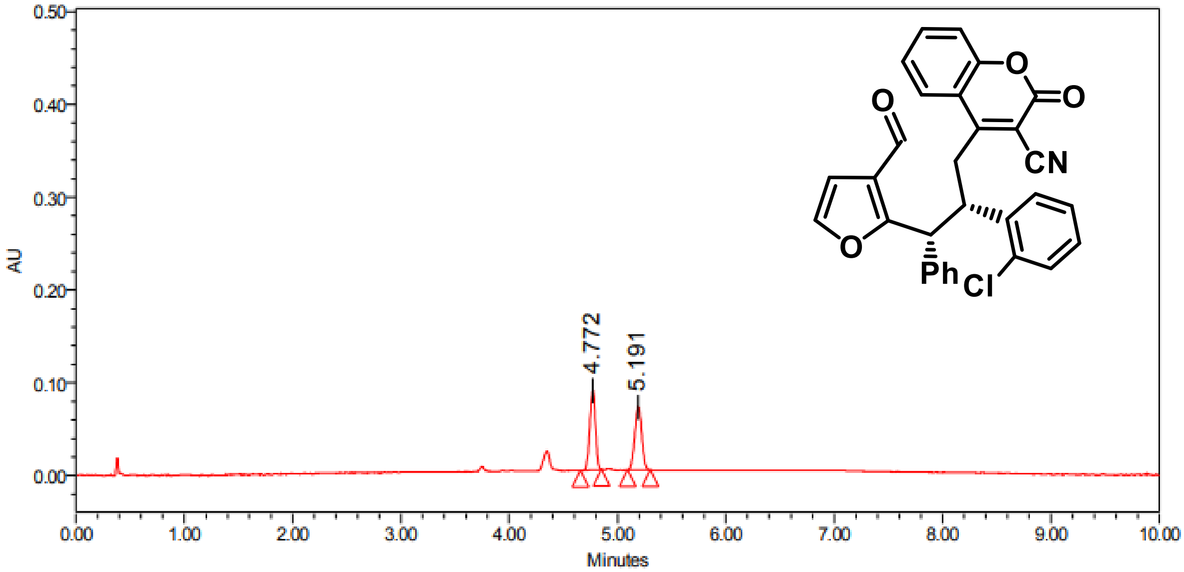

Peak Results

	RT	% Area
1	4.772	50.94
2	5.191	49.06

Enantiomerically enriched sample

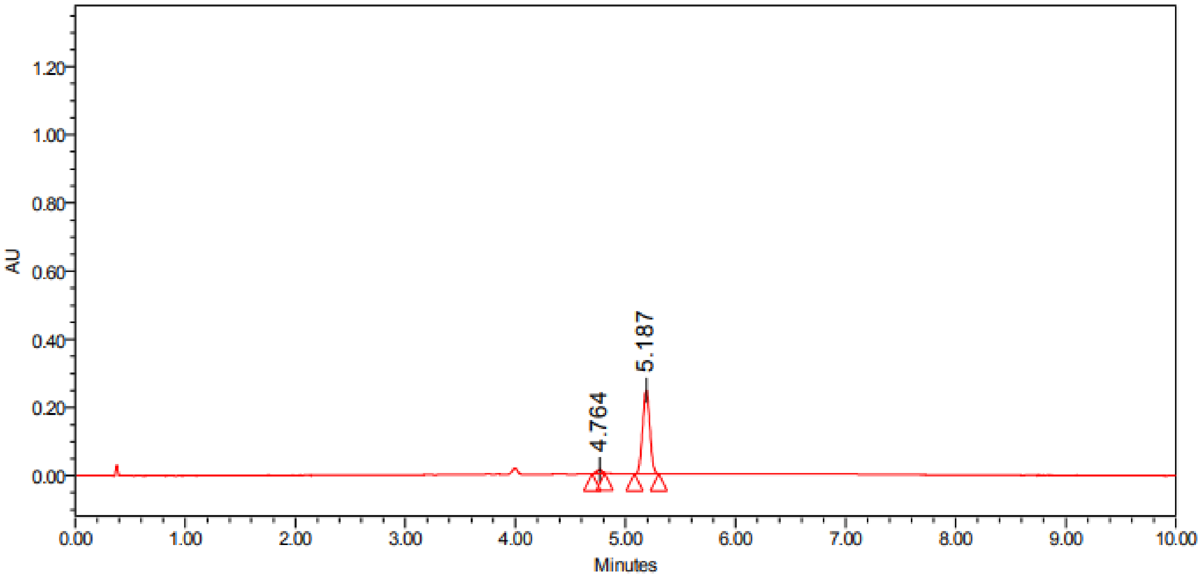

Peak Results

	RT	% Area
1	4.764	3.25
2	5.187	96.75

4-((2*R*,3*R*)-2-(3-chlorophenyl)-3-(3-formylfuran-2-yl)-3-phenylpropyl)-2-oxo-2*H*-chromene-3-carbonitrile major – 3d

Racemic sample

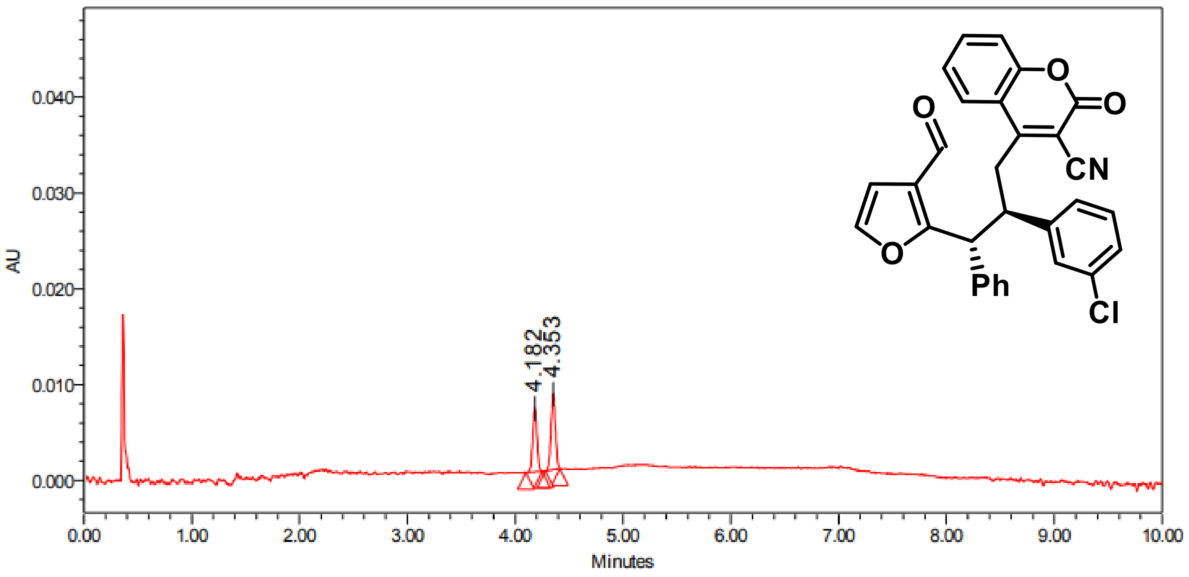

Peak Results

	RT	% Area
1	4.182	44.83
2	4.353	55.17

Enantiomerically enriched sample

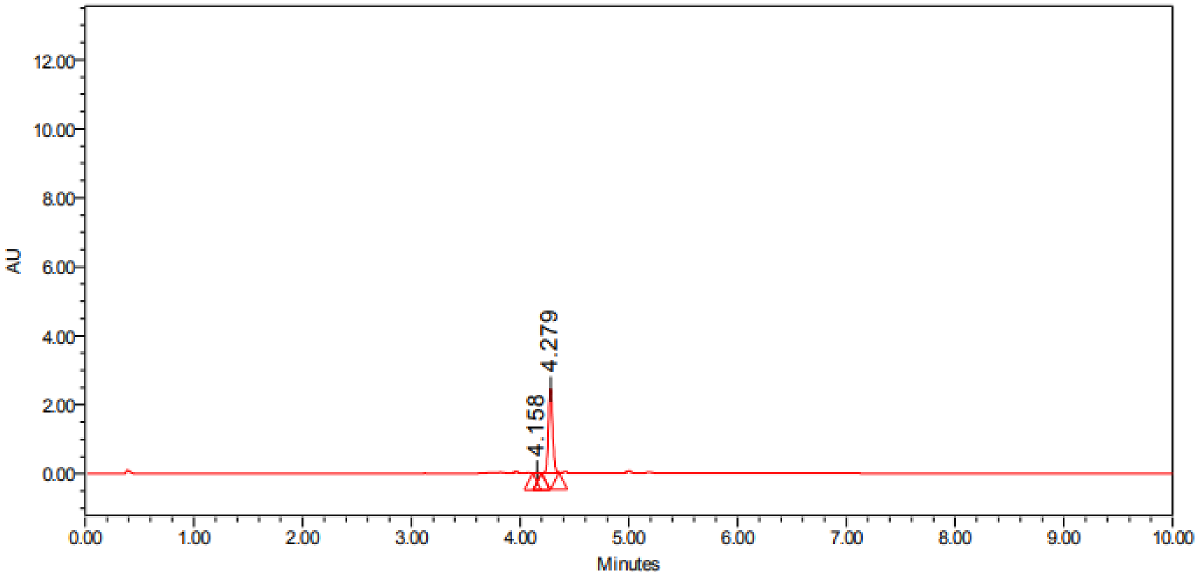

Peak Results

	RT	% Area
1	4.158	0.48
2	4.279	99.52

4-((2*S*,3*R*)-2-(3-chlorophenyl)-3-(3-formylfuran-2-yl)-3-phenylpropyl)-2-oxo-2*H*-chromene-3-carbonitrile minor – 3d

Racemic sample

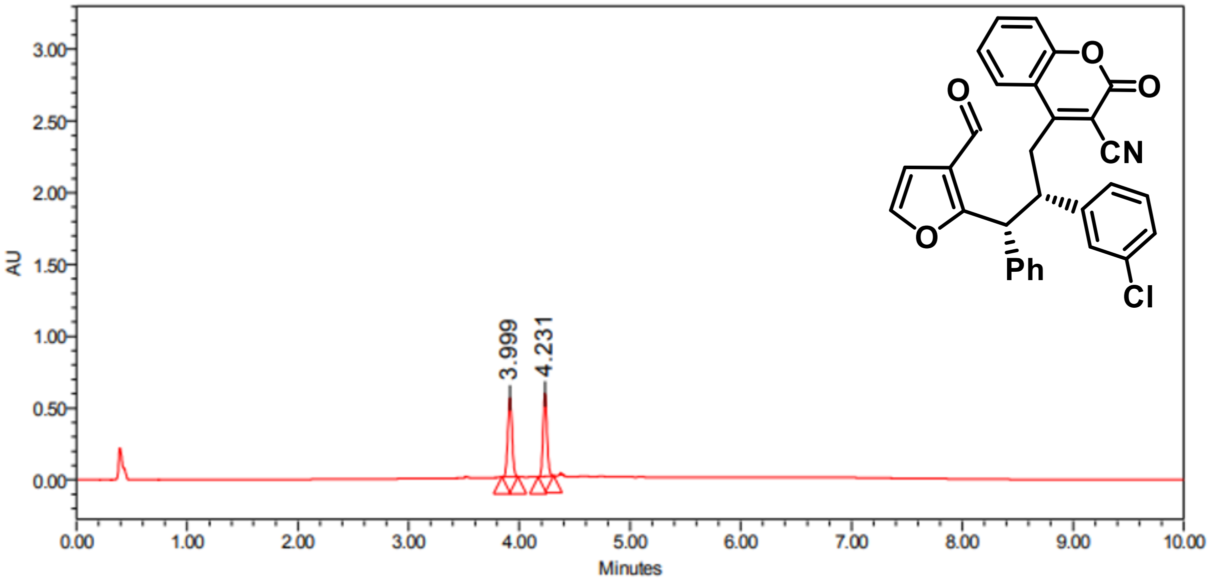

Peak Results

	RT	% Area
1	3.999	50.75
2	4.231	49.25

Enantiomerically enriched sample

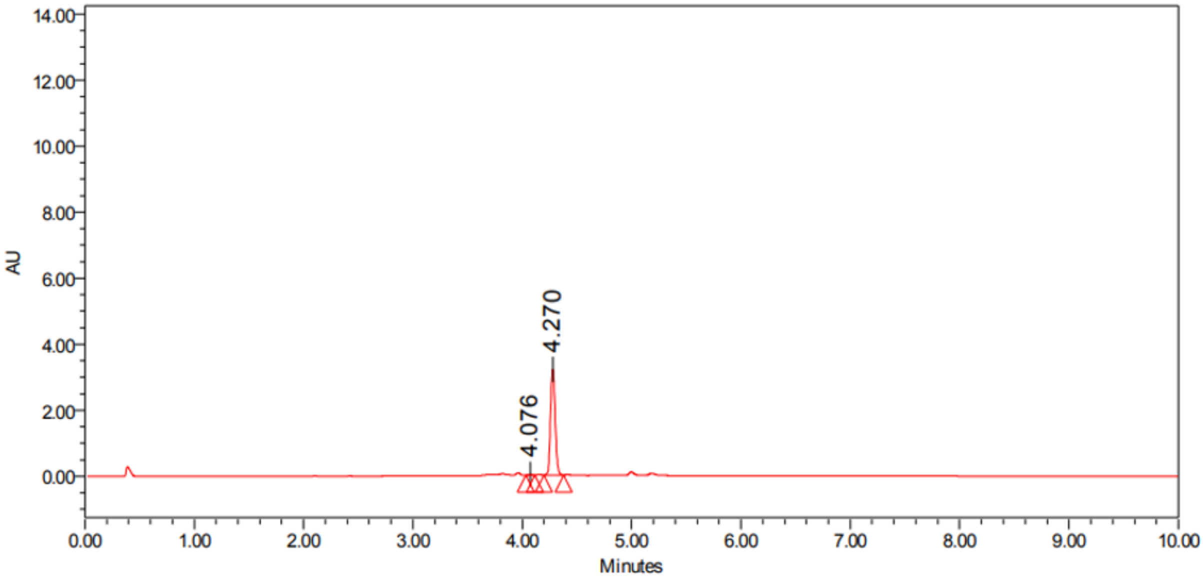

Peak Results

	RT	% Area
1	4.076	0.64
2	4.270	99.36

4-((2*R*,3*R*)-2-(4-chlorophenyl)-3-(3-formylfuran-2-yl)-3-phenylpropyl)-2-oxo-2*H*-chromene-3-carbonitrile major – 3e

Racemic sample

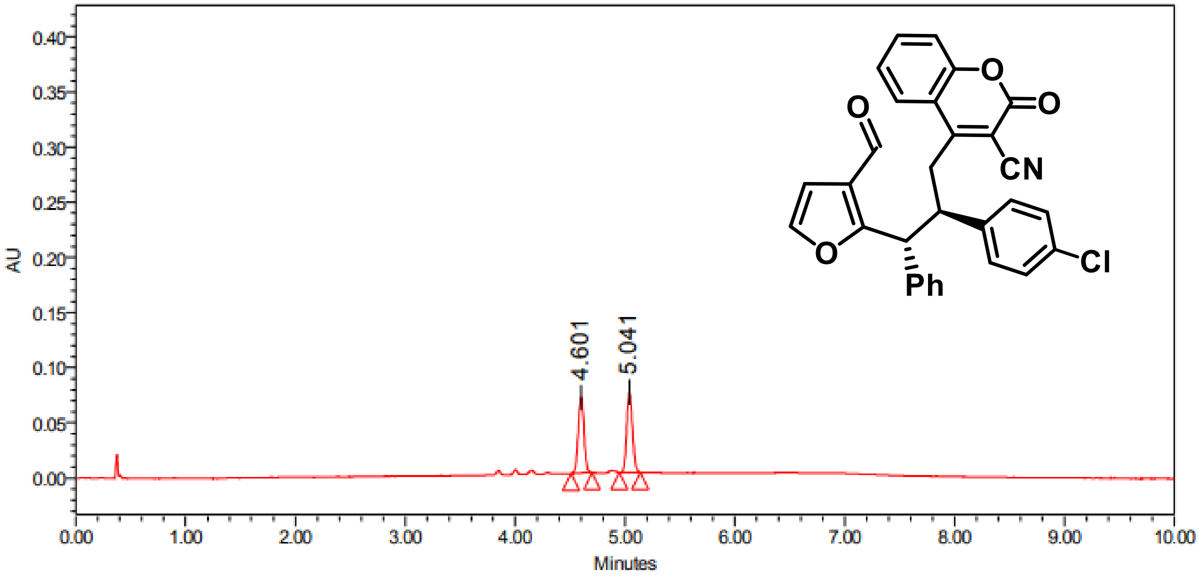

Peak Results

	RT	% Area
1	4.601	48.91
2	5.041	51.09

Enantiomerically enriched sample

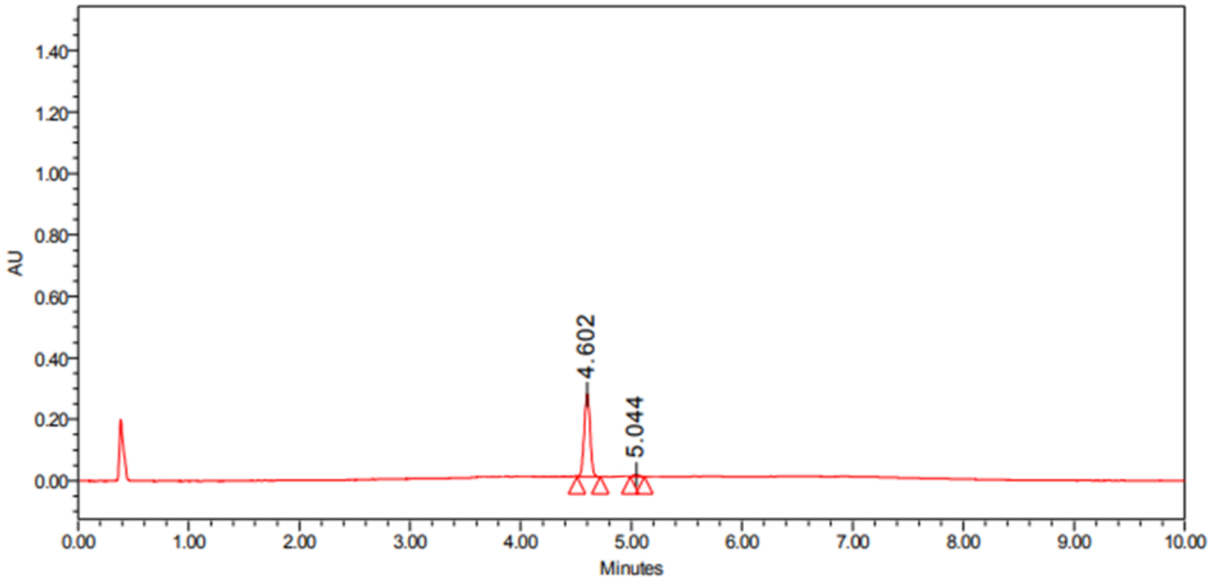

Peak Results

	RT	% Area
1	4.602	98.18
2	5.044	1.82

4-((2*S*,3*R*)-2-(4-chlorophenyl)-3-(3-formylfuran-2-yl)-3-phenylpropyl)-2-oxo-2*H*-chromene-3-carbonitrile minor – 3e

Racemic sample

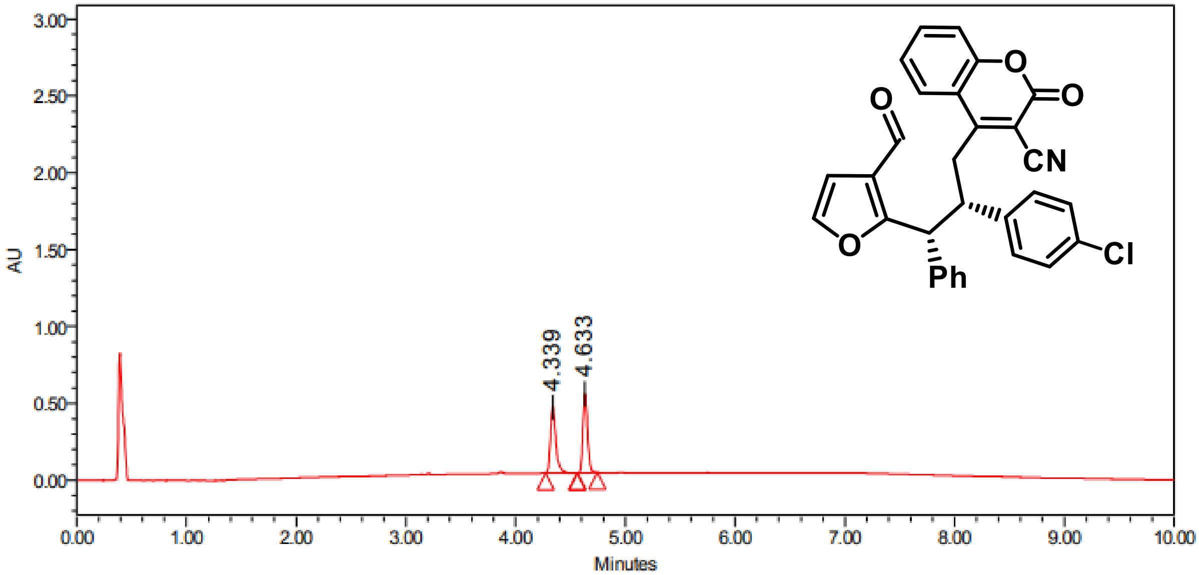

Peak Results

	RT	% Area
1	4.339	49.16
2	4.633	50.84

Enantiomerically enriched sample

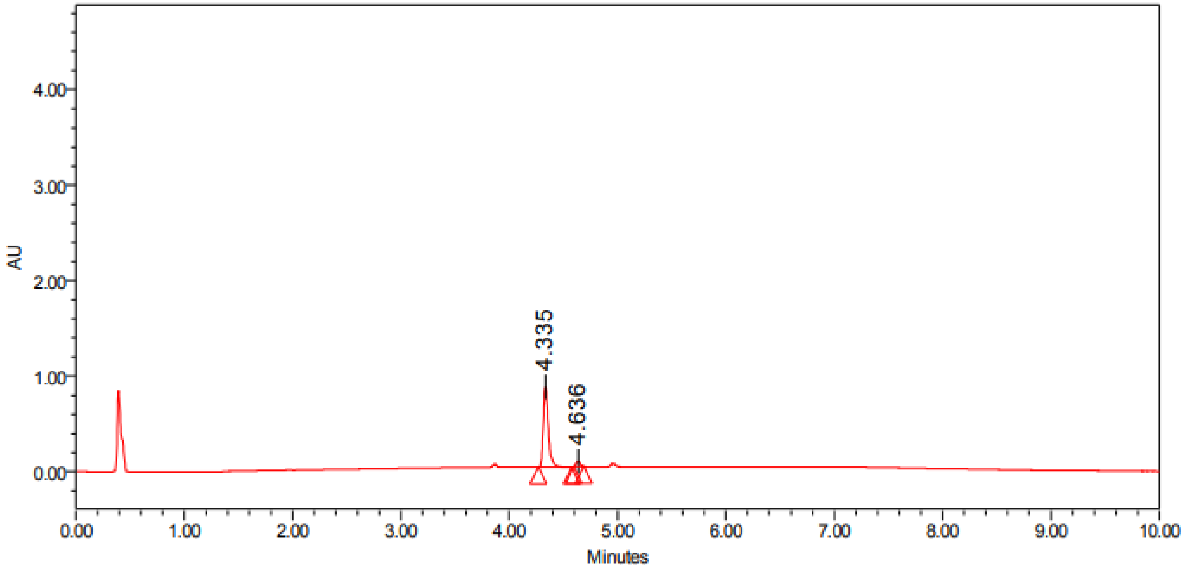

Peak Results

	RT	% Area
1	4.335	95.06
2	4.636	4.94

4-((2*R*,3*R*)-2-(4-bromophenyl)-3-(3-formylfuran-2-yl)-3-phenylpropyl)-2-oxo-2*H*-chromene-3-carbonitrile major – 3f

Racemic sample

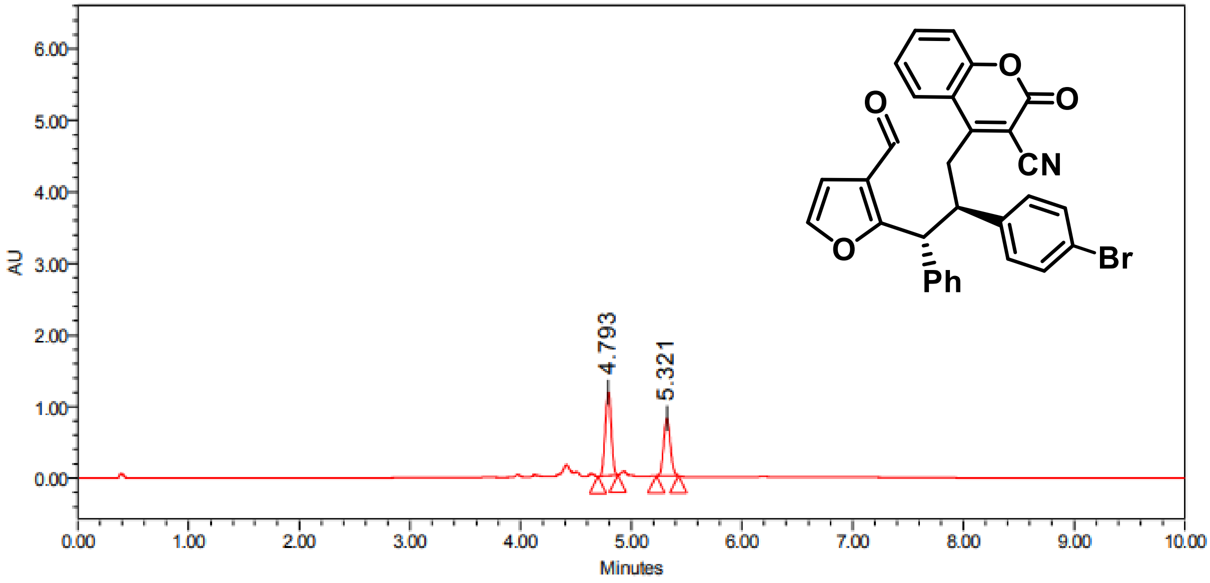

Peak Results

	RT	% Area
1	4.793	56.18
2	5.321	43.82

Enantiomerically enriched sample

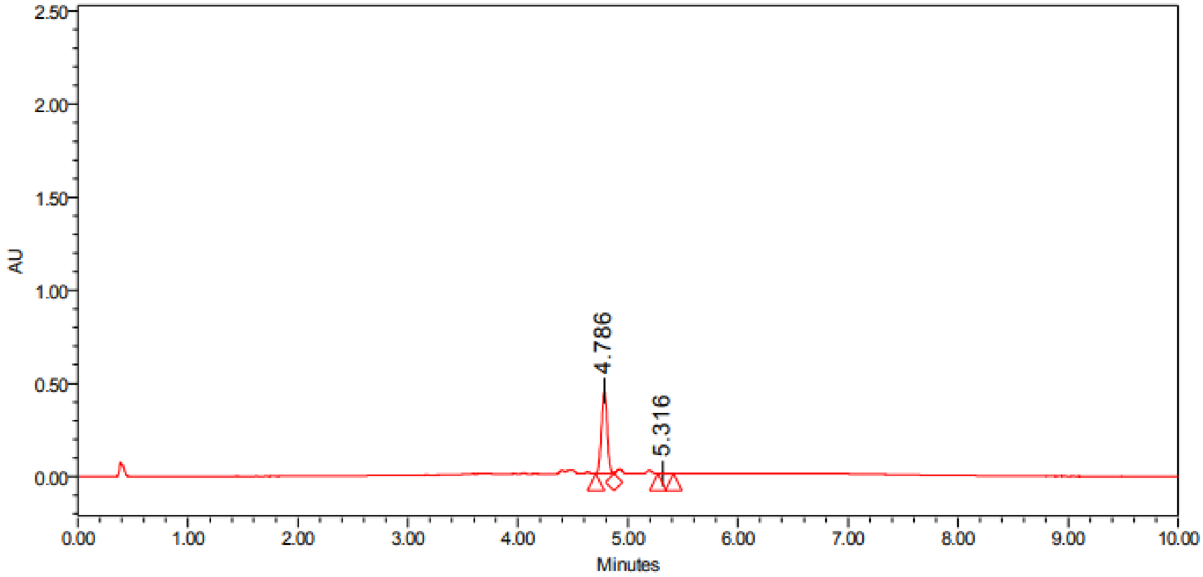

Peak Results

	RT	% Area
1	4.786	99.44
2	5.316	0.56

4-((2*S*,3*R*)-2-(4-bromophenyl)-3-(3-formylfuran-2-yl)-3-phenylpropyl)-2-oxo-2*H*-chromene-3-carbonitrile minor – 3f

Racemic sample

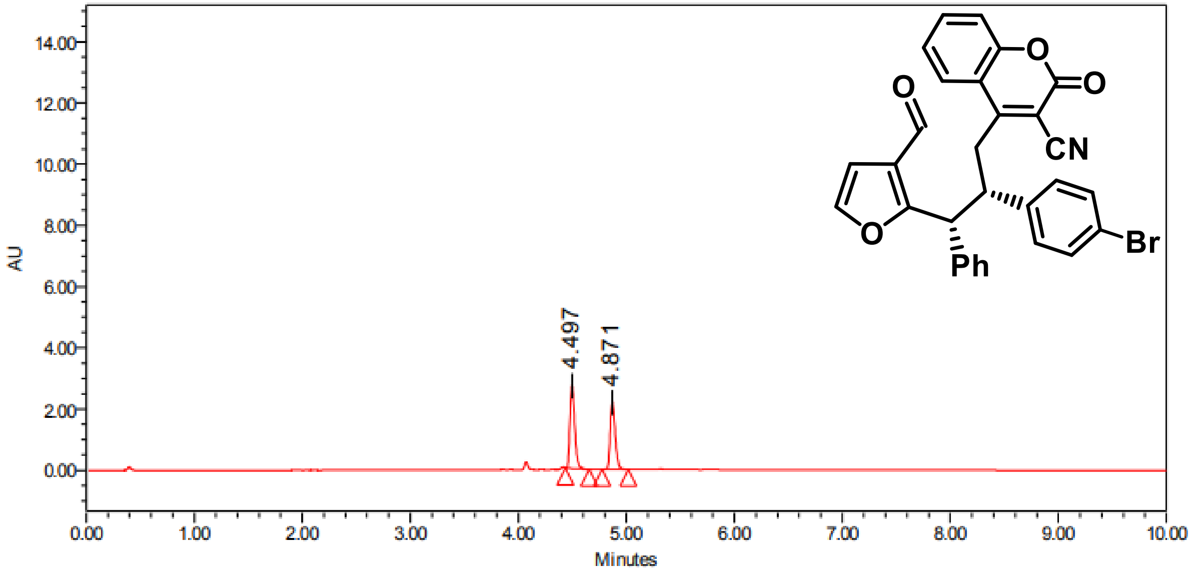

Peak Results

	RT	% Area
1	4.497	54.50
2	4.871	45.50

Enantiomerically enriched sample

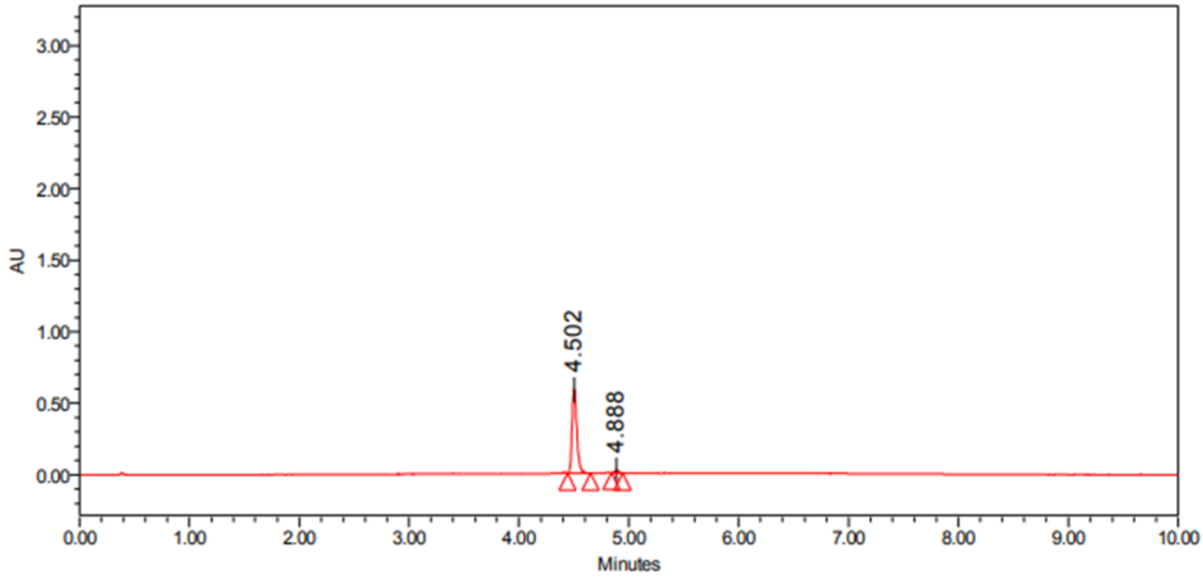

Peak Results

	RT	% Area
1	4.502	97.17
2	4.888	2.83

4-((2*R*,3*R*)-3-(3-formylfuran-2-yl)-3-phenyl-2-(4-(trifluoromethyl)phenyl)propyl)-2-oxo-2*H*-chromene-3-carbonitrile major – 3g

Racemic sample

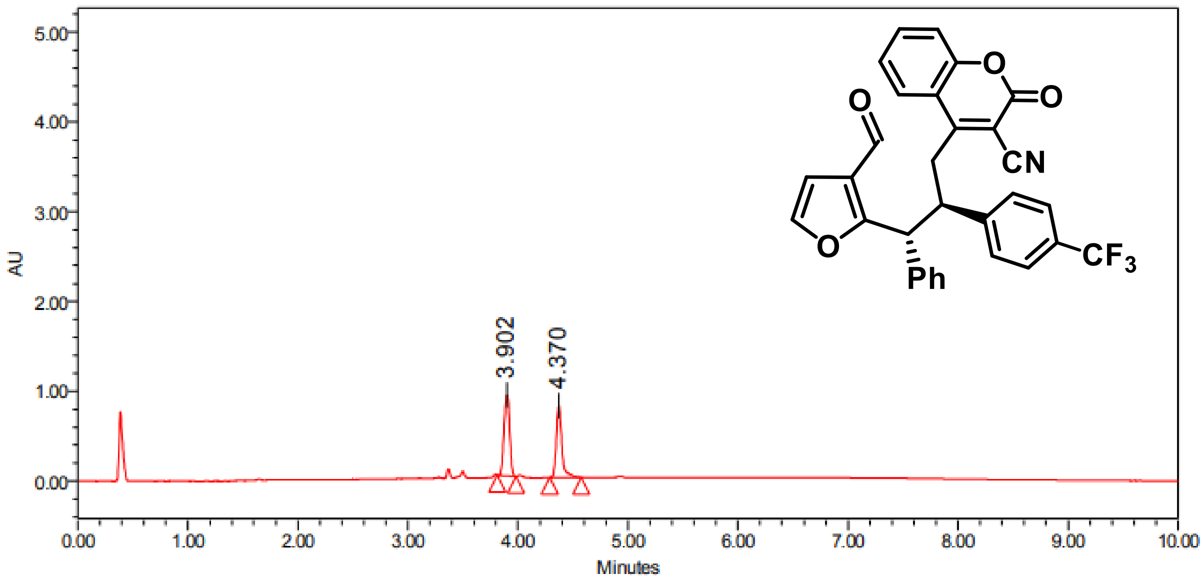

Peak Results

	RT	% Area
1	3.902	52.65
2	4.370	47.35

Enantiomerically enriched sample

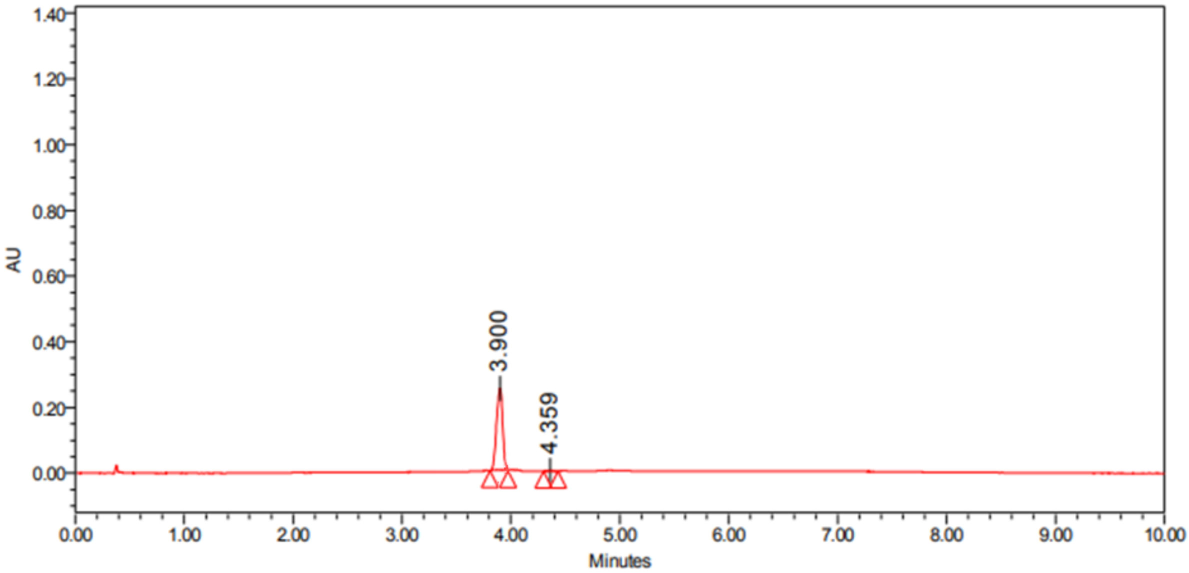

Peak Results

	RT	% Area
1	3.900	99.05
2	4.359	0.95

4-((2*S*,3*R*)-3-(3-formylfuran-2-yl)-3-phenyl-2-(4-(trifluoromethyl)phenyl)propyl)-2-oxo-2*H*-chromene-3-carbonitrile minor – 3g

Racemic sample

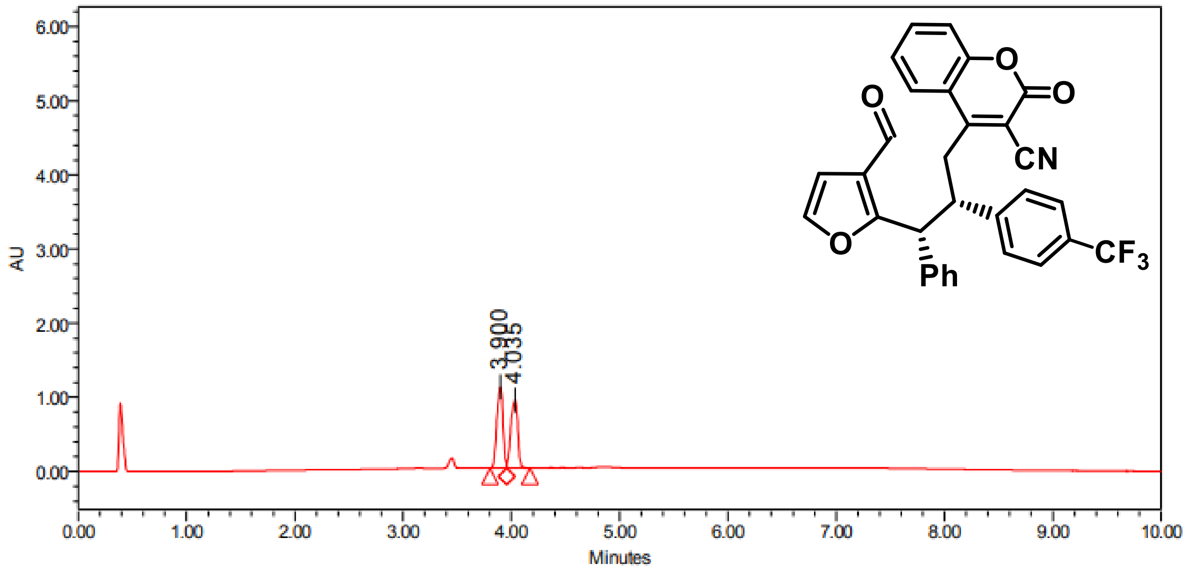

Peak Results

	RT	% Area
1	3.900	50.64
2	4.035	49.36

Enantiomerically enriched sample

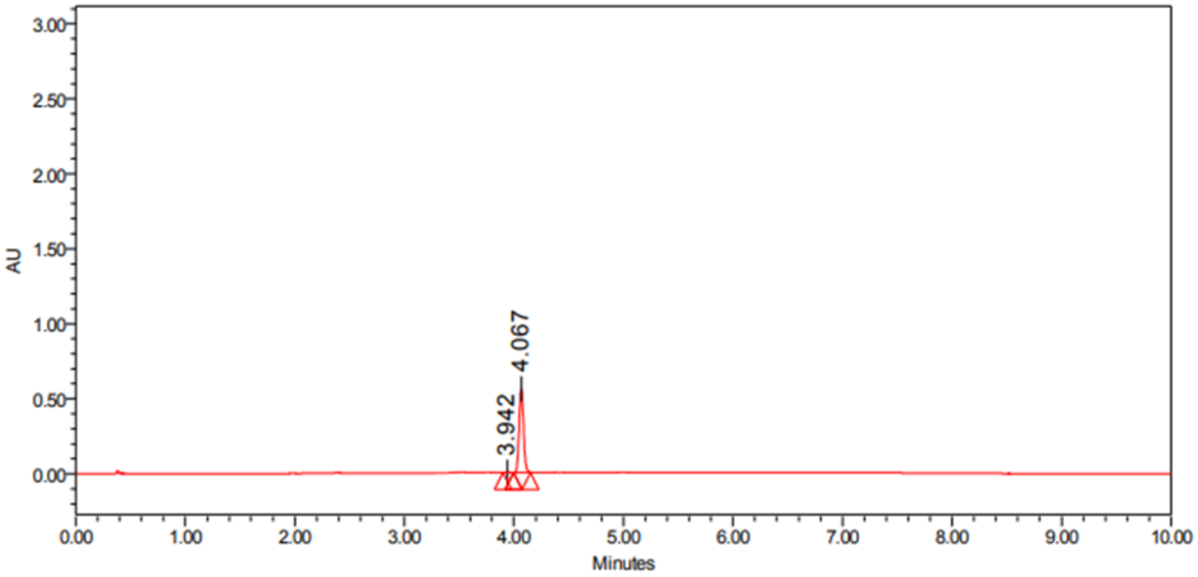

Peak Results

	RT	% Area
1	3.942	0.56
2	4.067	99.44

4-((2*R*,3*R*)-3-(3-formylfuran-2-yl)-3-phenyl-2-(*m*-tolyl)propyl)-2-oxo-2*H*-chromene-3-carbonitrile major – 3h

Racemic sample

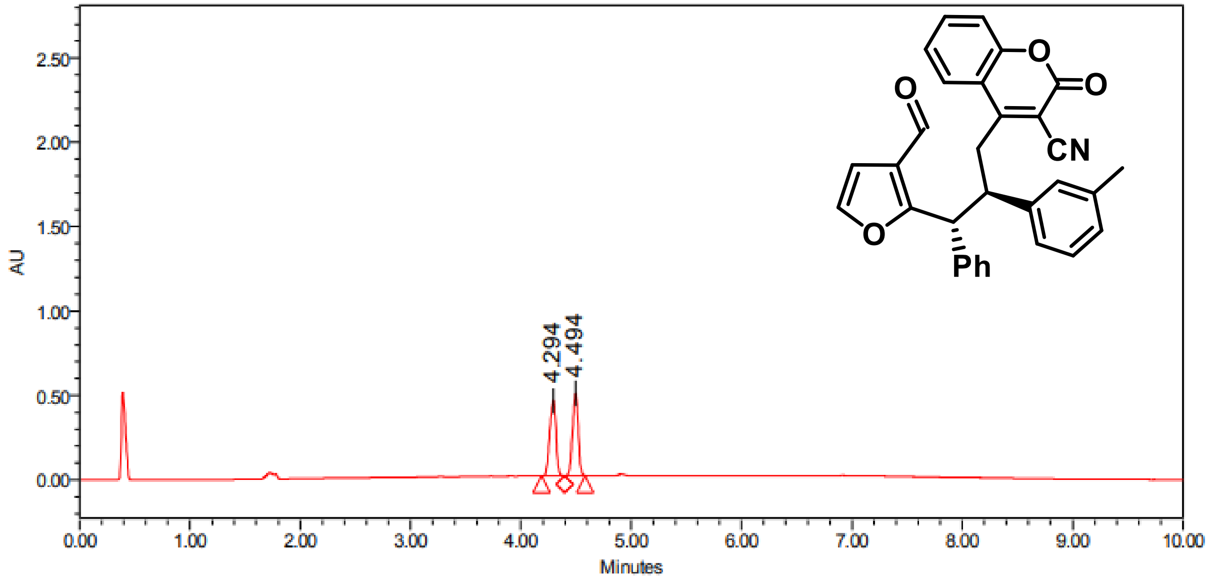

Peak Results

	RT	% Area
1	4.294	50.74
2	4.494	49.26

Enantiomerically enriched sample

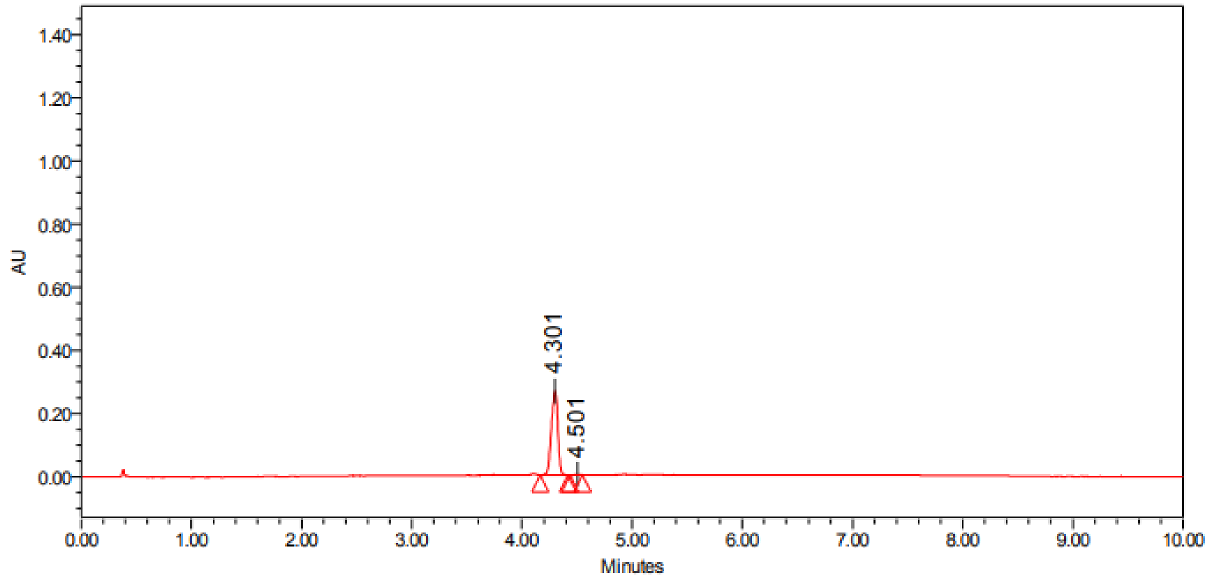

Peak Results

	RT	% Area
1	4.301	99.48
2	4.501	0.52

4-((2*S*,3*R*)-3-(3-formylfuran-2-yl)-3-phenyl-2-(*m*-tolyl)propyl)-2-oxo-2*H*-chromene-3-carbonitrile minor – 3h

Racemic sample

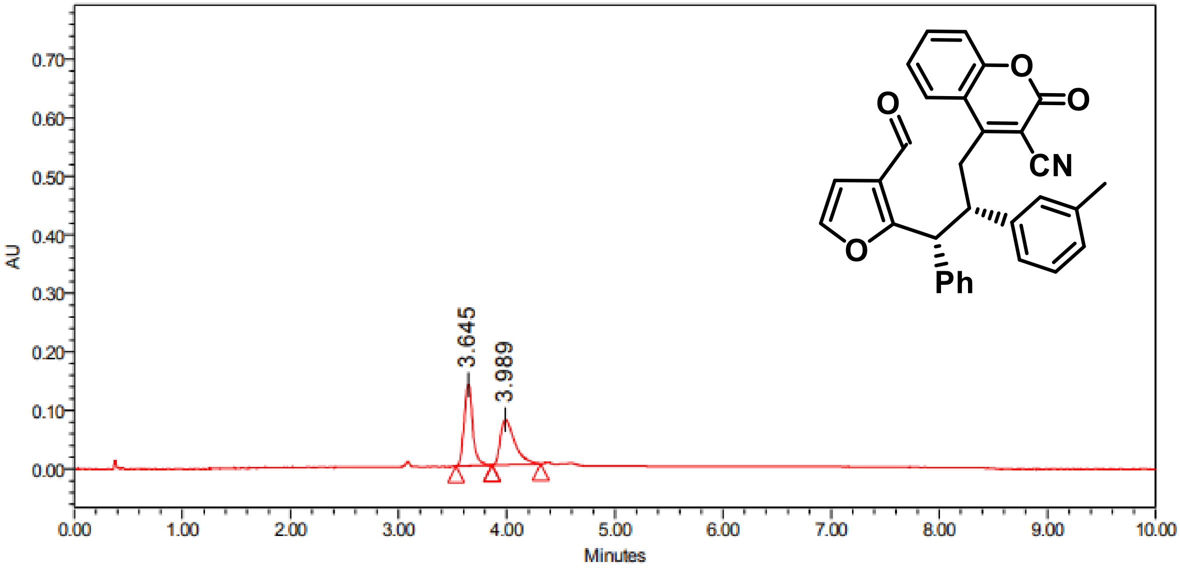

Peak Results

	RT	% Area
1	3.645	51.77
2	3.989	48.23

Enantiomerically enriched sample

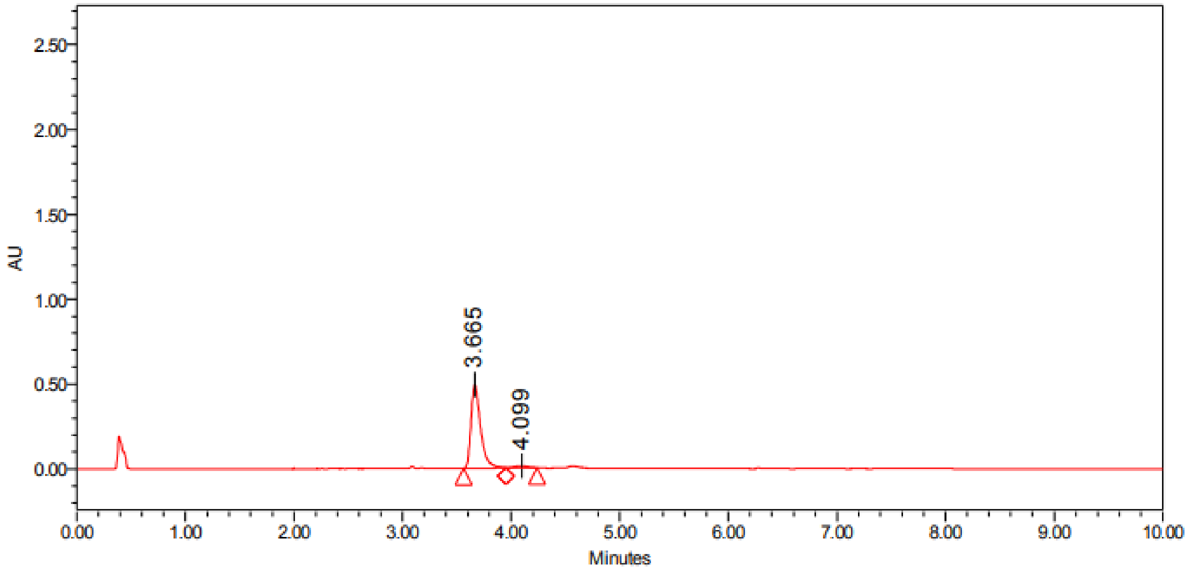

Peak Results

	RT	% Area
1	3.665	97.12
2	4.099	2.88

4-((2*R*,3*R*)-3-(3-formylfuran-2-yl)-3-phenyl-2-(*p*-tolyl)propyl)-2-oxo-2*H*-chromene-3-carbonitrile major – 3i

Racemic sample

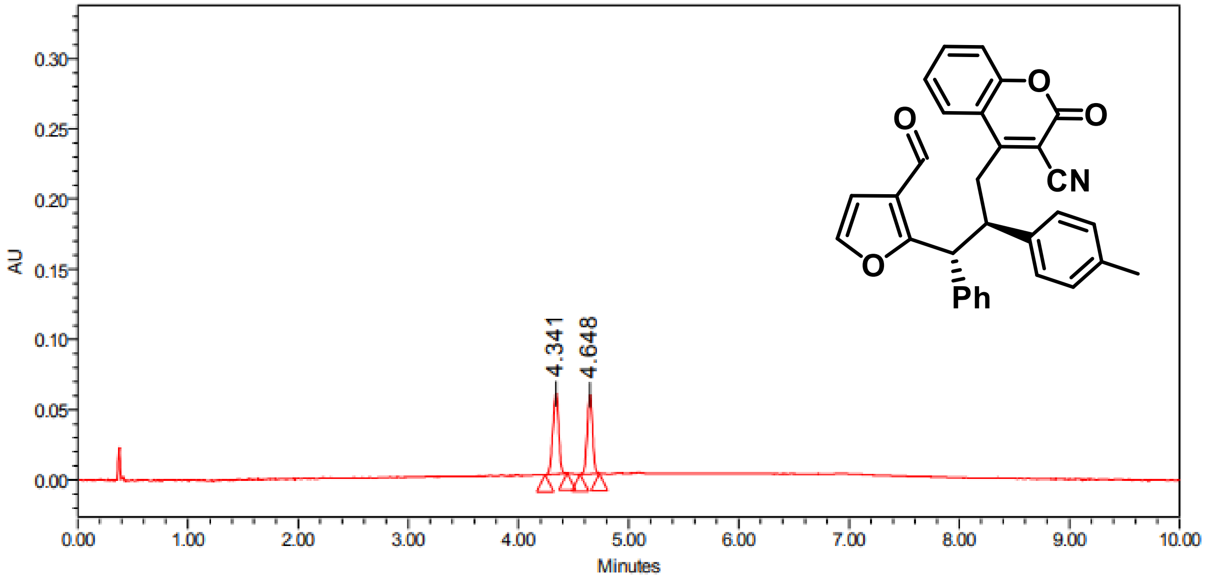

Peak Results

	RT	% Area
1	4.341	52.92
2	4.648	47.08

Enantiomerically enriched sample

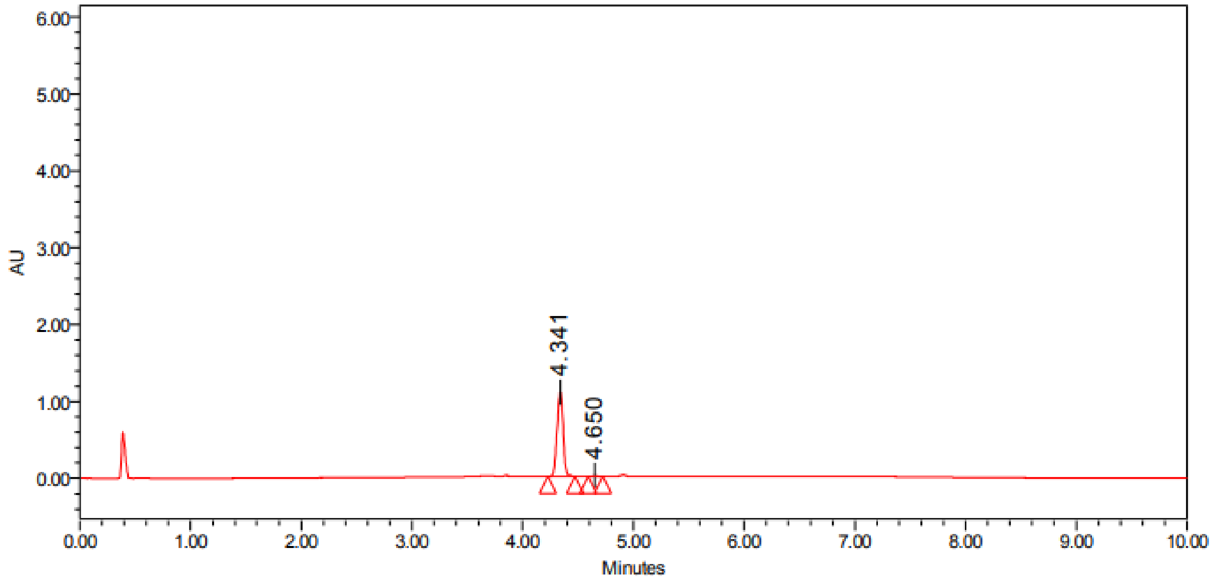

Peak Results

	RT	% Area
1	4.341	99.27
2	4.650	0.73

4-((2*S*,3*R*)-3-(3-formylfuran-2-yl)-3-phenyl-2-(*p*-tolyl)propyl)-2-oxo-2*H*-chromene-3-carbonitrile minor – 3i

Racemic sample

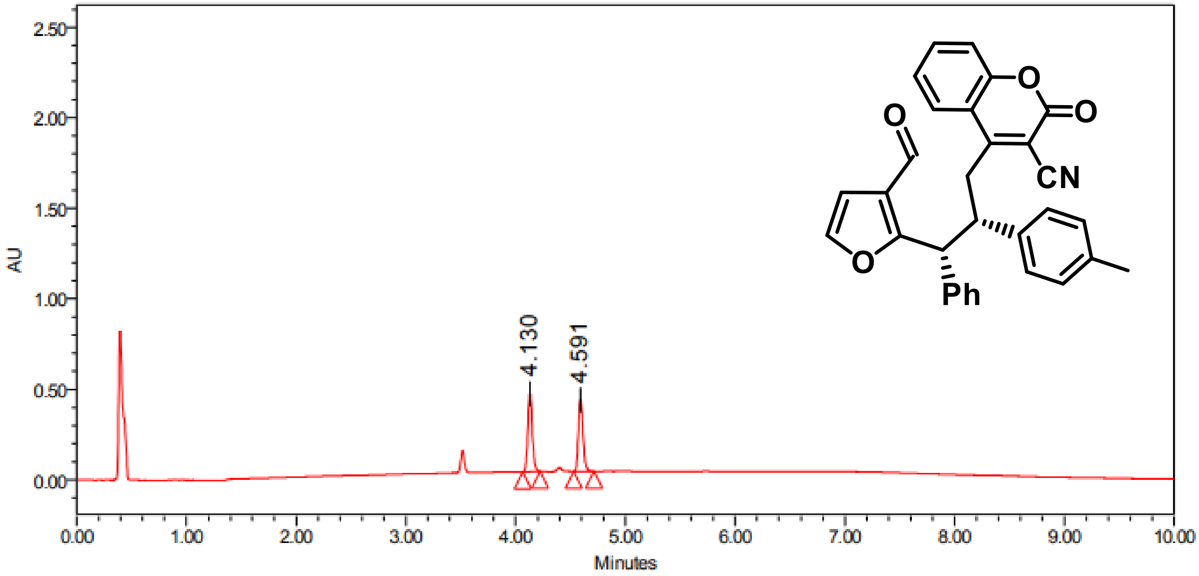

Peak Results

	RT	% Area
1	4.130	52.29
2	4.591	47.71

Enantiomerically enriched sample

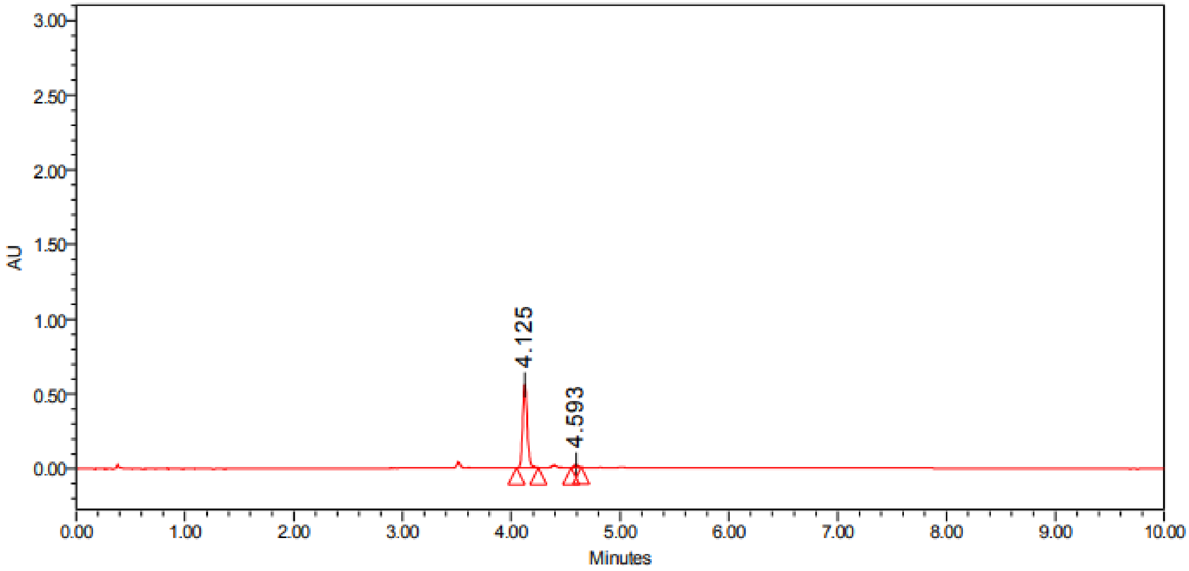

Peak Results

	RT	% Area
1	4.125	97.01
2	4.593	2.99

4-((2*R*,3*R*)-3-(3-formylfuran-2-yl)-2-(4-methoxyphenyl)-3-phenylpropyl)-2-oxo-2*H*-chromene-3-carbonitrile major – 3j

Racemic sample

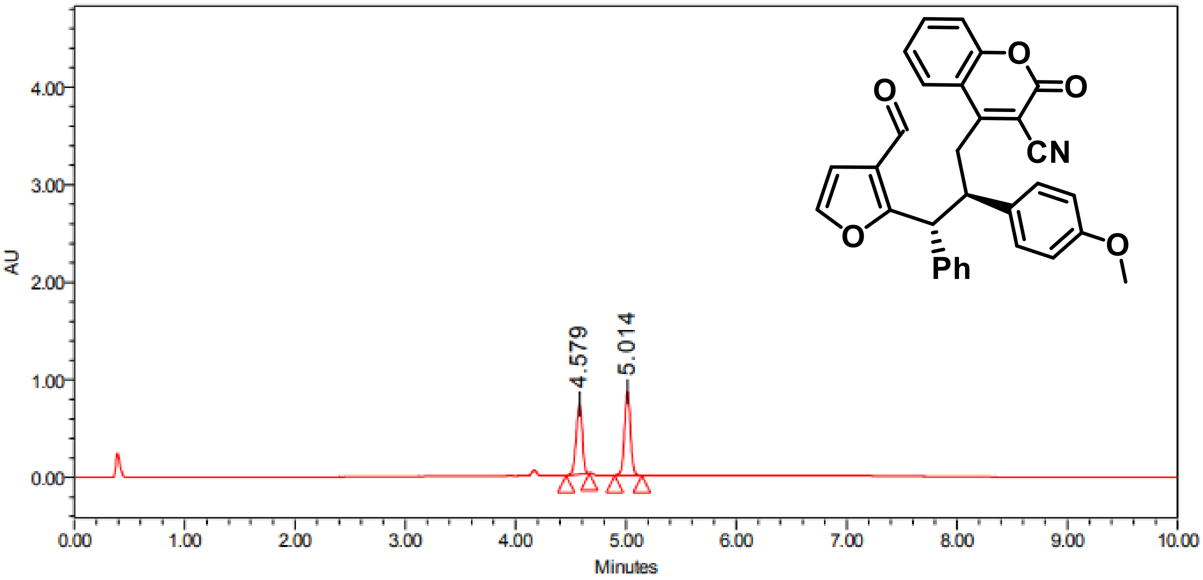

Peak Results

	RT	% Area
1	4.579	46.12
2	5.014	53.88

Enantiomerically enriched sample

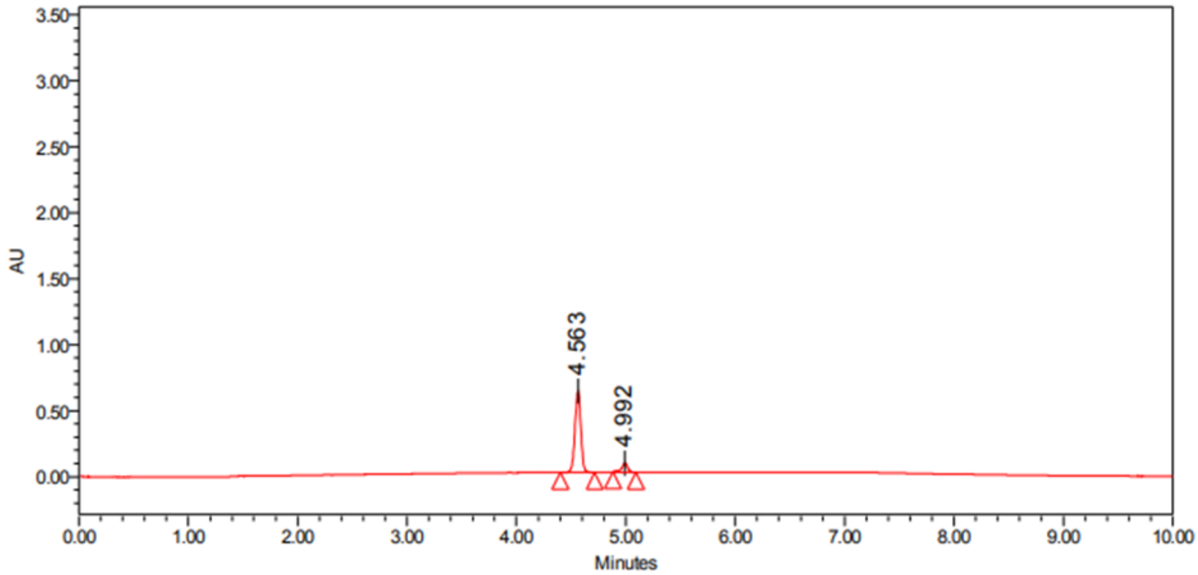

Peak Results

	RT	% Area
1	4.563	89.71
2	4.992	10.29

4-((2*S*,3*R*)-3-(3-formylfuran-2-yl)-2-(4-methoxyphenyl)-3-phenylpropyl)-2-oxo-2*H*-chromene-3-carbonitrile minor – 3j

Racemic sample

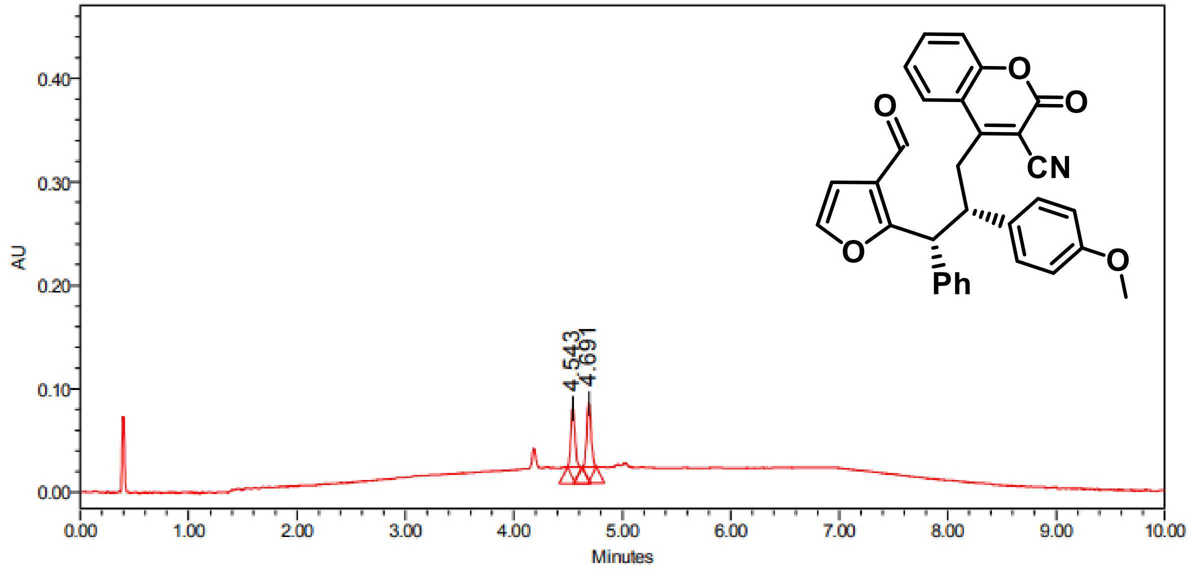

Peak Results

	RT	% Area
1	4.543	46.95
2	4.691	53.05

Enantiomerically enriched sample

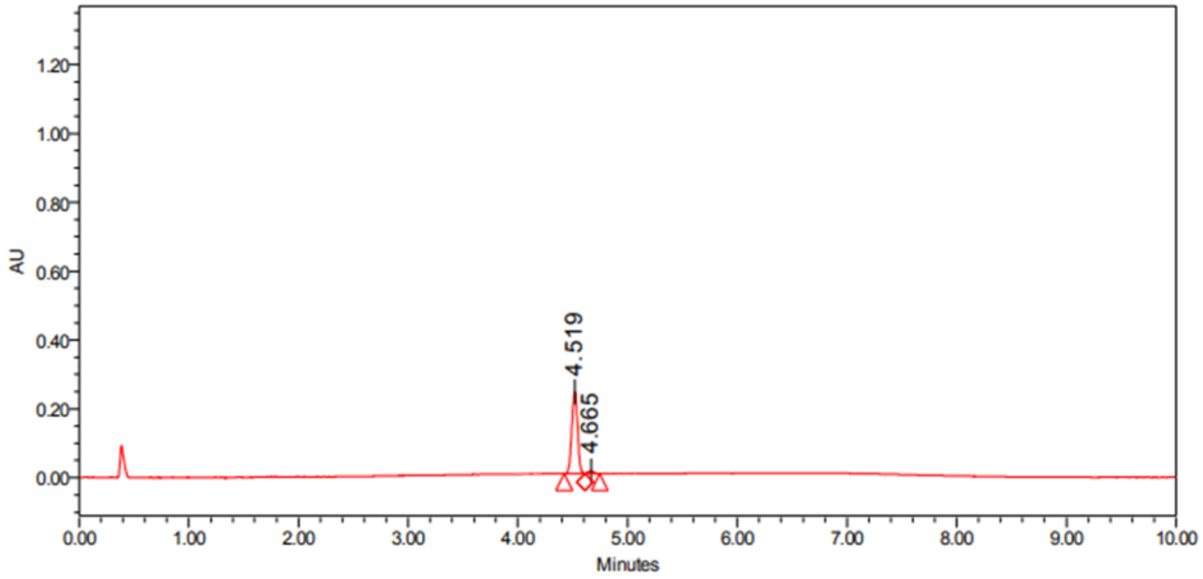

Peak Results

	RT	% Area
1	4.519	97.09
2	4.665	2.91

4-((2*R*,3*R*)-3-(3-formylfuran-2-yl)-2-(naphthalen-2-yl)-3-phenylpropyl)-2-oxo-2*H*-chromene-3-carbonitrile major – 3k

Racemic sample

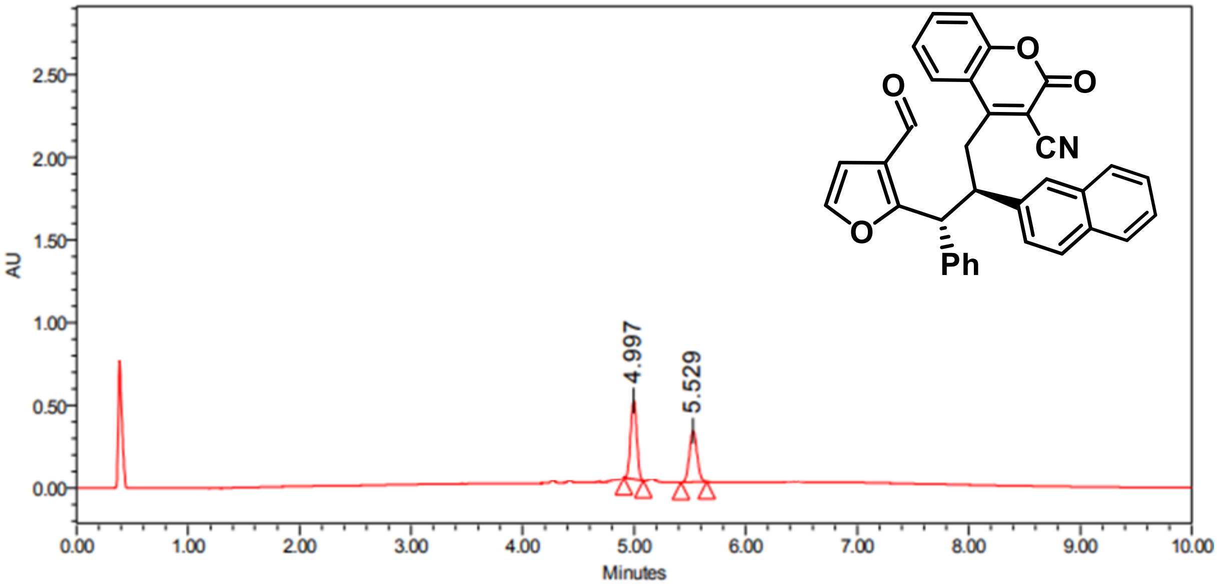

Peak Results

	RT	% Area
1	4.997	55.26
2	5.529	44.74

Enantiomerically enriched sample

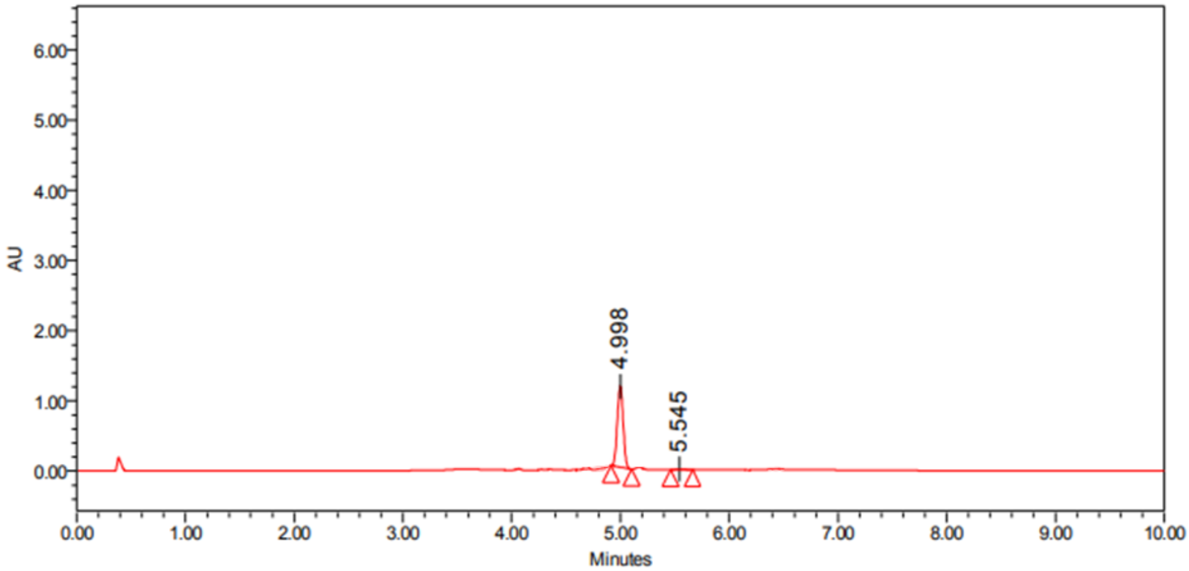

Peak Results

	RT	% Area
1	4.998	98.39
2	5.545	1.61

4-((2*S*,3*R*)-3-(3-formylfuran-2-yl)-2-(naphthalen-2-yl)-3-phenylpropyl)-2-oxo-2*H*-chromene-3-carbonitrile minor – 3k

Racemic sample

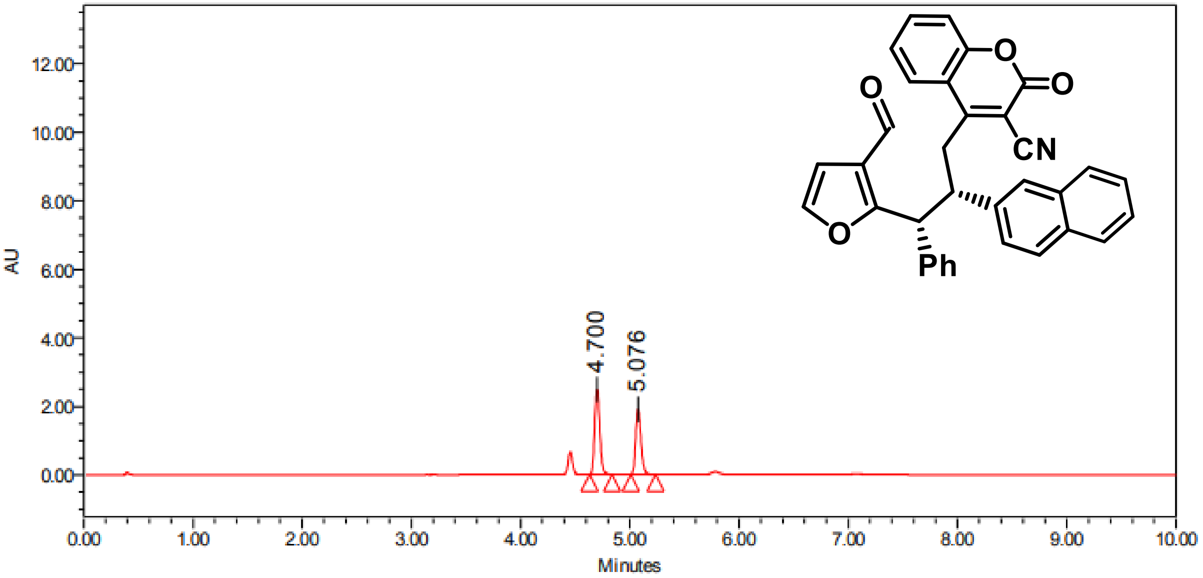

Peak Results

	RT	% Area
1	4.700	56.18
2	5.076	43.82

Enantiomerically enriched sample

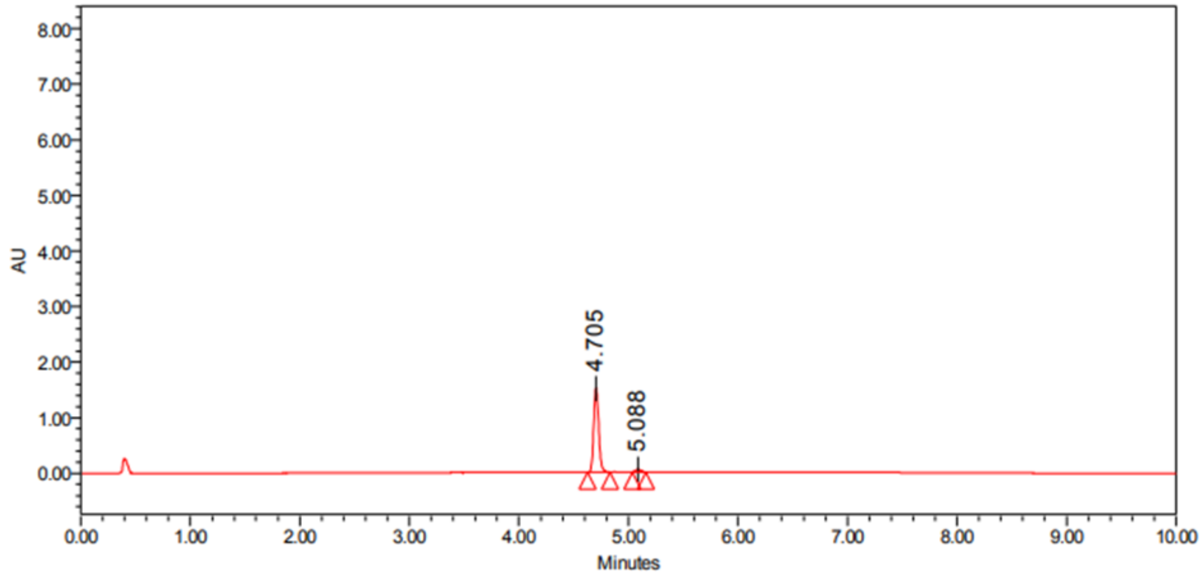

Peak Results

	RT	% Area
1	4.705	96.59
2	5.088	3.41

6-bromo-4-((2*R*,3*R*)-3-(3-formylfuran-2-yl)-2,3-diphenylpropyl)-2-oxo-2*H*-chromene-3-carbonitrile major – 3l

Racemic sample

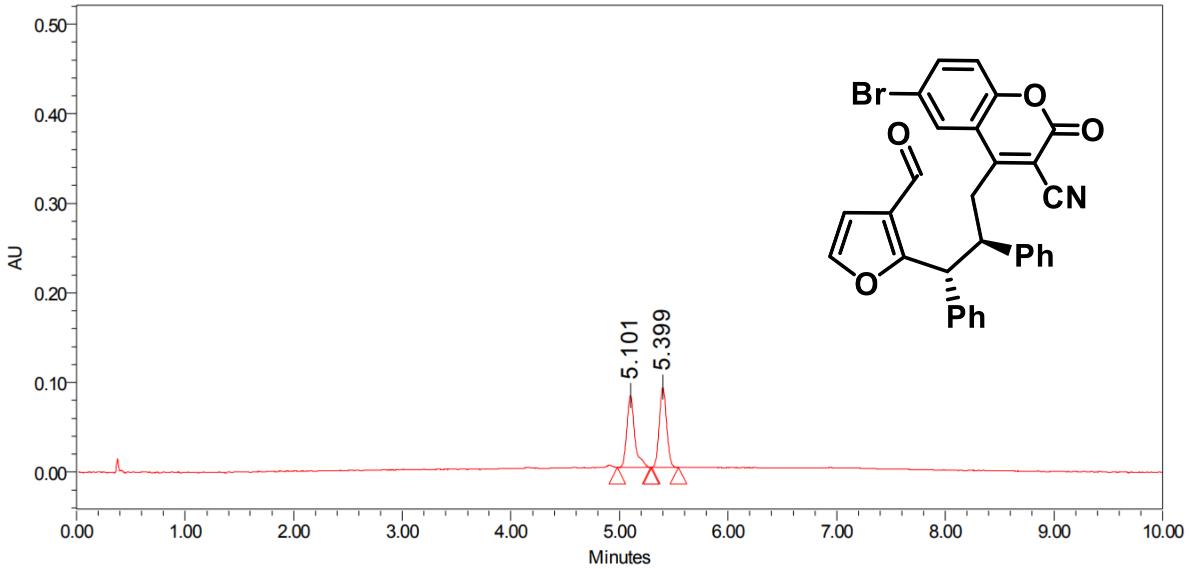

Peak Results

	RT	% Area
1	5.101	48.88
2	5.399	51.12

Enantiomerically enriched sample

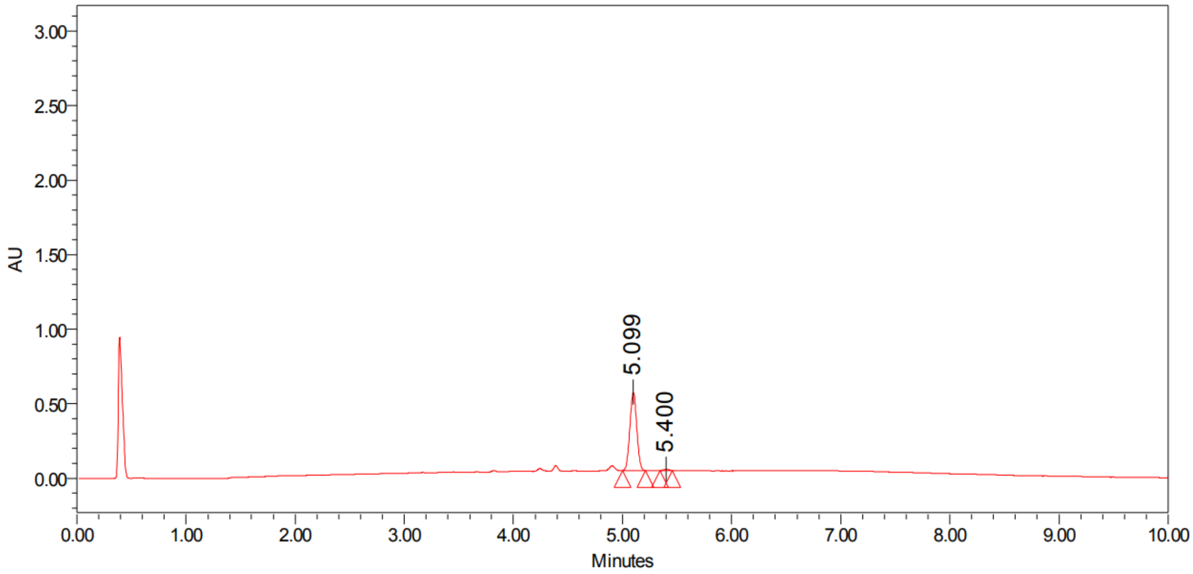

Peak Results

	RT	% Area
1	5.099	98.59
2	5.400	1.41

6-bromo-4-((2*S*,3*R*)-3-(3-formylfuran-2-yl)-2,3-diphenylpropyl)-2-oxo-2*H*-chromene-3-carbonitrile minor – 3l

Racemic sample

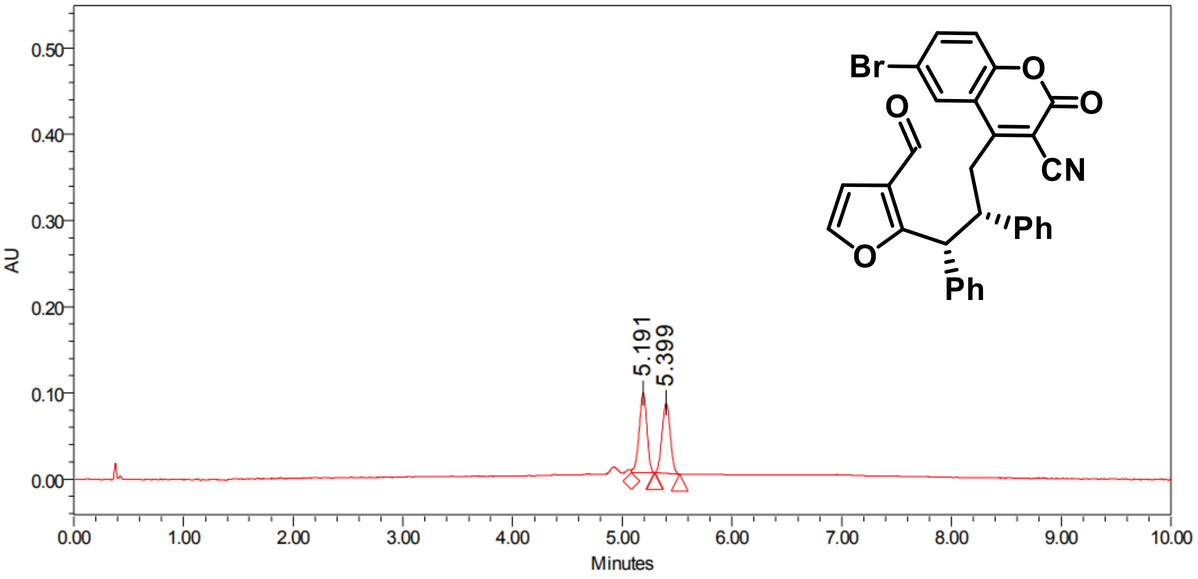

Peak Results

	RT	% Area
1	5.191	51.62
2	5.399	48.38

Enantiomerically enriched sample

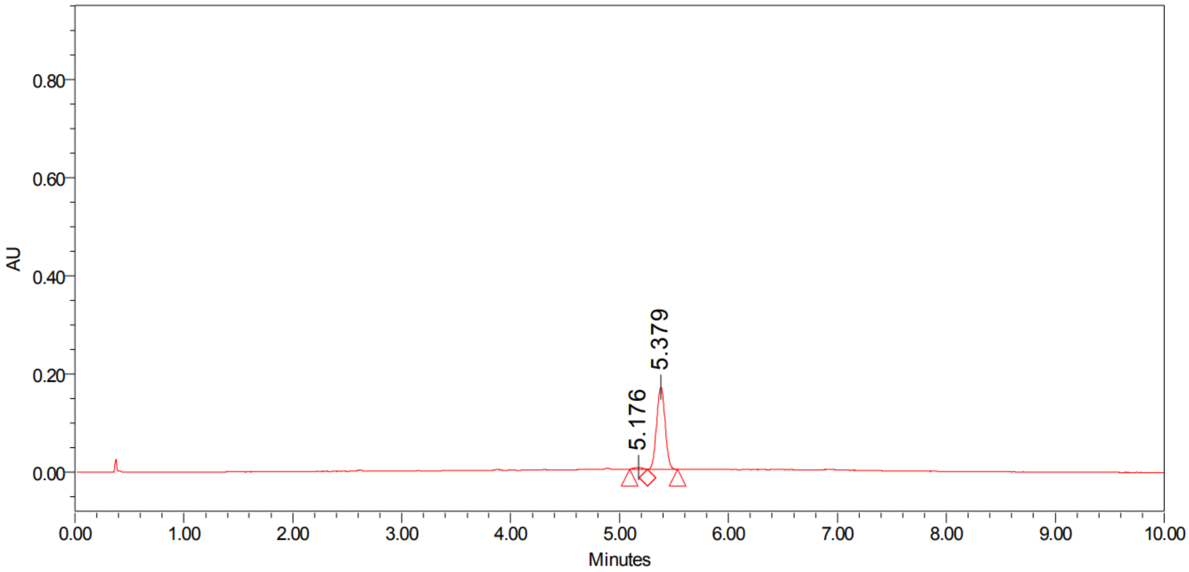

Peak Results

	RT	% Area
1	5.176	1.72
2	5.379	98.28

4-((2*R*,3*R*)-3-(3-formylfuran-2-yl)-2,3-diphenylpropyl)-7-methoxy-2-oxo-2*H*-chromene-3-carbonitrile major – 3m

Racemic sample

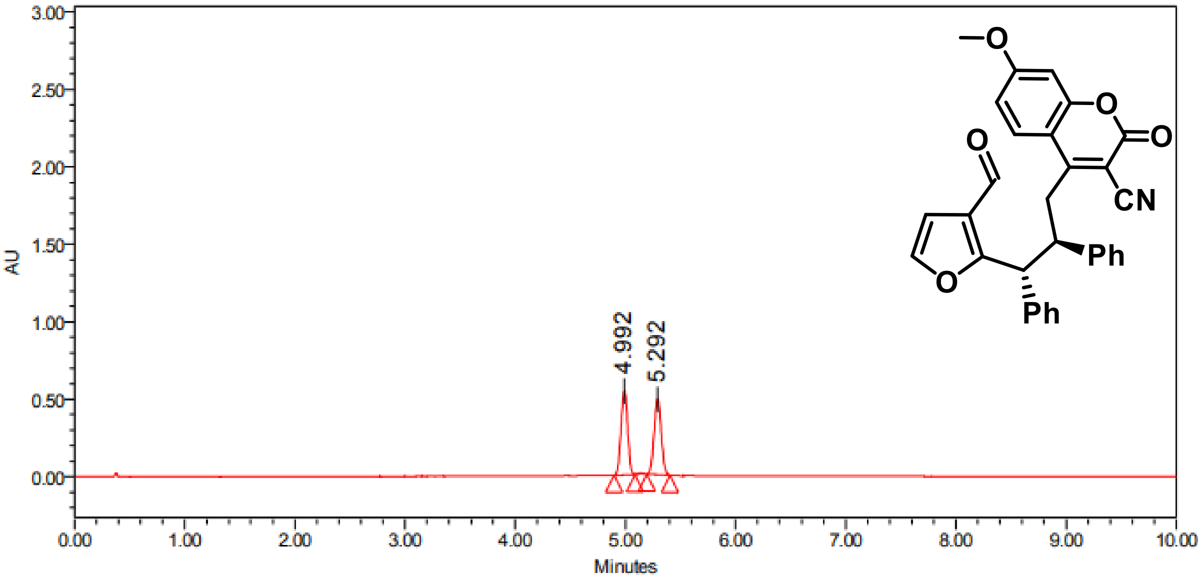

Peak Results

	RT	% Area
1	4.992	50.95
2	5.292	49.05

Enantiomerically enriched sample

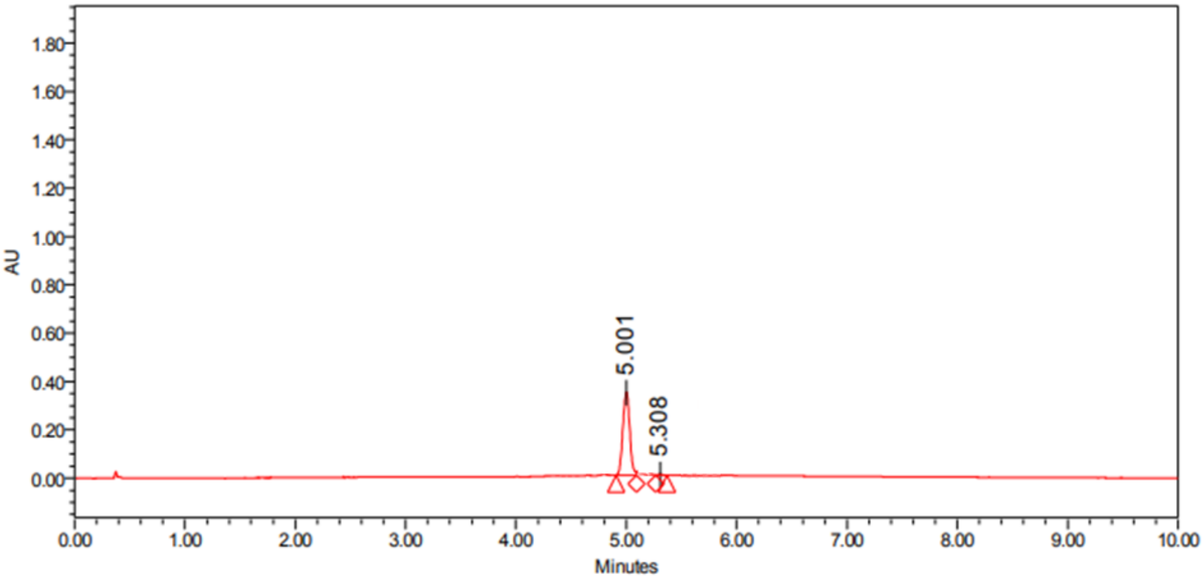

Peak Results

	RT	% Area
1	5.001	98.89
2	5.308	1.11

4-((2*S*,3*R*)-3-(3-formylfuran-2-yl)-2,3-diphenylpropyl)-7-methoxy-2-oxo-2*H*-chromene-3-carbonitrile minor – 3m

Racemic sample

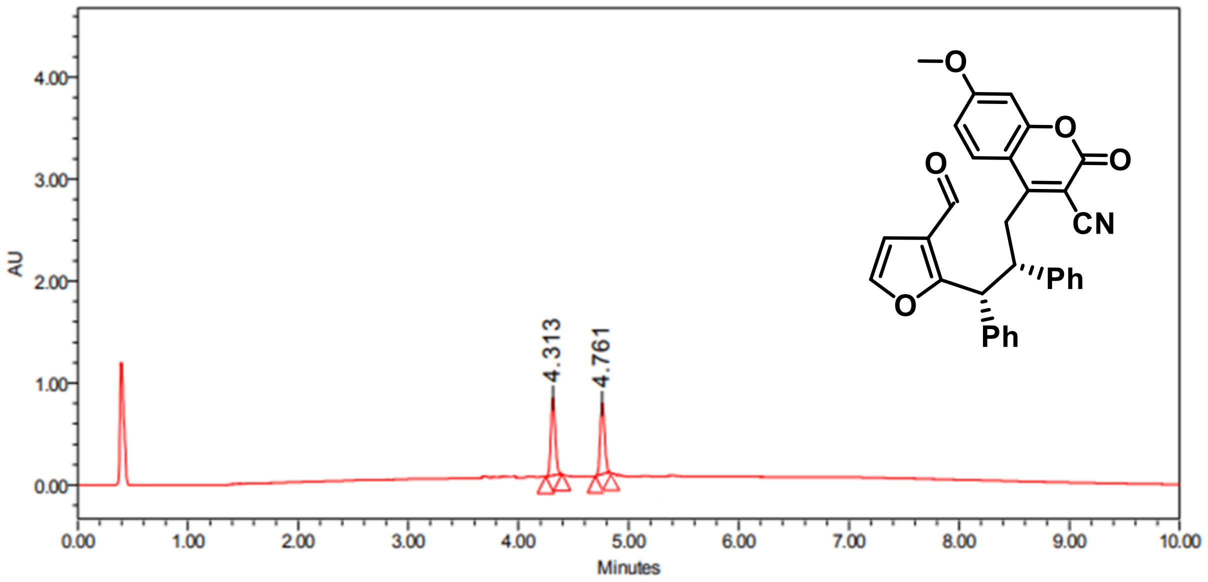

Peak Results

	RT	% Area
1	4.313	51.80
2	4.761	48.20

Enantiomerically enriched sample

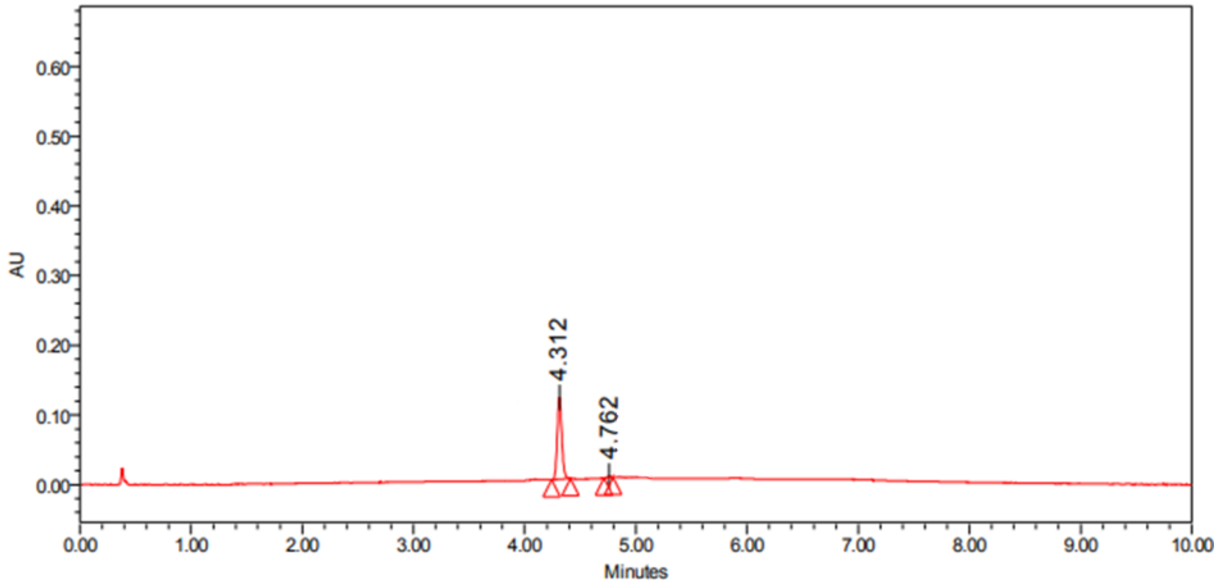

Peak Results

	RT	% Area
1	4.312	98.59
2	4.762	1.41

4-((*R*)-2-((*R*)-(3-formylfuran-2-yl)(phenyl)methyl)hexyl)-2-oxo-*2H*-chromene-3-carbonitrile major – 3n

Racemic sample

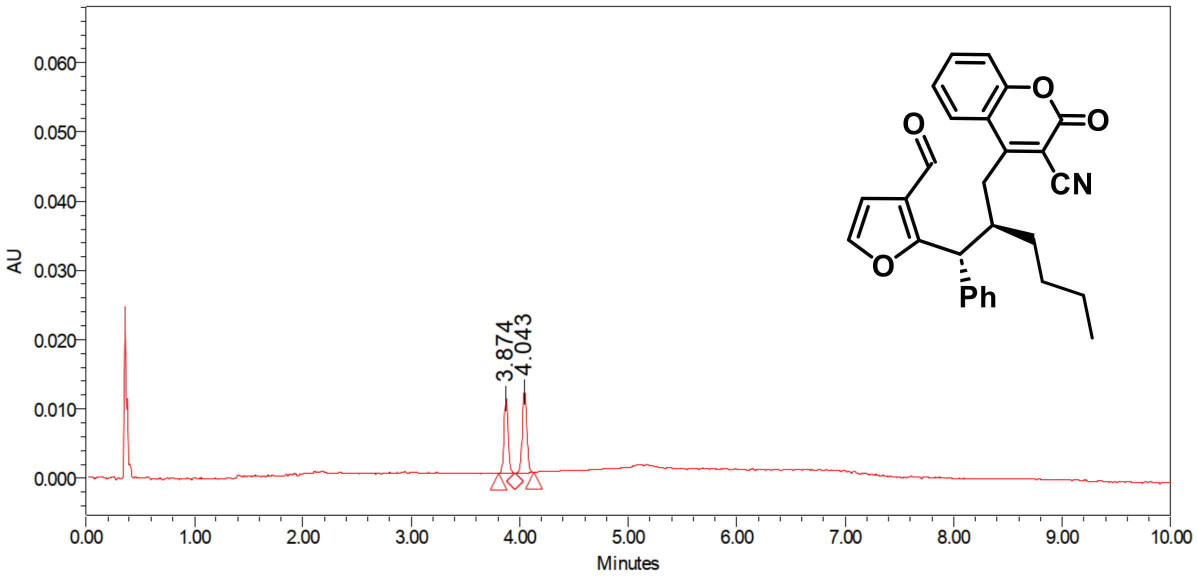

Peak Results

	RT	% Area
1	3.874	46.53
2	4.043	53.47

Enantiomerically enriched sample

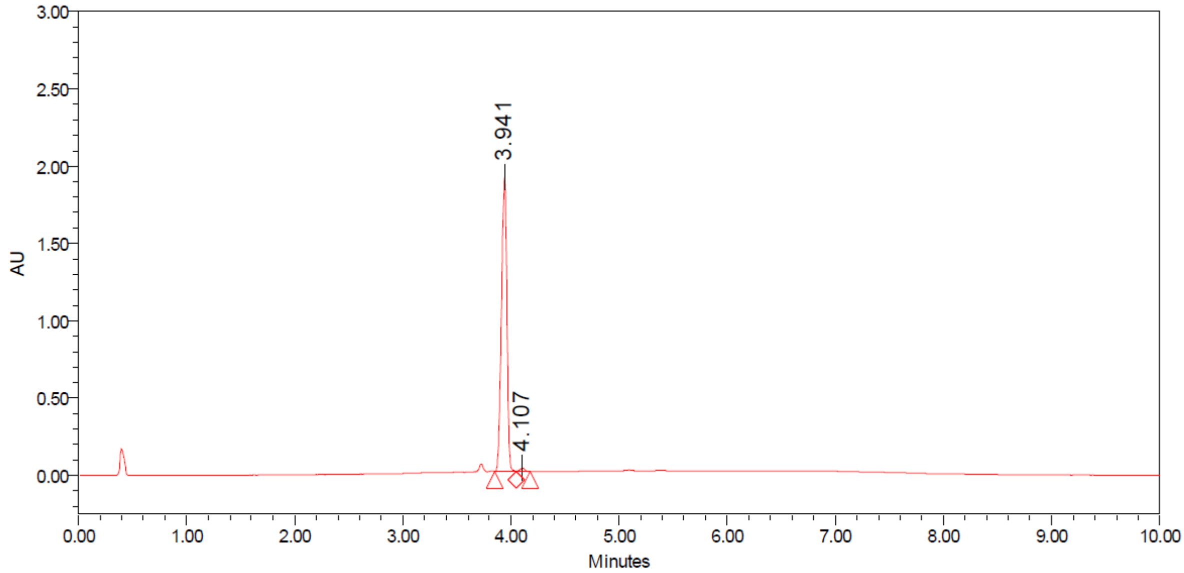

Peak Results

	RT	% Area
1	3.941	98.97
2	4.107	1.03

4-((*S*)-2-((*R*)-(3-formylfuran-2-yl)(phenyl)methyl)hexyl)-2-oxo-2*H*-chromene-3-carbonitrile minor – 3n

Racemic sample

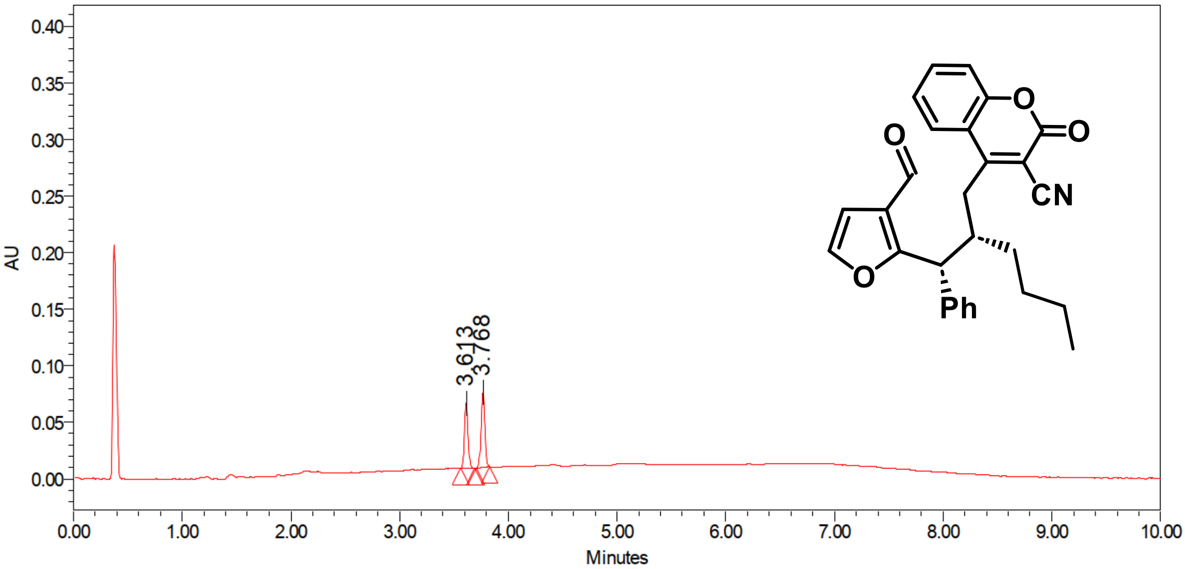

Peak Results

	RT	% Area
1	3.613	44.58
2	3.768	55.42

Enantiomerically enriched sample

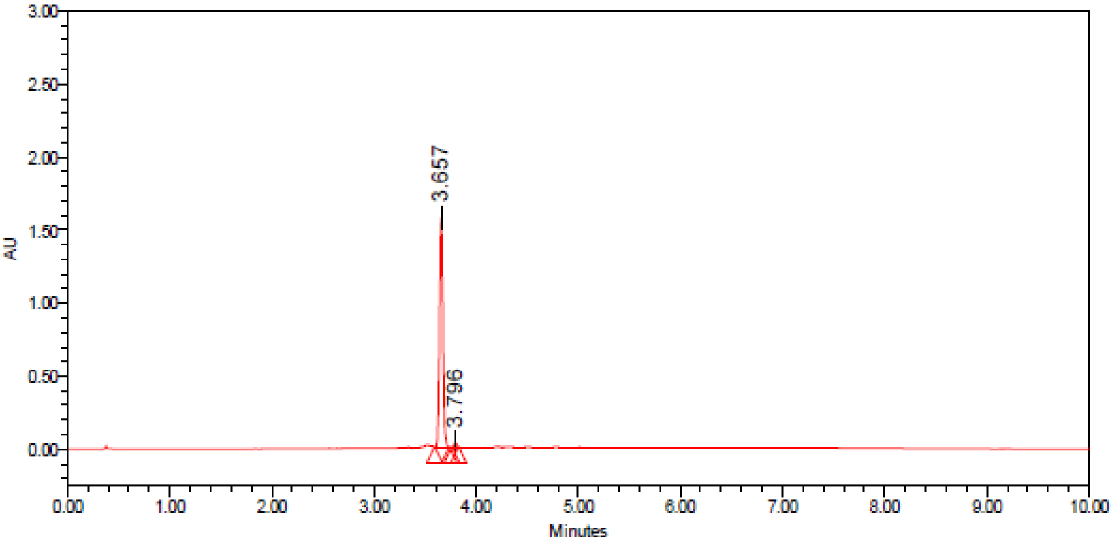

Peak Results

	RT	% Area
1	3.657	98.55
2	3.796	1.45

4-((2*R*,3*R*)-3-(3-formylfuran-2-yl)-2-phenyl-3-(3-(trifluoromethyl)phenyl)propyl)-2-oxo-2*H*-chromene-3-carbonitrile major – 3o

Racemic sample

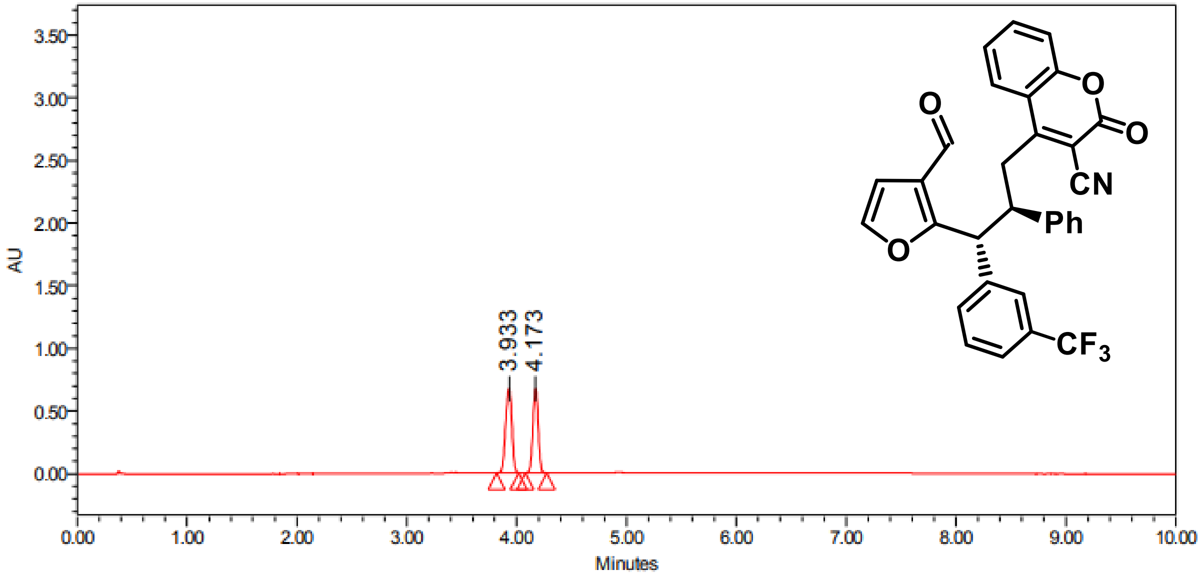

Peak Results

	RT	% Area
1	3.933	54.07
2	4.173	45.93

Enantiomerically enriched sample

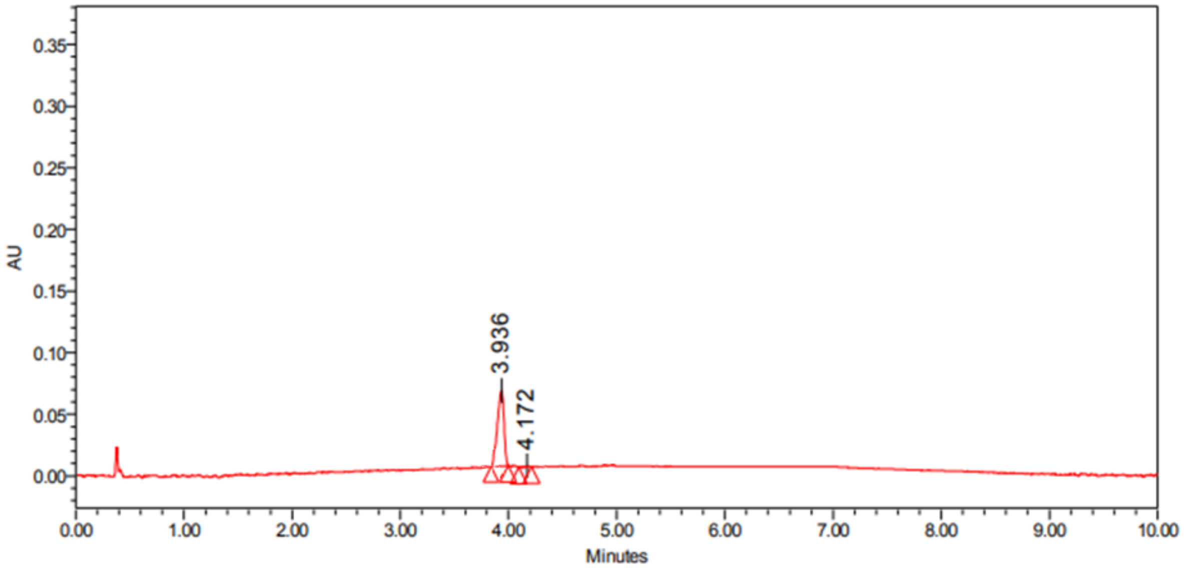

Peak Results

	RT	% Area
1	3.936	99.00
2	4.172	1.00

4-((2*S*,3*R*)-3-(3-formylfuran-2-yl)-2-phenyl-3-(3-(trifluoromethyl)phenyl)propyl)-2-oxo-2*H*-chromene-3-carbonitrile minor – 3o

Racemic sample

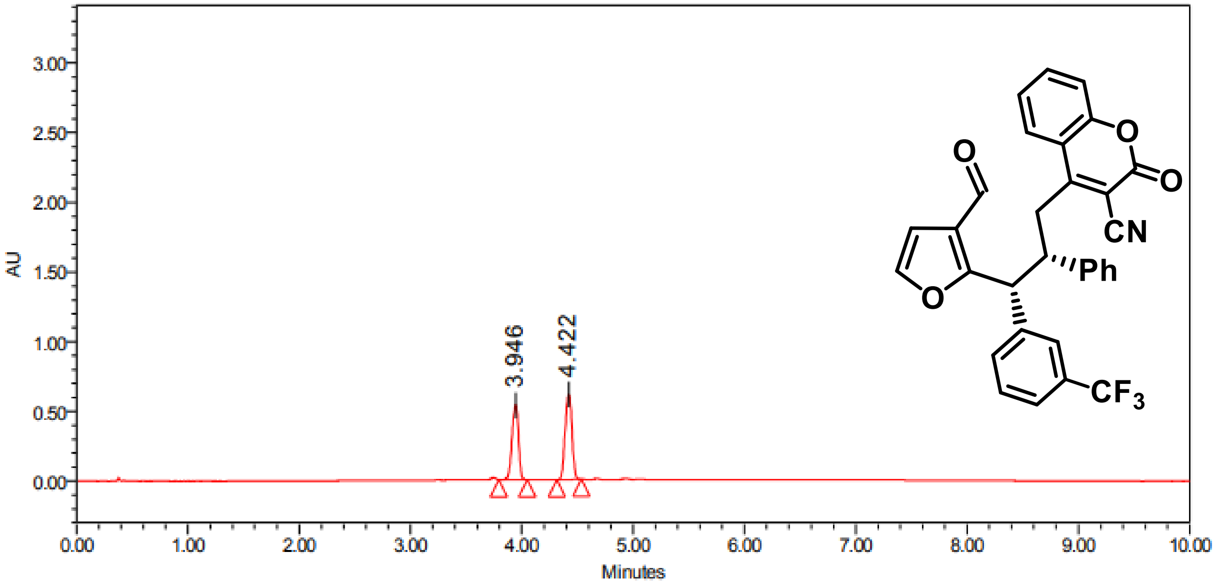

Peak Results

	RT	% Area
1	3.946	46.03
2	4.422	53.97

Enantiomerically enriched sample

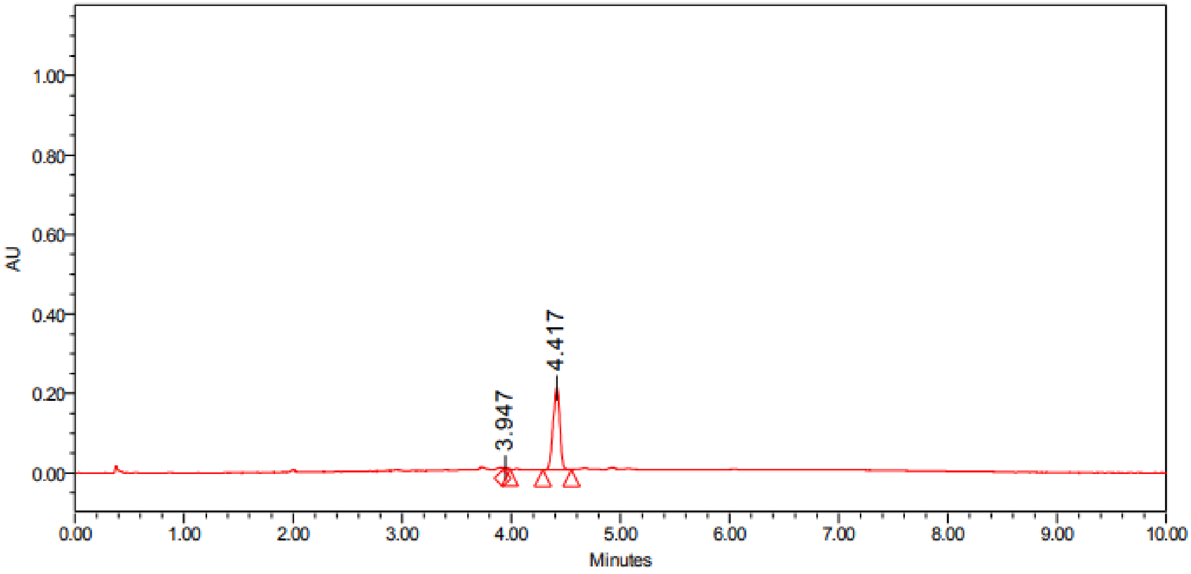

Peak Results

	RT	% Area
1	3.947	1.43
2	4.417	98.57

4-((2*R*,3*R*)-3-(4-fluorophenyl)-3-(3-formylfuran-2-yl)-2-phenylpropyl)-2-oxo-2*H*-chromene-3-carbonitrile major – 3p

Racemic sample

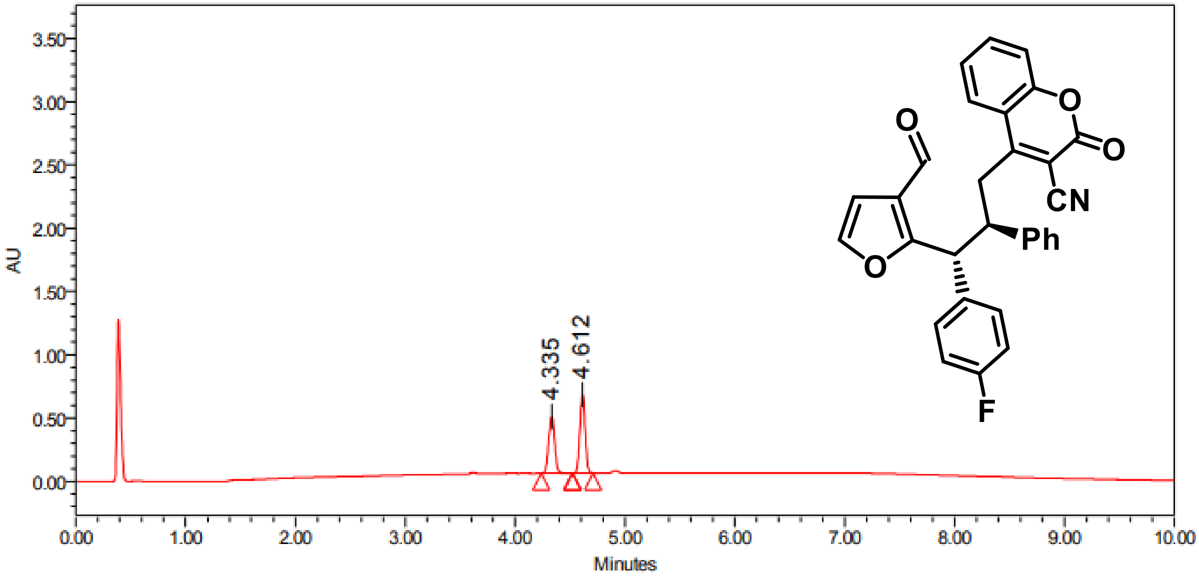

Peak Results

	RT	% Area
1	4.335	45.27
2	4.612	54.73

Enantiomerically enriched sample

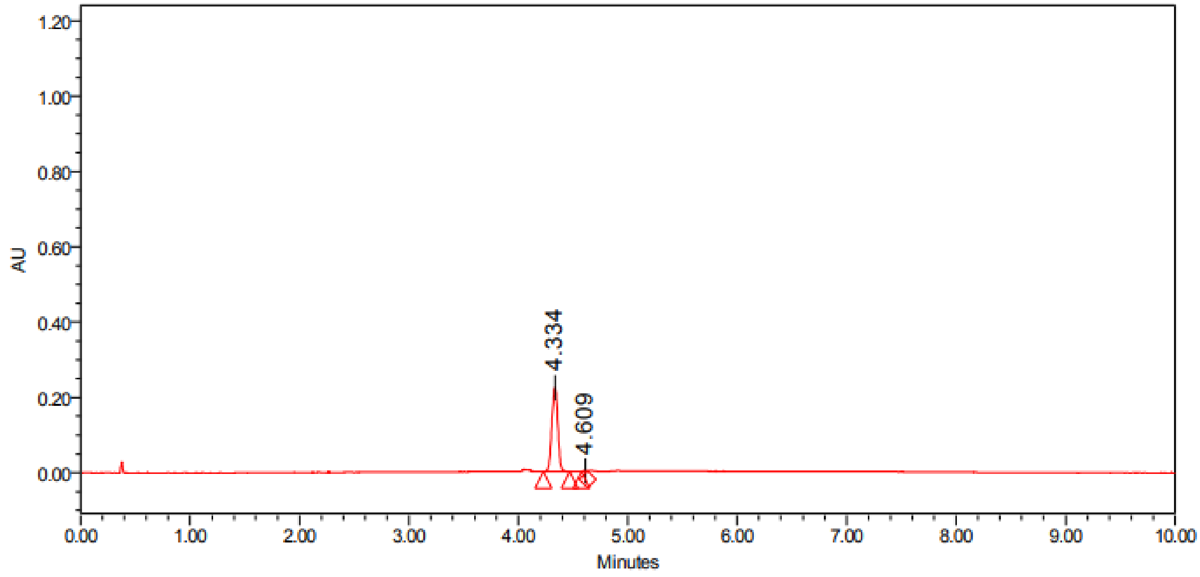

Peak Results

	RT	% Area
1	4.334	99.90
2	4.609	0.10

4-((2*S*,3*R*)-3-(4-fluorophenyl)-3-(3-formylfuran-2-yl)-2-phenylpropyl)-2-oxo-2*H*-chromene-3-carbonitrile minor – 3p

Racemic sample

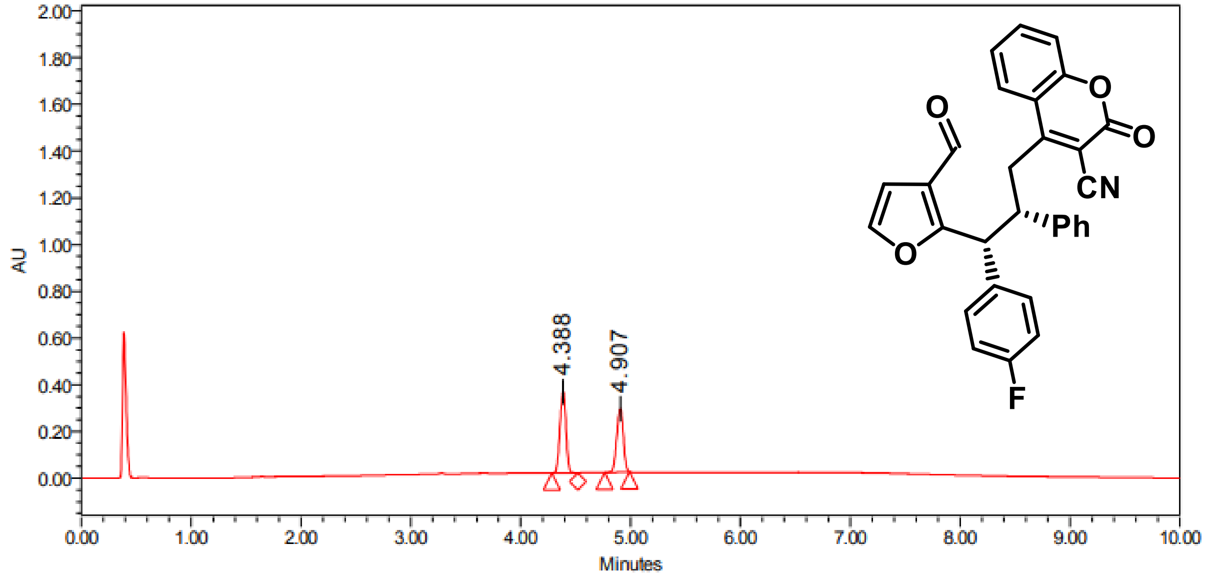

Peak Results

	RT	% Area
1	4.388	53.81
2	4.907	46.19

Enantiomerically enriched sample

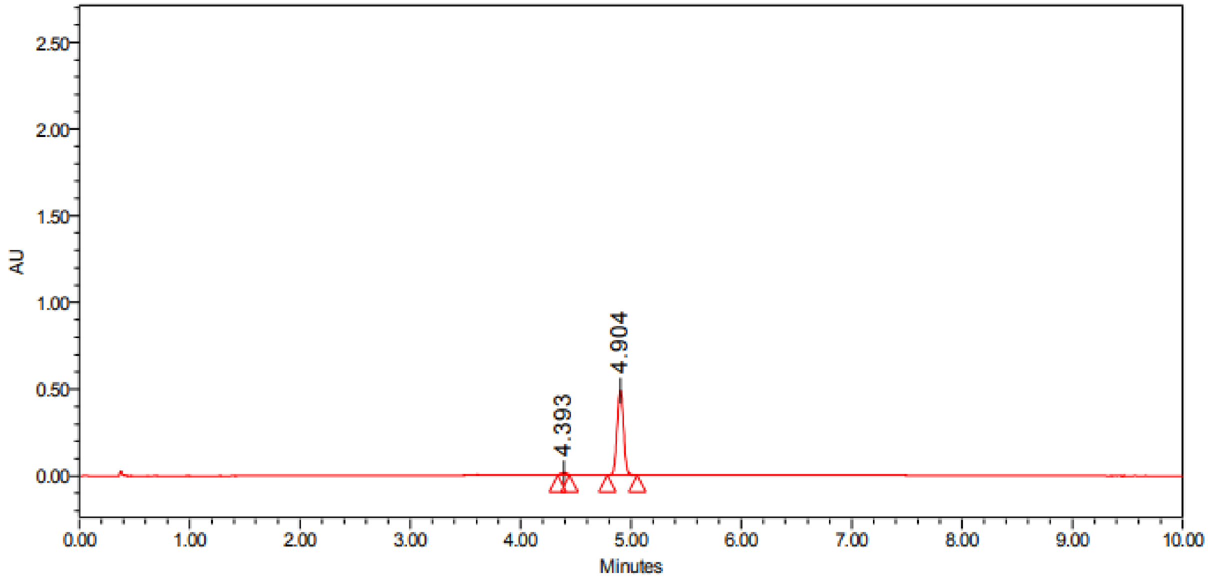

Peak Results

	RT	% Area
1	4.393	1.88
2	4.904	98.12

4-((2*R*,3*R*)-3-(3-formylfuran-2-yl)-2-phenyl-3-(*p*-tolyl)propyl)-2-oxo-2*H*-chromene-3-carbonitrile major – 3q

Racemic sample

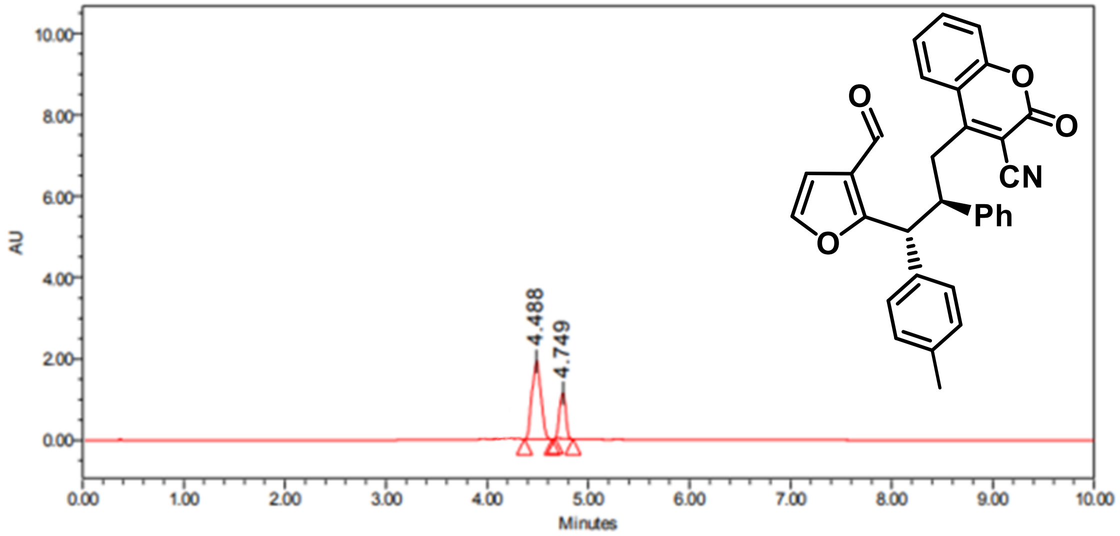

Peak Results

	RT	% Area
1	4.487	62.65
2	4.748	37.35

Enantiomerically enriched sample

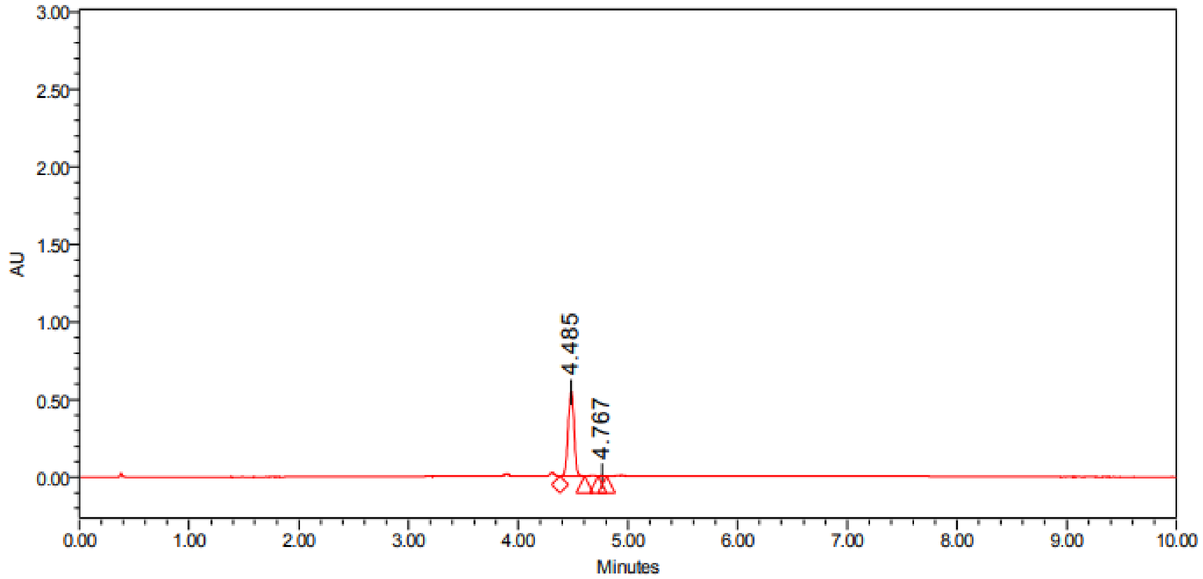

Peak Results

	RT	% Area
1	4.485	99.87
2	4.767	0.13

4-((2*S*,3*R*)-3-(3-formylfuran-2-yl)-2-phenyl-3-(*p*-tolyl)propyl)-2-oxo-2*H*-chromene-3-carbonitrile minor – 3q

Racemic sample

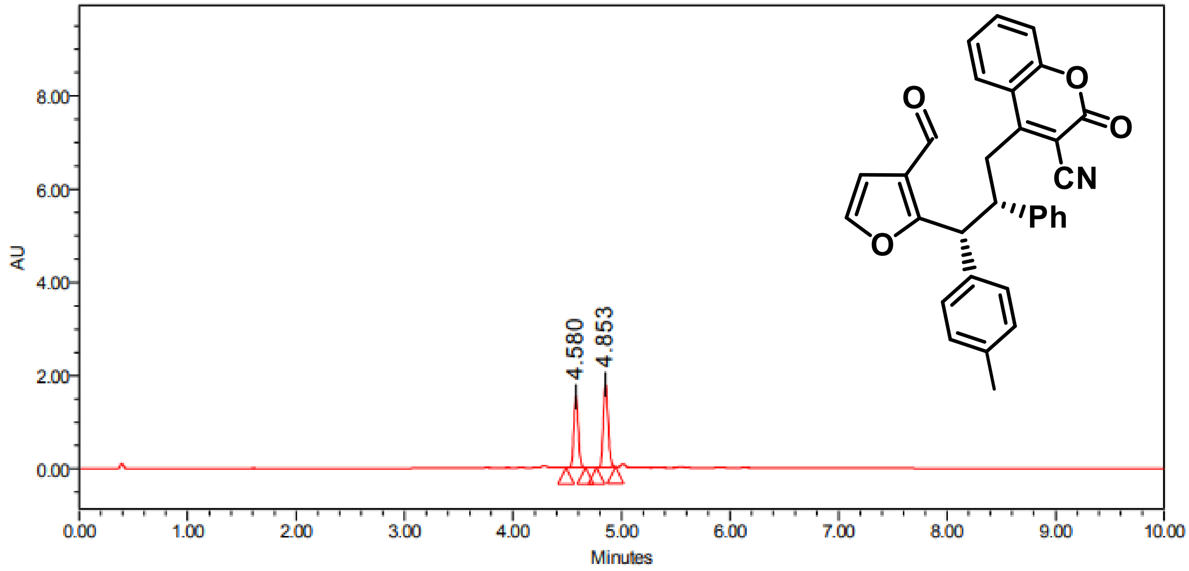

Peak Results

	RT	% Area
1	4.580	43.58
2	4.853	56.42

Enantiomerically enriched sample

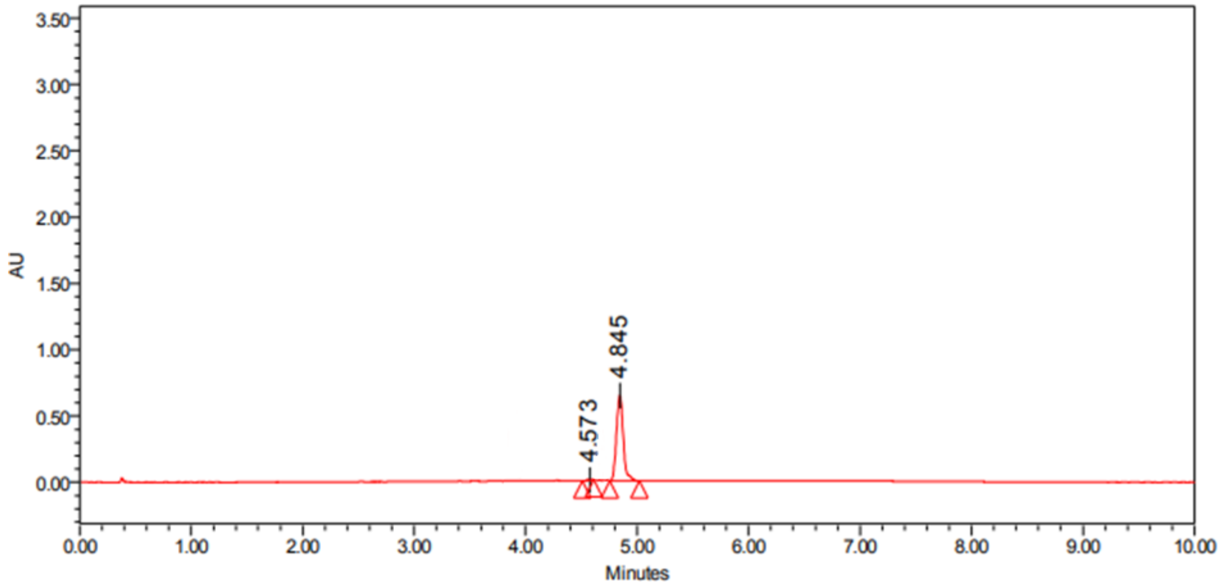

Peak Results

	RT	% Area
1	4.573	0.48
2	4.845	99.52

4-((2*R*,3*R*)-3-(3-formylfuran-2-yl)-3-(3-methoxyphenyl)-2-phenylpropyl)-2-oxo-2*H*-chromene-3-carbonitrile major – 3r

Racemic sample

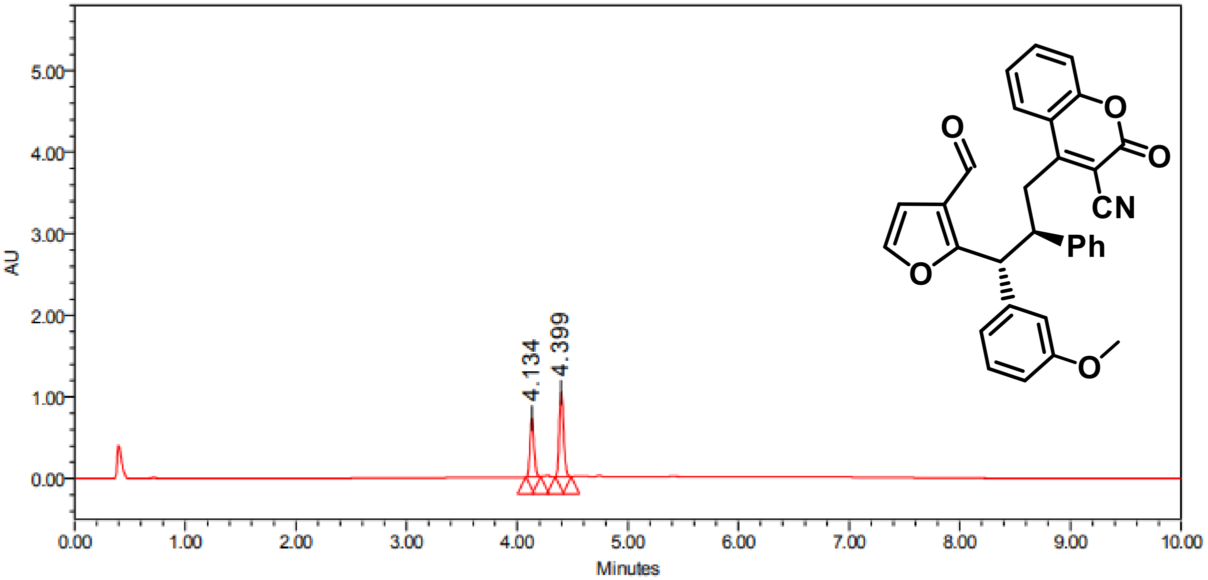

Peak Results

	RT	% Area
1	4.134	39.48
2	4.399	60.52

Enantiomerically enriched sample

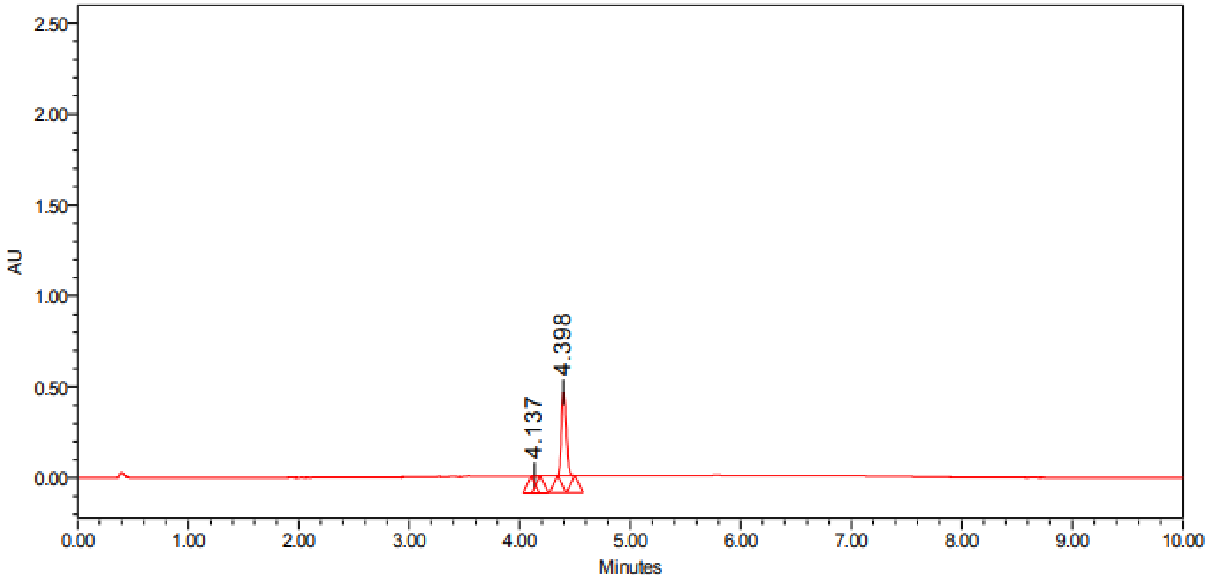

Peak Results

	RT	% Area
1	4.137	0.39
2	4.398	99.61

4-((2*S*,3*R*)-3-(3-formylfuran-2-yl)-3-(3-methoxyphenyl)-2-phenylpropyl)-2-oxo-2*H*-chromene-3-carbonitrile minor – 3r

Racemic sample

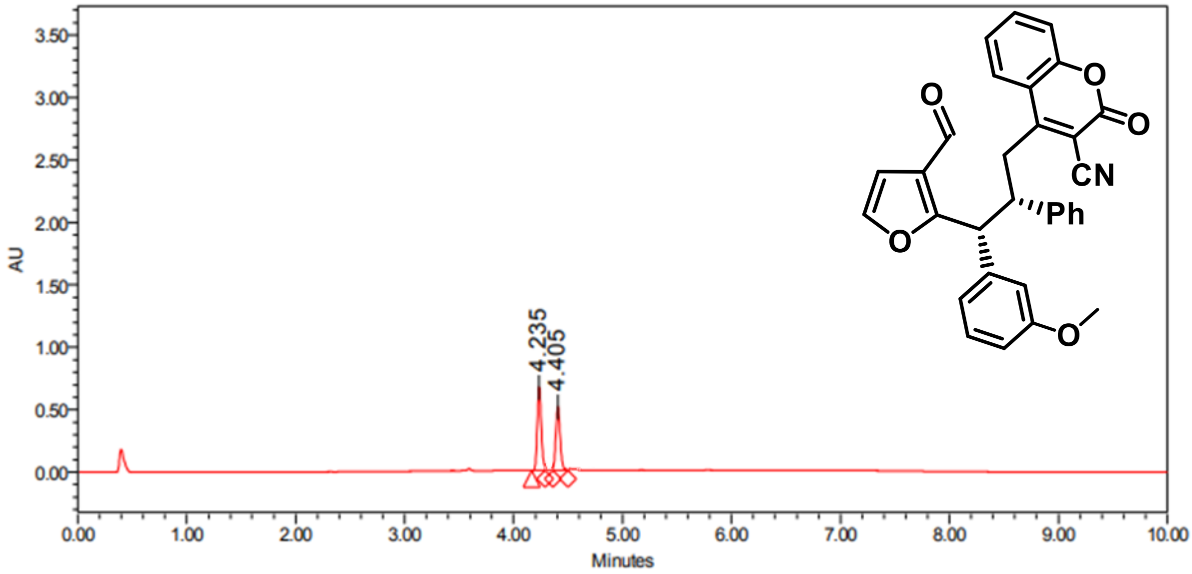

Peak Results

	RT	% Area
1	4.235	55.00
2	4.405	45.00

Enantiomerically enriched sample

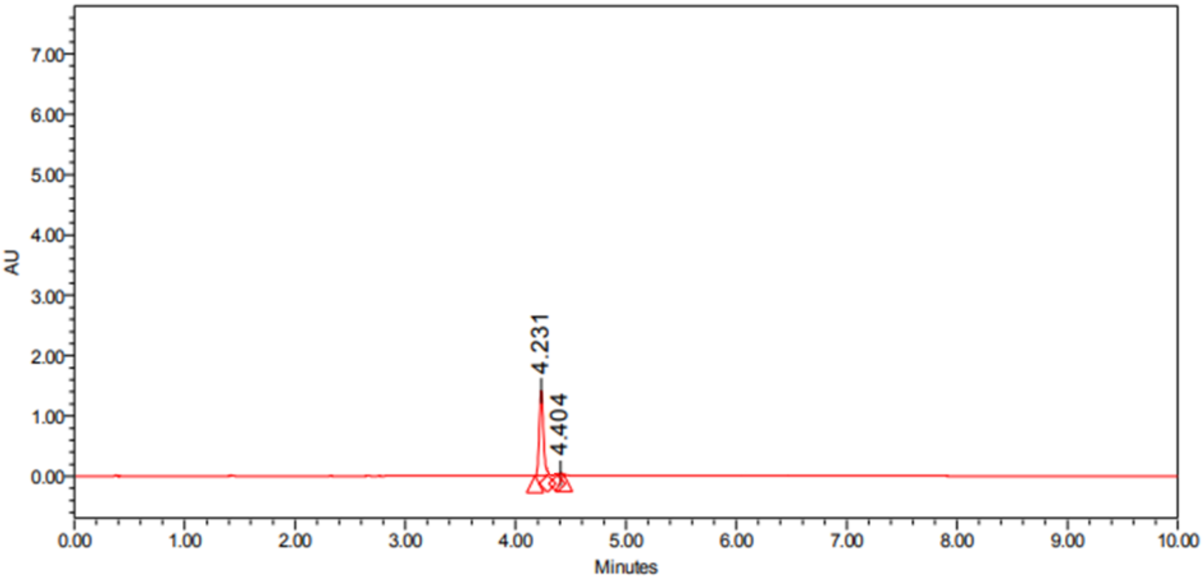

Peak Results

	RT	% Area
1	4.231	98.28
2	4.404	1.72

4-((2*R*,3*R*)-3-(5-formylfuran-2-yl)-2,3-diphenylpropyl)-2-oxo-2*H*-chromene-3-carbonitrile major – 3s

Racemic sample

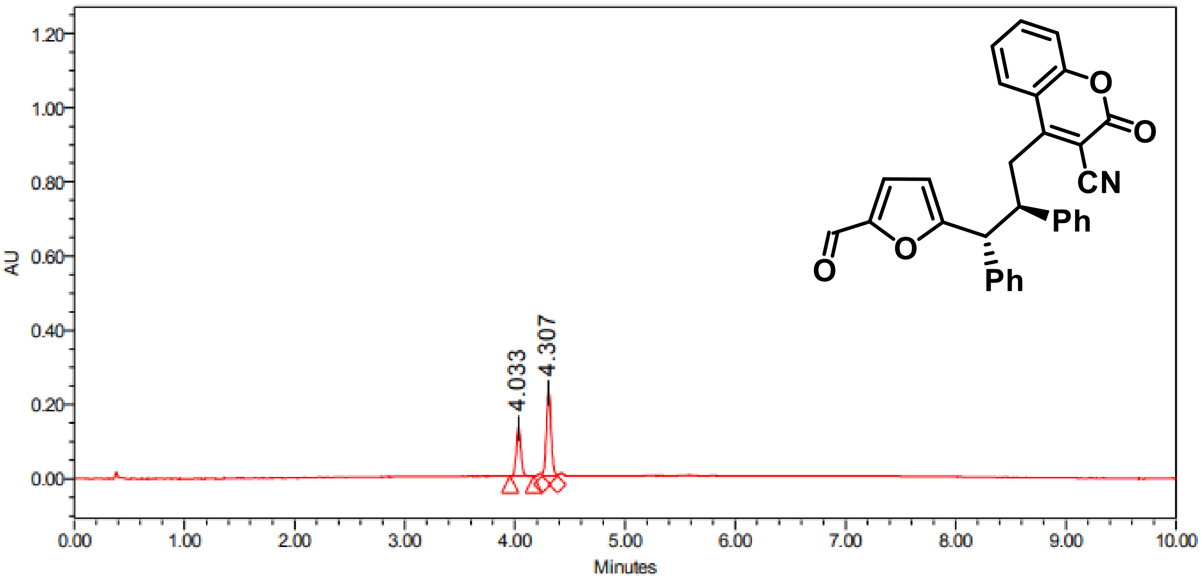

Peak Results

	RT	% Area
1	4.033	36.71
2	4.307	63.29

Enantiomerically enriched sample

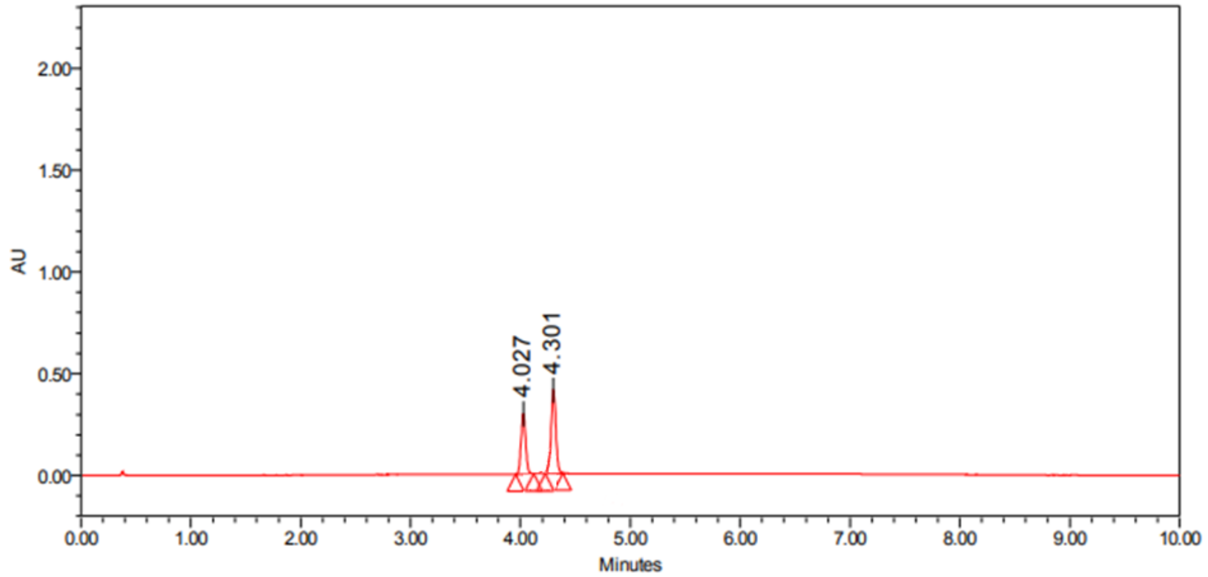

Peak Results

	RT	% Area
1	4.027	41.98
2	4.301	58.02

4-((2*S*,3*R*)-3-(5-formylfuran-2-yl)-2,3-diphenylpropyl)-2-oxo-2*H*-chromene-3-carbonitrile minor – 3s

Racemic sample

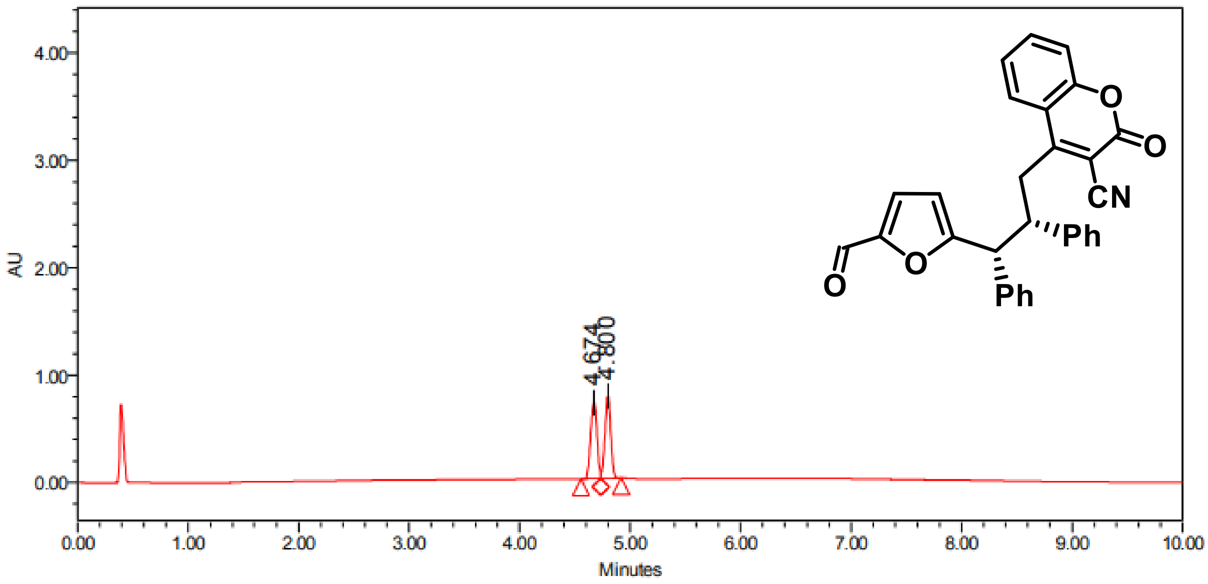

Peak Results

	RT	% Area
1	4.674	50.08
2	4.800	49.92

Enantiomerically enriched sample

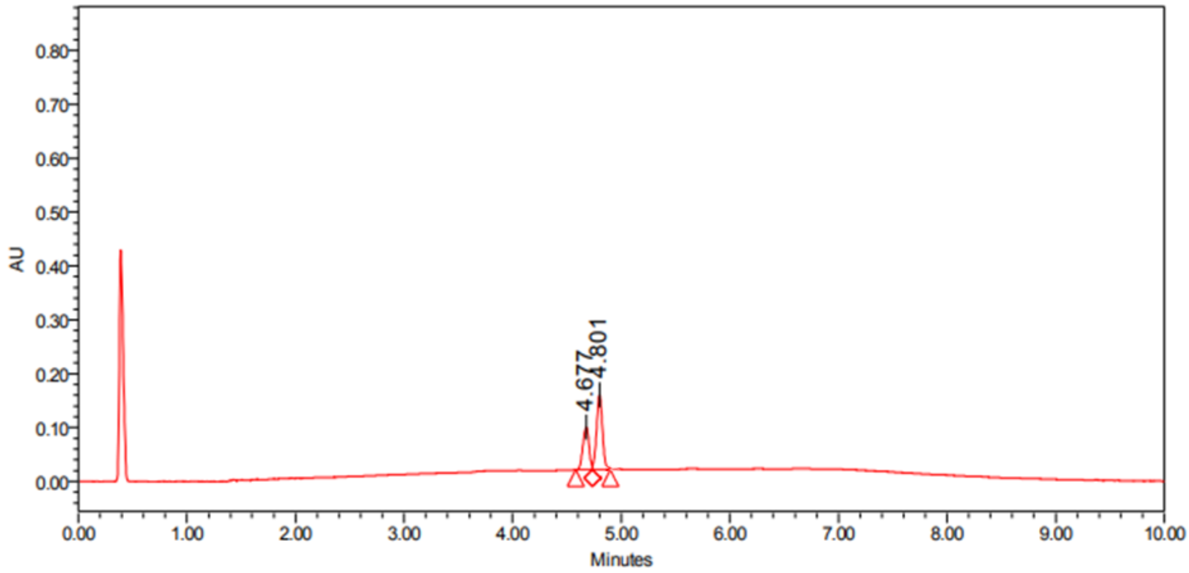

Peak Results

	RT	% Area
1	4.677	37.49
2	4.801	62.51
